# The First Myriapod Genome Sequence Reveals Conservative Arthropod Gene Content and Genome Organisation in the Centipede *Strigamia maritima*

**DOI:** 10.1371/journal.pbio.1002005

**Published:** 2014-11-25

**Authors:** Ariel D. Chipman, David E. K. Ferrier, Carlo Brena, Jiaxin Qu, Daniel S. T. Hughes, Reinhard Schröder, Montserrat Torres-Oliva, Nadia Znassi, Huaiyang Jiang, Francisca C. Almeida, Claudio R. Alonso, Zivkos Apostolou, Peshtewani Aqrawi, Wallace Arthur, Jennifer C. J. Barna, Kerstin P. Blankenburg, Daniela Brites, Salvador Capella-Gutiérrez, Marcus Coyle, Peter K. Dearden, Louis Du Pasquier, Elizabeth J. Duncan, Dieter Ebert, Cornelius Eibner, Galina Erikson, Peter D. Evans, Cassandra G. Extavour, Liezl Francisco, Toni Gabaldón, William J. Gillis, Elizabeth A. Goodwin-Horn, Jack E. Green, Sam Griffiths-Jones, Cornelis J. P. Grimmelikhuijzen, Sai Gubbala, Roderic Guigó, Yi Han, Frank Hauser, Paul Havlak, Luke Hayden, Sophie Helbing, Michael Holder, Jerome H. L. Hui, Julia P. Hunn, Vera S. Hunnekuhl, LaRonda Jackson, Mehwish Javaid, Shalini N. Jhangiani, Francis M. Jiggins, Tamsin E. Jones, Tobias S. Kaiser, Divya Kalra, Nathan J. Kenny, Viktoriya Korchina, Christie L. Kovar, F. Bernhard Kraus, François Lapraz, Sandra L. Lee, Jie Lv, Christigale Mandapat, Gerard Manning, Marco Mariotti, Robert Mata, Tittu Mathew, Tobias Neumann, Irene Newsham, Dinh N. Ngo, Maria Ninova, Geoffrey Okwuonu, Fiona Ongeri, William J. Palmer, Shobha Patil, Pedro Patraquim, Christopher Pham, Ling-Ling Pu, Nicholas H. Putman, Catherine Rabouille, Olivia Mendivil Ramos, Adelaide C. Rhodes, Helen E. Robertson, Hugh M. Robertson, Matthew Ronshaugen, Julio Rozas, Nehad Saada, Alejandro Sánchez-Gracia, Steven E. Scherer, Andrew M. Schurko, Kenneth W. Siggens, DeNard Simmons, Anna Stief, Eckart Stolle, Maximilian J. Telford, Kristin Tessmar-Raible, Rebecca Thornton, Maurijn van der Zee, Arndt von Haeseler, James M. Williams, Judith H. Willis, Yuanqing Wu, Xiaoyan Zou, Daniel Lawson, Donna M. Muzny, Kim C. Worley, Richard A. Gibbs, Michael Akam, Stephen Richards

**Affiliations:** 1 The Department of Ecology, Evolution and Behavior, The Alexander Silberman Institute of Life Sciences, The Hebrew University of Jerusalem, Givat Ram, Jerusalem, Israel; 2 The Scottish Oceans Institute, Gatty Marine Laboratory, University of St Andrews, St Andrews, Fife, United Kingdom; 3 Department of Zoology, University of Cambridge, Cambridge, United Kingdom; 4 Human Genome Sequencing Center, Department of Molecular and Human Genetics, Baylor College of Medicine, Houston, Texas, United States of America; 5 EMBL - European Bioinformatics Institute, Hinxton, Cambridgeshire, United Kingdom; 6 Institut für Biowissenschaften, Universität Rostock, Abt. Genetik, Rostock, Germany; 7 Departament de Genètica and Institut de Recerca de la Biodiversitat (IRBio), Universitat de Barcelona, Barcelona, Spain; 8 Consejo Nacional de Investigaciones Científicas y Tecnológicas (CONICET), Universidad Nacional de Tucumán, Facultad de Ciencias Naturales e Instituto Miguel Lillo, San Miguel de Tucumán, Argentina; 9 School of Life Sciences, University of Sussex, Brighton, United Kingdom; 10 Institute of Molecular Biology & Biotechnology, Foundation for Research & Technology - Hellas, Heraklion, Crete, Greece; 11 Department of Zoology, National University of Ireland, Galway, Ireland; 12 Department of Biochemistry, University of Cambridge, Cambridge, United Kingdom; 13 Evolutionsbiologie, Zoologisches Institut, Universität Basel, Basel, Switzerland; 14 Swiss Tropical and Public Health Institute, Basel, Switzerland; 15 Centre for Genomic Regulation, Barcelona, Barcelona, Spain; 16 Gravida and Genetics Otago, Biochemistry Department, University of Otago, Dunedin, New Zealand; 17 Razavi Newman Center for Bioinformatics, Salk Institute, La Jolla, California, United States of America; 18 Scripps Translational Science Institute, La Jolla, California, United States of America; 19 The Babraham Institute, Cambridge, United Kingdom; 20 Department of Organismic and Evolutionary Biology, Harvard University, Cambridge, Massachusetts, United States of America; 21 Universitat Pompeu Fabra (UPF), Barcelona, Spain; 22 Institució Catalana de Recerca i Estudis Avançats (ICREA), Barcelona, Spain; 23 Department of Biochemistry and Cell Biology, Center for Developmental Genetics, Stony Brook University, Stony Brook, New York, United States of America; 24 Department of Biology, Hendrix College, Conway, Arkansas, United States of America; 25 Faculty of Life Sciences, University of Manchester, Manchester, United Kingdom; 26 Center for Functional and Comparative Insect Genomics, University of Copenhagen, Copenhagen, Denmark; 27 Center for Genomic Regulation, Barcelona, Spain; 28 Department of Ecology and Evolutionary Biology, Rice University, Houston, Texas, United States of America; 29 Institut für Biologie, Martin-Luther-Universität Halle-Wittenberg, Halle, Germany; 30 School of Life Sciences, The Chinese University of Hong Kong, Shatin, NT, Hong Kong SAR, China; 31 Department of Biochemistry and Cell Biology, Faculty of Veterinary Medicine, Utrecht University, Utrecht, The Netherlands; 32 Department of Genetics, University of Cambridge, Cambridge, United Kingdom; 33 Max F. Perutz Laboratories, University of Vienna, Vienna, Austria; 34 Department of Laboratory Medicine, University Hospital Halle (Saale), Halle (Saale), Germany; 35 Department of Genetics, Evolution and Environment, University College London, London, United Kingdom; 36 Center for Integrative Bioinformatics Vienna, Max F. Perutz Laboratories, University of Vienna, Medical University of Vienna, Vienna, Austria; 37 Hubrecht Institute for Developmental Biology and Stem Cell Research, Utrecht, The Netherlands; 38 Harte Research Institute, Texas A&M University Corpus Christi, Corpus Christi, Texas, United States of America; 39 Department of Entomology, University of Illinois at Urbana-Champaign, Urbana, Illinois, United States of America; 40 Institute for Biochemistry and Biology, University Potsdam, Potsdam-Golm, Germany; 41 Research Platform “Marine Rhythms of Life”, Vienna, Austria; 42 Institute of Biology, Leiden University, Leiden, The Netherlands; 43 Bioinformatics and Computational Biology, Faculty of Computer Science, University of Vienna, Vienna, Austria; 44 Department of Cellular Biology, University of Georgia, Athens, Georgia, United States of America; The Wellcome Trust Sanger Institute, United Kingdom

## Abstract

Myriapods (e.g., centipedes and millipedes) display a simple homonomous body plan relative to other arthropods. All members of the class are terrestrial, but they attained terrestriality independently of insects. Myriapoda is the only arthropod class not represented by a sequenced genome. We present an analysis of the genome of the centipede *Strigamia maritima*. It retains a compact genome that has undergone less gene loss and shuffling than previously sequenced arthropods, and many orthologues of genes conserved from the bilaterian ancestor that have been lost in insects. Our analysis locates many genes in conserved macro-synteny contexts, and many small-scale examples of gene clustering. We describe several examples where *S. maritima* shows different solutions from insects to similar problems. The insect olfactory receptor gene family is absent from *S. maritima*, and olfaction in air is likely effected by expansion of other receptor gene families. For some genes *S. maritima* has evolved paralogues to generate coding sequence diversity, where insects use alternate splicing. This is most striking for the *Dscam* gene, which in *Drosophila* generates more than 100,000 alternate splice forms, but in *S. maritima* is encoded by over 100 paralogues. We see an intriguing linkage between the absence of any known photosensory proteins in a blind organism and the additional absence of canonical circadian clock genes. The phylogenetic position of myriapods allows us to identify where in arthropod phylogeny several particular molecular mechanisms and traits emerged. For example, we conclude that juvenile hormone signalling evolved with the emergence of the exoskeleton in the arthropods and that RR-1 containing cuticle proteins evolved in the lineage leading to Mandibulata. We also identify when various gene expansions and losses occurred. The genome of *S. maritima* offers us a unique glimpse into the ancestral arthropod genome, while also displaying many adaptations to its specific life history.

## Author Summary

Arthropods are the most abundant animals on earth. Among them, insects clearly dominate on land, whereas crustaceans hold the title for the most diverse invertebrates in the oceans. Much is known about the biology of these groups, not least because of genomic studies of the fruit fly *Drosophila*, the water flea *Daphnia*, and other species used in research. Here we report the first genome sequence from a species belonging to a lineage that has previously received very little attention—the myriapods. Myriapods were among the first arthropods to invade the land over 400 million years ago, and survive today as the herbivorous millipedes and venomous centipedes, one of which—*Strigamia maritima*—we have sequenced here. We find that the genome of this centipede retains more characteristics of the presumed arthropod ancestor than other sequenced insect genomes. The genome provides access to many aspects of myriapod biology that have not been studied before, suggesting, for example, that they have diversified receptors for smell that are quite different from those used by insects. In addition, it shows specific consequences of the largely subterranean life of this particular species, which seems to have lost the genes for all known light-sensing molecules, even though it still avoids light.

## Introduction

**Figure 1:**
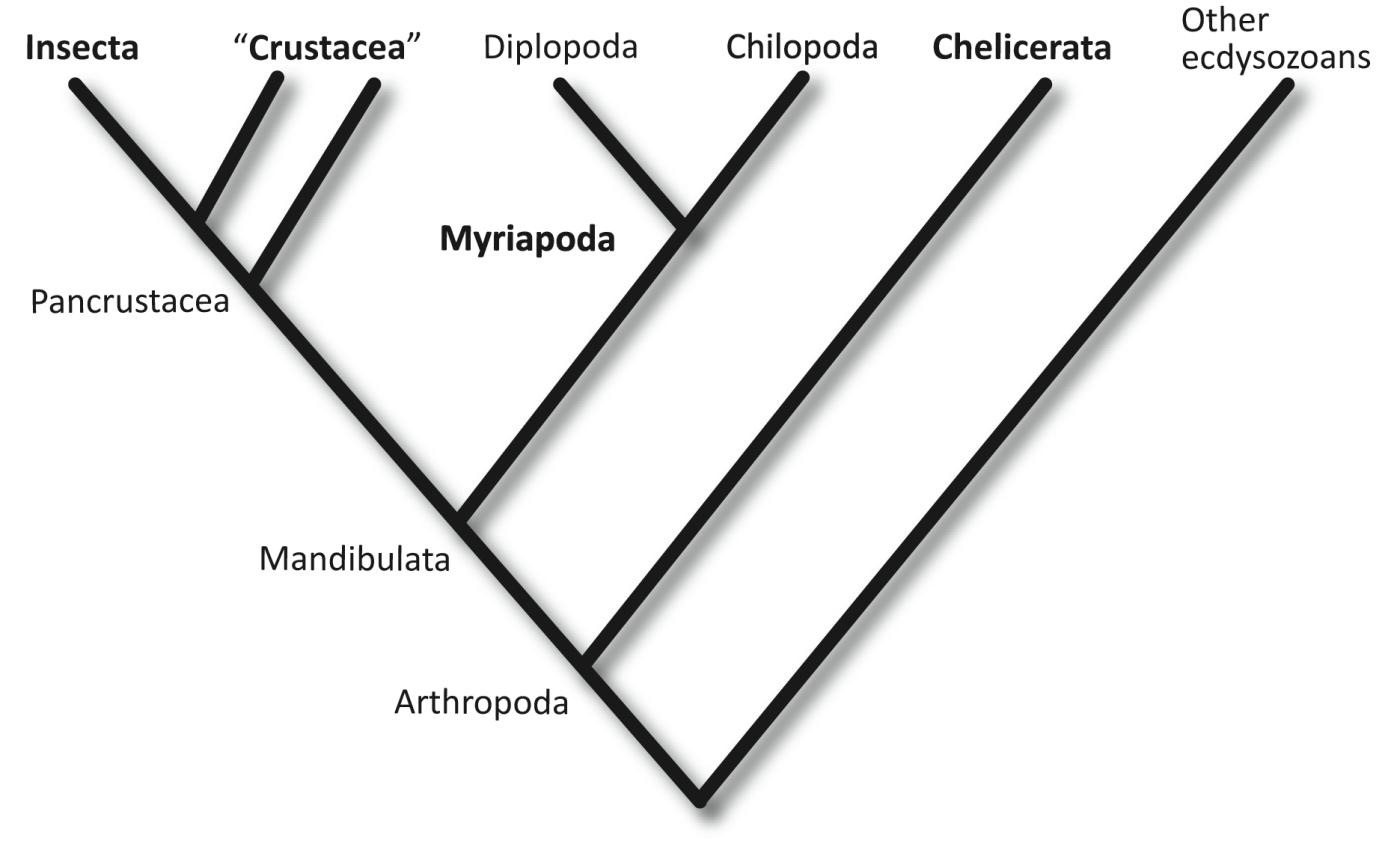
The phylogenetic position of the centipedes (Chilopoda), with respect to other arthropods, according to the currently best-supported phylogeny. (See text for details). The four traditionally accepted arthropod classes are marked in bold.

Arthropods are the most species-rich animal phylum on Earth. Of the four extant classes of arthropods (Insecta, Crustacea, Myriapoda, and Chelicerata) (Figure 1), only the Myriapoda (centipedes, millipedes, and their relatives) are currently not represented by any sequenced genome [1],[2]. This absence is particularly unfortunate, as myriapods have recently been recognised as the living sister group to the clade that encompasses all insects and crustaceans [3]–[6]. Hence, the Myriapoda are particularly well placed to provide an outgroup for comparison, to determine ancestral character states and the polarity of evolutionary change within insects and crustaceans, which together represent the most diverse animal clade on Earth.

Although *Drosophila melanogaster* is the best studied arthropod, it lacks many genes present in the ancestral bilaterian gene set, and chromosome rearrangements have disrupted all obvious evidence of synteny with other phyla [7]. Thus it is not fully representative of other arthropods. More comprehensive sampling of arthropod genomes will establish their basic structure, and determine when unique genomic characteristics of different taxa, such as the holometabolous insects, appear.

### Phylogenetic Position of the Myriapods

Myriapods are today represented by two major lineages—the herbivorous millipedes (Diplopoda) and the carnivorous centipedes (Chilopoda), together with two minor clades, the Symphyla, which look superficially like small white centipedes, and the minute Pauropoda [8]. All are characterised by a multi-segmented trunk of rather similar (homonomous) segments, with no differentiation into thorax or abdomen. All recent studies, molecular and morphological, support the monophyly of myriapods [3]–[5],[8]–[10] suggesting that they share a single common ancestor.

Myriapods, insects, and crustaceans have traditionally been identified as a clade of mandibulate arthropods, characterised by head appendages that include antennae and biting jaws [11]. Some molecular datasets have challenged this idea, suggesting instead that the myriapods are a sister group to the chelicerates [12],[13]. The most comprehensive phylogenomic datasets thus far reject this, and strongly support the phylogeny that proposes that the chelicerates are the most basal of the four major extant arthropod clades, and the mandibulates represent a true monophyletic group [3],[5],[10],[14]–[17].

Within the mandibulates, myriapods were believed until recently to share a common origin with insects as terrestrial arthropods. This view, based on a number of shared characters including uniramous limbs, air breathing through tracheae, the lack of a second pair of antennae, and excretion using Malpighian tubules, was widely supported by morphologically based phylogenies [9],[18]. However, molecular phylogenies robustly reject the sister group relationship between insects and myriapods, placing the origin of myriapods basal to the diversification of crustaceans [5], and identifying insects as a derived clade within the Crustacea [19]–[21]. As crustaceans are overwhelmingly a marine group today, and were so ancestrally, this implies that myriapods and insects represent independent invasions of the land (with the chelicerates representing an additional, unrelated invasion). Their shared characteristics are striking convergences, not synapomorphies.

### *S. maritima* as a Model Myriapod

We chose *S. maritima* as the species to sequence partly for pragmatic reasons: geophilomorph centipedes, such as *S. maritima*, have relatively small genome sizes, certainly compared to other centipedes [22]. More importantly, it is a species that has attracted interest for ecological and developmental studies [23]–[25], especially the process of segment patterning [26]–[32]. *S. maritima* is a common centipede of north western Europe, found along the coastline from France to the middle of Norway. It is a specialist of shingle beaches and rocky shores, occurring around the high tide mark, and feeding on the abundant crustaceans and insect larvae associated with the strand line. It is by far the most abundant centipede in these habitats around the British Isles, sometimes occurring at densities of thousands per square metre in suitable locations [25]. Eggs can be harvested from these abundant populations in large numbers with relatively little effort during the summer breeding season [27]. They can be reared in the lab from egg lay to at least the first free-living stage, adolescens I [24],[33].

Some aspects of *S. maritima* biology are not common to all centipedes. Notable among these is epimorphic development, wherein the embryos hatch from the egg with the final adult number of leg-bearing segments. Epimorphic development is found in two centipede orders: geophilomorphs (including *S. maritima*) and scolopendromorphs. In contrast, more basal clades display anamorphic development and add segments post-embryonically [34]. These anamorphic clades have relatively few leg-bearing segments, generally 15, while geophilomorphs have many more, up to nearly 200 in some species [6]. These unique characteristics probably arose at least 300 million years ago, as the earliest fossils of the much larger scolopendromorph centipedes date to the Upper Carboniferous [35]. These share the same mode of development as the geophilomorphs, and are their likely sister group. Geophilomorphs are also adapted to a subsurface life style, the whole order having lost all trace of eyes [36],[37], though apparently not photosensitivity [38].

We have sequenced the genome of *S. maritima* as a representative of the phylogenetically important myriapods. In contrast to the intensively sampled holometabolous insects, our analysis of this myriapod genome finds conservative gene sets and conserved synteny, shedding light on general genomic features of the arthropods.

## Results and Discussion

### Genome Assembly, Gene Densities, and Polymorphism

Genomic DNA from multiple individuals of a wild Scottish population of *S. maritima* was sequenced and assembled into a draft genome sequence spanning 176.2 Mb. This assembled sequence omits many repeat sequences including heterochromatin, which probably accounts for the difference between the assembly length and the total genome size estimate of 290 Mb. An analysis of repetitive elements within the assembly is presented in Text S1.

The assembly incorporates 14,992 automatically generated gene models, 1,095 of which have been additionally manually annotated. We re-sequenced four individuals comprising three females and one male. The frequency of identified polymorphism, with SNP density of 4.5 variants/kb, is comparable with the five variants per kb in the *Drosophila* genetic reference panel [39]. It is hard to say how typical this is for soil dwelling arthropods, as very little population data are available for such species.

### Phylome Analysis and Phylogenomics

To understand general patterns of gene evolution in *S. maritima* we reconstructed the evolutionary histories of all of its genes, i.e., the phylome. The resulting gene phylogenies, available through phylomeDB [40], were analysed to establish orthology and paralogy relationships with other arthropod genomes [41], transfer functional knowledge from annotated orthologues, and to detect and date gene duplication events [42]. Some 32% of *S. maritima* genes can be traced back to duplications specific to this myriapod lineage since its divergence from other arthropod groups included in the analysis. Functions enriched among these genes include those related to, among other processes, catabolism of peptidoglycans, sodium transport, glutamate receptor, and sensory perception of taste. Related to this latter function, two of the largest gene expansions specific to the *S. maritima* lineage detected in our analysis are the gustatory receptor (GR) and ionotropic receptor (IR) families encoding putative membrane-associated gustatory and/or olfactory receptors (see Text S1, and Chemosensory section below).

### Sex Chromosomes

**Figure 2:**
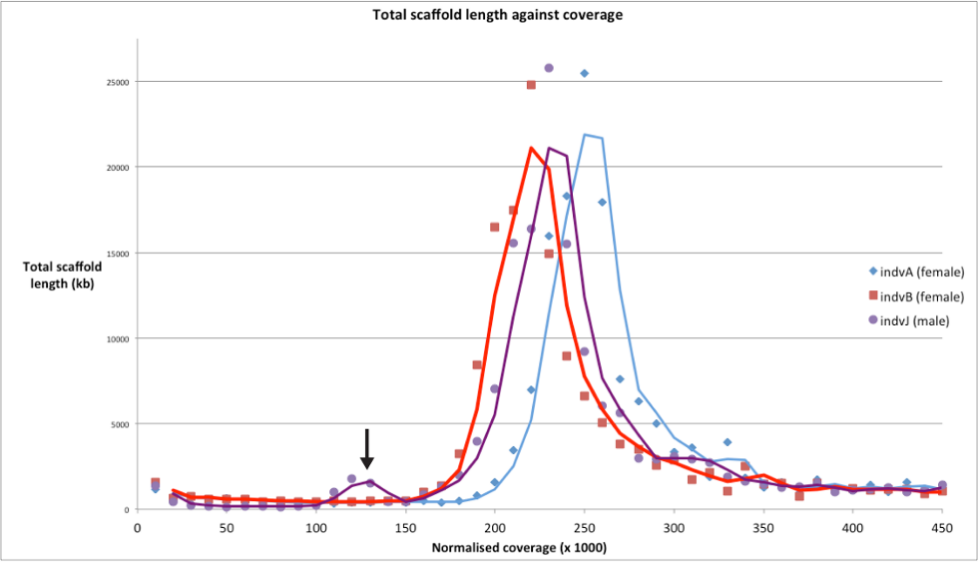
Plot showing that DNA from a male individual contains a distinct fraction of scaffolds that is underrepresented (black arrow), and presumably derives from heterogametic sex chromosomes. No such fraction is present in the sequenced DNA of two individual females. The data underlying this plot is presented in File S4.

No obviously differentiated sex chromosomes are apparent in the diploid *S. maritima* karyotype, which comprises one long pair of metacentric chromosomes, together with seven pairs of much shorter telocentric chromosomes (P. Woznicki, unpublished data; J. Green et al., unpublished). Read-depth data from the genome assembly show that a proportion of the genome is underrepresented compared to the bulk of the data. One obvious reason for underrepresentation would be sequences derived from sex chromosomes. To confirm this, the coverage of individual scaffolds from the assembly was examined in sequence obtained from single individuals. A distinct fraction of underrepresented scaffolds is present in DNA derived from a male, but absent in female sequence (Figure 2), implying an XY sex determination mechanism. Quantitative PCR from three scaffolds in the underrepresented fraction confirmed that they are present at approximately twice the copy number in females as in males, identifying them as X chromosome derived (J. Green et al., unpublished). Other scaffolds of this fraction contain male specific sequences, and therefore presumably derive from a Y chromosome (J. Green et al., unpublished) [31]. Combined with the karyotype data, this finding suggests that *S. maritima* possesses a weakly differentiated pair of X and Y chromosomes.

### Mitochondrial Genome

From the whole genome assembly, *S. maritima* scaffold scf7180001247661 was found to contain a complete copy of the mitochondrial coding regions, flanked by a TY1/Copia-like retrotransposon, which all together spanned approximately 20 kb. This is unusually large for a metazoan mitochondrial genome and, as mis-assembly was suspected, PCR was used to clone the DNA between the genes at either end of the scaffold. This enabled us to close the circle of the mitochondrial genome, correct frameshifts, and confirm an unusual gene arrangement, resulting in a final circular assembly of 14,983 bp (Table S11). The gene arrangement in the *S. maritima* mitochondrial genome is striking (Figure S6). It diverges dramatically from the basic arthropod genome arrangement and differs from all other known centipede mitochondrial gene arrangements [43]. Although small sections of the *S. maritima* gene order are conserved with respect to the arthropod ground pattern found in *Limulus polyphemus* and the lithobiomorph centipede *Lithobius forficatus* (e.g., trnaF-nad5-H-nad4-nad4L on the minus strand), other sections are completely rearranged to an extent unusual in arthropods, and metazoans (ACR and MJT, unpublished). This confounds attempts to use *S. maritima* mitochondrial gene order in phylogenetic reconstructions.

### Conserved Synteny with Other Phyla

With the exception of some conserved local gene clusters, the location of genes on the chromosomes of *Drosophila* and other Diptera retains no obvious trace of the ancestral bilaterian gene linkage. Other holometabolous insects such as *Bombyx mori* and *Tribolium castaneum* do show significant conservation of large-scale gene linkage with other phyla, for example, in the chordate *Branchiostoma floridae* (amphioxus) and the cnidarian *Nematostella vectensis* [44],[45]. The last common ancestor of these two lineages pre-dated the ancestor of all bilaterian animals, and yet the genomes of these species retain detectable conserved synteny: orthologous genes are found together on the same chromosomes, or chromosome fragments, far more often than would be expected by chance.

**Figure 3:**
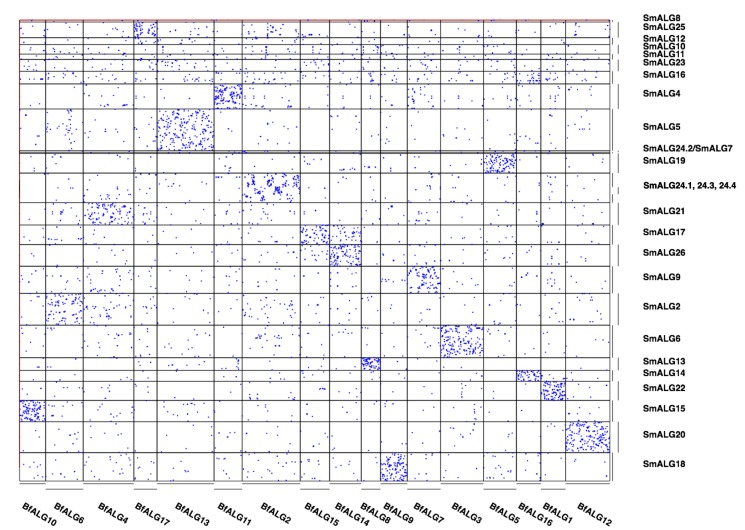
Conserved macro synteny signal between *S. maritima* and the chordate lancelet *B. floridae* clustered into ancestral linkage groups. Each dot represents a pair of genes, one in *B. floridae*, one in *S. maritima*, assigned to the same gene family by our orthology analysis. The ancestral linkage group identifiers refer to groups of scaffolds from the *S. maritima* (SmALG) or *B. floridae* (BfALG) assemblies, as detailed in File S2. The identification of ALGs is described in the SI. Note that two *S. maritima* scaffolds were divided across ALGs, and so appear multiple times in File S2.

We find the *S. maritima* genome also retains significant traces of the large-scale genome organisation that was present in the bilaterian ancestor. Although the assignment of scaffolds to chromosomes is not determined in *S. maritima*, there are sufficient gene linkage data within scaffolds to reveal clear retained synteny between amphioxus and *S. maritima* (Figure 3), at a higher level than any of the Insecta or Pancrustacea we have examined.

Of the 62 scaffolds with at least 20 genes from ancestral bilaterian orthology groups, 37 show enrichment of shared orthologues with one or (in the case of a single scaffold) two chordate ancestral linkage groups (ALGs) at a significance threshold of *p*<0.0001 (after Bonferroni correction for 1,116 pairwise ALG-scaffold comparisons). Of these scaffolds' genes that have predicted human orthologues, 57% are found in a conserved macro-synteny context. At a more relaxed significance threshold (*p*<0.01), 71% of these scaffolds have a significant association with at least one chordate ALG, and 17 of the 18 chordate ALGs hit at least one of these scaffolds.

Stronger synteny is also detected for the genome of the nematode *Caenorhabditis elegans* with *S. maritima* than with insects or other Metazoa. The *C. elegans* genome is highly rearranged, and shows low synteny with higher insects, or with chordates [7],[46],[47]. As members of the Ecdysozoa, nematodes last shared a common ancestor with the arthropods more recently than with chordates. This shared ancestry allows traces of conserved genome organisation to be detected with slowly rearranging arthropod genomes, even when it is only weakly apparent with chordates.

By implication, the last common ancestor of the arthropods retained significant synteny with the last common ancestor of bilaterians as well as the last common ancestors of other phyla, such as the Chordata. This conserved synteny is more complete with this *S. maritima* genome sequence, due to the relative scrambling of the genomes of those other arthropods that have been sequenced previously.

### Homeobox Gene Clusters: Hox, ParaHox, SuperHox, and Mega-homeobox

The clustering of genes in a genome is often of functional significance (e.g., reflecting co-regulation), as well as providing important insights into the origins of particular gene families when clusters are composed of genes from the same class or family. Gene clusters can also be a useful proxy for the degree of genome rearrangement. The homeobox gene super-class is one type of gene for which clustering has been extensively explored. *S. maritima* has 113 homeobox-containing genes, which is slightly more than seen in other sequenced arthropods such as *D. melanogaster*, *T. castaneum*, and *Apis mellifera*. This is due to some lineage-specific duplications in *S. maritima* as well as the retention of some homeobox families that have been lost in other arthropods, including Vax, Dmbx, and Hmbox (see Text S1).

The homeobox-containing genes of the Hox gene cluster are renowned for their role in patterning the anterior-posterior axis of animal embryos. *S. maritima* has an intact, well-ordered Hox cluster containing one orthologue of each of the ten expected arthropod Hox genes, except for Hox3. There are two potential Hox3 genes elsewhere in the *S. maritima* genome [48], but the true orthology of these genes remains slightly ambiguous; it remains possible that they are the first example of ecdysozoan Xlox ParaHox genes (see Text S1). The Hox cluster spans 457 kb (*labial* to *eve*), a span similar to assembled Hox clusters in a range of other invertebrate groups (crustacean, mollusc, echinoderm, cephalochordate). This suggests that the contrasting very large (and frequently broken) Hox clusters of Drosophilids and some other insects are a derived characteristic. However, the spectrum of alternatively spliced and polyadenylated transcripts encoded by the *Hox* genes of *S. maritima* is comparable with what is known from *D. melanogaster* (details in Text S1). Exceptionally among protostomes, the *S. maritima* Hox cluster retains tight linkage to one orthologue of *evx/evenskipped*, as it does in some chordates and cnidarians.

**Figure 4:**
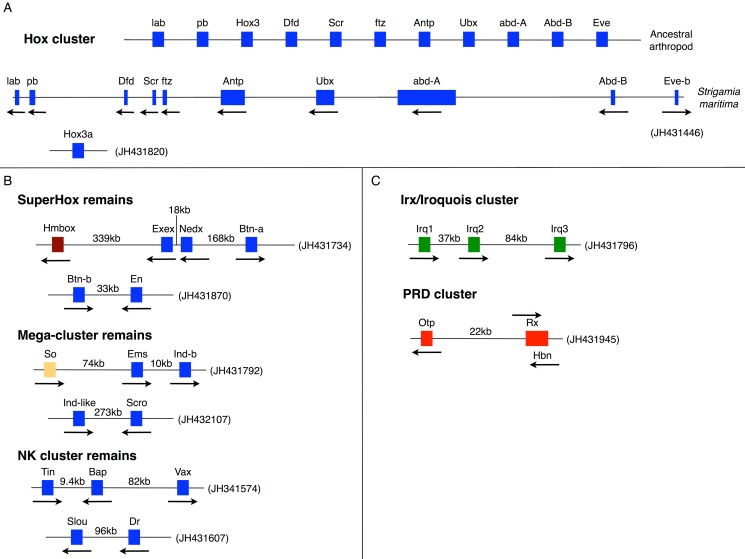
Homeobox gene clusters. (A) The Hox gene cluster of *S. maritima* compared to the cluster that can be deduced for the ancestral arthropod. *S. maritima* provides the first instance of an arthropod Hox cluster with tight linkage of an *Even-skipped (Eve)* gene (see text). Hox3 is the only gene missing from the *S. maritima* Hox cluster, but may be present elsewhere in the genome on a separate scaffold (see main text and Text S1 for details). The *S. maritima* cluster is drawn approximately to scale and spans 457 kb from the start codon of *labial (lab)* to the start codon of *Eve-b*. Arrows denote the transcriptional orientation. (B) Remains of clustering and linkage of ANTP class genes in *S. maritima*. The blue boxes are genes belonging to the ANTP class. The brown box is a gene belonging to the HNF class. The orange box is a gene belonging to the SINE class. The intergenic distances are indicated in kb. (C) Clusters of non-ANTP class homeobox genes in *S. maritima*. The green boxes are genes belonging to the TALE class. The red boxes are genes belonging to the PRD class. The intergenic distances are indicated in kb, except in the case of Rx-Hbn as these genes are overlapping but with opposite transcriptional orientations. All scaffold numbers are indicated in brackets.

Further instances of homeobox gene clustering and linkage, and reconstructions of ancestral states, are summarized in Figure 4 and Table 1 (and see Text S1). The Hox gene cluster is hypothesized to have evolved within the context of a Mega-homeobox cluster that existed before the origin of the bilaterians and consisted of an array of many ANTP-class genes [49]–[51]. By the time of the last common ancestor of bilaterians the Hox cluster existed within the context of a SuperHox cluster, containing the Hox genes themselves and at least eight further ANTP-class genes [52]. The conservative nature of the *S. maritima* genome has left several fragments from the Mega-homeobox and SuperHox clusters still intact (Figure 4; Table 1). Furthermore, homeobox linkages in *S. maritima* raise the possibility that further genes could have been members of the Mega-homeobox and SuperHox clusters, including the ANTP-class gene *Vax*, as well as the SINE-class gene *sine oculis* and the HNF-class gene *Hmbox* (see Text S1 for further details).

**Table 1:** Instances of homeobox gene clustering and linkage.

Gene Cluster	Details	Conclusion or Hypothesis
Hox Cluster	Intact well ordered, but lacking *Hox3* (Figure 4A). Two potential *Hox3* genes elsewhere in the genome, but these could also be *Xlox* homologues	Has *Xlox* really been lost from all lineages of the ecdysozoan super phylum?
*NK* - *Vax* linkage	Centipede has gene pair remnants from the ancestral NK cluster *slouch* and *drop*, and *tinman* and *bagpipe* (now with *Vax* linkage, which also seen in mollusc) (Figure 4B)	*Vax* linkage likely ancestral, *Vax* a new member of the ancestral ANTP class mega-homeobox cluster.
IRX/Iroquois	Cluster of three *Irx* genes(Figure 4C)	Independent expansion from *Drosophila* by duplication of *mirror*.
*Orthopedia*, *Rax*, and *Homeobrain*	Cluster present in *S. maritima* (Figure 4C)	An ancestral cluster also found in insects, cnidarians, and molluscs.
SuperHox cluster remains	Linkage of *BtnN* and *En* on Scaffold JH431870. Linkage of *Exex*-*Nedx*-*BtnA* on scaffold JH431734 (Figure 4B) with *Hmbox*.	Remnants of the Super-Hox cluster?
ParaHox - *NK* linkage (Mega-cluster remains)	Tight linkage of *Ems* (NK gene) with *IndB* (ParaHox gene), and *Ind-like* (ParaHox like) with *scro* (NK gene) (Figure 4B)	Possible remnant of ParaHox and NK clusters from ancestral Mega-Cluster^a^
SINE-ANTP class linkage	linkage of *sine oculis* & *Ems*	Also seen in humans and zebrafish - thus linkage of SINE and ANTP genes in bilaterian ancestor

Further details are provided in Text S1.

a Note these have become secondarily linked in vertebrates [50].

### Chemosensory Gene Families (Gustatory Receptors, Ionotropic Receptors, Odorant Binding Proteins, Chemosensory Proteins)

The chemosensory system of arthropods is best known in insects. During the evolutionary transition from water to terrestrial environments, insects evolved a new set of genes to detect airborne molecules (odorants) [53]–[55]. The independent colonization of land by insects and myriapods raises two interesting questions: (1) what are the genes involved in chemosensation in non-insect arthropods, and (2) what genes are responsible for the detection of airborne molecules in other terrestrial arthropods? We searched the *S. maritima* genome for homologues of the insect chemosensory genes, included in six gene families, three ligand binding protein families: odorant binding proteins (OBPs) [56],[57], chemosensory proteins (CSPs) [58],[59], and CheA/B [60],[61]; and three membrane receptor families: GRs [62],[63], odorant receptors (ORs) [64],[65], and IRs [66],[67].

**Figure 5:**
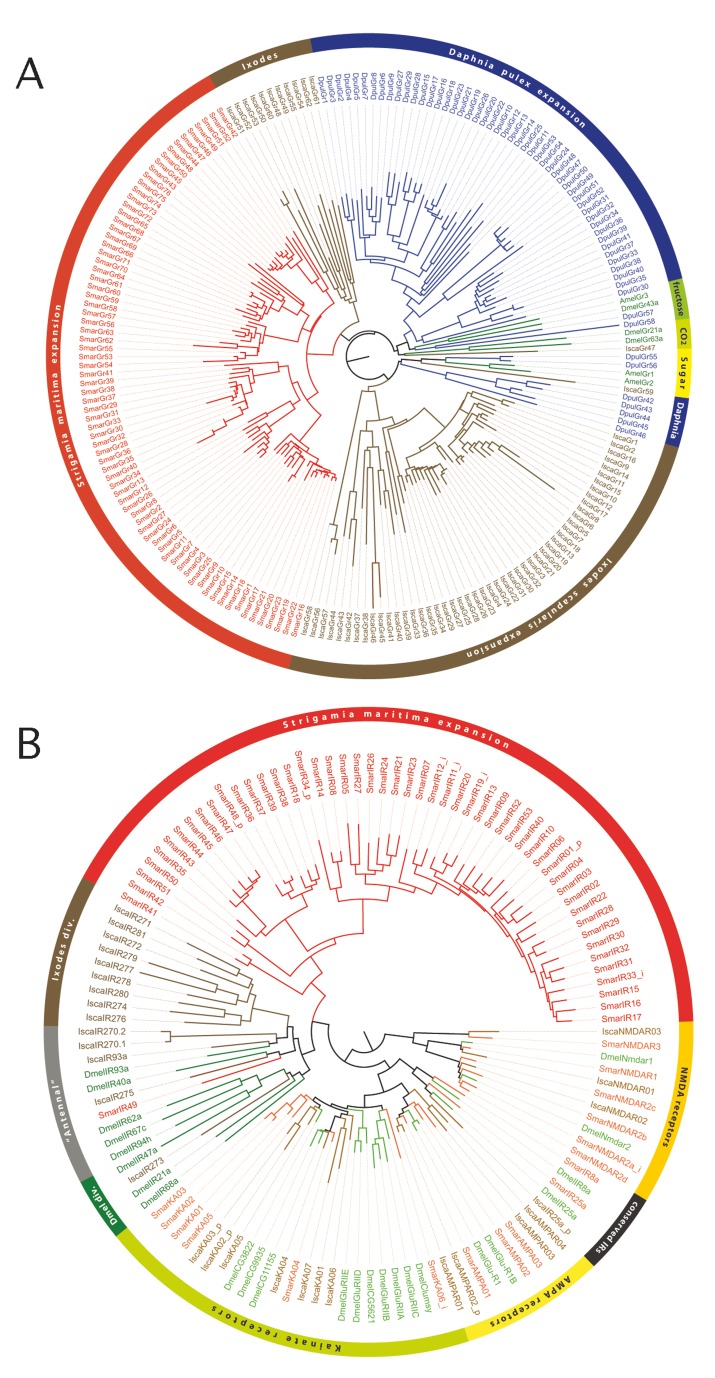
Expansion of chemosensory receptor families. (A) Phylogenetic relationships among *S. maritima* (Smar), *I. scapularis* (Isca), *D. pulex* (Dpul), and a few insect GRs that encode for sugar, fructose, and carbon dioxide receptors (Dmel, *D. melanogaster*, and Amel, *A. mellifera*). (B) Phylogenetic relationships among *S. maritima*, *I. scapularis*, and a few *D. melanogaster* IRs and IgluR genes (the suffix at the end of the protein names indicates: i, incomplete and p, pseudogene).

Of the ligand binding proteins, we found only two genes belonging to the CSP family, but no representatives of the OBP or CheA/B families. Among the membrane receptor families, we identified a number of genes of both the GR and IR families, but no OR genes. The GR family in *S. maritima* is represented by 77 genes, 17 of which seem to be pseudogenes, with similar numbers of genes and pseudogenes being fairly typical features of this gene family in other arthropods. A phylogenetic tree revealed that none of the *S. maritima* GR genes have 1∶1 orthology to other arthropod GRs. Instead, all *S. maritima* GRs cluster in a single clade, with six major subclades, representing separate expansions of the GR repertoire in the centipede lineage (Figure 5A and see Text S1). The IR family is known to be ancient [67], but *S. maritima* has a relative expansion of this family. The search for IRs led to the annotation of 69 genes, 15 of which belong to the IGluR subfamily, which is not involved in chemosensation, but is highly conserved among arthropods and animals in general. Among the remaining 54 IRs, three are orthologues of conserved IR genes that have been shown to have an olfactory function in *D. melanogaster*. However, 51 of the *S. maritima* IRs do not have orthologues either in *D. melanogaster* or in *Ixodes scapularis*, clustering together in a single clade (the expansion clade in Figure 5B). This finding suggests that most *S. maritima* IRs, as observed with GRs, have duplicated from a common ancestral gene exclusive to the centipede lineage.

The absence of the insect OR family agrees with the prediction of Robertson and colleagues [54] that this lineage of the insect chemoreceptor superfamily evolved with terrestriality in insects, and it is also missing from the water flea *Daphnia pulex* [53]. The same appears to be true for the OBPs. We therefore infer that, as centipedes adapted to terrestriality independently from the hexapods, they utilized a novel combination of expanded GR and IR protein families for olfaction, in addition to their more ancestral roles in gustation.

### Light Receptors and Circadian Clock Genes

*S. maritima*, like all species of the order Geophilomorpha, is blind [37]. Nevertheless, it avoids open spaces and negative phototaxis has been demonstrated in other species of Geophilomorpha [38],[68]. We searched the *S. maritima* genome for light receptor genes. Interestingly, we have found no opsin genes, no homologue of gustatory receptor 28b (GR28b), which is involved in larval light avoidance behaviour in *Drosophila* [69], and no cryptochromes. Thus, none of the known arthropod light receptors are present. Furthermore, there are no photolyases, which would repair UV light induced DNA damage. As a consequence, the critical avoidance of open spaces by *S. maritima* must either be mediated by other sensory instances than light perception, or *S. maritima* possesses yet unknown light receptor molecules.

The absence of light receptors, particularly cryptochromes, also raises the issue of the entrainment and composition of a potential *S. maritima* circadian clock. Strikingly, we could not identify any components of the major regulatory feedback loop of the canonical arthropod circadian clock (including *period*, *cycle*, *b-mal/clock*, *timeless*, *cryptochromes 1 and 2*, *jetlag* [70]). The only circadian clock genes found (*timeout*, *vrille*, *pdp1*, *clockwork orange*) are generally known to be involved in other physiological processes as well [71]–[73]. The extensive secondary gene loss of both light receptors and circadian clock genes raises questions about the actual existence of a circadian clock in *S. maritima*. One could hypothesize that a circadian clock may not be required in *S. maritima's* subsurface habitat, although other periodicities, such as tide cycles, might be important. If *S. maritima* does have a circadian clock then it must be operating via a mechanism distinct from the canonical arthropod system.

Other blind or subterranean animals do maintain a circadian rhythm, despite complete loss of vision and connection with the surface (e.g., Spalax) [74]–[76]. In other cases (e.g., blind cave crayfish [77]), despite the loss of vision, opsin proteins remain functional, and are hypothesized to have a role in circadian cycles. However, both these examples represent species that have become blind and subterranean relatively recently. To confirm that the loss of these genes is not general for all centipedes, we performed BLASTP analyses searching for the set of light sensing and circadian clock genes that are missing from *S. maritima* in RNAseq data from the house centipede *Scutigera coleoptrata* (NCBI SRA accession SRR1158078), a species with well-developed eyes. We find homologs to period, cycle, b-mal/clock, jetlag, cryptochrome1, cryptochrome 2, (6-4)-photolyase, and nina-e (rhodopsin 1), suggesting that both light sensing and circadian clock systems were present in ancestor of myriapods. Although we have no direct information about photoreceptors or circadian genes in other geophilomorph species, the fact that all geophilomorphs are blind suggests that the loss of the related genes is very ancient, and may date back to the origin of the clade.

### Putative Cuticular Proteins

A defining characteristic of arthropods is an exoskeleton with chitin and cuticular proteins as the primary components. Although several families of cuticular proteins have been recognized, the CPR family (Cuticular Proteins with the Rebers and Riddiford consensus) is by far the largest in every arthropod for which a complete genome is available, with 32 to >150 members [78]. Proteins in the CPR family have a consensus region in arthropods of about 28 amino acids, first recognized by Rebers and Riddiford [79], which was subsequently extended to ∼64 amino acid residues and shown to be necessary and sufficient for binding to chitin [80]. No clear instances of the Rebers and Riddiford (RR) consensus have been identified outside the arthropods. We identified 38 members of the CPR family in *S. maritima*. There are two main forms of the consensus, designated RR-1 and RR-2, with the former primarily associated with flexible cuticle, the latter with rigid cuticle. Interestingly, while chelicerates studied to date have no members of the RR-1 subfamily (as classified at CutProtFam-Pred, http://aias.biol.uoa.gr/CutProtFam-Pred/home.php), seven of the *S. maritima* CPR proteins clearly belong to this class. This would be consistent with the origin of the RR1-coding genes being in the mandibulate ancestor after this lineage had diverged from the chelicerate lineage. Further data are needed to verify that the identified proteins are indeed important constituents of the cuticle.

### Neuro-endocrine Hormone Signalling

Cell-to-cell communication in arthropods occurs via a variety of neurotransmitters and neuro-endocrine hormones, including biogenic amines, neuropeptides, protein hormones, juvenile hormone (JH), and ecdysone. These signalling molecules and their receptors steer central processes such as growth, metamorphosis, feeding, reproduction, and behaviour. Most receptors for biogenic amines, neuropeptides, and protein hormones are G protein-coupled receptors (GPCRs) [81]. Intracellularly, the G proteins initiate second messenger cascades [82]. JH and ecdysone, however, are lipophilic and can diffuse through the cell membrane to bind with nuclear receptors [83],[84]. In addition, ecdysone can also activate a specific GPCR, and initiate a second messenger cascade [85]. There is extensive cross-talk between these extracellular signal molecules.

*S. maritima* possesses 19 biogenic amine receptors, a number similar to the 18–22 biogenic amine receptors that have been identified in other arthropods (Table S19). In *S. maritima*, there are four octopamine GPCRs, one octopamine/tyramine, one tyramine, four dopamine and three serotonin GPCRs, three GPCRs for acetylcholine, one GPCR for adenosine, and two orphan biogenic amine receptors. Although this distribution resembles very much that of *Drosophila* and other arthropods, there are some interesting differences with *Drosophila*, which expresses two additional β-adrenergic-like octopamine receptors compared to *S. maritima*, while *S. maritima* expresses two putative β-adrenergic-like octopamine receptors (Sm-OctBetaRHK and Sm-D1/OctBeta), which are expressed in a number of insect and tick species, but not in *Drosophila* (Table S20) [86]. The true functional identities of all the putative *S. maritima* biogenic amine GPCRs awaits their cloning, functional expression, and pharmacological characterization in cell lines.

In addition, 36 neuropeptide and protein hormone precursor genes are present in this centipede. Each neuropeptide precursor contains one or more (up to seven) immature neuropeptide sequences (Figure S20). Interestingly, the centipede contains two CCHamide-1, two eclosion hormone, and two FMRFamide genes, whereas these genes are only present as single copies in the genomes of most other arthropods [87]. In concert with the presence of 36 neuropeptide genes, we found 33 genes for neuropeptide receptors (31 GPCRs and two guanylcyclase receptors) (see Table S21). As observed for the neuropeptide genes, a number of the neuropeptide receptor genes, which are only found as single copies in most other arthropods, have also been duplicated. *S. maritima* has two inotocin GPCR genes, two SIFamide, two corazonin, two eclosion hormone guanylcyclase receptor genes, two eclosion triggering hormone GPCR genes, three sulfakinin GPCR genes, and three LGR-4 (Leu-rich-repeats-containing-GPCR-4) genes. The latter receptors are orphans (GPCRs without an identified ligand) and only present as single-copy genes in most other arthropods [88]. Several of these duplicated GPCR genes are located in close vicinity to each other in the genome (Figure S21, suggesting recent duplication events. Furthermore, duplications of both the eclosion hormone and its receptor genes and the duplication of the ecdysis triggering hormone receptor genes suggest that the process of ecdysis (moulting) has undergone some sort of modification, perhaps requiring more complex control in the lineage leading to centipedes.

We summarize in Table S22 the neuropeptide/protein hormone signalling systems that are present or absent in selected arthropod genome sequences. Each arthropod species, including *S. maritima*, has its own characteristic pattern, or “barcode,” of present/absent neuropeptide signalling systems. However, the relationship between the specific neuropeptide “barcode” and physiology remains to be elucidated.

Insect JH is important for growth, moulting, and reproduction in arthropods [84]. This hormone is a terpenoid (unsaturated hydrocarbon) that is synthesized from acetyl-CoA by several enzymatic steps (Figure S22). In several insects the production of JH is stimulated by the neuropeptide allatotropin, while it is inhibited by either allotostatin-A, -B, or -C [89],[90]. We found that *S. maritima* has orthologues of many of the biosynthetic enzymes needed for JH biosynthesis in insects (Table S23). Also, the JH binding proteins are encoded in the centipede genome as well as JH degradation enzymes (Table S24). This implies that the complete JH system is present in this centipede. Similarly, neuropeptides that could stimulate or inhibit the synthesis and release of JH, such as allatotropin and the allatostatins -A, -B, and -C, are also present in *S. maritima* (Figure S22, suggesting that the overall functioning of the JH system in centipedes might be very similar to that of insects) (Table S23). To date, the existence of JH signalling systems has been demonstrated in insects, crustaceans, and recently in spider mites [89],[91],[92]. Its occurrence in *S. maritima* and spider mites (Chelicerata) indicates that JH signalling has deep evolutionary roots and we suggest that it might have evolved together with the emergence of the exoskeleton in arthropods.

### Developmental Signalling Systems

Certain signalling systems, including transforming growth factor (TGF)-beta, Wnt, and fibroblast growth factor (FGF), are used throughout development across the animal kingdom. Various lineage-specific modifications of these systems have occurred, particularly within the arthropods. With regards to TGF-beta signalling we found single orthologues of all members of the Activin family, except Alp (Activin-like protein) (see Figure S23; Text S1). In the BMP-family, the *S. maritima* genome contains two divergent BMP sequences, as well as a clear orthologue of *glass-bottom boat* (*gbb*) and two *decapentaplegic* (*dpp*) orthologues. In addition, the *S. maritima* sequences confirm the ancestral presence of an anti-dorsalizing morphogenetic protein (ADMP) and a BMP9/10 orthologue in arthropods, which are both absent from *Drosophila* [93]. Most interestingly, the *S. maritima* genome includes the antagonistic BMP ligand BMP3 (previously suggested to be present only in deuterostomes [94]), a potential *gremlin*/*neuroblastoma suppressor of tumorigenicity*, and two nearly identical *bambi* genes (absent from *Drosophila*), and the BMP inhibitor *noggin* (present in vertebrates but lost in most holometabolous insects). The multiple BMP-agonists and -antagonists indicate that considerable changes have occurred in the TGF-beta signalling system during arthropod evolution, particularly in the Holometabola.

Reconstructions of Wnt gene evolutionary history suggest that the ancestral bilaterian possessed at least 13 distinct Wnt gene subfamilies [95],[96]. This initial number has been secondarily reduced in many taxa. This trend of secondary gene loss is readily apparent within the arthropods, with holometabolous insects such as *D. melanogaster* retaining only seven Wnt subfamilies [97],[98]. In contrast, the Wnt signalling complement in *S. maritima* comprises 11 of the 13 Wnt-ligand subfamilies (Figure S24). Phylogenetic investigation has identified these genes as *wnt1*, *wnt2*, *wnt4*, *wnt5*, *wnt6*, *wnt7*, *wnt9*, *wnt10*, *wnt11*, *wnt16*, and *wntA*. *wnt3* and *wnt8* are missing from the *S. maritima* genome. While the absence of *wnt3* is common to protostomes, *wnt8* or *wnt8*-like sequences occur in other protostome genomes, including insects, spiders, and another myriapod, *Glomeris marginata* [97]. The Wnt genes are known to display a degree of linkage and clustering in many arthropods. Some conservation of this is also found in *S. maritima*, with *wnt1*, *wnt6*, and *wnt10* adjacent to each other on the same scaffold, possibly representing part of an ancient clustering (Table S25) [99].

The primary receptors for Wnt ligands in the canonical Wnt signalling pathway are the trans-membrane receptors of the Frizzled family. Five of these have been identified: *Frizzled1*, *Frizzled4*, *Frizzled5/8*, *Frizzled7*, and *Frizzled10*. As is the case for the *wnt* genes themselves, this is a larger number than is found in most arthropods. Other *Fz*-related genes are also present: *smoothened*, involved in Hedgehog signalling, and *secreted frizzled related protein*, which has inhibitory roles in Wnt signalling in other taxa. Putative non-canonical Wnt receptors are also encoded, including two subfamilies of *receptor tyrosine kinase-like orphan receptor* (*ror*). In addition to *ror2*, there is a lineage-specific duplication of *ror1*, making a total of three *ror* genes, as opposed to only one in *D. melanogaster*. Another Wnt agonist, the *R-spondin* orthologue was also found. As part of the Wnt-binding complex we found one *arrow*-LRP5/6-like Wnt-coreceptor gene in the genome: *lrp6*. Other LRP-molecules with potential Wnt-binding activity also exist: LRP1, LRP2, and LRP4. Because of the absence of an intracellular signalling domain these could potentially function as Wnt-inhibitors. Together, the large number of ligand and receptor genes point towards both the conservation of an ancestral Wnt signalling system and to a certain degree of unusual complexity in of this system in *S. maritima*.

Concerning the FGF pathway, we identified two closely related FGF receptors. These two *S. maritima* receptors are likely to stem from a duplication in the myriapod lineage that was independent from that which generated the two *Drosophila* FGFRs, *Heartless* and *Breathless* (Figure S25). The number of FGF ligands found in the genomes of insects such as *D. melanogaster* (three *fgf* genes) or *T. castaneum* (four *fgf* genes) is small when compared to 22 *fgf* genes found in the genomes of vertebrates. In the *S. maritima* genome, we identified three *fgf*-genes (Figure S26). One of them potentially represents an *fgf 18/8/24* orthologue to which the *fgf8*-like genes of *Tribolium* and of *Drosophila* (*pyramus* and *thisbe*) are associated. The second *S. maritima fgf* groups with the *fgf1* genes, while the third groups with the *fgf 16/9/20* clade (the first known arthropod member of this clade). Low support values for this grouping raise the possibility that it might actually be an orthologue of insect *branchless* genes. Other FGF-pathway genes present in *S. maritima* include *stumps* (Downstream-of-FGF-signalling [DOF]) and *sprouty related*.

### Protein Kinases

**Figure 6:**
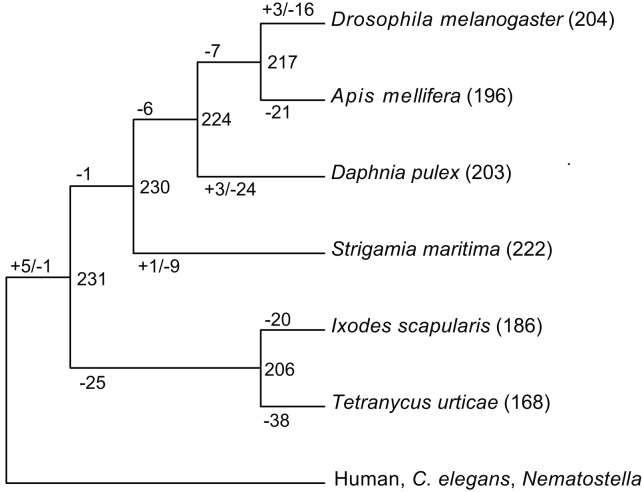
Ancestral protein kinases are extensively lost during arthropod evolution. *S. maritima* is an exception and retains the largest number of ancestral kinases. Numbers of kinase subfamilies in selected species are shown in parentheses after species names. The gains, losses, and inferred content of common ancestors are listed on internal branches. Kinases found in at least two species from human, *C. elegans* and *Nematostella vectenesis* were used as an outgroup.

Kinases make up about 2% of all proteins in most eukaryotes, while they phosphorylate over 30% of all proteins and regulate virtually all biological functions. We identified 393 protein kinases in the *S. maritima* genome, representing 2.6% of the proteome. We classified these into conserved families and subfamilies, compared the kinome to those of 26 other arthropods and inferred the evolutionary history of all kinases across the arthropods (Figure 6). We predict that an early arthropod had at least 231 distinct kinases and see considerable loss of ancestral kinases in most extant species. *S. maritima* has the smallest number of losses among the arthropods, with only ten kinases lost relative to the arthropod ancestor. In contrast, the two chelicerates *T. urticae* and *I. scapularis* have lost 63 and 45 kinases, respectively, and *D. melanogaster* lost 30, giving *S. maritima* the richest repertoire of conserved kinases of any arthropod examined. All but one of the losses in *S. maritima* have been lost in other arthropods, suggesting that these genes may be partially redundant or particularly prone to loss. The one unique loss is NinaC, which in *Drosophila* is required for vision, likely associated with other vision related gene loss described above. As in many other species, we also see some novelties and expansions of existing families: the SRPK kinase family, involved in splicing and RNA regulation, has expanded to 36 members, and the nuclear VRK family is expanded to 16. A novel family of receptor guanylate cyclases (nine genes) and three clusters of unique protein-kinase-like (PKL) kinases, containing 28 genes in total, are also seen, though their functions are not known.

### Developmental Transcription Factors

DNA binding proteins with the capacity to regulate the expression of other genes are central players in the control of development and many other processes. Since one of the original interests in *S. maritima* was for its developmental characteristics, we carried out a survey of developmentally relevant transcription factors, with an emphasis on transcription factors suspected to be involved in processes of axial specification, segmentation, mesoderm formation, and brain development. We identified orthologues of ∼80 transcription factors of the Zinc finger and helix-loop-helix families in addition to the 113 homeobox-containing transcription factors already discussed (see Text S1). In no case did we fail to find at least one orthologue of the gene families expected from our knowledge of *Drosophila*, though individual duplications and losses among gene families were not uncommon. Among the set of pair-rule segmentation genes, for example, *S. maritima* has multiple homologues of *paired*, *even-skipped*, *odd-skipped*, *odd-paired*, and *hairy*-like genes, but only a single orthologue of *sloppy-paired* and *runt*-like genes, whereas *Drosophila* has multiple *runt* and *sloppy-paired* genes but only single orthologues of *even-skipped* and *odd-paired*. Where both lineages have multiple copies, (*paired*, *hairy*, *odd-skipped*), sequence alone rarely defines one-to-one orthologous relationships, and the evolutionary history remains unclear [29]. Other notable duplications include *caudal* (three genes) and *brachyury* (two genes). In a number of cases, transcription factors known to play a role in vertebrate development, but apparently missing from *Drosophila* and other insects, are retained in *S. maritima*. Examples include the homeobox genes *Dmbx* and *Vax* noted above, and the FoxJ1, FoxJ2, and FoxL1 subfamilies of *forkhead/Fox* factors.

One of the developmental transcription factors provides an example where insects use isoforms to generate alternative proteins that are encoded by paralogous genes in *S. maritima*. Two centipede orthologues of the developmental transcription factor *cap‘n’collar* encode isoforms that differ at their N-terminal end. The longer protein, encoded by the gene *cnc1*, contains sequence motifs that align to *Drosophila cnc* isoform C (Figure S27, which is broadly expressed throughout embryonic development) [100]. *S. maritima cnc1* is similarly expressed ubiquitously, whereas the other orthologue, *cnc2*, shows a segment specific pattern of expression similar to that of the shorter *Drosophila cnc* isoform B (VSH and MA, unpublished) [100].

### Immune System

Arthropods can mount an innate immune response against pathogenic bacteria, fungi, viruses, and metazoan parasites. The nature of the responses to these invaders, such as phagocytosis, encapsulation, melanisation, or the synthesis of antimicrobial peptides, is often similar across arthropods [101]. Furthermore, key aspects of innate immunity are conserved between insects and mammals, which suggests an ancient origin of these defences. Previous studies have revealed extensive conservation of key pathways and gene families across the insects and crustaceans [102]. Beyond the Pancrustacea the extent of immunity gene conservation is unclear. Therefore, we searched the *S. maritima* genome for homologues of immunity genes characterised in other arthropods.

**Figure 7:**
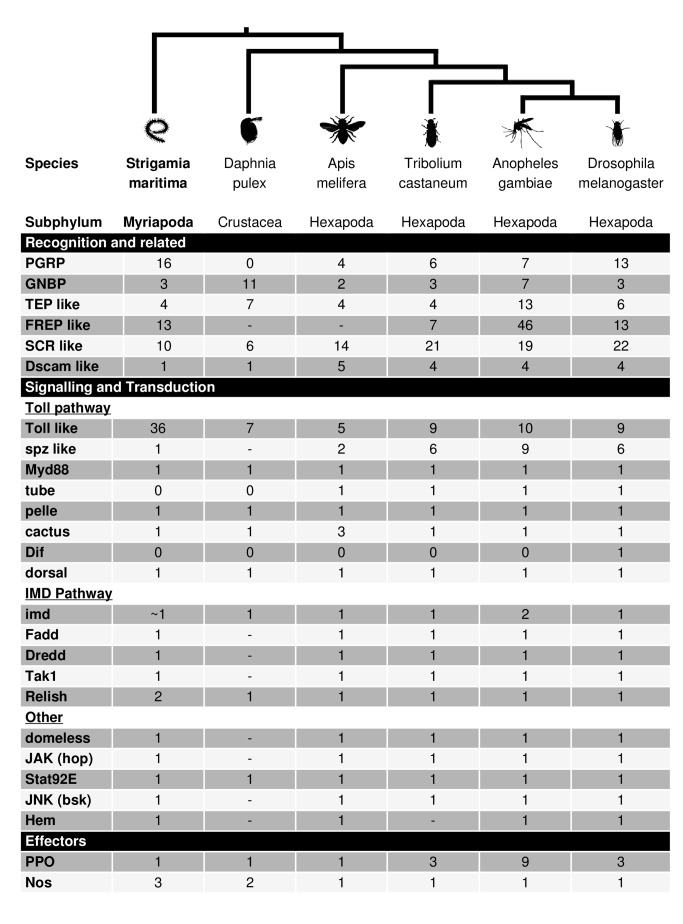
Presence and absence of immunity genes in different arthropods. Counts of immune genes are shown for *S. maritima*, *D. pulex* [131], *A. mellifera* [86], *T. castaneum*, *Anopheles gambiae*, and *D. melanogaster* [132]. ∼, identity of the gene is uncertain; -, not investigated.

We found conservation of most immunity gene families between insects and *S. maritima* (Table S30), suggesting that the immune gene complement known from *Drosophila* was largely present in the most recent common ancestor of the myriapods and pancrustaceans. The humoral immune response of insects recognises infection using proteins that bind to conserved molecular patterns on pathogens [103]. Sequence homologues for the major recognition protein families found in *Drosophila*, peptidoglycan recognition proteins (PGRPs), and gram-negative bacteria-binding proteins (GNBPs), were found with the expected protein domains. These proteins then activate signalling pathways [103], and all four major insect immune signalling pathways (Toll, IMD, JAK/STAT, and JNK) are present in *S. maritima*, with 1∶1 sequence homologues of most pathway members. The cellular immune response of insects relies on receptors and opsonins including thioester-containing proteins (TEPs), fibrinogen related proteins (FREPs), and scavenger receptors [103],[104], and these are also present in *S. maritima*, often with protein domains in the same arrangement as *Drosophila*. We also find sequence homologues for effector gene classes including nitric oxide synthases (NOS) and prophenoloxidase (PPO). However, we failed to identify any antimicrobial peptide homologues, possibly as these genes are often short and highly divergent between species. In insects, it is common to find that certain immune gene families have undergone expansions in certain lineages [105]. Again, this is mirrored in *S. maritima*, where we found lineage-specific expansions of the PGRP and Toll-like receptor genes (TLRs) (Figure 7). Overall, the presence of the main families of immunity genes suggests that there is also functional conservation of the immune response.

The innate immune system is thought to rely on a small number of immune receptors that bind to conserved molecules associated with pathogens. This view was challenged by the discovery in *Drosophila* that the gene *Dscam* (Down syndrome cell adhesion molecule), which has the potential to generate over 150,000 different protein isoforms by alternative splicing, functions as an immune receptor in addition to its roles in nervous system development [106]. Dscam family members are membrane receptors composed of several immunoglobulin (Ig) and fibronectin domains (FNIII). In pancrustaceans one member of the Dscam family has a large number of internal exon duplications and a sophisticated mechanism of mutually exclusive alternative splicing, which enables a single *Dscam* locus to somatically generate thousands of isoforms, which differ in half of two Ig domains (Ig2 and Ig3) and in another complete Ig domain (Ig7). This creates a high diversity of adhesion properties, useful for immune responses.

**Figure 8:**
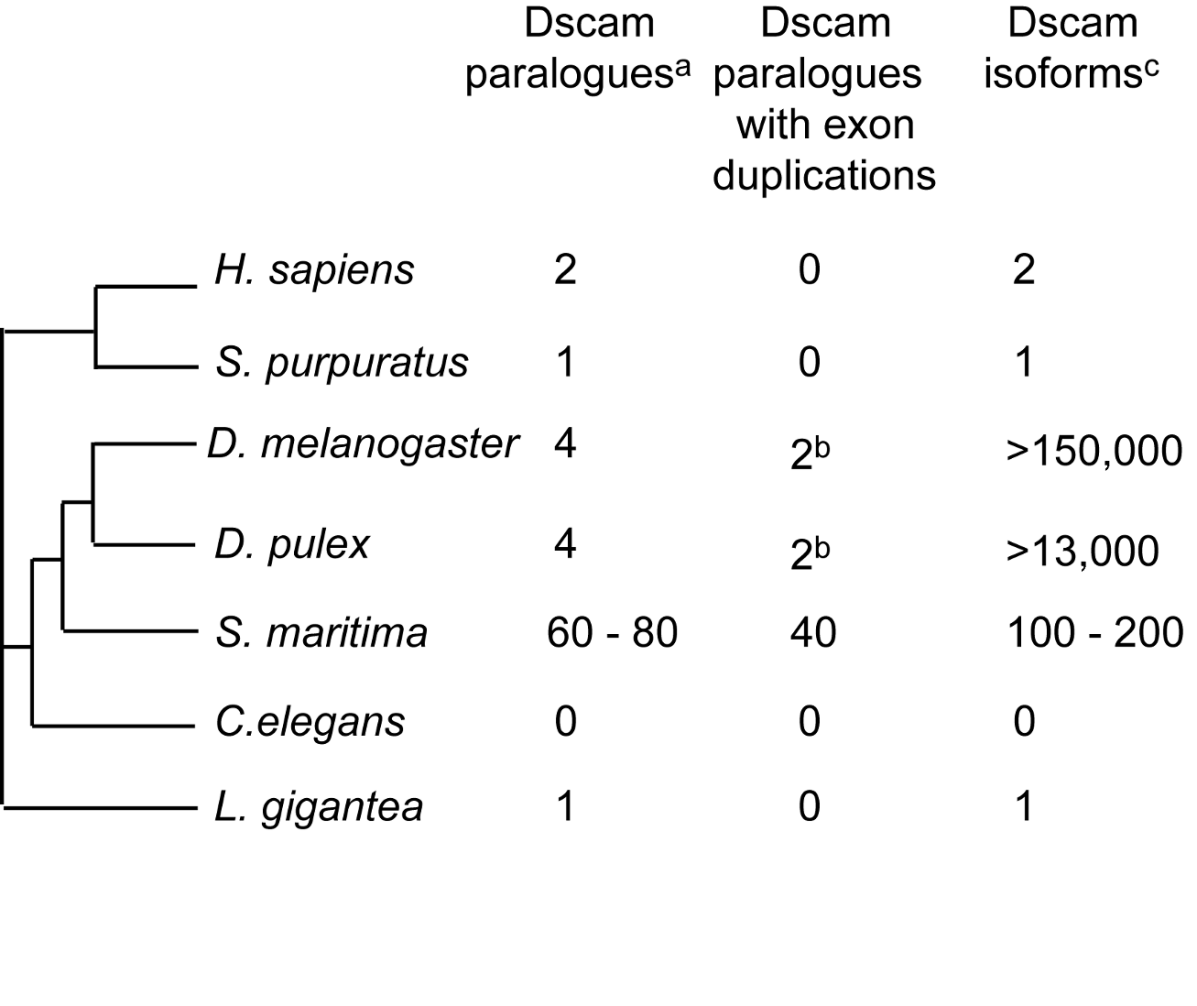
Dscam diversity caused either by gene and/or exon duplication in different Metazoa. ^a^Only canonical Dscam paralogues were considered. ^b^In *D. melanogaster* and *D. pulex* the paralogue Dscam-L2 has two Ig7 alternative coding exons. ^c^Potential number of Dscam isoforms, circulating in one individual, produced by mutually exclusive alternative splicing of duplicated exons.

We found that *S. maritima* has evolved a different strategy to generate a diversity of Dscam isoforms [107]. The genome contains 60 to 80 canonical *Dscam* paralogues and over 20 other *Dscam* related incomplete or non-canonical genes (Figure 8). In 40 *Dscam* genes, the exon coding for Ig7 is duplicated two to five times (but not the exons coding for Ig2 and Ig3, which are duplicated in pancrustaceans). Our analysis of transcripts suggests that many of those duplicated exons might be alternatively spliced in a mutually exclusive fashion, supporting the hypothesis that the mechanism of mutually exclusive alternative splicing of *Dscam* probably evolved in the common ancestor of both pancrustaceans and myriapods. According to our phylogenetic analysis, which included 12 paralogues, the *S. maritima Dscams* share a common origin and arose by duplication in the centipede lineage [107]. In the chelicerate *I. scapularis*, *Dscam* has also been duplicated extensively, both by whole-gene and by domain duplications [107]. These *Dscam* homologues however do not have a canonical domain composition and whether or not alternative splicing is also present in chelicerates remains unknown. The independent evolution of Dscam diversification in different arthropod groups (one locus with dozens of exon duplications in pancrustaceans versus many gene duplications coupled with a few exon duplications in *S. maritima* (Figure 8) suggests that the functional diversity in adhesion properties was important in the early evolution of arthropods. Whether all of these genes function in the immune system or nervous system development remains to be determined.

The short-interfering RNA (siRNA) pathway is the primary defence of insects against RNA viruses, while the piRNA pathway silences transposable elements in the germ line and micro RNAs (miRNAs) function in gene regulation [108]. These RNAi pathways appear to be intact in *S. maritima*, as we found homologues of key genes, including *Ago1* and *Dicer-1* in the miRNA pathway, *Ago2* and *Dcr2* in the siRNA pathway, and *Ago3* and *piwi* in the piRNA pathway (Table S30). We found two paralogues of *Ago2* and three paralogues of *piwi*, suggesting that RNAi may be more complex than in *D. melanogaster*. In other arthropods, expansion of the piwi family has been linked to neo- or subfunctionalization of germ line and soma roles, and so it remains to be seen whether this is also the case for *S. maritima*.

### Selenoproteins

Selenoproteins are peculiar proteins including a selenocysteine (Sec) residue, a very reactive amino acid typically found in the catalytic site of redox proteins, which is inserted through the recoding of a UGA codon [109]. While vertebrates possess 24–38 selenoproteins [110], insects have very few (*D. melanogaster* has three) or none at all. Several events of complete selenoproteome loss have been observed in insects [111]. These were ascribed to the fundamental differences in the insect antioxidant systems, which would favour selenoprotein loss or their conversion to standard proteins (cysteine homologues). The analysis of a myriapod selenoproteome is then crucial for a phylogenetic mapping of such differences.

The *S. maritima* genome was found to be surprisingly rich in selenoproteins: we have identified 20 predicted proteins (Table S26). Downstream of the coding sequence of each selenoprotein gene, we detected a selenocysteine insertion sequence (SECIS) element, the stem-loop structure necessary to target the Sec recoding machinery during selenoprotein translation. The full set of factors necessary for selenocysteine insertion and production was also found: tRNA-Sec, SecS, SBP2, eEFsec, pstk, secp43, SPS2. The centipede selenoproteome is rather similar to that predicted for the ancestral vertebrate (see [110]). This supports the idea that selenoprotein losses are specific to insects and can be ascribed to changes in that lineage, supporting the idea that a massive selenoproteome reduction occurred specifically in insects. A notable difference with vertebrates was found for the protein methionine sulfoxide reductase A (MsrA). This enzyme catalyzes the reduction of methionine-L-oxide to methionine, repairing proteins that were inactivated by oxidation. A selenoenzyme from this family has been previously characterized in the green alga *Chlamydomonas*, and selenocysteine containing forms were also observed in some non-insect arthropods [112]. In contrast, only cysteine homologues are present in vertebrate and insect genomes. We found a Sec-containing MsrA in the centipede genome, as well as in arthropods *D. pulex*, *I. scapularis*, and also in the chordate *B. floridae*. This, along with phylogenetic reconstruction analysis, supports the idea that the selenoprotein MsrA was present in their last common ancestor, and was later converted to a cysteine homologue independently in insects and vertebrates.

The two major antioxidant selenoprotein families in vertebrates, glutathione peroxidases (GPx), and thioredoxin reductases (TrxR), were also found with selenocysteine in the centipede genome. In contrast, all holometabolous insects possess only cysteine forms, and consistently, important differences were noted in these and other enzymes in the glutathione and thioredoxin system (see [113] for an overview). Thus, on the basis of gene content, we expect the antioxidant systems of *S. maritima* to be more similar to vertebrates and other animals than to holometabolous insects like *D. melanogaster*.

### DNA Methylation

Invertebrate DNA methylation occurs predominantly on gene bodies (exons and introns), via addition of a methyl group to a cytosine residue in a CpG context [114]–[116]. The exact function of gene body methylation is currently unknown, though it is correlated with active transcription in a wide range of species [116], and has been implicated in alternative splicing [117],[118] and regulation of chromatin organization [118]. Methylated cytosines are susceptible to deamination, to form a uracil, which is recognized as a thymine. Thus, over evolutionary time, highly methylated genes (in germ-line cells) will have comparatively low CpG content. The “observed CpG/expected CpG” (CpG_(o/e)_) ratio is an indicator of C-methylation: plots of CpG_(o/e)_ for a gene set produce a bimodal distribution where a proportion of the genes have an evolutionary history of methylation [119]. In contrast, species without methylation systems, such as *D. melanogaster*, yield a unimodal distribution [119].

**Figure 9:**
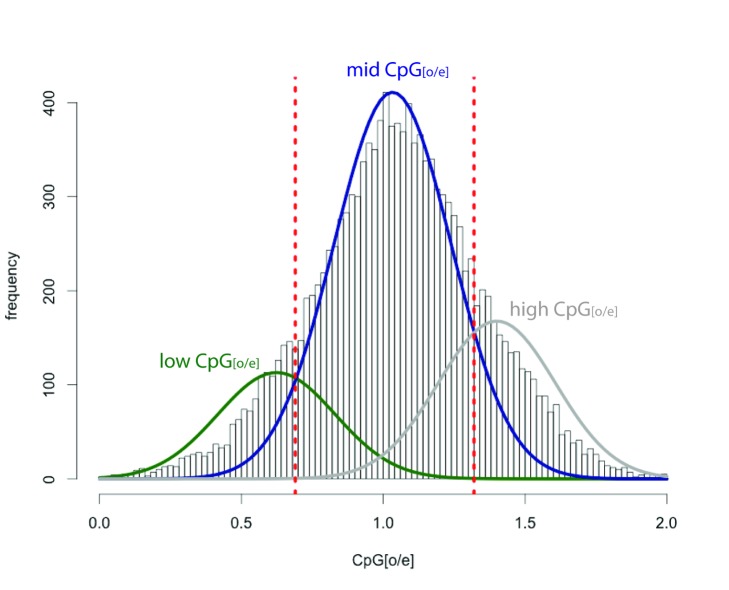
Frequency histogram of CpG_(o/e)_ observed in *S. maritima* gene bodies. The y-axis depicts the number of genes with the specific CpG_(o/e)_ values given on the x-axis. The distribution of CpG_(o/e)_ in *S. maritima* is a trimodal distribution, with a low-CpG_(o/e)_ peak consistent with the presence of historical DNA methylation in *S. maritima* and the presence of a high CpG_(o/e)_ peak. The data underlying this plot are available in File S4.

The *S. maritima* gene body CpG_(o/e)_ plot has a trimodal distribution, with the majority of genes having a ratio close to 1 (Figure 9; Text S1). Underlying this major peak are two smaller peaks, one “low” and one “high” centred around ratios of 0.62 and 1.48, respectively. This “high” peak, that contains genes with higher than expected CpG content, is unusual and is not seen in this analysis of other arthropods [91],[119]–[121]. Applying the same analysis to 1,000 bp windows across the entire genome (including both coding and non-coding regions) reveals a similar peak of high CpG content (Figure S29). This implies that the peak of “high” CpG content seen in gene bodies is due to unusually high CpG content in some regions of the genome rather than a specific feature of those coding regions. The “low” peak, however, indicates that 9.5% of genes have been methylated in the germ-line over evolutionary time. The number of genes contained within the “low” peak in *S. maritima* is smaller than observed in insect species with methylation, which can be as high as 40% in exceptional species such as the pea aphid and the honeybee [119],[120], where the mechanism is likely involved in polyphenism and caste differences respectively. However, the number of genes methylated is less in non-social hymenopteran such as *Nasonia vitripennis*, in beetles, and in mites [91],[121],[122]. Consistent with the low-levels of germ-line methylation detected, the genome contains a single orthologue of the de novo DNA methylation enzyme Dnmt3 and four orthologues of the maintenance DNA methyltransferases Dnmt1(a–d). Two of the Dnmt1 orthologues have lost amino acids that are required for methyltransferase activity, but these genes are represented in the transcriptome data, and are thus unlikely to be pseudogenes. One Dnmt1 gene shows sex-specific splicing, with a shorter transcript producing a truncated protein seen in female-derived transcription libraries. We also find a single orthologue of Tet1, a putative DNA demethylation enzyme [123],[124]. Taken together these data indicate that *S. maritima* has an active DNA methylation system, and that over evolutionary time a small number of genes have been methylated in the germ-line, resulting in a lower than expected CpG dinucleotide content.

### Non-Protein-Coding RNAs in the *S. maritima* Genome

We annotated over 900 homologues of known non-coding RNAs in the *S. maritima* genome, including over 600 predicted tRNAs (plus an additional 300 tRNA pseudogenes), 71 copies of 5S rRNA and 12 5.8S rRNAs, 88 copies of RNA components of the major spliceosome, and three out of the four RNA components of the minor U12 spliceosome, and 54 microRNA genes. As is common for whole genome assemblies, we did not identify intact copies of the 18S or 28S rRNAs. Further details of our methodology are provided in Text S1.

The predicted tRNA gene set includes all anticodons necessary to code for the 21 amino acids, including four potential SeC tRNAs. We identify a massive expansion of the tRNA-Ala-GGC family, with 322 sequences classified as functional tRNAs by tRNAscan-SE and an additional 172 classified as pseudogenes. These appear scattered throughout the scaffolds of the genome assembly. It is highly likely that the majority of these genes are pseudogenes, and the expansion may represent co-option of the tRNA into a transposable element.

Three *S. maritima* microRNA genes have been reported previously, and are available in the miRBase database (version 18) [125]. Two of these, mir-282 and mir-965, have homologues in crustaceans and insects. The third, mir-3930, is specific to myriapods [15]. In addition, we found 52 homologues of known microRNAs (Figure S34). These include 28 homologues of the 34 ancient microRNA families found throughout the Bilateria [126]. Four of these families were previously reported to be lost at various stages during animal evolution and, consistent with this, we failed to identify them in the *S. maritima* genome. Surprisingly, we also could not identify the *S. maritima* homologue of mir-125, a member of the ancient mir-100/let-7/mir-125 cluster, which is found in almost all bilaterians and has a well-established function in the regulation of development of many species [127]–[129]. Mir-100 and let-7 are well-conserved and localized within a 1 kb region on the same scaffold in *S. maritima*. Whilst we cannot rule out the possibility that the missing mir-125 is an artefact of the draft-quality genome assembly, the size of the scaffold strongly suggests that it is not present in the mir-100/let-7 cluster. We also identified 17 homologues of microRNAs common to ecdysozoans, and nine microRNAs known only from arthropods. Among the former, there are five homologues of mir-2 localized in close proximity to each other and downstream of mir-71. This clustering is conserved across protostomes, and it has previously been shown that the mir-2 family underwent various expansions during evolution [130]. Finally, we discovered a homologue of mir-2788, which was previously only known from insects, suggesting that this microRNA had an earlier origin.

### Conclusions

The sequencing of the centipede genome extends significantly the diversity of available arthropod genomes, and provides novel information pertinent to a range of evolutionary questions. Myriapods show a simple body organization that has remained relatively unchanged in comparison to their ancestors from the Silurian or even earlier [6], leading to an expectation of general conservatism. The myriapods are descendants of an independent terrestrialisation event from the hexapods and chelicerates, opening the opportunity for studying convergent evolution in these taxa. Naturally, *S. maritima* itself has its own evolutionary history, including both lineage specific features of the geophilomorphs and adaptations to their subterranean environment, allowing us to identify specific genomic signatures of ecological adaptations. Finally, the phylogenetic position of the myriapods within the arthropods has been the subject of intense debate for several years, and the availability of genomic data for a myriapod should contribute to the future resolution of this debate.

The morphological conservatism of centipedes is mirrored in many conservative aspects of the *S. maritima* genome. From the analyses of the various gene families outlined above it becomes clear that the *S. maritima* genome has undergone much less gene loss and rearrangement than the genomes of other sequenced arthropods, in particular those of the holometabolous insects such as *D. melanogaster*. This prototypical nature of the *S. maritima* genome is illustrated by the conservation of synteny relative to the arthropod and bilaterian ancestors, and the conservation of some ancient gene linkages and clustering, as seen for numerous homeobox genes. As such, the *S. maritima* genome can serve as a guide to the ancestral state of the arthropod genomes, or as a reference in the reconstruction of evolutionary events in the history of arthropod genomes.

**Figure 10:**
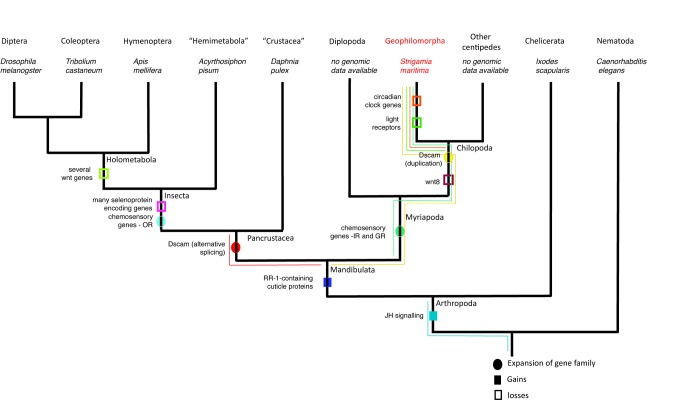
Arthropod phylogenetic tree (with nematode outgroup) showing selected events of gene loss, gene gain, and gene family expansions. Main taxa are listed on the tips, with representative species for which there is a fully sequenced genome listed below. Major nodes are also named. Data from the genome of *S. maritima* allow us to map when in arthropod evolution these events occurred, even when these events did not occur on the centipede lineage. A plausible node for the occurrence of each event is marked and colour-coded, with the possible range marked with a thin line of the same colour. The events, listed from left to right are: (1) Dscam alternative splicing as a strategy for increasing immune diversity is known from *D. melanogaster*, as well as the crustacean *D. pulex*, and thus probably evolved in the lineage leading to pancrustacea, after the split from centipedes. (2) Several wnt genes have been lost in holometabolous insects, leaving only seven of the 13 ancestral families. This loss occurred gradually over arthropod evolution, but reached its peak at the base of the Holometabola. (3) Selenoproteins are rare in insects. The presence of a large number of selenoproteins in *S. maritima* as well as in other non-insect arthropods suggests that the loss of many selenoproteins occurred at the base of the Insecta. (4) Expansion of chemosensory gene families occurred independently in different arthropod lineages as they underwent terrestrialisation. The OR family is expanded in insects only. (5) Chemosensory genes of the GR and IR genes have undergone a lineage specific expansion in the genome of *S. maritima*. As these are probably also linked with terrestrialisation we suggest that this expansion happened at the base of the Chilopoda, but it could have also occurred later in the lineage leading to *S. maritima*. (6) Cuticular proteins of the RR-1 family are numerous in the *S. maritima* genome. They are found in other arthropods, but not in chelicerates nor in any non-arthropod species. This suggests that the RR-1 subfamily evolved at the base of the Mandibulata. (7) The genome of *S. maritima* has a large complement of wnt genes, but is missing *wnt8*. Since this gene is found in the Diplopod *G. marginata* (a species without a fully sequenced genome), the loss most likely occurred at the base of the Chilopoda. (8) Unlike the situation in *D. melanogaster*, immune diversity in the *S. maritima* genome is achieved through multiple copies of the Dscam gene. This expansion of the family could have happened at any time after the split between Myriapoda and Pancrustacea. (9) Both circadian rhythm genes and many light receptors are missing in *S. maritima*. These losses are most likely due to the subterranean life style of geophilomorph centipedes and are probably specific to this group. However, we cannot rule out the possibility that they were lost somewhere in the lineage leading to myriapods. (10) The existence of JH signalling in *S. maritima* as well as in all other arthropods studied to date strengthens the idea that this signalling system evolved with the exoskeleton of arthropods, though its origins could be even more ancient and date back to the origin of moulting at the base of the Ecdysozoa.

The independent terrestrialisation of the myriapods and insects is evidenced by the use of different evolutionary solutions to similar problems. Figure 10 summarizes some of the gene gains and losses observed. We see this most clearly in the independent expansions of gustatory receptor proteins in myriapods and insects and the differential expansions of ionotropic and odorant receptors to deal with terrestrial chemosensation in the two lineages. Similarly, though probably not for the same reasons, we see a divergent solution for the generation of Dscam diversity in the immune response through the use of paralogues instead of the insect strategy of alternative splicing. The chelicerates also attained terrestriality independently. However, our understanding of chelicerate genomes still lags behind our understanding of insect, and now myriapod, genomes. Thus, extending this comparison to chelicerates, intriguing as it may be, will have to await future analysis of their genomes.

Lineage specific features of the *S. maritima* genome include the apparent loss of all known photoreceptors and a loss of the canonical circadian clock system based around *period* and its associated gene network. The characterization of whether *S. maritima* does have a circadian clock, and if it does how this is controlled, awaits further work, as does the pinpointing of when in their evolutionary history these systems were lost. The absence of the microRNA miR-125 is another surprising evolutionary loss. The extensive rearrangement of the mitochondrial genome is striking in comparison with the general conservatism seen in other known arthropod mitochondrial genomes, and especially in contrast with the conservative nature of *S. maritima*'s nuclear genome.

## Materials and Methods

The *S. maritima* raw sequence, and assembled genome sequence data are available at the NCBI under bioproject PRJNA20501 (http://www.ncbi.nlm.nih.gov/bioproject/PRJNA20501) Assembly ID GCA_000239455.1. The genome was sequenced using 454 sequencing technology, assembled using the celera assembler, annotated using a combination of the Maker 2.0 pipeline, and custom perl scripts followed by manual annotation of selected genes. Text S1 includes detailed methods for these steps, and additionally for the individuals sequenced, library construction and sequencing protocols used, repeat analysis, RNA sequencing, phylome db analysis, specific protocols for manual annotation of gene families, CpG analysis, and phylome and synteny re-construction.

## Supporting Information

Figure S1 **Frequency histogram showing the distribution of gene lengths in the** ***S. maritima*** **genome.** Gene length data used in this plot are available in File S4.
(PDF)

Figure S2 **Multi-gene phylogeny for the 18 species included in the phylogenomics analysis.** 1,491 widespread single-copy sets of orthologue sequences in at least 15 out of the 18 species were concatenated into a single alignment of 842,150 columns. Then, a maximum-likelihood tree was inferred using LG as evolutionary model by using PhyML.
(PDF)

Figure S3 **Multi-gene phylogeny for 12 species included in the phylogenomics analysis plus five additional Chelicerata species.** 1,491 widespread single-copy sets of orthologue sequences were concatenated into a single alignment of 829,729 positions. Then, a maximum-likelihood tree was inferred using LG as the evolutionary model by using PhyML.
(PDF)

Figure S4 **Alternative topological placements of** ***S. maritima*** **relative to the main arthropod groups considered in the study: Chelicerata and Pancrustacea.** Internal organization of each group was initially collapsed and, therefore, optimized during maximum-likelihood reconstruction.
(PDF)

Figure S5 **Clusters of genes specifically expanded in the centipede lineage.** On the plot, only clusters grouping five or more protein-coding genes were considered. The data underlying this plot are available in File S4.
(PDF)

Figure S6 **Mitochondrial gene organisation.** Shaded regions represent differences from the ground pattern. Gene translocations in Myriapoda have been noted in *Scutigerella causeyae* (Myriapoda: Symphyla) [49]. The previous example of the small conserved region trnaF-nad5-H-nad4-nad4L on the minus strand between *Limulus*, *Lithobius*, and *Strigamia* is not a conserved feature in all Chilopoda, because *Scutigera colepotrata* have an interruption between nad5 and H-nad4 with elements on the minus and plus strands accompanied by a translocation of nad4L to a position immediately preceding nad5.
(PDF)

Figure S7 **Classification of all** ***S. maritima*** **(Sma) homeodomains (excluding Pax2/5/8/sv) via phylogenetic analysis using** ***T. castaneum*** **(Tca) and** ***B. floridae*** **(Bfl) homeodomains.** This phylogenetic analysis was constructed using neighbour-joining with a JTT distance matrix and 1,000 bootstrap replicates. Gene classes are indicated by colours. The genes coloured in grey are those genes that cannot be assigned to known classes. Further classification was performed using additional domains outside the homeodomain and by performing additional phylogenetic analysis for particular gene classes using maximum-likelihood and bayesian approaches. Pax2/5/8/sv is excluded due to the gene possessing only a partial homeobox.
(PDF)

Figure S8 **Phylogenetic analysis of ANTP class homeodomains of** ***S. maritima*** **(Sma) using** ***T. castaneum*** **(Tca) and** ***B. floridae*** **(Bfl) for comparison.** These phylogenetic analyses were constructed using neighbour-joining with a JTT distance matrix, 1,000 bootstrap replicates (support values in black). Nodes with support equal to or above 500 in the maximum-likelihood (LG+G) analysis are in blue and nodes with posterior probabilities equal to or above 0.5 (LG+G) in the Bayesian analysis are in red.
(PDF)

Figure S9 **Phylogenetic analysis of PRD class homeodomains of** ***S. maritima*** **(Sma) using** ***T. castaneum*** **(Tca) and** ***B. floridae*** **(Bfl) for comparison.** These phylogenetic analyses were constructed using neighbour-joining with a JTT distance matrix, 1,000 bootstrap replicates (support values in black). Nodes with support equal to or above 500 in the maximum-likelihood (LG+G) analysis are in blue and nodes with posterior probabilities equal to or above 0.5 (LG+G) in the Bayesian analysis are in red.
(PDF)

Figure S10 **Phylogenetic analysis of HNF class homeodomains of** ***S. maritima*** **(Sma) using** ***B. floridae*** **(Bfl), human (** ***Homo sapiens*** **, Hsa), and sea anemone (** ***N. vectensis*** **, Nve) for comparison.** These phylogenetic analyses were constructed using neighbour-joining with a JTT distance matrix, 1,000 bootstrap replicates (support values in black). Nodes with support equal to or above 500 in the maximum-likelihood (LG+G) analysis are in blue and nodes with posterior probabilities equal to or above 0.5 (LG+G) in the Bayesian analysis are in red.
(PDF)

Figure S11 **Phylogenetic analysis of Xlox/Hox3 genes of** ***S. maritima*** **(Sma) using a selection of Hox1, Hox2, Hox3, Hox4, and Xlox sequences.** This analysis was based upon the whole coding sequence of the genes, and was constructed using neighbour-joining with a JTT distance matrix and 1,000 bootstrap replicates. The blue support value (of 333) is the node that reveals the affinity between the Xlox/Hox3 genes of *S. maritima* and Xlox sequences. Ame, *A. mellifera*; Bfl, *B. floridae*; Cte, *Capitella teleta*; Dme, *D. melanogaster*; Lgi, *Lottia gigantea*; and Tca, *T. castaneum*.
(PDF)

Figure S12 **Multiple alignment of relevant residues of the Hox1, Hox2, Hox3, Hox4, and Xlox sequences of different lineages compared to** ***S. maritima*** **Hox3a and Hox3b sequences.** Three paired class genes are included as an outgroup. The grading of purple colouring of the amino acids shows the identity level of these sequences. The red rectangles in the multiple alignment delimit the core of the hexapeptide motif and the homeodomain. This is the alignment used to construct the phylogenetic tree in Figure S13. Ame, *A. mellifera*; Bfl, *B. floridae*; Cte, *Capitella teleta*; Dme, *D. melanogaster*; Lgi, *Lottia gigantea*; and Tca, *T. castaneum*.
(PDF)

Figure S13 **Phylogenetic analysis of** ***S. maritima*** **Xlox/Hox3 homeodomain and hexapeptide motifs using a selection of Hox1, Hox2, Hox3, Hox4, and Xlox sequences.** This analysis used a section of the coding sequence including the hexapeptide and some flanking residues plus the homeodomain (alignment in Figure S12). Three paired class genes are included as an outgroup. This phylogeny was constructed using neighbour-joining with the JTT distance matrix and 1,000 bootstrap replicates. Maximum likelihood support values are shown in blue and Bayesian posterior probabilities in red. Ame, *A. mellifera*; Bfl, *B. floridae*; Cte, *Capitella teleta*; Dme, *D. melanogaster*; Lgi, *Lottia gigantean*; Tca, *T. castaneum*.
(PDF)

Figure S14 **Fisher's exact test to distinguish whether** ***S. maritima*** **scaffold 48457 has significant synteny conservation with ParaHox or Hox chromosomes of humans.** No significant Hox or ParaHox association is found.
(PDF)

Figure S15 **Phylogenetic analysis of TALE class homeodomains of** ***S. maritima*** **(Sma) using** ***T. castaneum*** **(Tca) and** ***B. floridae*** **(Bfl), including the Iroquois/Irx genes.** These phylogenetic analyses were constructed using neighbour-joining with a JTT distance matrix, 1,000 bootstrap replicates (support values in black). Nodes with support equal to or above 500 in the maximum-likelihood (LG+G) analysis are in blue and nodes with posterior probabilities equal to or above 0.5 (LG+G) in the Bayesian analysis are in red.
(PDF)

Figure S16 **RNA processing in the Hox cluster of** ***S. maritima*** **.** The transcriptome of *S. maritima (Sm)* eggs (blue), females (green), and males (red) was mapped to the Hox gene cluster (top panel; see Figure 4 in the main text) and transcript models were inferred for each gene within the cluster (shaded area) taking into account the presence of ORF and polyadenylation signals (PAS) to support the existence of RNA processing events. We note the occurrence of more than one mRNA isoform of six *S. maritima* Hox genes (i.e., *Antp*, *Ubx*, *abd-A*, *lab*, *Dfd*, *pb*). In all these six cases alternative polyadenylation (APA) generates mRNAs bearing distinct 3′ UTRs (alternative UTR sizes at the bottom). Alternative splicing (AS) with concomitant alternative promoter use (APU) events concern two *S. maritima* Hox genes *Dfd* and *ftz* (see alternative ORF sizes at the bottom). We also see that some genes such as *S. maritima Ubx* display high heterogeneity in 3′UTR sequences within the embryonic transcriptome (“eggs” data) suggesting the possibility that *S. maritima Ubx* APA might be developmentally controlled and/or display a tissue-specific pattern (see inset for further details on symbols).
(PDF)

Figure S17 **RNA processing in the** ***S. maritima*** **and** ***D. melanogaster*** **Hox clusters.** (A) The incidence of alternatively processed mRNAs is comparable between *S. maritima* and *D. melanogaster*, in that over 75% of the *S. maritima* Hox genes undergo RNA processing of one type or another. Similarly, seven out of the eight *Drosophila* Hox genes produce different mRNA isoforms (FlyBase, http://flybase.org/). (B) Three *D. melanogaster* Hox genes undergo AS (blue) and five produce different transcripts via APA (red, FlyBase http://flybase.org/). In addition five fruit fly Hox genes form different RNA species by APU (green). (C) Classification of all alternatively processed mRNA events in the *S. maritima* Hox cluster based on the same categorisation as in (B). Note that patterns of AS and APA affecting *S. maritima* and *D. melanogaster* Hox genes are relatively comparable; in contrast, APU seems more prevalent in the *Drosophila* (five out of eight genes) than in the centipede (two out of nine genes) Hox genes.
(PDF)

Figure S18 **Phylogenetic tree of the** ***S. maritima*** **,** ***D. pulex*** **,** ***I. scapularis*** **, and representative insect GRs, part one.** This is a corrected distance tree and was rooted at the midpoint in the absence of a clear outgroup, an approach that clearly indicates the distinctiveness of the centipede GRs. It is a more detailed version of Figure 5A. The *S. maritima*, *D. pulex*, *I. scapularis*, and representative insect gene/protein names are highlighted in red, blue, brown, and green, respectively, as are the branches leading to them to emphasize gene lineages. Bootstrap support levels in percentage of 10,000 replications of neighbour-joining with uncorrected distance is shown above major branches. Comments on major gene lineages are on the right. Suffixes after the gene/protein names are: PSE, pseudogene; FIX, sequence fixed with raw reads; JOI, gene model joined across scaffolds. Note than in Figure 5A for space reasons the IsGr47 and 59 proteins are included in the carbon dioxide and sugar receptor groupings, respectively; however, there is no bootstrap support for these branches, and no such functional assignment is claimed. Similarly, it is unlikely that the DpGr57/58 proteins are fructose receptors.
(PDF)

Figure S19 **Phylogenetic tree of the** ***S. maritima*** **,** ***D. pulex*** **,** ***I. scapularis*** **, and representative insect GRs, part two.** This is a corrected distance tree and was rooted at the midpoint in the absence of a clear outgroup, an approach that clearly indicates the distinctiveness of the centipede GRs. It is a more detailed version of Figure 5A. The *S. maritima*, *D. pulex*, *I. scapularis*, and representative insect gene/protein names are highlighted in red, blue, brown, and green, respectively, as are the branches leading to them to emphasize gene lineages. Bootstrap support levels in percentage of 10,000 replications of neighbour-joining with uncorrected distance is shown above major branches. Comments on major gene lineages are on the right. Suffixes after the gene/protein names are: PSE, pseudogene; FIX, sequence fixed with raw reads; JOI, gene model joined across scaffolds. Note than in Figure 5A for space reasons the IsGr47 and 59 proteins are included in the carbon dioxide and sugar receptor groupings, respectively; however, there is no bootstrap support for these branches, and no such functional assignment is claimed. Similarly, it is unlikely that the DpGr57/58 proteins are fructose receptors.
(PDF)

Figure S20 **Neuropeptide precursor sequences identified in the** ***S. maritima*** **genome.** The putative signal peptides (predicted by SignalP) are underlined, the putative active neuropeptides or protein hormones (based on similarity to neuropeptides or protein hormones identified in other invertebrates) are marked in yellow. Green indicates putative basic cleavage sites flanking the putative neuropeptides. Glycines used for amidation are shown in blue, cysteines proposed to form cysteine bridges are shown in red. Dots indicate missing N- or C-termini.
(DOCX)

Figure S21 **Examples of tandem duplications of neuropeptide receptor genes.** Structure of the two inotocin receptor genes found head-to-head on opposite strands of scaffold JH431865 (A). Structure of the two SIFamide receptor genes found tail-to-head on the same strand of scaffold JH432116 (B).
(PDF)

Figure S22 **Schematic diagram showing sesquiterpenoids/juvenoids synthesis (upper) and degradation (lower) pathways in arthropods.** Molecules/hormones in synthesis are shown in bold, enzymes are shown in italics, and species/clades are shown in bold italics.
(PDF)

Figure S23 **Phylogenetic analysis of the TGFβ ligands in arthropods.** See Text S1 for details. Abbreviations: Ag, *Anopheles gambiae*; Am, *A. mellifera*; Ap, *Acyrthosiphon pisum*; Ca, *Clogmia albipunctata*; Dm, *Drosophila melanogaster*; Dp, *D. pulex*; Is, *I. scapularis*; Lg, *Lottia gigantea* Ma, *Megaselia abdita*; Nv, *Nasonia vitripennis*; Ph, *Pediculus humanus*; Tc, *T. castaneum*;.
(EPS)

Figure S24 **Range of Wnt genes present in** ***S. maritima*** **.** Wnt genes present and number of *Wnt* subfamilies absent in *S. maritima* in comparison with other arthropods and three non-arthropod outgroups.
(TIF)

Figure S25 **Phylogeny of FGFR genes indicating that FGFR genes duplicated independently in** ***S. maritima*** **and** ***D. melanogaster*** **.** See text for details. Alignment was performed using Clustal-Omega (http://www.ebi.ac.uk/Tools/services/web). The evolutionary history was inferred using the neighbour-joining method with bootstrapping to determine node support values (10,000 replicates). The evolutionary distances were computed using the Poisson correction method. Evolutionary analyses were conducted in MEGA5.
(EPS)

Figure S26 **Phylogeny including the three FGF genes of** ***S. maritima*** **.** See text for details. Alignment was performed using Clustal-Omega (http://www.ebi.ac.uk/Tools/services/web). The evolutionary history was inferred using the neighbour-joining method with bootstrapping to determine node support values (10,000 replicates). The evolutionary distances were computed using the Poisson correction method. Evolutionary analyses were conducted in MEGA5.
(EPS)

Figure S27 ***Cap ‘n’ collar (cnc)*** **genes.** (A) The two genes are located on different scaffolds. *Cnc1* is a long transcript consisting of 11 exons. *Cnc2* is shorter (eight exons), the three exons at the 3′ end of the gene that encode the C-terminal region of the protein including the conserved domain (B) show a similar structure. (B) *S. maritima* Cnc protein structure. Both proteins contain the bZip domain in a similar position at the C-terminus. *Cnc1* encodes a long protein (925 amino acids). Bits of the N-terminal region (blue lines) align with *D. melanogaster* Cnc isoform C and *T. castaneum* Cnc variant A. (C) Cnc protein sequence alignment, only showing the aligning bits in the N-terminal region. Blue lines show short stretches of sequence that form a consensus motif. These motifs are not present in the proteins encoded by *Sm-cnc2*, *Dm-cnc* isoforms A and B, and *T. castaneum cnc* variant B.
(JPG)

Figure S28 **Frequency histograms of observed versus expected dinucleotide content in** ***S. maritima*** **gene bodies.** (A–P) The y-axis depicts the number of genes with the specific dinucleotide_[o/e]_ values given on the x-axis. The distribution of all dinucleotide pairs, with the exception of CpG, is best described as a unimodal distribution. The distribution of CpG dinucleotides is best described as a trimodal distribution, with “high” and “low” CpG_[o/e]_ classes. The data underlying this figure are available in File S5.
(TIF)

Figure S29 **Frequency histogram of CpG_[o/e]_ observed in 1,000 bp windows of the** ***S. maritima*** **genome.** The y-axis depicts the number of genes with the specific CpG_[o/e]_ values given on the x-axis. The distribution of CpG_[o/e]_ in *S. maritima* genome is a bimodal distribution, with a high CpG_[o/e]_ peak observed similar to that observed in the gene bodies (Figure 9). The data underlying this figure are available in File S6.
(TIF)

Figure S30 **Contrasting patterns of DNA methylation, as measured by over- and underrepresentation of CpG dinucleotides in coding regions (CpG_(o/e)_), within arthropod species.** In all graphs the y-axis depicts the number of genes with the specific CpG_(o/e)_ values given on the x-axis. (A) *D. melanogaster* coding regions show a unimodal peak reflective of the lack of DNA methylation in this species. (B) *A. mellifera* shows a bimodal peak consisting of genes with a lower than expected CpG_(o/e)_ (green distribution) and a higher than expected CpG_(o/e)_ (blue distribution). The presence of a bimodal distribution in this species is consistent with depletion of CpG dinculeotides in the coding regions of genes over evolutionary time as a result of DNA methylation. (C) A single unimodal peak is also observed for *Tetranychus urticae*, a species that has very low levels of DNA methylation. (D) The *S. maritima* distribution is best explained as a mixture of three distinct distributions that we have deemed “low” (green distribution), “medium” (blue distribution), and “high” (grey distribution). The genes within the low distribution likely contain genes that are historically methylated, whilst the “high” distribution can be explained by regions of the genome that are comparatively CpG-rich (as determined by the analysis of the *S. maritima* genome, Figure S29). The data underlying this figure are available in File S7.
(PDF)

Figure S31 **Chromosomal organisation of histone gene clusters in** ***S. maritima*** **.** In insects such as *Drosophila* [115] and the pea aphid [109] histone encoding genes are present in quintet clusters, each cluster containing one gene from each of the five classes of histone. Only one such cluster could be identified in *S. maritima* (A). The other four clusters identified in the *S. maritima* genome (B–D) all consist of two to three copies of a histone encoding gene of a single class. It is possible that these have arisen as a result of recent gene duplication.
(EPS)

Figure S32 ***S. maritima vasa*** **DEAD-box helicase germline gene phylogeny.** Maximum likelihood tree of *vasa/PL10* family genes. One gene is a likely *vasa* orthologue (SMAR015390), one groups with the *PL10* family (SMAR005518), and the majority group in an apparently distinct DEAD-box-containing clade. Bootstrap values for 2,000 replicates are shown at each node. Accession numbers for protein sequences are as follows: *Apis* Belle (XP_391829.3), *Apis* Vasa (NP_001035345.1), *Danio* PL10 (NP_571016.2), *Danio* Vasa (AAI29276.1), *Drosophila* Belle (NP_536783.1), *Drosophila* Vasa (NP_723899.1), *Gryllus* Vasa (BAG65665.1), *Mus* Mvh (NP_001139357.1), *Mus* PL10 (NP_149068.1), *Nasonia* Belle (XP_001605842.1), *Nasonia* Vasa (XP_001603956.2), *Nematostella* PL10 (XP_001627306.1), *Nematostella* Vasa 1 (XP_001628238.1), *Nematostella* Vasa 2 (XP_001639051.1), *Oncopeltus* Vasa (AGJ83330.1), *Parhyale* Vasa (ABX76969.1), *Tribolium* Belle (NP_001153721.1), *Tribolium* Vasa (NP_001034520.2), *Xenopus* PL10 (NP_001080283.1), *Xenopus* VLG1 (NP_001081728.1).
(EPS)

Figure S33 **Phylogenomic inventory of meiotic genes in arthropods.** Red genes are specific to meiosis in model species in which functional data are available. “+” and “−” indicate the presence and absence of orthologues, respectively. Numbers indicate copy number of duplicated genes.
(PDF)

Figure S34 **Patterns of microRNA gain and loss across the animal kingdom with the inclusion of** ***S. maritima*** **.** The number of microRNAs that were gained or lost at each node are shown in green and red, respectively, and names are listed below each taxon. MicroRNAs that are found in the *S.maritima* genome are in bold, and families for which more than one homologue is found are marked with an asterisk. The tree depicts the Mandibulata hypothesis rather than the Myriochelata, as in [124].
(EPS)

Table S1 **Detailed overview for the repetitive elements in** ***S. maritima*** **.** For each group the number of elements (putative families), the number of their fragments or copies in the genome, the cumulative length, the proportion of the assembly, and some features are shown. This includes elements containing nested inserts of other elements (n), elements that appear to be complete (i.e., all typical structural and coding parts present, even if containing stop codons or frameshifts), elements with a RT or *Tase* domain detected (n), elements that potentially could be active as they contain an intact ORF with all the typical domains even though they could lack other structural features like terminal repeats, and elements that contain an intact ORF for the RT domain or parts of the *Tase* domain and could thus be partly active. The elements that could not be categorized or contained features of protein coding regions are shown at the bottom, whereby they probably do not belong to the transposable elements.
(XLSX)

Table S2 **Set of species used in the comparative genomics analyses related to the** ***S. maritima*** **genome.** Columns include, in this order, scientific names, the species code according to UNIPROT, the number of the longest unique transcript used in the analyses, the data source, and the date in which data were retrieved.
(DOCX)

Table S3 **Orthologues detected between a given species and** ***S. maritima*** **.** First column indicates how many trees have been used to detect such orthologues. Columns “uniq” refers to the number of orthologues detected for each pair of species after removing redundancy. In one-to-many and many-to-many orthology relationships it is possible to count a given protein more than once. Regarding the ratios values, “all” column refers to the orthology ratio computed using all orthologue pairs meanwhile “uniq” refers to the ratio computed using “uniq” columns.
(DOCX)

Table S4 **Orthology ratios for a given species related to** ***S. maritima*** **.** This table is similar to Table S3, but in this case orthology relationships with ten or more proteins for any of the species are discarded in order to avoid biases introduced by species-specific gene family expansions.
(DOCX)

Table S5 **Newly added Chelicerata species used to increase the taxon sampling for the species phylogeny.** First column indicates the scientific species name, the second one indicates which strategy has been used to identify single copy protein-coding genes. Third column shows how many single-copy genes have been identified in each species from the initial set of 1,491 used to reconstruct the species phylogeny. Last two columns show the data source and the date on which data were retrieved.
(DOCX)

Table S6 **Results after applying the different statistical tests implemented in CONSEL for the alternative placement of** ***S. maritima*** **relative to Pancrustacea and Chelicerata groups of species (as shown in Figure S4) in the context of the 18 species used for the phylogenomics analyses.** The “item” column relates to Figure S4 as follows: (1) topology arrangement corresponding to Figure S4 left-hand panel, in which *S. maritima* was grouped with Chelicerata species. (2) Topology arrangement corresponding to Figure S4 central panel, in which *S. maritima* branches off before the split of Pancrustacea and Chelicerata. (3) Topology arrangement corresponding to Figure S4 right-hand panel, in which *S. maritima* was grouped with Pancrustacea species.
(DOCX)

Table S7 **Results after applying the different statistical tests implemented in CONSEL for the alternative placement of** ***S. maritima*** **relative to the two arthropod groups, Pancrustacea and Chelicerata (as shown in Figure S4), with the inclusion of extra chelicerates.** Taxon sampling for the Chelicerata was increased after including sequences from five additional species. In order to reduce any potential bias introduced by distant and/or fast-evolving out-groups, six out-group species from the initial set were removed. The “item” column relates to Figure S4 as follows: (1) topology arrangement corresponding to Figure S4 left-hand panel, in which *S. maritima* was grouped with Chelicerata species. (2) Topology arrangement corresponding to Figure S4 central panel, in which *S. maritima* branches off before the split of Pancrustacea and Chelicerata. (3) Topology arrangement corresponding to Figure S4 right-hand panel, in which *S. maritima* was grouped with Pancrustacea species.
(DOCX)

Table S8 **Enriched functional GO Terms for the ten largest clusters of duplicated** ***S. maritima*** **protein-coding genes specifically expanded in the centipede lineage, as compared with the whole genome.**
(DOCX)

Table S9 **Statistics regarding the duplications of centipede genes relative to seven specific ages detected using all available trees on the phylome.**
(DOCX)

Table S10 **Enriched functional GO terms for proteins duplicated at the different relative ages shown in Table S9.** Columns show relative age, gene ontology namespace, the GO term id, and its name, respectively.
(DOCX)

Table S11 **Overview of** ***S. maritima*** **mitochondrial genome.**
(DOCX)

Table S12 **Species used in the synteny analyses and the sources of their sequence data.**
(DOCX)

Table S13 **Block-synteny summary statistics for pairs of species.** Hs, *Homo sapiens*; Bf, *B. floridae*; Sm, *S. maritima*; Lg, *Lottia gigantea*; Ct, *Capitella teleta*; Nv, *N. vectensis*; Ta, *Trichoplax adhaerens*; Ag, *Anopheles gambiae*; Bm, *B. mori*.
(DOCX)

Table S14 **Summary of numbers of homeobox genes per class of** ***Strigamia*** **,** ***Branchiostoma*** **, and** ***Tribolium*** **.**
(DOCX)

Table S15 **Names and identification numbers of all** ***S. maritima*** **homeobox genes along with their orthologues from the beetle,** ***T. castaneum*** **, and amphioxus,** ***B. floridae*** **.**
(XLS)

Table S16 **One-to-one** ***S. maritima*** **to human orthologues starting from genes on** ***S. maritima*** **scaffold 48457, which contains** ***SmaHox3a*** **.** The third column is the chromosomal location of the human orthologue. Human Hox chromosomes are 2, 7, 12, and 17 and the ParaHox chromosomes are 4, 5, 13, and X.
(DOCX)

Table S17 **Evolutionary conservation of RNA processing modes in the** ***S. maritima*** **and** ***D. melanogaster*** **Hox clusters.** Type of RNA processing event concerning each one of the *S. maritima* (left) and *D. melanogaster* (right) Hox genes. We note that orthologous genes in both species undergo similar types of RNA processing: the three posterior-most Hox genes: *Ubx*, *abd-a*, and *Abd-b* display a specific type of APA (tandem APA) in both *S. maritima* and *D. melanogaster* (conserved patterns highlighted by red asterisks) providing an example of what might be a feature present in the ancestral Hox cluster to insects and myriapods. Nonetheless, for most other Hox genes, RNA processing patterns differ markedly between *S. maritima* and *D. melanogaster*, indicating that the conserved incidence of alternative RNA processing across arthropods can only be proposed for the posterior-most Hox genes.
(PDF)

Table S18 **Details of SmGr family genes and proteins.** Columns are: Gene, the gene and protein name we are assigning (suffixes are PSE, pseudogene; FIX, assembly was repaired; JOI, gene model spans scaffolds); OGS, the official gene number in the 13,233 proteins (prefix is Smar_temp_); Scaffold, the genome assembly scaffold ID, prefix is scf718000 (amongst 14,739 scaffolds in assembly Smar05272011); Coordinates, the nucleotide range from the first position of the start codon to the last position of the stop codon in the scaffold; Strand – + is forward and − is reverse; introns, number of introns; ESTs, presence of an EST contig with appropriate splicing in one of the three transcriptome assemblies (F, female; M, male; E, eggs); AAs, number of encoded amino acids in the protein; comments, comments on the OGS gene model, repairs to the genome assembly, and pseudogene status (numbers in parentheses are the number of obvious pseudogenizing mutations).
(DOC)

Table S19 **Total numbers of biogenic amine receptors in different species.**
(DOCX)

Table S20 **A comparison between the** ***D. melanogaster*** **and** ***S. maritima*** **biogenic amine receptors.** The orthologues are given next to each other. When there is no orthologue, a dash (–) is written instead.
(XLSX)

Table S21 **Genes encoding neuropeptide precursors and neuropeptide receptors annotated in** ***S. maritima*** **.** Abbreviations: ACP, adipokinetic hormone/corazonin-related neuropeptide; AKH, adipokinetic hormone; ADF, antidiuretic factor; AST, allatostatin; CCAP, crustacean cardio-active peptides; DH (Calc.-like), calcitonin-like diuretic hormone; DH (CRF-like), corticotropin releasing factor-like diuretic hormone; EH, eclosion hormone; ETH, ecdysis triggering hormone; GPA2, glycoprotein hormone A2; GPB5, glycoprotein hormone B5; ILP, insulin-like peptide; ITP, ion transport peptide; NPF, neuropeptide F; NPLP, neuropeptide-like precursor; PDF, pigment dispersing factor; PTTH, prothoracicotropic hormone; sNPF, short neuropeptide F.
(EPS)

Table S22 **Presence or absence of neuropeptide signaling systems in arthropods.** The centipede *S. maritima* contains two CCHamide-1, two eclosion hormone and two FMRFamide genes (2 p). In some cases neuropeptide precursors could not be identified, but the corresponding receptor genes are present (R). We assume that this is due to sequencing gaps. For abbreviations see Table S21.
(DOC)

Table S23 **Genes commonly implicated in arthropod juvenoids biosynthesis (green) and degradation (blue), and their potential regulators (purple) [98]–[101].** Common abbreviations, and presence in the centipede *S. maritima*.
(DOCX)

Table S24 **List of genes commonly implicated as potential regulators of arthropod juvenoids biosynthesis (purple) [98]–[101].** Common abbreviations, and presence in the centipede *S. maritima*.
(DOCX)

Table S25 **Wnt genes in the genome of** ***S. maritima*** **.** SMAR, the gene identification number, and scaffold, the scaffold identification number. Wnt 1, 6, and 10 are clustered together on the same scaffold (yellow highlighting), which is likely a remnant of the ancestral wnt gene cluster (see text for details).
(PDF)

Table S26 **Selenoproteins in the** ***S. maritima*** **genome.**
(DOCX)

Table S27 **Histone encoding loci of** ***S. maritima*** **.**
(DOCX)

Table S28 **Number of loci within the genomes of arthropod species encoding the five classes of histones.** Orthologues for *A. aegypti*, *D. pulex*, *T. urticae*, and *I. scapularis* were obtained by BLAST analysis. Orthologues for *A. mellifera* and A. *pisum* were obtained from published literature [108],[109].
(DOCX)

Table S29 **Germ line and RNAi genes annotated in the** ***S. maritima*** **genome.** The name of the *Drosophila* orthologue is shown unless indicated with “(Mo),” for mouse.
(DOCX)

Table S30 **Details of the manually annotated genes of** ***S. maritima*** **.**
(XLSX)

File S1 **One2One_GOTerms_GenomeIDs for Orthology-based functional annotation.**
(XLSX)

File S2 **Strigamia_pals for** **Figure 3** **.**
(XLSX)

File S3 **Gustatory receptor sequences.**
(XLSX)

File S4 **Raw data for** **Figure 2** **,** **Figure 9** **, Figure S1, and Figure S5.**
(XLSX)

File S5 **Raw data for Figure S28.**
(XLSX)

File S6 **Raw data for Figure S29.**
(XLSX)

File S7 **Raw data for Figure S30.**
(XLSX)

Text S1 **Supporting Methods Text.**
(DOCX)

## Abbreviations

FGF: fibroblast growth factor
GR: gustatory receptor
GPCR: G protein-coupled receptor
JH: juvenile hormone
OR: odorant receptor
RR: Rebers and Riddiford
TGF: transforming growth factor
