# Supplementary figures and images for: The First Myriapod Genome Sequence Reveals Conservative Arthropod Gene Content and Genome Organisation in the Centipede Strigamia maritima
Source: PLoS Biol. 2014 Nov 25;12(11):e1002005. doi: 10.1371/journal.pbio.1002005 (PMC4244043; doi:10.1371/journal.pbio.1002005)

# *S. maritima* gene lengths

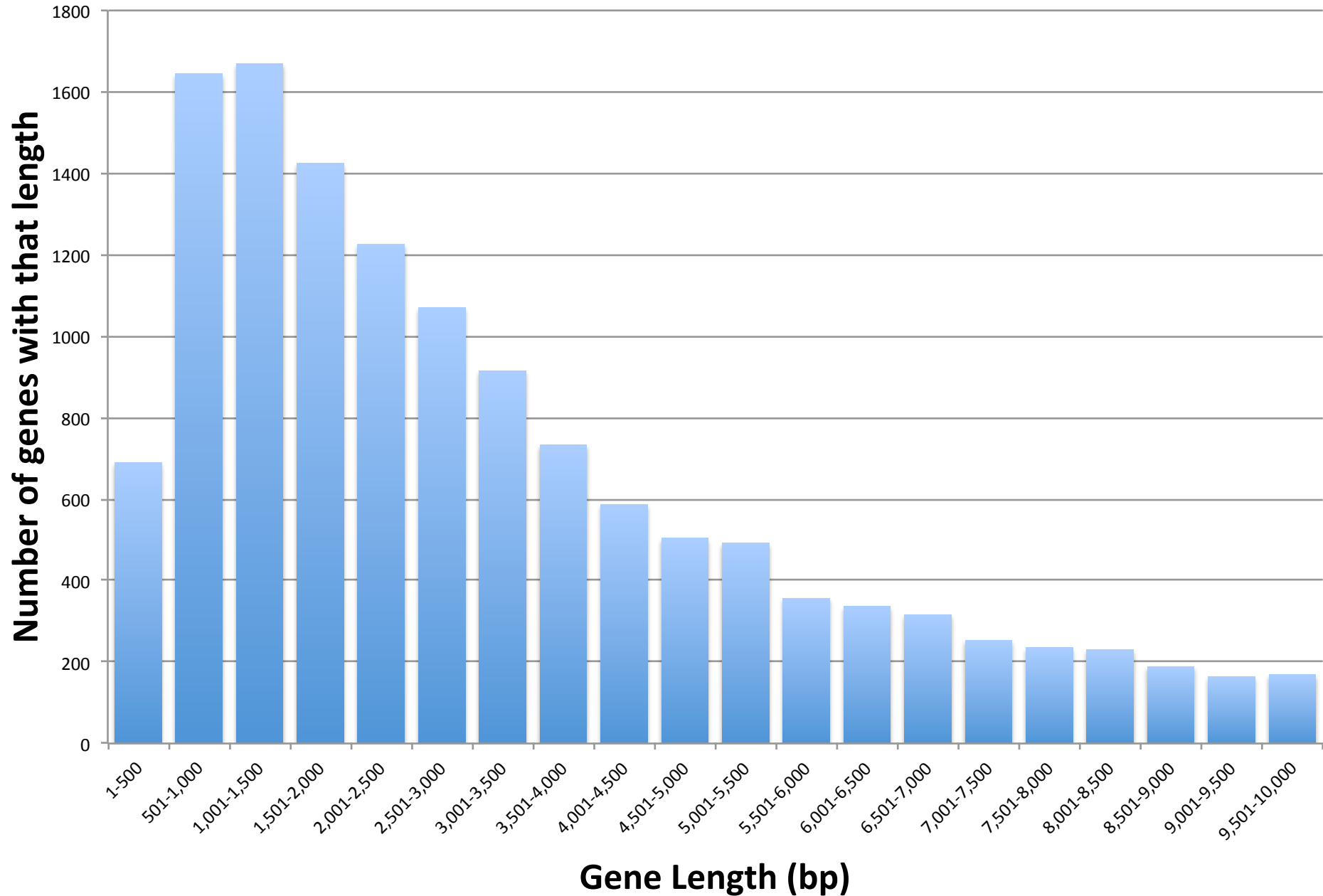

Supplement: Figure S1 — Frequency histogram showing the distribution of gene lengths in the S. maritima genome. Gene length data used in this plot are available in File S4. (PDF) [file pbio.1002005.s001.pdf]

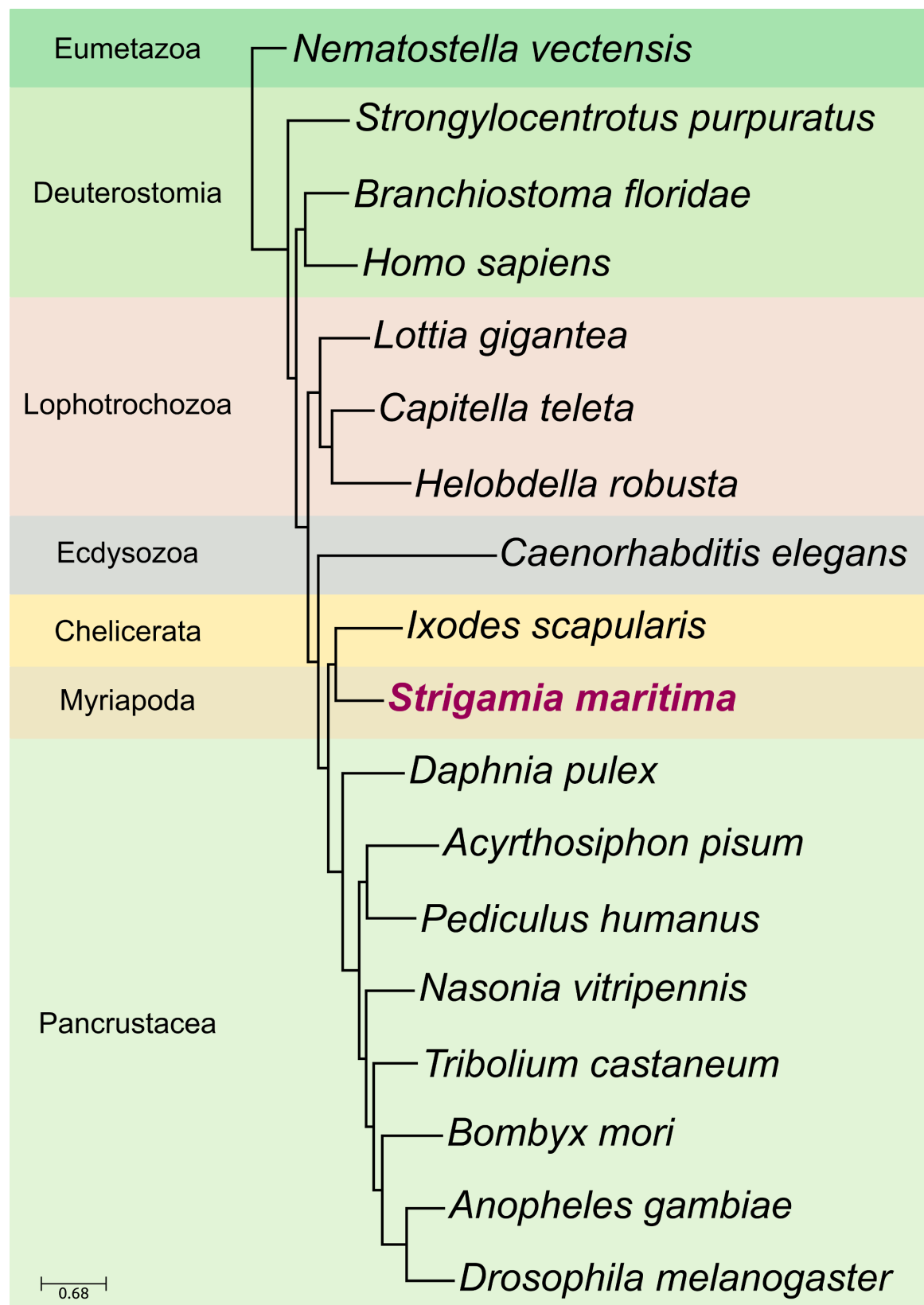

Supplement: Figure S2 — Multi-gene phylogeny for the 18 species included in the phylogenomics analysis. 1,491 widespread single-copy sets of orthologue sequences in at least 15 out of the 18 species were concatenated into a single alignment of 842,150 columns. Then, a maximum-likelihood tree was inferred using LG as evolutionary model by using PhyML. (PDF) [file pbio.1002005.s002.pdf]

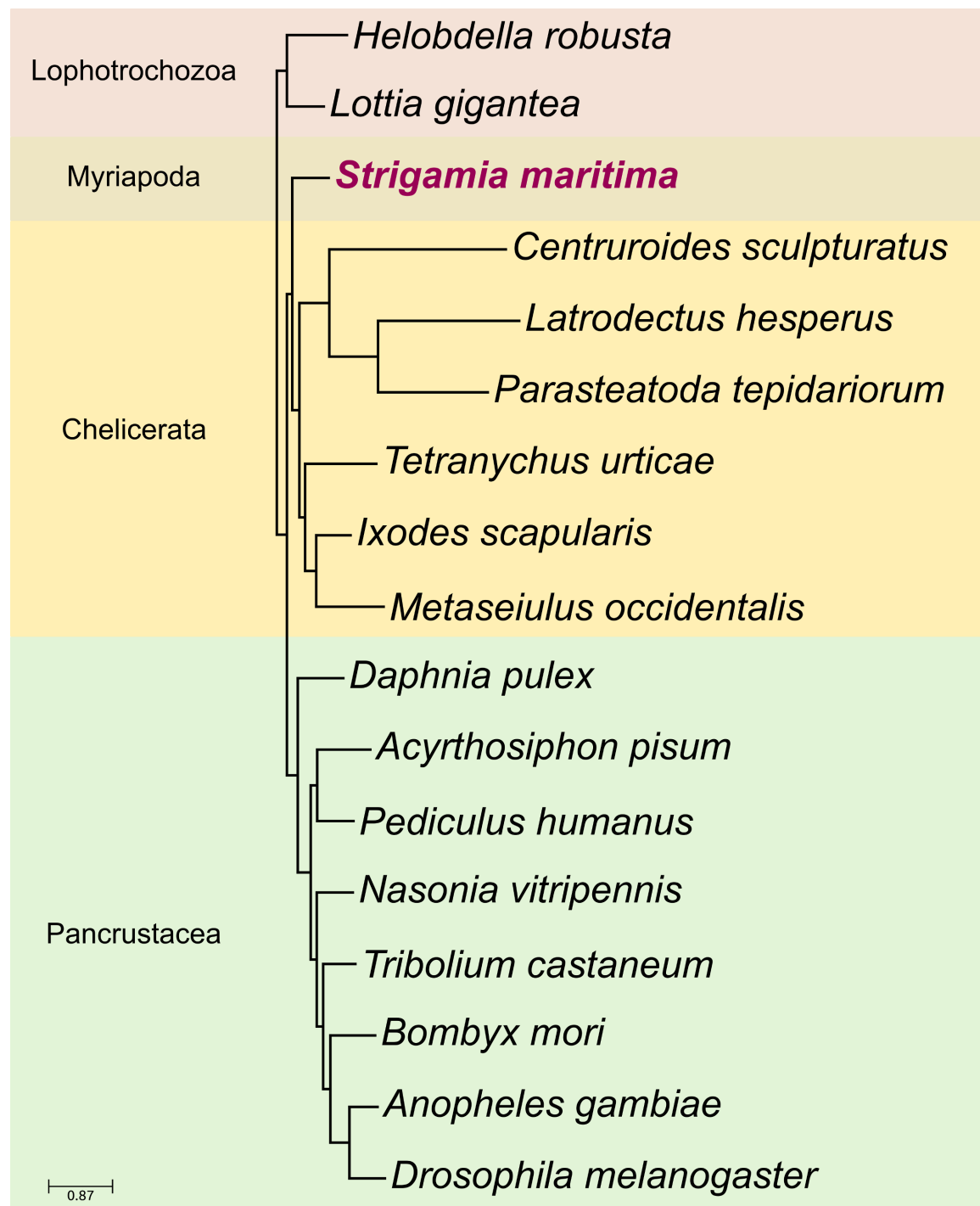

Supplement: Figure S3 — Multi-gene phylogeny for 12 species included in the phylogenomics analysis plus five additional Chelicerata species. 1,491 widespread single-copy sets of orthologue sequences were concatenated into a single alignment of 829,729 positions. Then, a maximum-likelihood tree was inferred using LG as the evolutionary model by using PhyML. (PDF) [file pbio.1002005.s003.pdf]

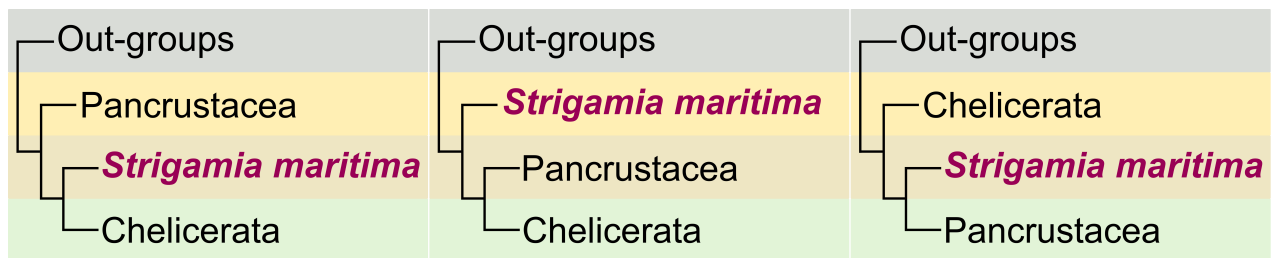

Supplement: Figure S4 — Alternative topological placements of S. maritima relative to the main arthropod groups considered in the study: Chelicerata and Pancrustacea. Internal organization of each group was initially collapsed and, therefore, optimized during maximum-likelihood reconstruction. (PDF) [file pbio.1002005.s004.pdf]

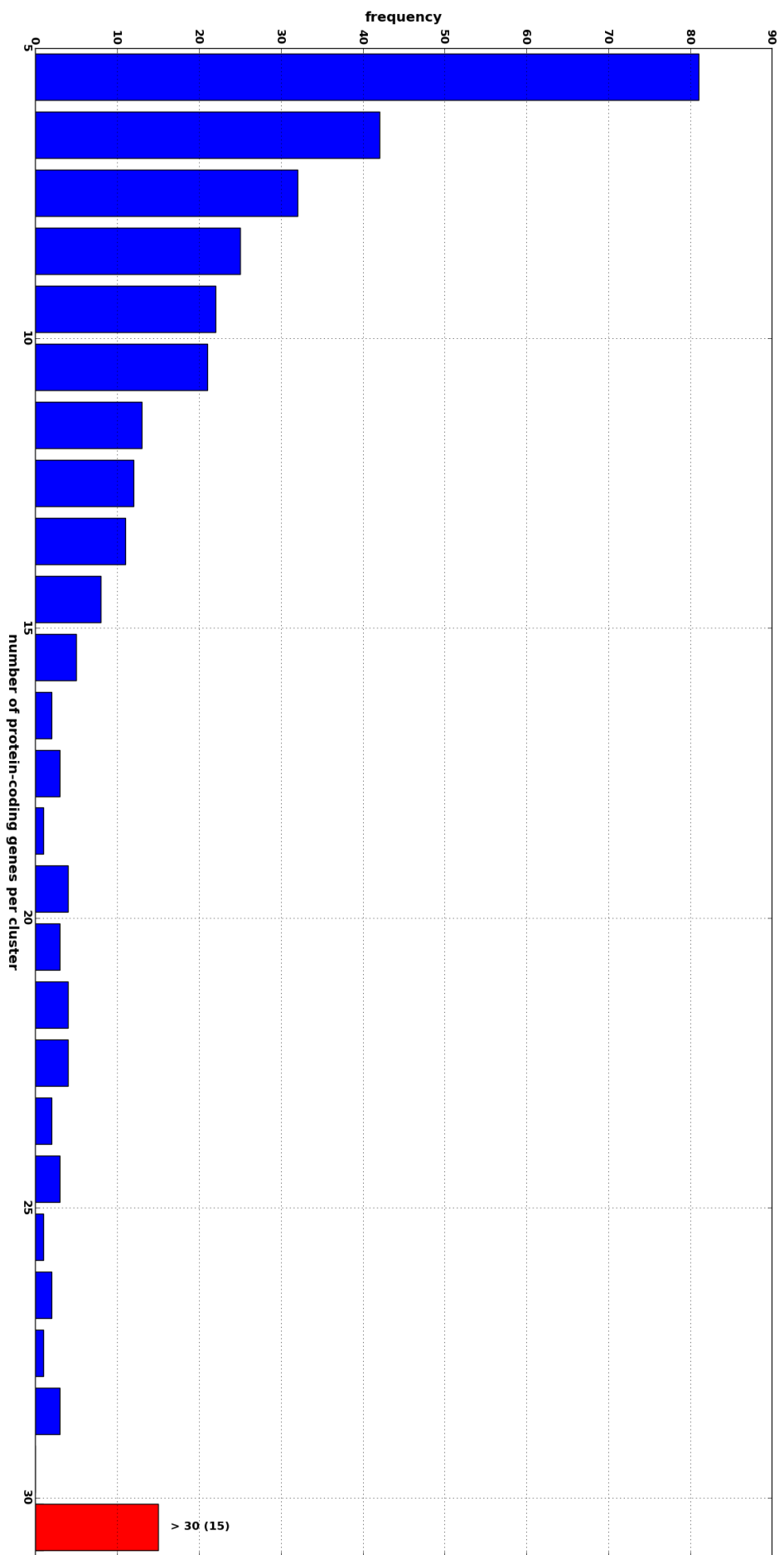

Supplement: Figure S5 — Clusters of genes specifically expanded in the centipede lineage. On the plot, only clusters grouping five or more protein-coding genes were considered. The data underlying this plot are available in File S4. (PDF) [file pbio.1002005.s005.pdf]

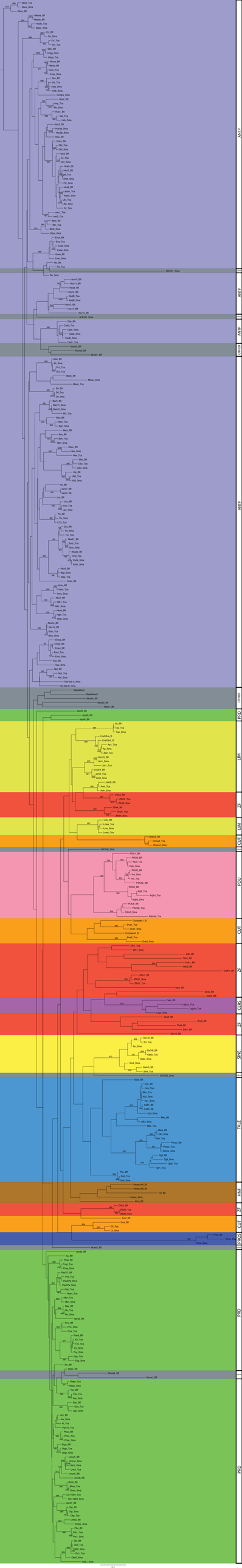

Supplement: Figure S7 — Classification of all S. maritima (Sma) homeodomains (excluding Pax2/5/8/sv) via phylogenetic analysis using T. castaneum (Tca) and B. floridae (Bfl) homeodomains. This phylogenetic analysis was constructed using neighbour-joining with a JTT distance matrix and 1,000 bootstrap replicates. Gene classes are indicated by colours. The genes coloured in grey are those genes that cannot be assigned to known classes. Further classification was performed using additional domains outside the homeodomain and by performing additional phylogenetic analysis for particular gene classes using maximum-likelihood and bayesian approaches. Pax2/5/8/sv is excluded due to the gene possessing only a partial homeobox. (PDF) [file pbio.1002005.s007.pdf]

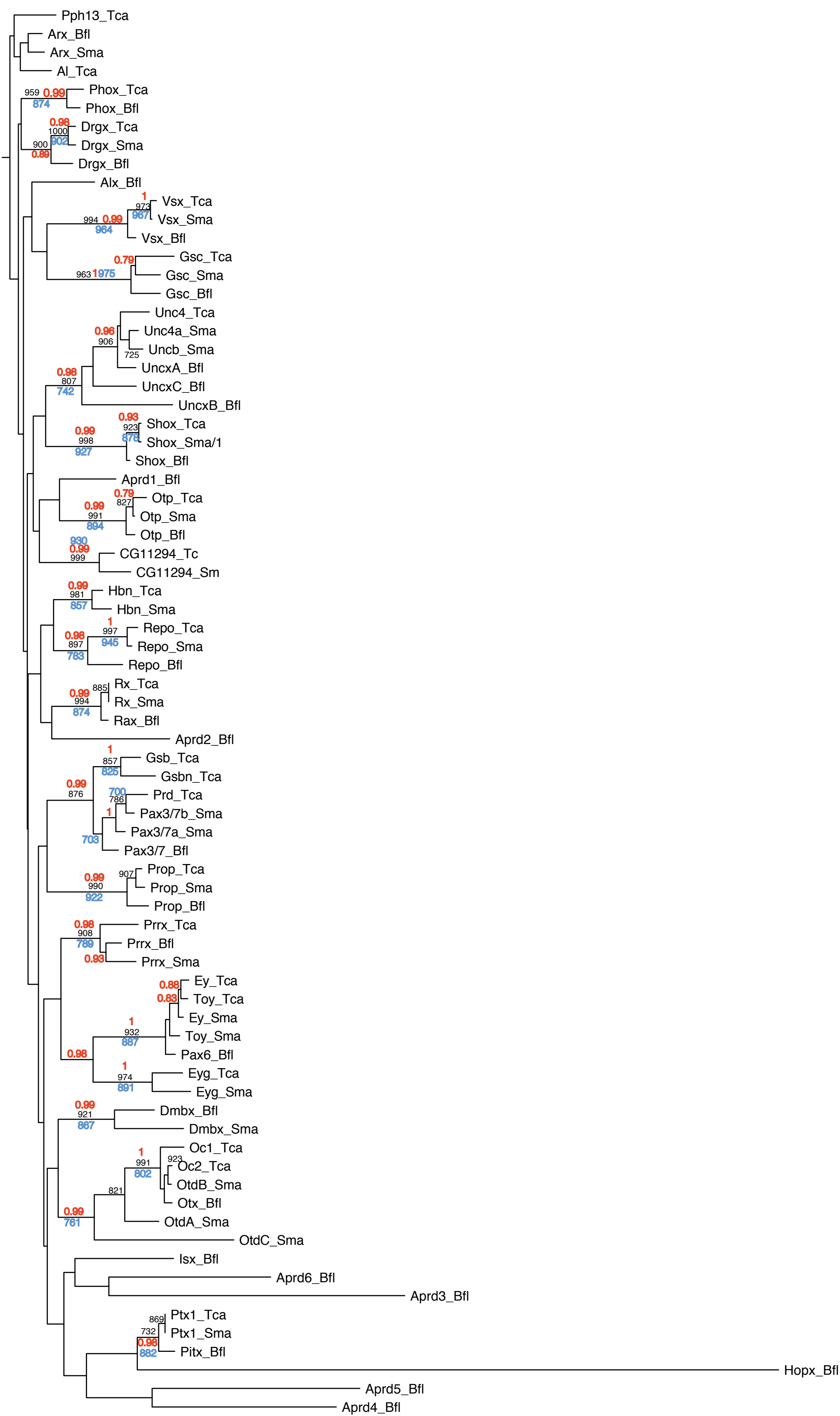

0.2

Supplement: Figure S9 — Phylogenetic analysis of PRD class homeodomains of S. maritima (Sma) using T. castaneum (Tca) and B. floridae (Bfl) for comparison. These phylogenetic analyses were constructed using neighbour-joining with a JTT distance matrix, 1,000 bootstrap replicates (support values in black). Nodes with support equal to or above 500 in the maximum-likelihood (LG+G) analysis are in blue and nodes with posterior probabilities equal to or above 0.5 (LG+G) in the Bayesian analysis are in red. (PDF) [file pbio.1002005.s009.pdf]

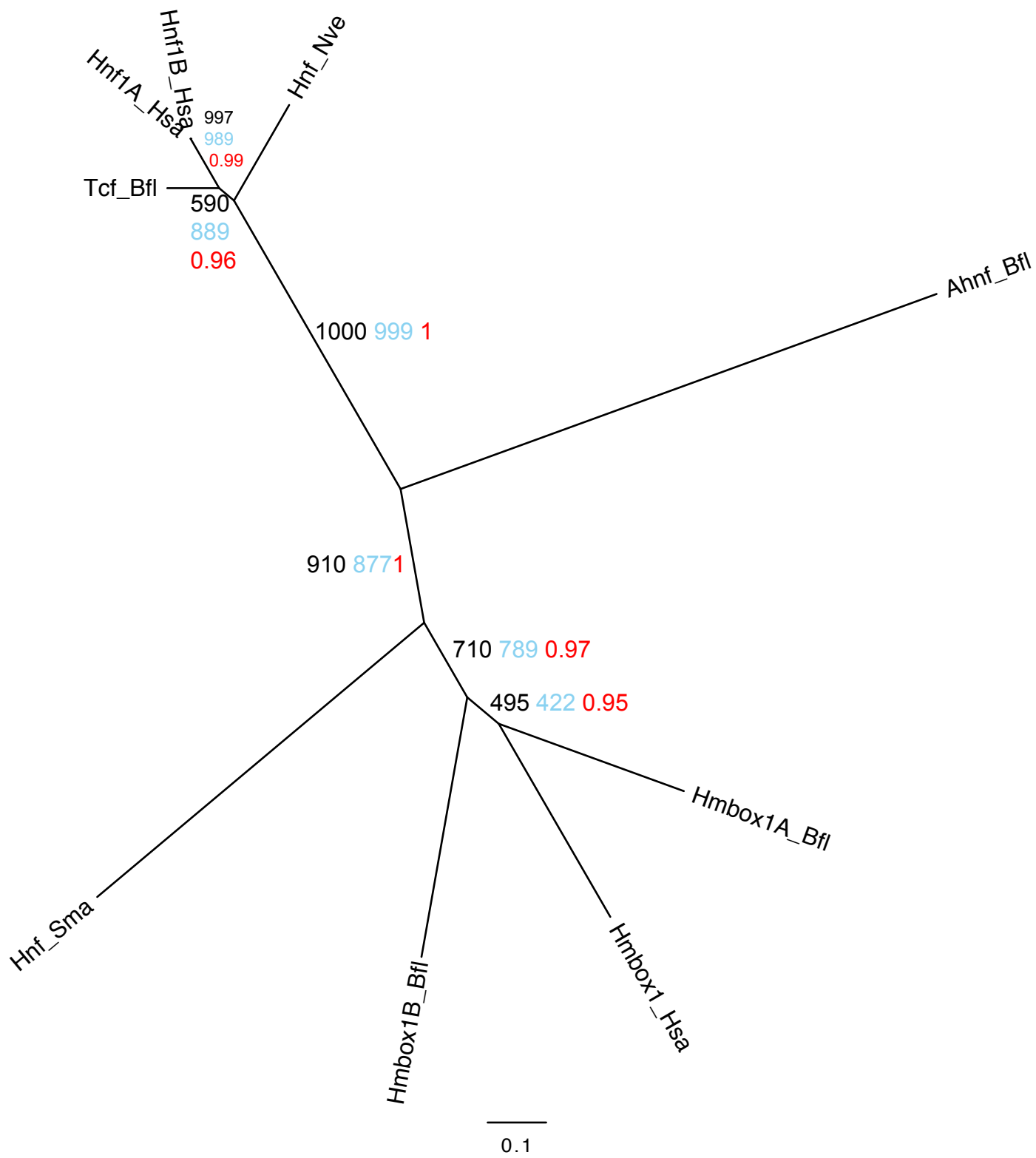

Supplement: Figure S10 — Phylogenetic analysis of HNF class homeodomains of S. maritima (Sma) using B. floridae (Bfl), human ( Homo sapiens , Hsa), and sea anemone ( N. vectensis , Nve) for comparison. These phylogenetic analyses were constructed using neighbour-joining with a JTT distance matrix, 1,000 bootstrap replicates (support values in black). Nodes with support equal to or above 500 in the maximum-likelihood (LG+G) analysis are in blue and nodes with posterior probabilities equal to or above 0.5 (LG+G) in the Bayesian analysis are in red. (PDF) [file pbio.1002005.s010.pdf]

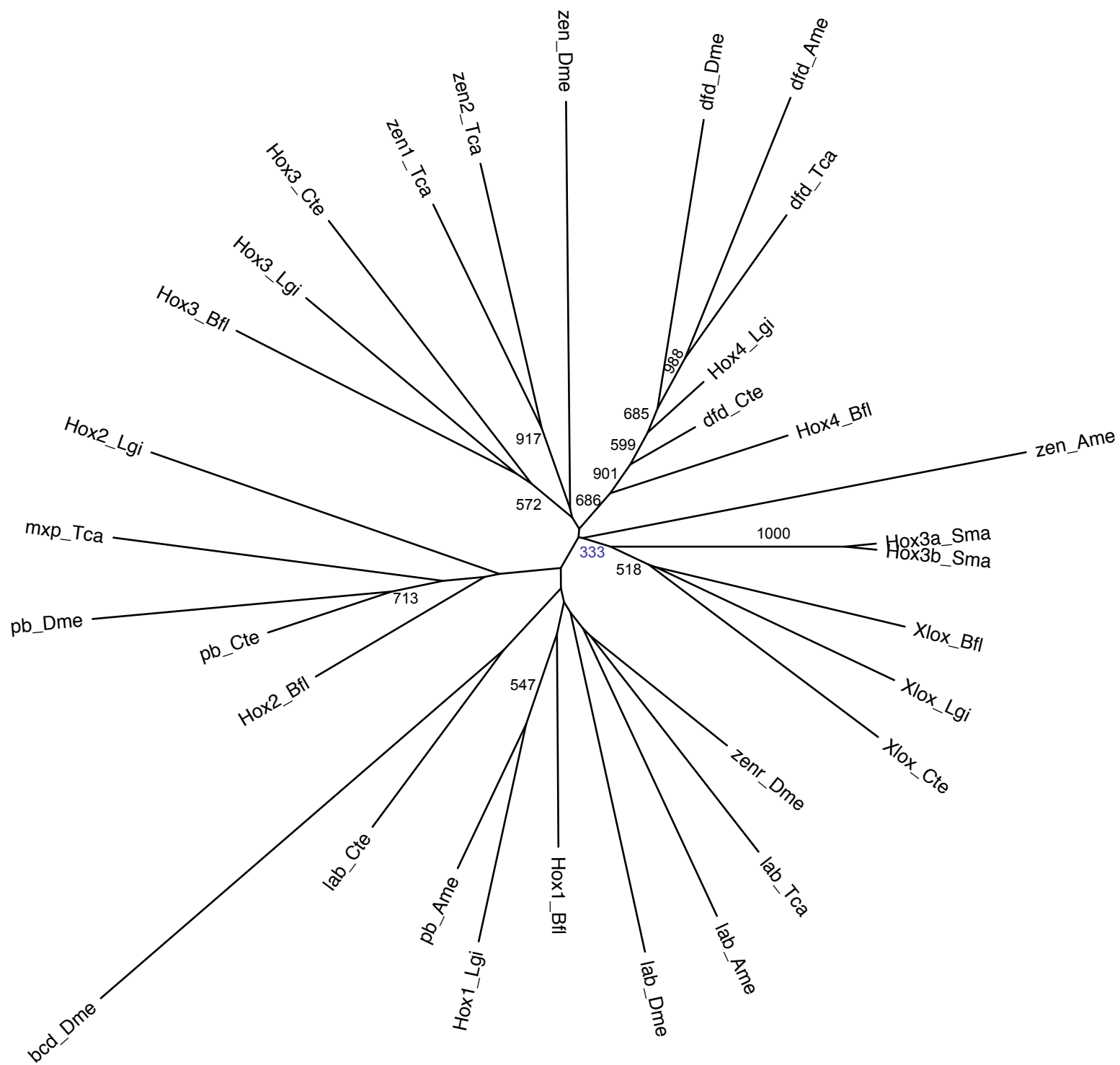

0.3

Supplement: Figure S11 — Phylogenetic analysis of Xlox/Hox3 genes of S. maritima (Sma) using a selection of Hox1, Hox2, Hox3, Hox4, and Xlox sequences. This analysis was based upon the whole coding sequence of the genes, and was constructed using neighbour-joining with a JTT distance matrix and 1,000 bootstrap replicates. The blue support value (of 333) is the node that reveals the affinity between the Xlox/Hox3 genes of S. maritima and Xlox sequences. Ame, A. mellifera; Bfl, B. floridae; Cte, Capitella teleta; Dme, D. melanogaster; Lgi, Lottia gigantea; and Tca, T. castaneum. (PDF) [file pbio.1002005.s011.pdf]

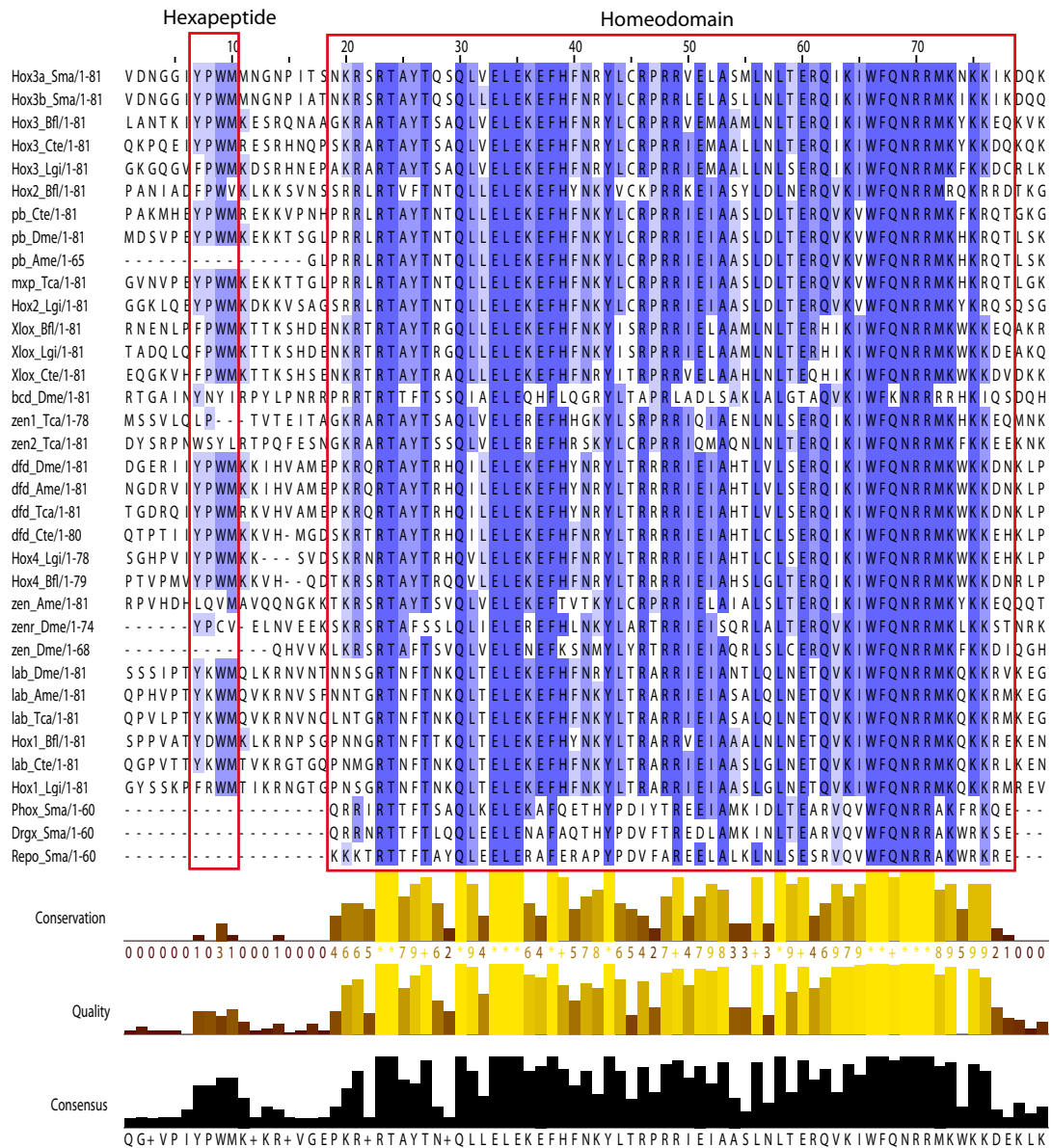

Supplement: Figure S12 — Multiple alignment of relevant residues of the Hox1, Hox2, Hox3, Hox4, and Xlox sequences of different lineages compared to S. maritima Hox3a and Hox3b sequences. Three paired class genes are included as an outgroup. The grading of purple colouring of the amino acids shows the identity level of these sequences. The red rectangles in the multiple alignment delimit the core of the hexapeptide motif and the homeodomain. This is the alignment used to construct the phylogenetic tree in Figure S13. Ame, A. mellifera; Bfl, B. floridae; Cte, Capitella teleta; Dme, D. melanogaster; Lgi, Lottia gigantea; and Tca, T. castaneum. (PDF) [file pbio.1002005.s012.pdf]

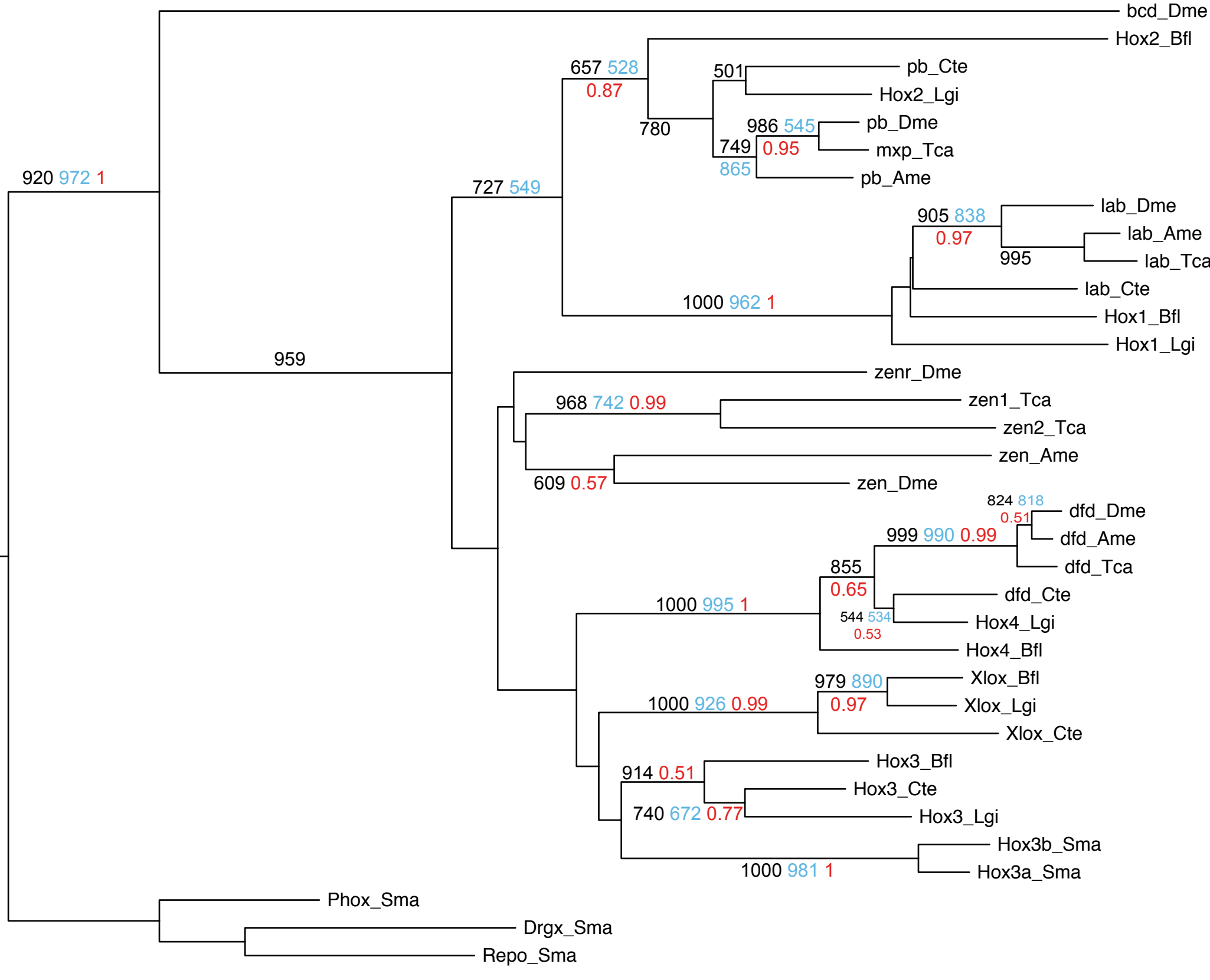

0.2

Supplement: Figure S13 — Phylogenetic analysis of S. maritima Xlox/Hox3 homeodomain and hexapeptide motifs using a selection of Hox1, Hox2, Hox3, Hox4, and Xlox sequences. This analysis used a section of the coding sequence including the hexapeptide and some flanking residues plus the homeodomain (alignment in Figure S12). Three paired class genes are included as an outgroup. This phylogeny was constructed using neighbour-joining with the JTT distance matrix and 1,000 bootstrap replicates. Maximum likelihood support values are shown in blue and Bayesian posterior probabilities in red. Ame, A. mellifera; Bfl, B. floridae; Cte, Capitella teleta; Dme, D. melanogaster; Lgi, Lottia gigantean; Tca, T. castaneum. (PDF) [file pbio.1002005.s013.pdf]

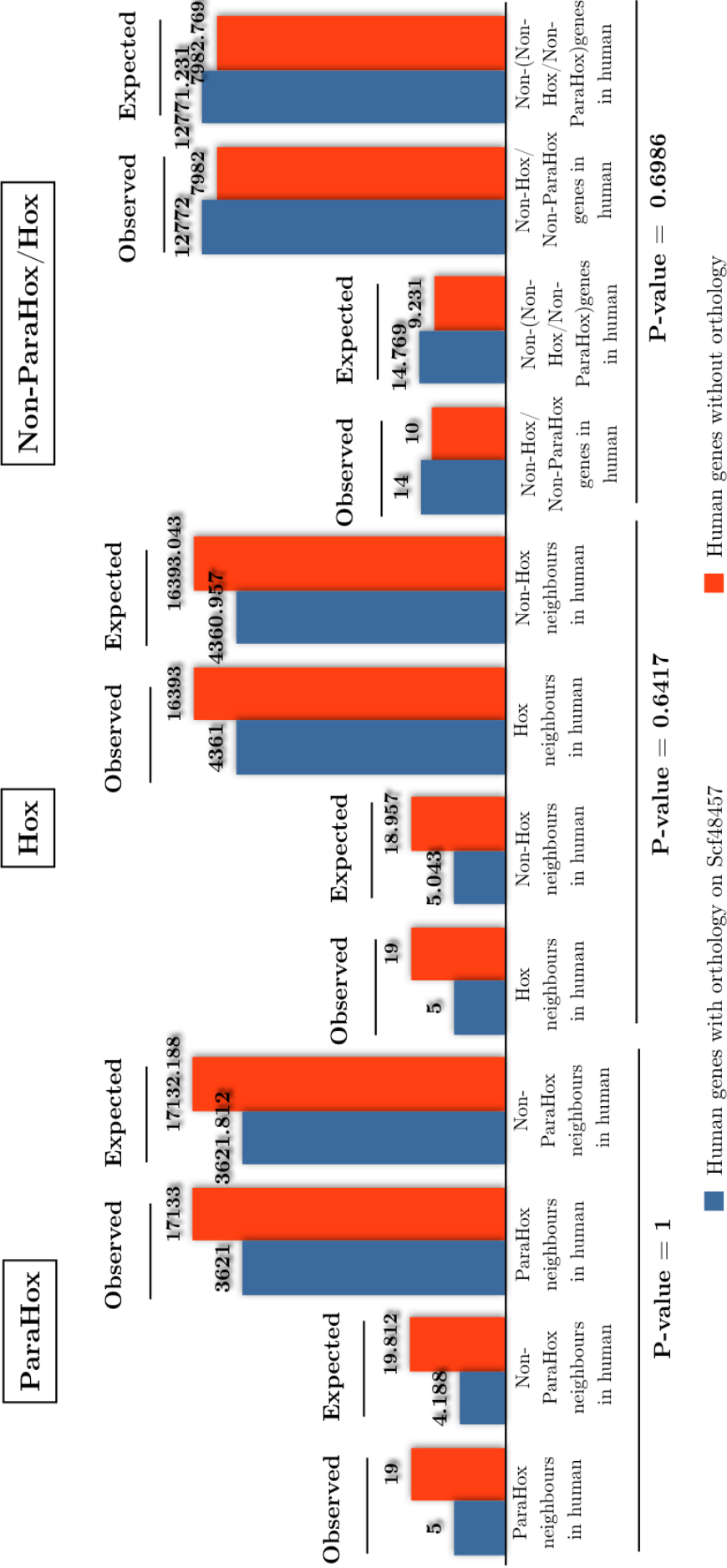

Supplement: Figure S14 — Fisher's exact test to distinguish whether S. maritima scaffold 48457 has significant synteny conservation with ParaHox or Hox chromosomes of humans. No significant Hox or ParaHox association is found. (PDF) [file pbio.1002005.s014.pdf]

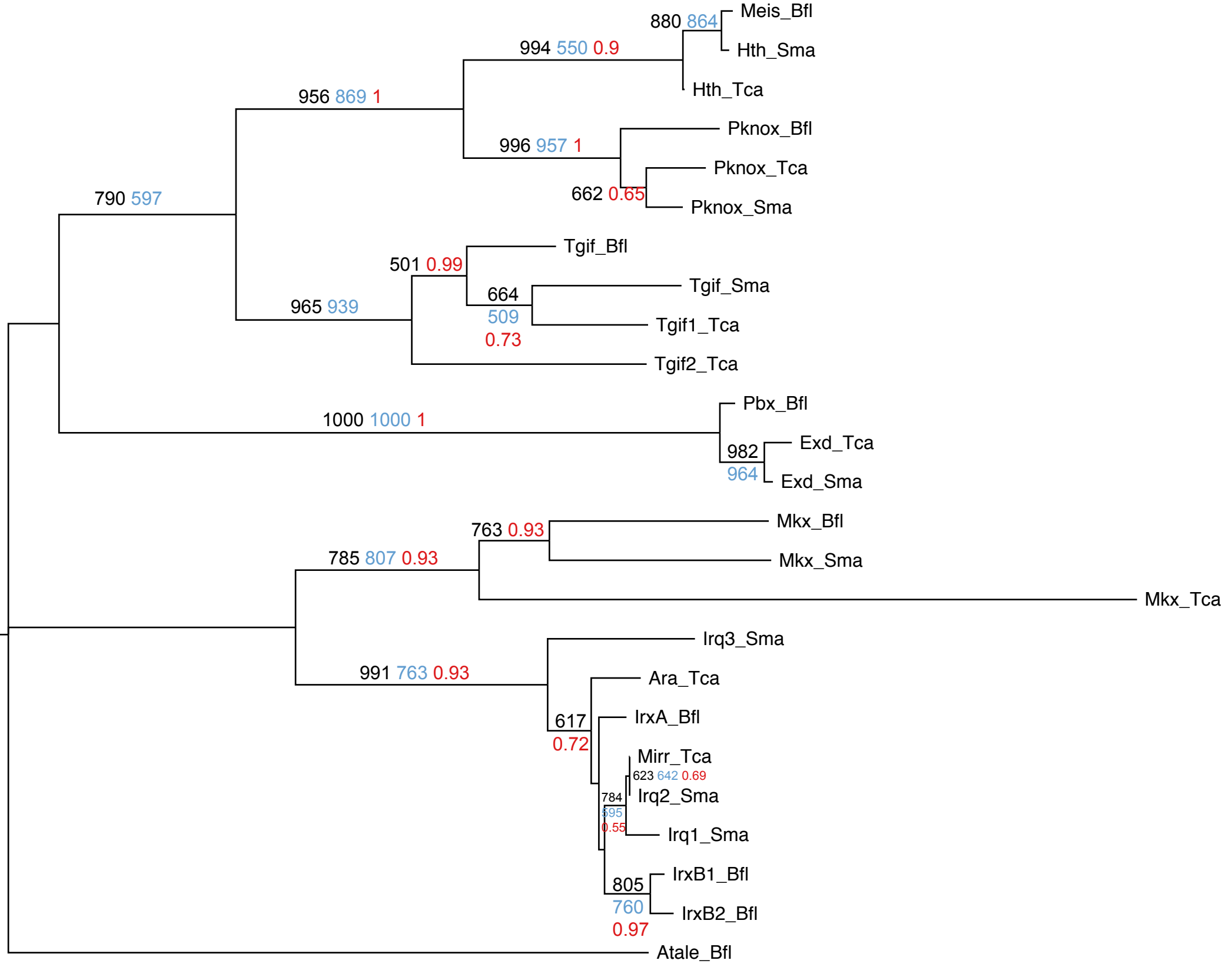

0.1

Supplement: Figure S15 — Phylogenetic analysis of TALE class homeodomains of S. maritima (Sma) using T. castaneum (Tca) and B. floridae (Bfl), including the Iroquois/Irx genes. These phylogenetic analyses were constructed using neighbour-joining with a JTT distance matrix, 1,000 bootstrap replicates (support values in black). Nodes with support equal to or above 500 in the maximum-likelihood (LG+G) analysis are in blue and nodes with posterior probabilities equal to or above 0.5 (LG+G) in the Bayesian analysis are in red. (PDF) [file pbio.1002005.s015.pdf]

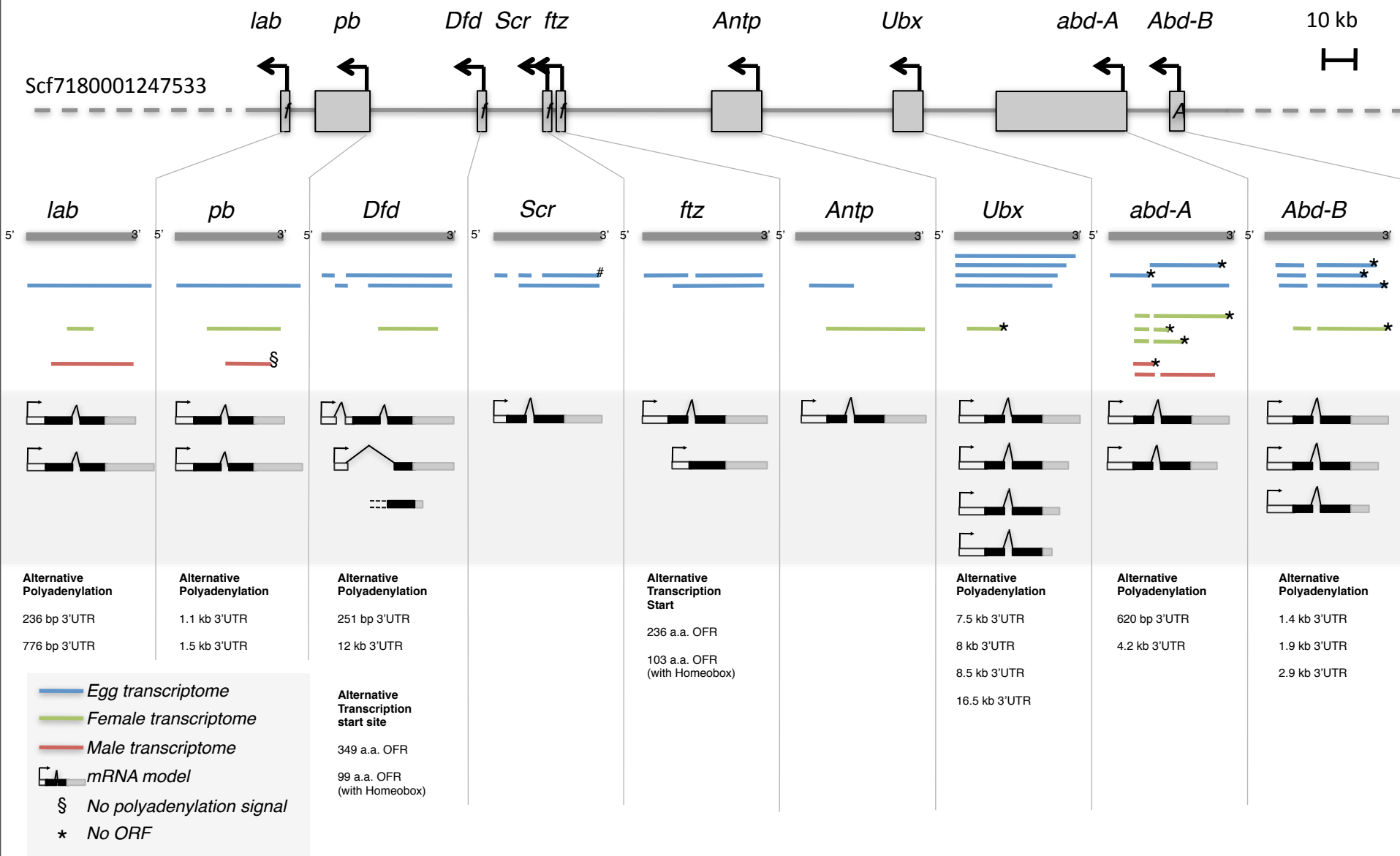

Supplement: Figure S16 — RNA processing in the Hox cluster of S. maritima . The transcriptome of S. maritima (Sm) eggs (blue), females (green), and males (red) was mapped to the Hox gene cluster (top panel; see Figure 4 in the main text) and transcript models were inferred for each gene within the cluster (shaded area) taking into account the presence of ORF and polyadenylation signals (PAS) to support the existence of RNA processing events. We note the occurrence of more than one mRNA isoform of six S. maritima Hox genes (i.e., Antp, Ubx, abd-A, lab, Dfd, pb). In all these six cases alternative polyadenylation (APA) generates mRNAs bearing distinct 3′ UTRs (alternative UTR sizes at the bottom). Alternative splicing (AS) with concomitant alternative promoter use (APU) events concern two S. maritima Hox genes Dfd and ftz (see alternative ORF sizes at the bottom). We also see that some genes such as S. maritima Ubx display high heterogeneity in 3′UTR sequences within the embryonic transcriptome (“eggs” data) suggesting the possibility that S. maritima Ubx APA might be developmentally controlled and/or display a tissue-specific pattern (see inset for further details on symbols). (PDF) [file pbio.1002005.s016.pdf]

**A**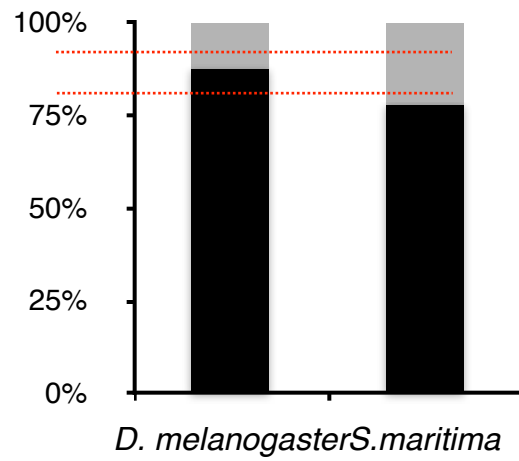**B**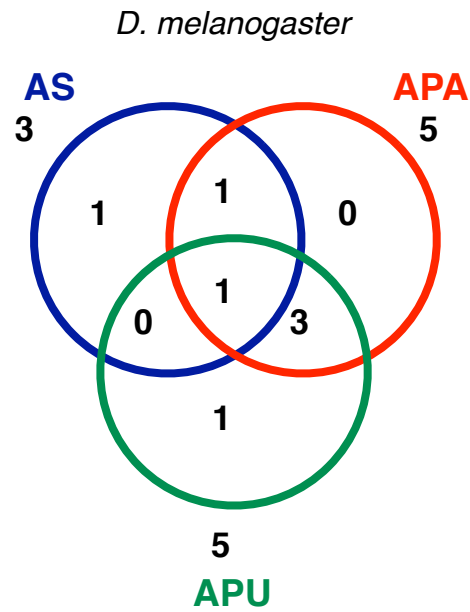**C**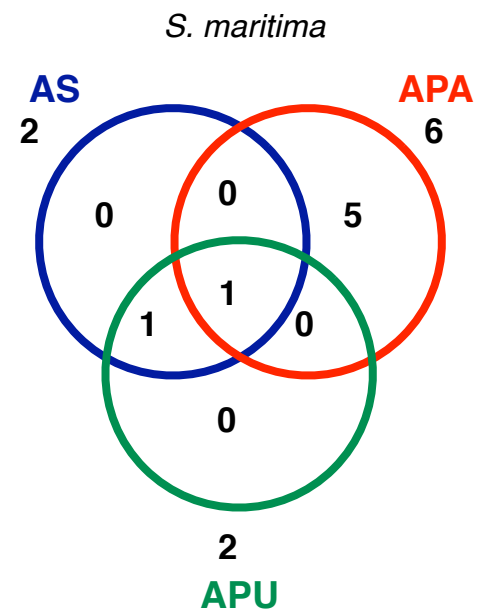

Supplement: Figure S17 — RNA processing in the S. maritima and D. melanogaster Hox clusters. (A) The incidence of alternatively processed mRNAs is comparable between S. maritima and D. melanogaster, in that over 75% of the S. maritima Hox genes undergo RNA processing of one type or another. Similarly, seven out of the eight Drosophila Hox genes produce different mRNA isoforms (FlyBase, http://flybase.org/). (B) Three D. melanogaster Hox genes undergo AS (blue) and five produce different transcripts via APA (red, FlyBase http://flybase.org/). In addition five fruit fly Hox genes form different RNA species by APU (green). (C) Classification of all alternatively processed mRNA events in the S. maritima Hox cluster based on the same categorisation as in (B). Note that patterns of AS and APA affecting S. maritima and D. melanogaster Hox genes are relatively comparable; in contrast, APU seems more prevalent in the Drosophila (five out of eight genes) than in the centipede (two out of nine genes) Hox genes. (PDF) [file pbio.1002005.s017.pdf]

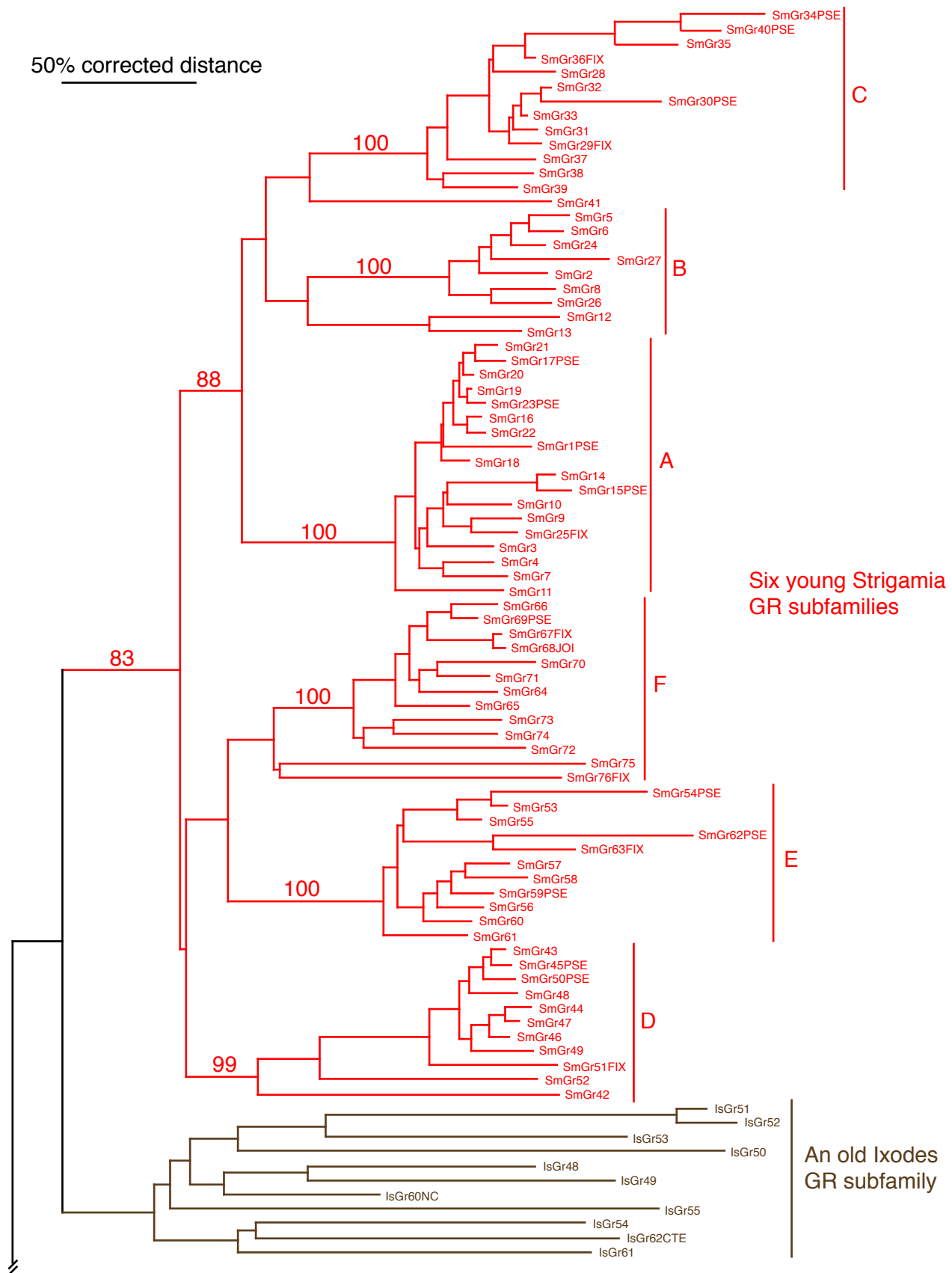

Supplement: Figure S19 — Phylogenetic tree of the S. maritima , D. pulex , I. scapularis , and representative insect GRs, part two. This is a corrected distance tree and was rooted at the midpoint in the absence of a clear outgroup, an approach that clearly indicates the distinctiveness of the centipede GRs. It is a more detailed version of Figure 5A. The S. maritima, D. pulex, I. scapularis, and representative insect gene/protein names are highlighted in red, blue, brown, and green, respectively, as are the branches leading to them to emphasize gene lineages. Bootstrap support levels in percentage of 10,000 replications of neighbour-joining with uncorrected distance is shown above major branches. Comments on major gene lineages are on the right. Suffixes after the gene/protein names are: PSE, pseudogene; FIX, sequence fixed with raw reads; JOI, gene model joined across scaffolds. Note than in Figure 5A for space reasons the IsGr47 and 59 proteins are included in the carbon dioxide and sugar receptor groupings, respectively; however, there is no bootstrap support for these branches, and no such functional assignment is claimed. Similarly, it is unlikely that the DpGr57/58 proteins are fructose receptors. (PDF) [file pbio.1002005.s019.pdf]

A

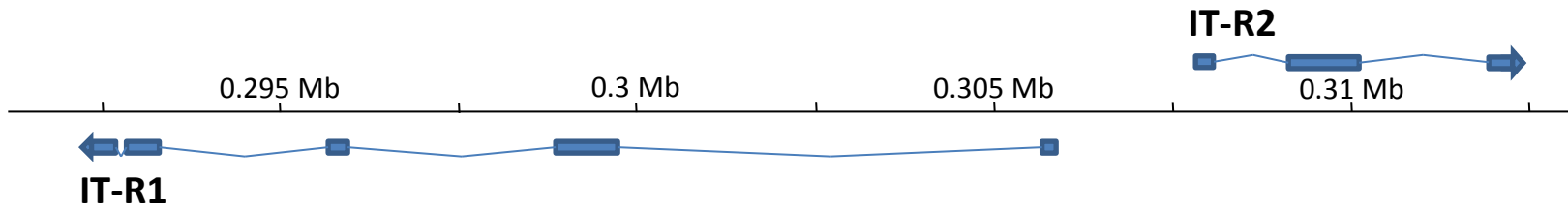

B

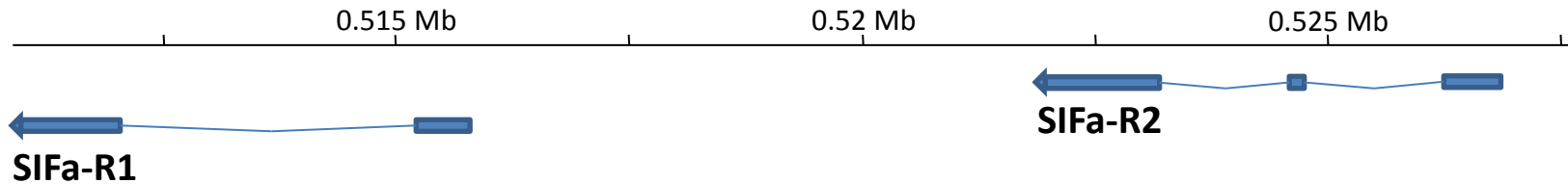

Supplement: Figure S21 — Examples of tandem duplications of neuropeptide receptor genes. Structure of the two inotocin receptor genes found head-to-head on opposite strands of scaffold JH431865 (A). Structure of the two SIFamide receptor genes found tail-to-head on the same strand of scaffold JH432116 (B). (PDF) [file pbio.1002005.s021.pdf]

# Synthesis

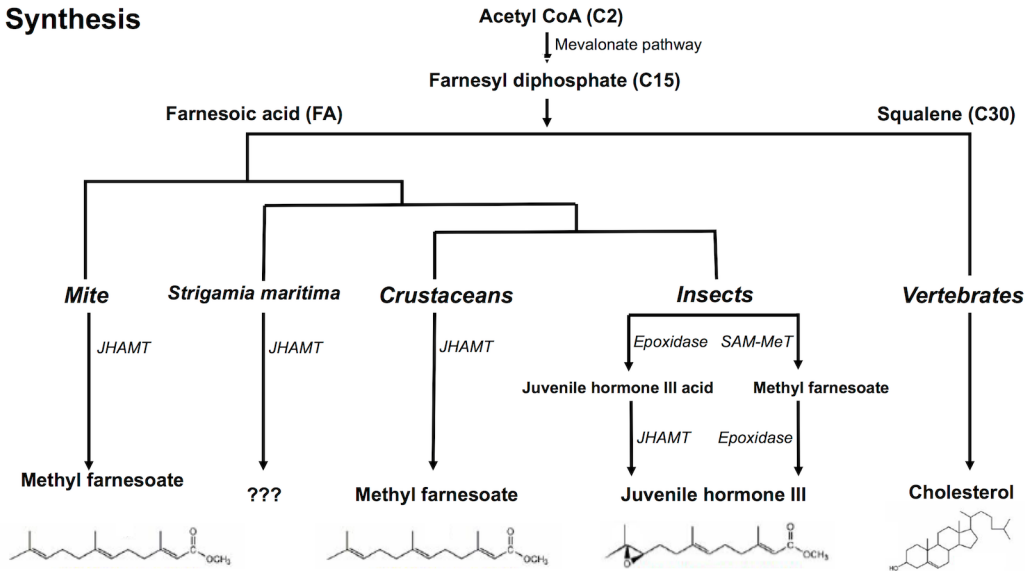

# Degradation

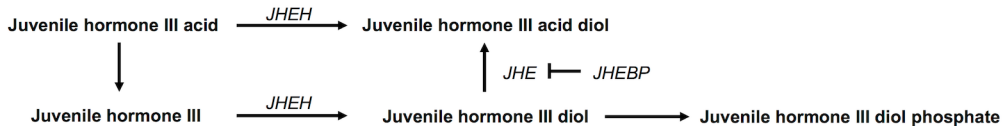

Supplement: Figure S22 — Schematic diagram showing sesquiterpenoids/juvenoids synthesis (upper) and degradation (lower) pathways in arthropods. Molecules/hormones in synthesis are shown in bold, enzymes are shown in italics, and species/clades are shown in bold italics. (PDF) [file pbio.1002005.s022.pdf]

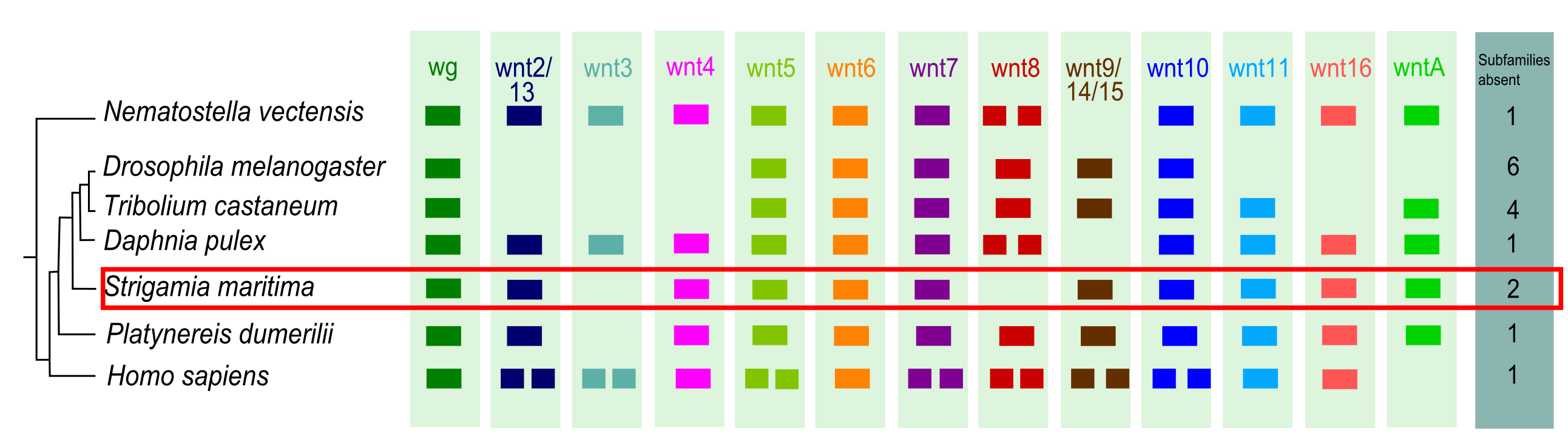

Supplement: Figure S24 — Range of Wnt genes present in S. maritima . Wnt genes present and number of Wnt subfamilies absent in S. maritima in comparison with other arthropods and three non-arthropod outgroups. (TIF) [file pbio.1002005.s024.tif]

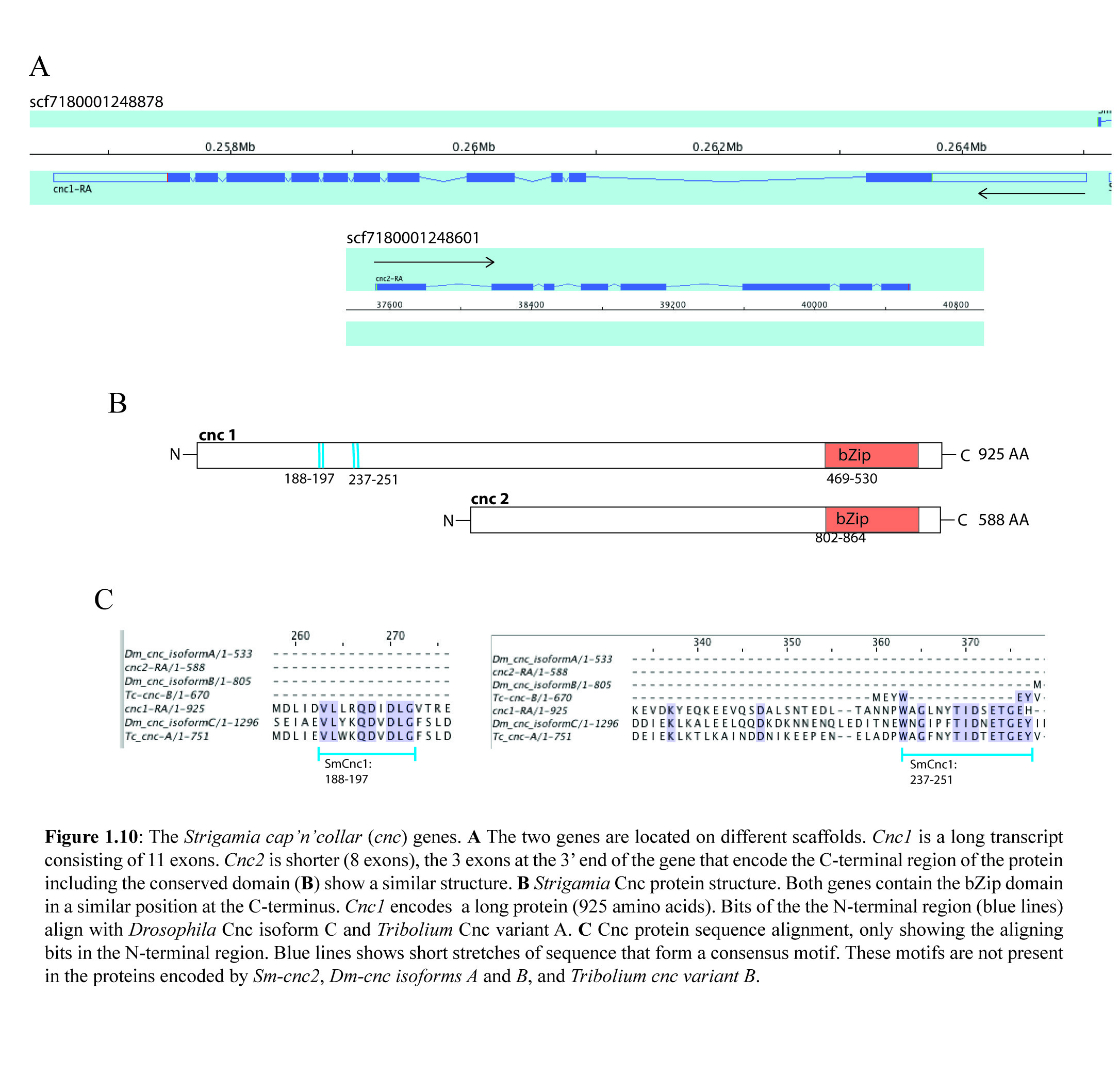

Supplement: Figure S27 — Cap ‘n’ collar (cnc) genes. (A) The two genes are located on different scaffolds. Cnc1 is a long transcript consisting of 11 exons. Cnc2 is shorter (eight exons), the three exons at the 3′ end of the gene that encode the C-terminal region of the protein including the conserved domain (B) show a similar structure. (B) S. maritima Cnc protein structure. Both proteins contain the bZip domain in a similar position at the C-terminus. Cnc1 encodes a long protein (925 amino acids). Bits of the N-terminal region (blue lines) align with D. melanogaster Cnc isoform C and T. castaneum Cnc variant A. (C) Cnc protein sequence alignment, only showing the aligning bits in the N-terminal region. Blue lines show short stretches of sequence that form a consensus motif. These motifs are not present in the proteins encoded by Sm-cnc2, Dm-cnc isoforms A and B, and T. castaneum cnc variant B. (JPG) [file pbio.1002005.s027.jpg]

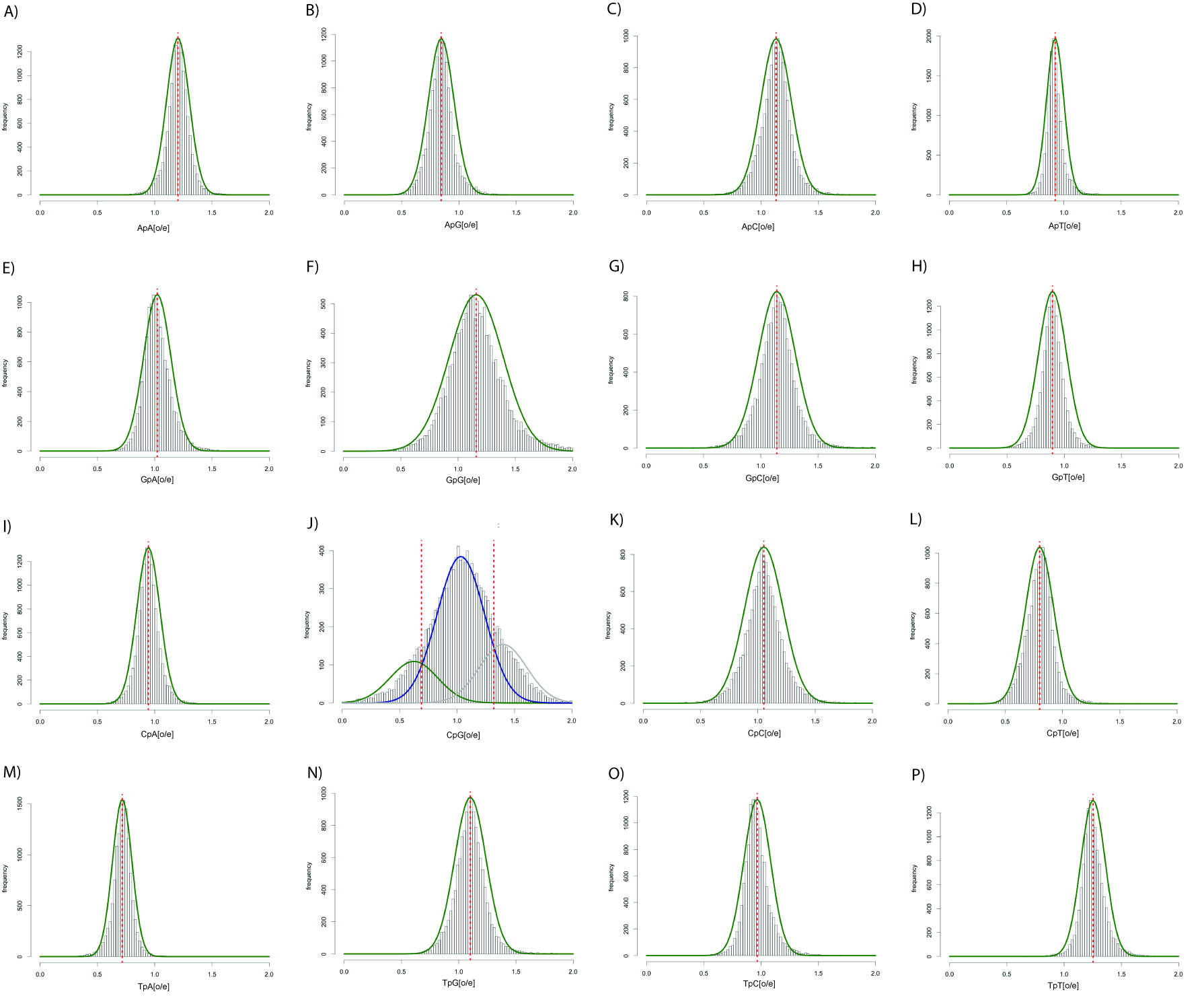

Supplement: Figure S28 — Frequency histograms of observed versus expected dinucleotide content in S. maritima gene bodies. (A–P) The y-axis depicts the number of genes with the specific dinucleotide[o/e] values given on the x-axis. The distribution of all dinucleotide pairs, with the exception of CpG, is best described as a unimodal distribution. The distribution of CpG dinucleotides is best described as a trimodal distribution, with “high” and “low” CpG[o/e] classes. The data underlying this figure are available in File S5. (TIF) [file pbio.1002005.s028.tif]

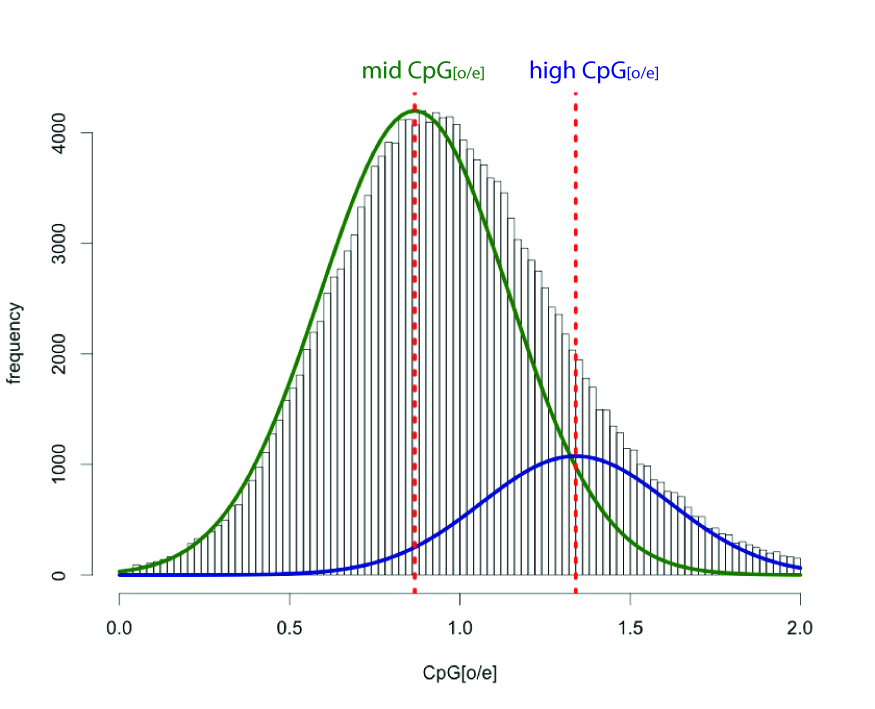

Supplement: Figure S29 — Frequency histogram of CpG[o/e] observed in 1,000 bp windows of the S. maritima genome. The y-axis depicts the number of genes with the specific CpG[o/e] values given on the x-axis. The distribution of CpG[o/e] in S. maritima genome is a bimodal distribution, with a high CpG[o/e] peak observed similar to that observed in the gene bodies (Figure 9). The data underlying this figure are available in File S6. (TIF) [file pbio.1002005.s029.tif]

A. *Drosophila melanogaster*

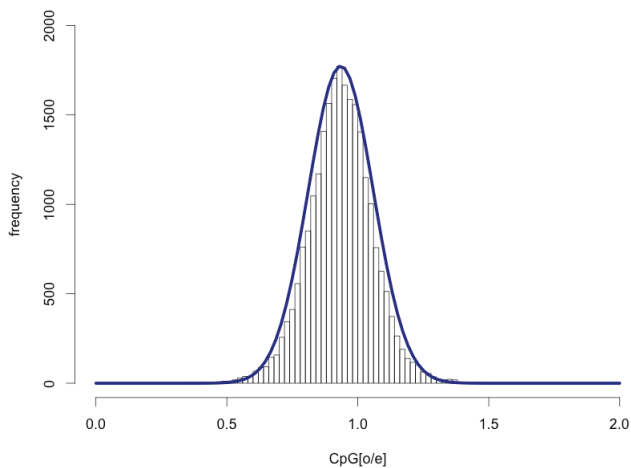

B. *Apis mellifera*

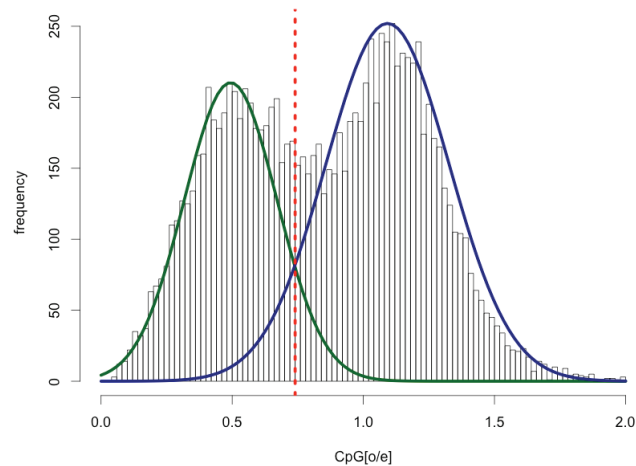

C. *Tetranychus urticae*

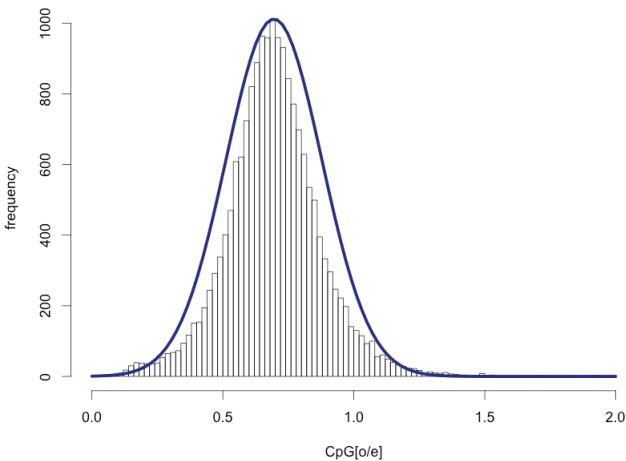

D. *Strigamia maritima*

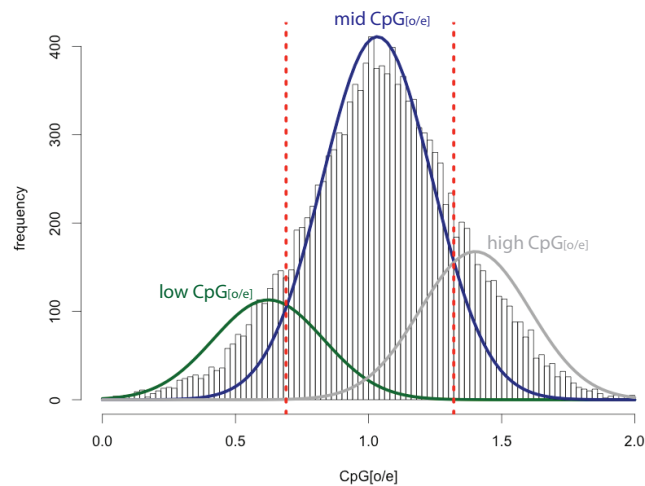

Supplement: Figure S30 — Contrasting patterns of DNA methylation, as measured by over- and underrepresentation of CpG dinucleotides in coding regions (CpG(o/e)), within arthropod species. In all graphs the y-axis depicts the number of genes with the specific CpG(o/e) values given on the x-axis. (A) D. melanogaster coding regions show a unimodal peak reflective of the lack of DNA methylation in this species. (B) A. mellifera shows a bimodal peak consisting of genes with a lower than expected CpG(o/e) (green distribution) and a higher than expected CpG(o/e) (blue distribution). The presence of a bimodal distribution in this species is consistent with depletion of CpG dinculeotides in the coding regions of genes over evolutionary time as a result of DNA methylation. (C) A single unimodal peak is also observed for Tetranychus urticae, a species that has very low levels of DNA methylation. (D) The S. maritima distribution is best explained as a mixture of three distinct distributions that we have deemed “low” (green distribution), “medium” (blue distribution), and “high” (grey distribution). The genes within the low distribution likely contain genes that are historically methylated, whilst the “high” distribution can be explained by regions of the genome that are comparatively CpG-rich (as determined by the analysis of the S. maritima genome, Figure S29). The data underlying this figure are available in File S7. (PDF) [file pbio.1002005.s030.pdf]

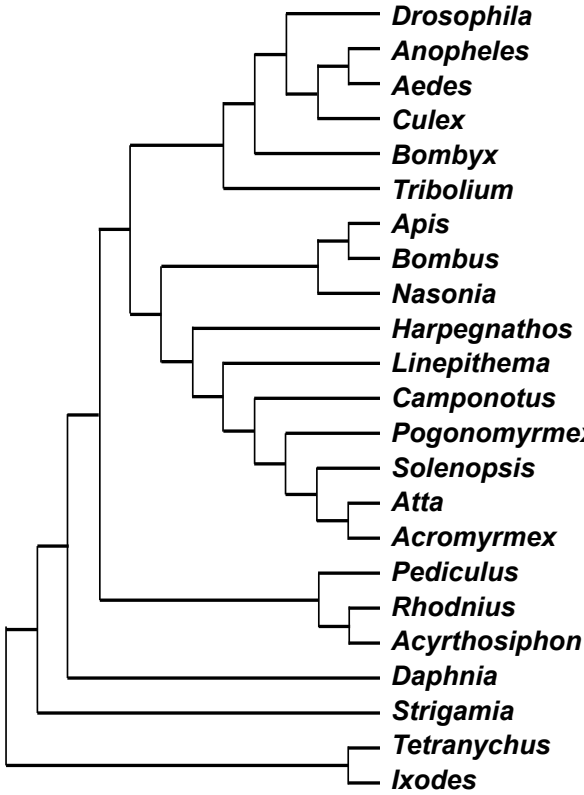[illegible]

Supplement: Figure S33 — Phylogenomic inventory of meiotic genes in arthropods. Red genes are specific to meiosis in model species in which functional data are available. “+” and “−” indicate the presence and absence of orthologues, respectively. Numbers indicate copy number of duplicated genes. (PDF) [file pbio.1002005.s033.pdf]
