# Supplementary material for: The First Myriapod Genome Sequence Reveals Conservative Arthropod Gene Content and Genome Organisation in the Centipede Strigamia maritima
Source: PLoS Biol. 2014 Nov 25;12(11):e1002005. doi: 10.1371/journal.pbio.1002005 (PMC4244043; doi:10.1371/journal.pbio.1002005)
Supplement: Figure S6 — Mitochondrial gene organisation. Shaded regions represent differences from the ground pattern. Gene translocations in Myriapoda have been noted in Scutigerella causeyae (Myriapoda: Symphyla) [49]. The previous example of the small conserved region trnaF-nad5-H-nad4-nad4L on the minus strand between Limulus, Lithobius, and Strigamia is not a conserved feature in all Chilopoda, because Scutigera colepotrata have an interruption between nad5 and H-nad4 with elements on the minus and plus strands accompanied by a translocation of nad4L to a position immediately preceding nad5. (PDF) [file pbio.1002005.s006.pdf]

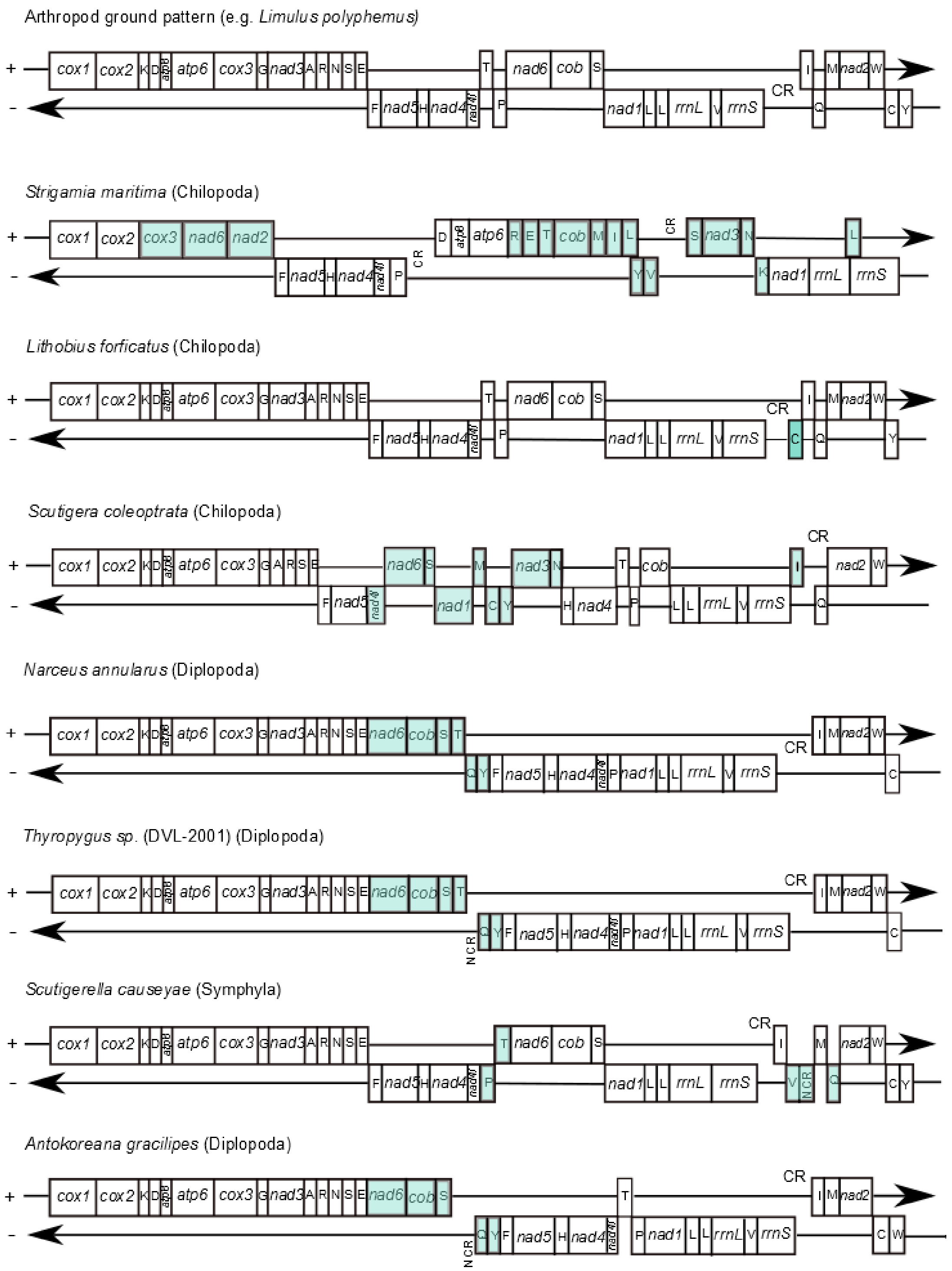
Arthropod ground pattern (e.g. Limulus polyphemus)
Strigamia maritima (Chilopoda)
Lithobius forficatus (Chilopoda)
Scutigera coleoptrata (Chilopoda)
Narceus annularus (Diplopoda)
Thyropygus sp. (DVL-2001) (Diplopoda)
Scutigerella causeyae (Symphyla)
Antokoreana gracilipes (Diplopoda)
