# Supplementary material for: The First Myriapod Genome Sequence Reveals Conservative Arthropod Gene Content and Genome Organisation in the Centipede Strigamia maritima
Source: PLoS Biol. 2014 Nov 25;12(11):e1002005. doi: 10.1371/journal.pbio.1002005 (PMC4244043; doi:10.1371/journal.pbio.1002005)
Supplement: Figure S18 — Phylogenetic tree of the S. maritima , D. pulex , I. scapularis , and representative insect GRs, part one. This is a corrected distance tree and was rooted at the midpoint in the absence of a clear outgroup, an approach that clearly indicates the distinctiveness of the centipede GRs. It is a more detailed version of Figure 5A. The S. maritima, D. pulex, I. scapularis, and representative insect gene/protein names are highlighted in red, blue, brown, and green, respectively, as are the branches leading to them to emphasize gene lineages. Bootstrap support levels in percentage of 10,000 replications of neighbour-joining with uncorrected distance is shown above major branches. Comments on major gene lineages are on the right. Suffixes after the gene/protein names are: PSE, pseudogene; FIX, sequence fixed with raw reads; JOI, gene model joined across scaffolds. Note than in Figure 5A for space reasons the IsGr47 and 59 proteins are included in the carbon dioxide and sugar receptor groupings, respectively; however, there is no bootstrap support for these branches, and no such functional assignment is claimed. Similarly, it is unlikely that the DpGr57/58 proteins are fructose receptors. (PDF) [file pbio.1002005.s018.pdf]

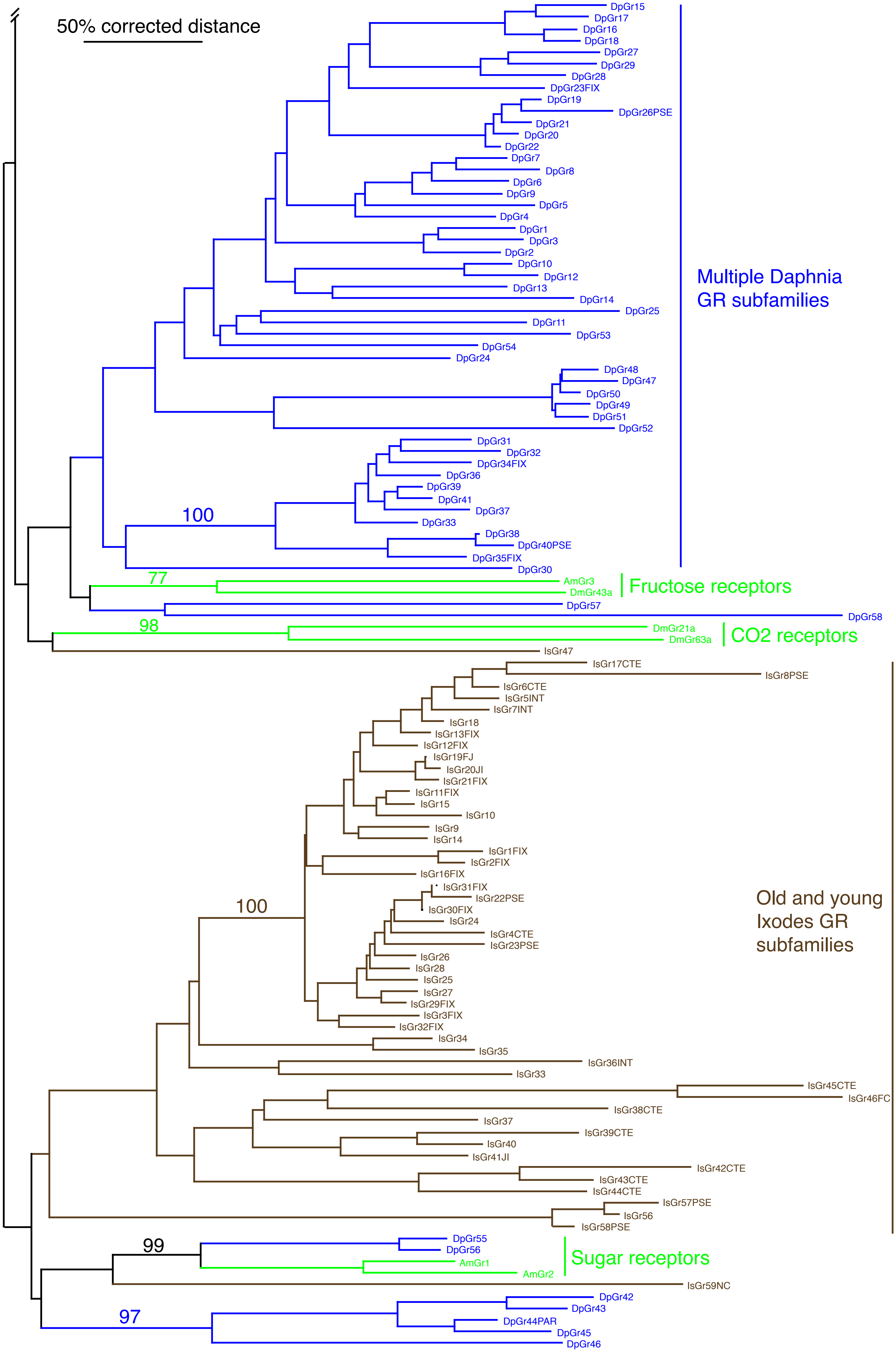

50% corrected distance
DpGr15
DpGr17
DpGr16
DpGr18
DpGr27
DpGr29
DpGr28
DpGr23FIX
DpGr19
DpGr26PSE
DpGr21
DpGr20
DpGr22
DpGr7
DpGr8
DpGr6
DpGr9
DpGr5
DpGr4
DpGr1
DpGr3
DpGr2
DpGr10
DpGr12
DpGr13
DpGr14
DpGr25
DpGr11
DpGr53
DpGr54
DpGr24
DpGr48
DpGr47
DpGr50
DpGr49
DpGr51
DpGr52
DpGr31
DpGr32
DpGr34FIX
DpGr36
DpGr39
DpGr41
DpGr37
DpGr33
100
DpGr38
DpGr40PSE
DpGr35FIX
DpGr30
Multiple Daphnia GR subfamilies
77
AmGr3
DmGr43a
Fructose receptors
DpGr57
DpGr58
98
DmGr21a
DmGr63a
CO2 receptors
IsGr47
IsGr17CTE
IsGr8PSE
IsGr6CTE
IsGr5INT
IsGr7INT
IsGr18
IsGr13FIX
IsGr12FIX
IsGr19FJ
IsGr20JI
IsGr21FIX
IsGr11FIX
IsGr15
IsGr10
IsGr9
IsGr14
IsGr1FIX
IsGr2FIX
IsGr16FIX
IsGr31FIX
IsGr22PSE
IsGr30FIX
IsGr24
IsGr4CTE
IsGr23PSE
IsGr26
IsGr28
IsGr25
IsGr27
IsGr29FIX
IsGr3FIX
IsGr32FIX
IsGr34
IsGr35
100
IsGr36INT
IsGr33
IsGr45CTE
IsGr46FC
IsGr38CTE
IsGr37
IsGr39CTE
IsGr40
IsGr41JI
IsGr42CTE
IsGr43CTE
IsGr44CTE
IsGr57PSE
IsGr56
IsGr58PSE
Old and young Ixodes GR subfamilies
99
DpGr55
DpGr56
AmGr1
AmGr2
Sugar receptors
IsGr59NC
97
DpGr42
DpGr43
DpGr44PAR
DpGr45
DpGr46
