# Supplementary material for: The First Myriapod Genome Sequence Reveals Conservative Arthropod Gene Content and Genome Organisation in the Centipede Strigamia maritima
Source: PLoS Biol. 2014 Nov 25;12(11):e1002005. doi: 10.1371/journal.pbio.1002005 (PMC4244043; doi:10.1371/journal.pbio.1002005)
Supplement: Figure S20 — Neuropeptide precursor sequences identified in the S. maritima genome. The putative signal peptides (predicted by SignalP) are underlined, the putative active neuropeptides or protein hormones (based on similarity to neuropeptides or protein hormones identified in other invertebrates) are marked in yellow. Green indicates putative basic cleavage sites flanking the putative neuropeptides. Glycines used for amidation are shown in blue, cysteines proposed to form cysteine bridges are shown in red. Dots indicate missing N- or C-termini. (DOCX) [file pbio.1002005.s020.docx]

>Smar-ACP
MKWIAVYLLLTIIVLTIVAPVEGQVTFSRDWTPAGKRGMDCGFVKTKLLRDIAVLLQVKYHFAELCMLTLAWLMLDGGQFTEILWTSCDGAFP

>Smar-AKH
MTKFTWLSMTLLVLMVFITVDVNGQINFSPGWGQGKRSLSDDKPVNGYSDCSETMIEVYRLLK

>Smar-Allatostatin A
MTFAVTWWCLLLTAPTLLMSEYIYDISSESDSNDQEKRGLNTPWKLPEGYVYLRKVDPSTGEYQIGKKDNQYRRRFSFGLGKRFHSDVLLENNEDEVGKRGSQRNRHFGLGKRQPVDYLQGRIGRYNMGLGKRSVDSREAEEVAIEEMKRGASKFNFGLGKRTKPYSFGLGKRWDDRGVEENLIEEYKRAKTYGFGLGKRDEEEMEEEKKNRPYQFGLGKRDRSYSFGLGKRMEEEKKT

>Smar-Allatostatin B
MLLSWTSSVTIVLVIASVLAASASEDKRAWSDLNGAWGKRNWDQLRGVWGKRGAGQLPNSVWGKREDAPSDWNAFRGSWGKRNNWNKLQGVWGKRDSSDWNKLQGLWGKRASWTHST

>Smar-Allatostatin C
MASSGKFCILIFALVLTLSHVTSKSIGEHEHPNFNTDLSLVDDDGSMDTALINYLFARQMIKRLQSSMDVTDLQRKRSYWKQCAFNAVSCFGKK

>Smar-Allatostatin CC
MYSLILVFCVCMLTLPYVSCQIEMNSLKNMAKTFHLSSSDSNYFQHPVKRSTMLLDRLVTALQKAFKQETQEVTGMELQRRRPNGRVYWRCYFNAVSCFRRKK

>Smar-Allatotropin
MKPVCLVILLFGLLVSATSSSTDEPANRVRQTRGFKNSALATARGFGKRTMLNDLVDSTDRAIMSNEQLADLMSRNPQFAQQILTKFVDTDGDGVLSFRE

>Smar-Bursicon alpha
MVTLVFMVAAMMCIFSSRLATADECHITPVIHVLKYPGCNQKPIPSFACQGRCTSYVSGSKMWQMERSCMCCQEMGVREANVTLHCPHARPGEPKFRKVTTRAPVDCMCRPCTSVEKHLVQPQESAPWLSDPNFNDAILSV

>Smar-Bursicon beta
MTNTWSAFAFFTIITFVLFITTKSLRAFPESTCETLPSFIHIIKEEYDSRTKLVRTCEGDVAVNKCEGTCTSQMQPSVTTSTGFLKECYCCRESYLQEREVILQRCFNFDGETLAGDMSLMKIRLKEPAECQCYKCGE

>Smar-Corazonin
MGFQKTKLLLIVASILVFIICTSGQTFQYSKGWEPGRKRAVDRSYQVRDWDAGRKRENIRGLDSSTAWILGLKRAAFN

>Smar-CCAP
MQYVTIHGFVTLLLIVFSCTIGCAAQKRRPFCNAFTGCGRKRSELPASNVNDLETDLLLDKLSHQILGLVHVLEALHMRIETSRQNQQPLPMIETDSRIPNYILDRKRRSTKI

>Smar-CCHamide 1-1
MHGCRCNTALFILLVLSLLVSSATGRRGCLNYGHSCLGAHGKRSSNRQAMRRDIANFLPLHKTAAEYNTISEENLLRDESDTRKWNDKWRDMVISALEKDD

>Smar-CCHamide 1-2
MSTLKFIFALLTLAVYVYQVQGLRGCTNYGHSCFGAHGKRTPKNDEKDQTSFLDSTENTKHVNQITTNDVPGVKNGLNSAFLRKWMTALRQSGNDEILQ

>Smar-DH31
...DKRNLDLGFSRGFSGSQAAKHLMGLAAANFAGGPGRRKRSEE

>Smar-DH44 (CRF-like)
MLLRLFSFIVCVCAIACVGARSLSRLQECTDCTLVPADDNRYSIQDDFNNKGFILKARRIPKWSLFANSPDEVSSERMIHGFSMTRLDGTKKRNDGTNLSIVNPVEVIRQRMMIDAARERQNQIDANAEMLREIGKRQPKAWRSDWH

>Smar-EFLamide
MKLLCIDPSLFIFTYLVLSLIPSTNLVSSQEIERVPESYGMDSSVLKRSARISQEDVYRMFVLLNKIKDRGGIKRIGSEFIGKRYSQAEDIEKKLGSEFLGKRGIGSEFLGKRSQRSQAEN

>Smar-EH
MRQSESQRAAVSTVVILLLLHLDRASSRSINLCIQNCAQCKKMFGPYFEGQLCAETCIELRAKMTPDCADANSISPFLNKFER

>Smar-EH-like
MACLLSAACVLASVACISEESSLGVCIRNCGQCKRMYGDFFLGQHCAEECLQTEGRGDLPDCNNPKSLYRFLGKT

>Smar-Elevenin
MKIVIRSVIPLLICLILLLVFVSHSEQVDCRIYVFAPKCRGISAKRGIDPPGRQQQPNQKLDINADPYAPYEYNPTDYSWDERASDSNYGSRISSDFVAPIFDSSSPPSRFKNSDDRDQGRFLRALVKMYFDQRQDSED

>Smar-ETH
MTTFTSSFFVPTQMYILVLFAVFLLQTEAQFFAKTSKNLPRIGRRVDHQQEVSEIIPPSFKHLLAFVRKFDSDANGCLSPEELIEIPLFQMAFENEDFTPLEIAADVVEEYKSEVEGNLKDIVAKVLAASYAEKK

>Smar-FMRFamide
MQQVLLSFLVVTIPALAIPVSARCFLQPSLGGVPNENSSPLLCTLMSSRESADEVSEDDDKLMDSDALEPINNELTSEENSMDDDQKDALLRSLRQEPGHKFLRFGRASSNHNFLRFGRDPEHKFLRFGRDQHKFLRFGRSVANGEENRLRLRENQSKMTVLPMFMRLGRGPEHVFMRFGRQGSNSEGHKFMRFGRTQNDTPVQDENTQQEKA

>Smar-FMRFamide-like
..TEQKFARFDRSNFDENGDPQENWLDSENRKLLSLRSARLIRELGRKRGSLEKNFLRFGRSLENGNDINTKRDTSSLDDSHMSYLKSDQLNQNKLVPKRNKLENNFLRFGRWKTENLNEQYE

>Smar-ILP
MSSHLRTAAAVLILLSVVLSIAAQESAYFQELFTPHEIKIRAKQKYCGRNLVEVLQLVCARNVLNNLEAEEISDLLGNDFARSLMGIKHKIQTRGITDECCRKGCSFNELKSYCAEEP

>Smar-Inotocin
MKSTHFVNIFIYSVFIFIMADGCYITNCPPGGKRSGNEKSGRGVRQCTPCGPGGIGRCYGPDICCGANVGCFVGTRESAICRLENLYSLPCQNEGRACGTDGTCSADGFCCSTDQCKADESCRGKVHHTNNLQRVLDGEIDLNDVMGPQR

>Smar-ITP
MNHTLFFRVFVVLSAIIASTCLVSARSLNLEDVSGVHRINKRSFHTLGCLGDYDTAGFSRLDRLCEDCYDMYRDSQVRAMCRSSCFTTDTFKKCAEALLVNMEEEKLGDVVNRLYGRD

>Myosuppressin
MKLLTLYKILLVISVVIIPSIFSHPPPQCDTDDPLPPRLLRICNALRTISEYTELMEDYLDEEVMHTLAVSDMKRDERDTGHVFMRFG

>Smar-NPF
MSLSISTRSTLALICVIVVLYVFCAPAQATSGPDQAVSMTEALKYLQALDKYYSQVARPRFGRSLPMRYPSKDMSVEAEANRLLEQRRR

>Smar-NPLP1
MQGRTCLRMLLLAIVFSHLTQGALQDTGPEATTISGRLTELSGQQERIKPPVKRYVGSVARAGGLPPFFHGKRHEIESTDDEDENNEIIKRYLGSIVRQGIFSHNKRQDEEEMLDDAVEKRHLGSVLRAGDSRLVGRDLFSSLQDKRFMGSLARAGELGPGGRMSGKKRYLGSVARVDGIPFRAKRTPQDTESPDWESEENLDDNEEDVGNEEEWEEDDLLIPFKRNIASLARNGWLPHTRSLRRHDAPTYSVSEEAPKRNIYYRPASASGRSRLLGWLRREEARLKEEAEARNGQGKRTIAALARSGELPVRYRFSRSADRLPAPPFMRMATYRGGGGGGYSIRSPHAFASLHEGGWNRFKRSFEQLDRMQMHLDALDDLCDSWDLERCGPHKGEKKSMERHSSESRKDEGGEMSMTV

>Smar-Proctolin
MTVKCAVVFALLMTLMYCWSAQCRYLPSRADNTRAEEIREILRE...

>Smar-Pyrokinin
MWVSLCVWFGIFCSGFGLSLFEVDKRQGLIPFPRIGRALPVDNSILLSSQEIRDALYQGLFRSNRLKRDVEVEGGDDWAEITNDGFQGSPDEKKTVEKTSKLAPRLGRSRYRIGASPFQPRLGRAYVSFGPRLGRSKLPPRLGKRLNRN

>Smar-RYamide
MFSSKQSTLFYLLTILGMCALFWRVESQQFYPNGRYGRSDKMPALSDVRGTREMTVSFFGDGTVQCTYTGYPDFYRCK...

>Smar-SIFamide
MASKTTIILLVVAIVAICLVVDVTSANYRKPPFNGSIFGKRAPEDSTAEKLFAMCAIATDACSQWFPASEAK

>Smar-sNPF
MHSIITCSFLLFATLFILTFPLENASPAPYTDYDNIRELYELLLRNEALNDRTGHQVVRKGSRDPSLRLRFGRRSDPAWQEGREPSLRLRFGRSADDVRGYSKQLRFGRSDDTAWQHDVASSENDVIEN

>Smar-Sulfakinin
MNCTVIFLVHYLVLVCTFVSSSNGSPSVARSSKHDSRLANIMAPYLYLKLHDQAARRPVDSESESVKDEVESEFDDFFESFDKTKRNFDDYGHMRFGKREFDDYGHPRYGRSA

>Smar-Tachykinin
MMNFRGIWSLRLSIFIIFFFGPIFGQQVQSLNDKIKNMQLDEDNLLRKSRNVFMGMRGKKMSMDQDRDLQRAATSIAEIKRINGFMAMRGNKINSIHNIFDEVENPYIFPGDKRAKGFLGMRGKKQPSGSEMNRWSNSKFFAMRGKRD

>Smar-Trunk (PTTH?)
MMKRKKVSFLFGFVLGFYWLAGGQGNATGENVMTNFLETMGKATSKTSKTTETNDRLWTCEWSEHWLDLGNDYFPRYIRTAKCTTEKCWFNFFKCEGRAFTVKVLRKRQDECISHVNNKTVILLEDWVFEERAVNFCCVCVPVWKKKKATLIFFFFSS
