# Supplementary material for: The First Myriapod Genome Sequence Reveals Conservative Arthropod Gene Content and Genome Organisation in the Centipede Strigamia maritima
Source: PLoS Biol. 2014 Nov 25;12(11):e1002005. doi: 10.1371/journal.pbio.1002005 (PMC4244043; doi:10.1371/journal.pbio.1002005)
Supplement: Table S2 — Set of species used in the comparative genomics analyses related to the S. maritima genome. Columns include, in this order, scientific names, the species code according to UNIPROT, the number of the longest unique transcript used in the analyses, the data source, and the date in which data were retrieved. (DOCX) [file pbio.1002005.s036.docx]

**Table S2**. **Set of species used in the comparative genomics analyses related to the *S. maritima* genome.**

| **Species name** | **Species Code** | **Unique longest transcripts** | **Source** | **As on** |
| --- | --- | --- | --- | --- |
| *Strigamia maritima* | STRMM | 14,959 | Ensembl - Metazoa 20 | 09/2013 |
| *Pediculus humanus* | PEDHC | 10,761 | Vectorbase | 01/2011 |
| *Lottia gigantea* | LOTGI | 23,701 | JGI | 09/2010 |
| *Capitella teleta* | 283909 | 31,857 | Ensembl - Metazoa 20 | 09/2013 |
| *Nematostella vectensis* | NEMVE | 24,424 | Quest for Orthologs – 2011.04 | 10/2011 |
| *Caenorhabditis elegans* | CAEEL | 20,333 | WormBase | 05/2012 |
| *Helobdella robusta* | HELRO | 23,327 | JGI | 04/2012 |
| *Daphnia pulex* | DAPPU | 30,335 | JGI | 12/2011 |
| *Ixodes scapularis* | IXOSC | 20,473 | Quest for Orthologs - 2011.04 | 12/2012 |
| *Acyrthosiphon pisum* | ACYPI | 27,584 | Aphid | 11/2011 |
| *Tribolium castaneum* | TRICA | 16,573 | BeetleBASE - HGSC | 12/2011 |
| *Bombyx mori* | BOMMO | 14,593 | SilkDB | 12/2011 |
| *Anopheles gambiae* | ANOGA | 12,580 | VectorBASE | 05/2012 |
| *Drosophila melanogaster* | DROME | 13,755 | Quest for Orthologs - 2012.05 | 07/2012 |
| *Nasonia vitripennis* | NASVI | 15,073 | BCM | 11/2011 |
| *Strongylocentrotus purpuratus* | STRPU | 28,760 | HGSC | 12/2011 |
| *Branchiostoma floridae* | BRAFL | 28,394 | Quest for Orthologs - 2011.04 | 12/2012 |
| *Homo sapiens* | HUMAN | 19,997 | Quest for Orthologs - 2012.05 | 07/2012 |
