# Supplementary material for: The First Myriapod Genome Sequence Reveals Conservative Arthropod Gene Content and Genome Organisation in the Centipede Strigamia maritima
Source: PLoS Biol. 2014 Nov 25;12(11):e1002005. doi: 10.1371/journal.pbio.1002005 (PMC4244043; doi:10.1371/journal.pbio.1002005)
Supplement: Table S3 — Orthologues detected between a given species and S. maritima . First column indicates how many trees have been used to detect such orthologues. Columns “uniq” refers to the number of orthologues detected for each pair of species after removing redundancy. In one-to-many and many-to-many orthology relationships it is possible to count a given protein more than once. Regarding the ratios values, “all” column refers to the orthology ratio computed using all orthologue pairs meanwhile “uniq” refers to the ratio computed using “uniq” columns. (DOCX) [file pbio.1002005.s037.docx]

**Table S3**. **Orthologues detected between a given species and *S. maritima*.**

| **number of trees used** | ***S. maritima*** | | **Other species** | | | **ratios** | |
| --- | --- | --- | --- | --- | --- | --- | --- |
|  | **orthologues** | **uniq** | **Sp. Code** | **orthologues** | **uniq** | **all** | **uniq** |
| 5726 | 16944 | 7050 | NEMVE | 7770 | 5660 | 2.18 | 1.25 |
| 6404 | 18891 | 7707 | TRICA | 8889 | 6291 | 2.13 | 1.23 |
| 6116 | 16729 | 7195 | LOTGI | 7895 | 5924 | 2.12 | 1.21 |
| 4946 | 12607 | 6004 | BOMMO | 6058 | 4649 | 2.08 | 1.29 |
| 5231 | 13335 | 6346 | IXOSC | 6775 | 5133 | 1.97 | 1.24 |
| 6359 | 17196 | 7590 | 283909 | 8858 | 6680 | 1.94 | 1.14 |
| 5862 | 12841 | 6721 | PEDHC | 6645 | 5309 | 1.93 | 1.27 |
| 4649 | 11604 | 5629 | HELRO | 6088 | 4809 | 1.91 | 1.17 |
| 5802 | 16228 | 7230 | STRPU | 9065 | 6956 | 1.79 | 1.04 |
| 5555 | 19058 | 6679 | NASVI | 11170 | 7577 | 1.71 | 0.88 |
| 5446 | 12060 | 6256 | ANOGA | 7078 | 5384 | 1.70 | 1.16 |
| 5478 | 12482 | 6272 | DROME | 7543 | 5642 | 1.65 | 1.11 |
| 5848 | 14966 | 6918 | ACYPI | 9199 | 6798 | 1.63 | 1.02 |
| 4204 | 9401 | 4992 | CAEEL | 5810 | 4465 | 1.62 | 1.12 |
| 6207 | 16226 | 7269 | HUMAN | 10170 | 7871 | 1.60 | 0.92 |
| 5918 | 14604 | 7007 | DAPPU | 9511 | 6509 | 1.54 | 1.08 |
| 5804 | 15252 | 6945 | BRAFL | 11364 | 6957 | 1.34 | 1.00 |
