# Supplementary material for: The First Myriapod Genome Sequence Reveals Conservative Arthropod Gene Content and Genome Organisation in the Centipede Strigamia maritima
Source: PLoS Biol. 2014 Nov 25;12(11):e1002005. doi: 10.1371/journal.pbio.1002005 (PMC4244043; doi:10.1371/journal.pbio.1002005)
Supplement: Table S4 — Orthology ratios for a given species related to S. maritima . This table is similar to Table S3, but in this case orthology relationships with ten or more proteins for any of the species are discarded in order to avoid biases introduced by species-specific gene family expansions. (DOCX) [file pbio.1002005.s038.docx]

**Table S4**. **Orthology ratios for a given species related to *S. maritima.***

| **number of trees used** | ***S. maritima*** | | **Other species** | | | **ratios** | |
| --- | --- | --- | --- | --- | --- | --- | --- |
|  | **orthologues** | **uniq** | **Sp. code** | **orthologues** | **uniq** | **all** | **uniq** |
| 5382 | 9869 | 6397 | NEMVE | 6883 | 5429 | 1.43 | 1.18 |
| 4700 | 7065 | 5288 | BOMMO | 5314 | 4502 | 1.33 | 1.17 |
| 5637 | 8039 | 6142 | PEDHC | 6149 | 5252 | 1.31 | 1.17 |
| 5799 | 9148 | 6531 | LOTGI | 7021 | 5739 | 1.30 | 1.14 |
| 4996 | 7687 | 5643 | IXOSC | 5918 | 4925 | 1.30 | 1.15 |
| 6019 | 8979 | 6700 | TRICA | 7085 | 5899 | 1.27 | 1.14 |
| 6036 | 9632 | 6770 | 283909 | 7623 | 6204 | 1.26 | 1.09 |
| 5217 | 7545 | 5682 | ANOGA | 6104 | 5203 | 1.24 | 1.09 |
| 4048 | 6365 | 4556 | CAEEL | 5178 | 4231 | 1.23 | 1.08 |
| 4438 | 6917 | 5033 | HELRO | 5663 | 4720 | 1.22 | 1.07 |
| 5447 | 8834 | 6279 | STRPU | 7233 | 5998 | 1.22 | 1.05 |
| 5452 | 8505 | 6288 | BRAFL | 7007 | 5868 | 1.21 | 1.07 |
| 5622 | 8561 | 6210 | DAPPU | 7184 | 5901 | 1.19 | 1.05 |
| 5237 | 7538 | 5720 | DROME | 6407 | 5444 | 1.18 | 1.05 |
| 5529 | 8208 | 6122 | ACYPI | 7219 | 6092 | 1.14 | 1.00 |
| 5905 | 8897 | 6505 | HUMAN | 9054 | 7613 | 0.98 | 0.85 |
| 5095 | 7678 | 5771 | NASVI | 8076 | 6818 | 0.95 | 0.85 |
