# Supplementary material for: The First Myriapod Genome Sequence Reveals Conservative Arthropod Gene Content and Genome Organisation in the Centipede Strigamia maritima
Source: PLoS Biol. 2014 Nov 25;12(11):e1002005. doi: 10.1371/journal.pbio.1002005 (PMC4244043; doi:10.1371/journal.pbio.1002005)
Supplement: Table S5 — Newly added Chelicerata species used to increase the taxon sampling for the species phylogeny. First column indicates the scientific species name, the second one indicates which strategy has been used to identify single copy protein-coding genes. Third column shows how many single-copy genes have been identified in each species from the initial set of 1,491 used to reconstruct the species phylogeny. Last two columns show the data source and the date on which data were retrieved. (DOCX) [file pbio.1002005.s039.docx]

**Table S5. Newly added Chelicerata species used to increase the taxon sampling for the species phylogeny.**

| **Species name** | **Strategy used** | **Identified proteins** | **Source** | **As on** |
| --- | --- | --- | --- | --- |
| *Centruroides sculpturatus* | Exonerate | 756 | BCM - HGSC | 07/2013 |
| *Latrodectus hesperus* | Exonerate | 512 | BCM - HGSC | 02/2013 |
| *Parasteatoda tepidariorum* | Exonerate | 1,058 | BCM - HGSC | 01/2013 |
| *Metaseiulus occidentalis* | BBH - Blast | 699 | BCM - HGSC | 07/2013 |
| *Tetranychus urticae* | BBH - Blast | 659 | BEG - UGent | 11/2012 |
