# Supplementary material for: The First Myriapod Genome Sequence Reveals Conservative Arthropod Gene Content and Genome Organisation in the Centipede Strigamia maritima
Source: PLoS Biol. 2014 Nov 25;12(11):e1002005. doi: 10.1371/journal.pbio.1002005 (PMC4244043; doi:10.1371/journal.pbio.1002005)
Supplement: Table S6 — Results after applying the different statistical tests implemented in CONSEL for the alternative placement of S. maritima relative to Pancrustacea and Chelicerata groups of species (as shown in Figure S4) in the context of the 18 species used for the phylogenomics analyses. The “item” column relates to Figure S4 as follows: (1) topology arrangement corresponding to Figure S4 left-hand panel, in which S. maritima was grouped with Chelicerata species. (2) Topology arrangement corresponding to Figure S4 central panel, in which S. maritima branches off before the split of Pancrustacea and Chelicerata. (3) Topology arrangement corresponding to Figure S4 right-hand panel, in which S. maritima was grouped with Pancrustacea species. (DOCX) [file pbio.1002005.s040.docx]

**Table S6.** **Results after applying the different statistical tests implemented in CONSEL for the alternative placement of *S. maritima* relative to Pancrustacea and Chelicerata groups of species (as shown in Fig. S4) in the context of the 18 species used for the phylogenomics analyses.**

| **rank** | **item** | **obs** | **au** | **np** | **bp** | **pp** | **kh** | **sh** | **wkh** | **wsh** |
| --- | --- | --- | --- | --- | --- | --- | --- | --- | --- | --- |
| 1st | (1) | -479.7 | 1.000 | 1.000 | 1.000 | 1.000 | 1.000 | 1.000 | 1.000 | 1.000 |
| 2nd | (3) | -479.7 | 1e-05 | 1e-06 | 0 | 5e-209 | 0 | 0 | 0 | 0 |
| 3rd | (2) | 1089.7 | 9e-09 | 2e-07 | 0 | 0 | 0 | 0 | 0 | 0 |
