# Supplementary material for: The First Myriapod Genome Sequence Reveals Conservative Arthropod Gene Content and Genome Organisation in the Centipede Strigamia maritima
Source: PLoS Biol. 2014 Nov 25;12(11):e1002005. doi: 10.1371/journal.pbio.1002005 (PMC4244043; doi:10.1371/journal.pbio.1002005)
Supplement: Table S7 — Results after applying the different statistical tests implemented in CONSEL for the alternative placement of S. maritima relative to the two arthropod groups, Pancrustacea and Chelicerata (as shown in Figure S4), with the inclusion of extra chelicerates. Taxon sampling for the Chelicerata was increased after including sequences from five additional species. In order to reduce any potential bias introduced by distant and/or fast-evolving out-groups, six out-group species from the initial set were removed. The “item” column relates to Figure S4 as follows: (1) topology arrangement corresponding to Figure S4 left-hand panel, in which S. maritima was grouped with Chelicerata species. (2) Topology arrangement corresponding to Figure S4 central panel, in which S. maritima branches off before the split of Pancrustacea and Chelicerata. (3) Topology arrangement corresponding to Figure S4 right-hand panel, in which S. maritima was grouped with Pancrustacea species. (DOCX) [file pbio.1002005.s041.docx]

**Table S7.** **Results after applying the different statistical tests implemented in CONSEL for the alternative placement of *S. maritima* relative to the two arthropod groups, Pancrustacea and Chelicerata (as shown in Fig. S4), with the inclusion of extra chelicerates.**

| **rank** | **item** | **obs** | **au** | **np** | **bp** | **pp** | **kh** | **sh** | **wkh** | **wsh** |
| --- | --- | --- | --- | --- | --- | --- | --- | --- | --- | --- |
| 1st | (1) | -68.5 | 0.786 | 0.718 | 0.723 | 1.000 | 0.739 | 0.872 | 0.739 | 0.871 |
| 2nd | (3) | 68.5 | 0.316 | 0.245 | 0.237 | 2e-30 | 0.261 | 0.390 | 0.261 | 0.394 |
| 3rd | (2) | 153.1 | 0.072 | 0.037 | 0.040 | 3e-67 | 0.069 | 0.118 | 0.069 | 0.119 |
