# Supplementary material for: The First Myriapod Genome Sequence Reveals Conservative Arthropod Gene Content and Genome Organisation in the Centipede Strigamia maritima
Source: PLoS Biol. 2014 Nov 25;12(11):e1002005. doi: 10.1371/journal.pbio.1002005 (PMC4244043; doi:10.1371/journal.pbio.1002005)
Supplement: Table S8 — Enriched functional GO Terms for the ten largest clusters of duplicated S. maritima protein-coding genes specifically expanded in the centipede lineage, as compared with the whole genome. (DOCX) [file pbio.1002005.s042.docx]

**Table S8**. **Enriched functional GO Terms for the 10 largest clusters of duplicated *S. maritima* protein-coding genes as compared with the whole genome.**

| **Number of protein in cluster** | **Ontology** | **Go Term** | **Go Term Name** |
| --- | --- | --- | --- |
| 50 | Cellular component | GO:0016020 | membrane |
| 50 | Cellular component | GO:0030288 | outer membrane-bounded periplasmic space |
| 50 | Cellular component | GO:0030312 | external encapsulating structure |
| 50 | Molecular function | GO:0004871 | signal transducer activity |
| 50 | Molecular function | GO:0005215 | transporter activity |
| 50 | Molecular function | GO:0005234 | extracellular-glutamate-gated ion channel activity |
| 50 | Molecular function | GO:0022857 | transmembrane transporter activity |
| 52 | Molecular function | GO:0005515 | protein binding |
| 52 | Molecular function | GO:0008270 | zinc ion binding |
| 52 | Molecular function | GO:0043167 | ion binding |
| 52 | Biological process | GO:0006259 | DNA metabolic process |
| 52 | Biological process | GO:0006278 | RNA-dependent DNA replication |
| 52 | Biological process | GO:0009058 | biosynthetic process |
| 52 | Biological process | GO:0034641 | cellular nitrogen compound metabolic process |
| 52 | Molecular function | GO:0003723 | RNA binding |
| 52 | Molecular function | GO:0003964 | RNA-directed DNA polymerase activity |
| 52 | Molecular function | GO:0016779 | nucleotidyltransferase activity |
| 54 | Molecular function | GO:0005515 | protein binding |
| 63 | Cellular component | GO:0016021 | integral to membrane |
| 63 | Biological process | GO:0050877 | neurological system process |
| 63 | Biological process | GO:0050912 | detection of chemical stimulus involved in sensory perception of taste |
| 63 | Molecular function | GO:0008527 | taste receptor activity |
| 76 | Cellular component | GO:0005634 | nucleus |
| 76 | Cellular component | GO:0043226 | organelle |
| 76 | Molecular function | GO:0003677 | DNA binding |
| 79 | Cellular component | GO:0005634 | nucleus |
| 79 | Cellular component | GO:0043226 | organelle |
| 79 | Biological process | GO:0006259 | DNA metabolic process |
| 79 | Biological process | GO:0015074 | DNA integration |
| 79 | Molecular function | GO:0003676 | nucleic acid binding |
| 79 | Molecular function | GO:0003677 | DNA binding |
| 79 | Molecular function | GO:0008270 | zinc ion binding |
| 79 | Molecular function | GO:0043167 | ion binding |
| 98 | Cellular component | GO:0016020 | membrane |
| 98 | Biological process | GO:0006810 | transport |
| 98 | Biological process | GO:0006814 | sodium ion transport |
| 98 | Molecular function | GO:0005272 | sodium channel activity |
| 98 | Molecular function | GO:0022857 | transmembrane transporter activity |
| 201 | Biological process | GO:0006259 | DNA metabolic process |
| 201 | Biological process | GO:0015074 | DNA integration |
| 201 | Biological process | GO:0034641 | cellular nitrogen compound metabolic process |
| 201 | Molecular function | GO:0003676 | nucleic acid binding |
| 292 | Cellular component | GO:0005622 | intracellular |
| 292 | Cellular component | GO:0005623 | cell |
| 292 | Cellular component | GO:0005634 | nucleus |
| 292 | Cellular component | GO:0043226 | organelle |
| 292 | Biological process | GO:0006259 | DNA metabolic process |
| 292 | Biological process | GO:0015074 | DNA integration |
| 292 | Molecular function | GO:0003676 | nucleic acid binding |
| 292 | Molecular function | GO:0003677 | DNA binding |
| 292 | Molecular function | GO:0008270 | zinc ion binding |
| 292 | Molecular function | GO:0043167 | ion binding |
