# Supplementary material for: The First Myriapod Genome Sequence Reveals Conservative Arthropod Gene Content and Genome Organisation in the Centipede Strigamia maritima
Source: PLoS Biol. 2014 Nov 25;12(11):e1002005. doi: 10.1371/journal.pbio.1002005 (PMC4244043; doi:10.1371/journal.pbio.1002005)
Supplement: Table S9 — Statistics regarding the duplications of centipede genes relative to seven specific ages detected using all available trees on the phylome. (DOCX) [file pbio.1002005.s043.docx]

**Table S9**. **Statistics regarding the duplications of centipede genes relative to seven specific ages detected using all available trees on the phylome.**

| **Age** | **Events** | **Trees with events (trees: 11,112)** | **Ratio** |
| --- | --- | --- | --- |
| **1:** *S. maritima* | 10767 | 4097 | 0.9690 |
| **2:** Arthropoda I | 152 | 127 | 0.0137 |
| **3:** Arthropoda II | 1214 | 871 | 0.1093 |
| **4:** Ecdysozoa | 125 | 111 | 0.0112 |
| **5:** Protostomia | 486 | 391 | 0.0437 |
| **6:** Bilateria | 2333 | 1517 | 0.2100 |
| **7:** Eumetazoa | 6065 | 2645 | 0.5458 |
