# Supplementary material for: The First Myriapod Genome Sequence Reveals Conservative Arthropod Gene Content and Genome Organisation in the Centipede Strigamia maritima
Source: PLoS Biol. 2014 Nov 25;12(11):e1002005. doi: 10.1371/journal.pbio.1002005 (PMC4244043; doi:10.1371/journal.pbio.1002005)
Supplement: Table S10 — Enriched functional GO terms for proteins duplicated at the different relative ages shown in Table S9. Columns show relative age, gene ontology namespace, the GO term id, and its name, respectively. (DOCX) [file pbio.1002005.s044.docx]

**Table S10**. **Enriched functional GO terms for proteins duplicated at the different relative ages shown in Table S9.**

| **Age** | **Ontology** | **Go Term** | **Go Name** |
| --- | --- | --- | --- |
| **1** | Biological process | GO:0006259 | DNA metabolic process |
| **1** | Biological process | GO:0006278 | RNA-dependent DNA replication |
| **1** | Biological process | GO:0006313 | transposition |
| **1** | Biological process | GO:0006810 | transport |
| **1** | Biological process | GO:0006814 | sodium ion transport |
| **1** | Biological process | GO:0009253 | peptidoglycan catabolic process |
| **1** | Biological process | GO:0015074 | DNA integration |
| **1** | Biological process | GO:0032196 | transposition |
| **1** | Biological process | GO:0034641 | cellular nitrogen compound metabolic process |
| **1** | Biological process | GO:0050877 | neurological system process |
| **1** | Molecular function | GO:0003676 | nucleic acid binding |
| **1** | Molecular function | GO:0003964 | RNA-directed DNA polymerase activity |
| **1** | Molecular function | GO:0004523 | ribonuclease H activity |
| **1** | Molecular function | GO:0004803 | transposase activity |
| **1** | Molecular function | GO:0005234 | extracellular-glutamate-gated ion channel activity |
| **1** | Molecular function | GO:0005272 | sodium channel activity |
| **1** | Molecular function | GO:0008745 | N-acetylmuramoyl-L-alanine amidase activity |
| **1** | Molecular function | GO:0016705 | oxidoreductase activity |
| **1** | Molecular function | GO:0022857 | transmembrane transporter activity |
| **1** | Cellular Component | GO:0016020 | membrane |
| **3** | Biological process | GO:0006030 | chitin metabolic process |
| **3** | Biological process | GO:0006066 | alcohol metabolic process |
| **3** | Biological process | GO:0006259 | DNA metabolic process |
| **3** | Biological process | GO:0006508 | proteolysis |
| **3** | Biological process | GO:0006810 | transport |
| **3** | Biological process | GO:0006814 | sodium ion transport |
| **3** | Biological process | GO:0015074 | DNA integration |
| **3** | Biological process | GO:0055085 | transmembrane transport |
| **3** | Molecular function | GO:0003676 | nucleic acid binding |
| **3** | Molecular function | GO:0004252 | serine-type endopeptidase activity |
| **3** | Molecular function | GO:0004601 | peroxidase activity |
| **3** | Molecular function | GO:0005215 | transporter activity |
| **3** | Molecular function | GO:0005234 | extracellular-glutamate-gated ion channel activity |
| **3** | Molecular function | GO:0008061 | chitin binding |
| **3** | Molecular function | GO:0008233 | peptidase activity |
| **3** | Molecular function | GO:0008812 | choline dehydrogenase activity |
| **3** | Molecular function | GO:0020037 | heme binding |
| **3** | Molecular function | GO:0022857 | transmembrane transporter activity |
| **3** | Cellular Component | GO:0005921 | gap junction |
| **3** | Cellular Component | GO:0016020 | membrane |
| **4** | Biological process | GO:0006208 | pyrimidine nucleobase catabolic process |
| **4** | Biological process | GO:0006810 | transport |
| **4** | Biological process | GO:0006811 | ion transport |
| **4** | Molecular function | GO:0005230 | extracellular ligand-gated ion channel activity |
| **4** | Molecular function | GO:0022857 | transmembrane transporter activity |
| **4** | Cellular Component | GO:0016020 | membrane |
| **4** | Cellular Component | GO:0045211 | postsynaptic membrane |
| **5** | Biological process | GO:0005975 | carbohydrate metabolic process |
| **5** | Biological process | GO:0006508 | proteolysis |
| **5** | Biological process | GO:0006810 | transport |
| **5** | Biological process | GO:0006835 | dicarboxylic acid transport |
| **5** | Biological process | GO:0006836 | neurotransmitter transport |
| **5** | Biological process | GO:0007166 | cell surface receptor signaling pathway |
| **5** | Biological process | GO:0007186 | G-protein coupled receptor signaling pathway |
| **5** | Biological process | GO:0009253 | peptidoglycan catabolic process |
| **5** | Molecular function | GO:0004930 | G-protein coupled receptor activity |
| **5** | Molecular function | GO:0005328 | neurotransmitter:sodium symporter activity |
| **5** | Molecular function | GO:0008233 | peptidase activity |
| **5** | Molecular function | GO:0008745 | N-acetylmuramoyl-L-alanine amidase activity |
| **5** | Molecular function | GO:0016810 | hydrolase activity |
| **5** | Molecular function | GO:0022857 | transmembrane transporter activity |
| **5** | Cellular Component | GO:0016020 | membrane |
| **5** | Cellular Component | GO:0016021 | integral to membrane |
| **6** | Biological process | GO:0006508 | proteolysis |
| **6** | Biological process | GO:0006810 | transport |
| **6** | Biological process | GO:0006811 | ion transport |
| **6** | Biological process | GO:0006836 | neurotransmitter transport |
| **6** | Biological process | GO:0007186 | G-protein coupled receptor signaling pathway |
| **6** | Biological process | GO:0015074 | DNA integration |
| **6** | Biological process | GO:0055085 | transmembrane transport |
| **6** | Molecular function | GO:0004252 | serine-type endopeptidase activity |
| **6** | Molecular function | GO:0004871 | signal transducer activity |
| **6** | Molecular function | GO:0004872 | receptor activity |
| **6** | Molecular function | GO:0005234 | extracellular-glutamate-gated ion channel activity |
| **6** | Molecular function | GO:0005328 | neurotransmitter:sodium symporter activity |
| **6** | Molecular function | GO:0008233 | peptidase activity |
| **6** | Molecular function | GO:0022857 | transmembrane transporter activity |
| **6** | Molecular function | GO:0050254 | rhodopsin kinase activity |
| **6** | Cellular Component | GO:0005886 | plasma membrane |
| **6** | Cellular Component | GO:0016020 | membrane |
| **6** | Cellular Component | GO:0016021 | integral to membrane |
| **6** | Cellular Component | GO:0030054 | cell junction |
| **6** | Cellular Component | GO:0030288 | outer membrane-bounded periplasmic space |
| **6** | Cellular Component | GO:0030312 | external encapsulating structure |
| **6** | Cellular Component | GO:0045211 | postsynaptic membrane |
| **7** | Biological process | GO:0006184 | GTP catabolic process |
| **7** | Biological process | GO:0006468 | protein phosphorylation |
| **7** | Biological process | GO:0006508 | proteolysis |
| **7** | Biological process | GO:0006754 | ATP biosynthetic process |
| **7** | Biological process | GO:0006810 | transport |
| **7** | Biological process | GO:0006812 | cation transport |
| **7** | Biological process | GO:0006836 | neurotransmitter transport |
| **7** | Biological process | GO:0006913 | nucleocytoplasmic transport |
| **7** | Biological process | GO:0007017 | microtubule-based process |
| **7** | Biological process | GO:0007018 | microtubule-based movement |
| **7** | Biological process | GO:0007156 | homophilic cell adhesion |
| **7** | Biological process | GO:0007165 | signal transduction |
| **7** | Biological process | GO:0007223 | Wnt receptor signaling pathway |
| **7** | Biological process | GO:0007264 | small GTPase mediated signal transduction |
| **7** | Biological process | GO:0008152 | metabolic process |
| **7** | Biological process | GO:0009056 | catabolic process |
| **7** | Biological process | GO:0015031 | protein transport |
| **7** | Biological process | GO:0016055 | Wnt receptor signaling pathway |
| **7** | Biological process | GO:0043401 | steroid hormone mediated signaling pathway |
| **7** | Biological process | GO:0043687 | post-translational protein modification |
| **7** | Biological process | GO:0044281 | small molecule metabolic process |
| **7** | Biological process | GO:0051246 | regulation of protein metabolic process |
| **7** | Biological process | GO:0051276 | chromosome organization |
| **7** | Biological process | GO:0051603 | proteolysis involved in cellular protein catabolic process |
| **7** | Biological process | GO:0055085 | transmembrane transport |
| **7** | Biological process | GO:0055114 | oxidation-reduction process |
| **7** | Biological process | GO:0065003 | macromolecular complex assembly |
| **7** | Molecular function | GO:0003707 | steroid hormone receptor activity |
| **7** | Molecular function | GO:0003777 | microtubule motor activity |
| **7** | Molecular function | GO:0003924 | GTPase activity |
| **7** | Molecular function | GO:0003995 | acyl-CoA dehydrogenase activity |
| **7** | Molecular function | GO:0004222 | metalloendopeptidase activity |
| **7** | Molecular function | GO:0004298 | threonine-type endopeptidase activity |
| **7** | Molecular function | GO:0004386 | helicase activity |
| **7** | Molecular function | GO:0004674 | protein serine/threonine kinase activity |
| **7** | Molecular function | GO:0004702 | receptor signaling protein serine/threonine kinase activity |
| **7** | Molecular function | GO:0004767 | sphingomyelin phosphodiesterase activity |
| **7** | Molecular function | GO:0004871 | signal transducer activity |
| **7** | Molecular function | GO:0005328 | neurotransmitter:sodium symporter activity |
| **7** | Molecular function | GO:0005509 | calcium ion binding |
| **7** | Molecular function | GO:0005524 | ATP binding |
| **7** | Molecular function | GO:0005525 | GTP binding |
| **7** | Molecular function | GO:0008026 | ATP-dependent helicase activity |
| **7** | Molecular function | GO:0008233 | peptidase activity |
| **7** | Molecular function | GO:0008568 | microtubule-severing ATPase activity |
| **7** | Molecular function | GO:0016301 | kinase activity |
| **7** | Molecular function | GO:0016491 | oxidoreductase activity |
| **7** | Molecular function | GO:0016772 | transferase activity |
| **7** | Molecular function | GO:0016887 | ATPase activity |
| **7** | Molecular function | GO:0016905 | myosin heavy chain kinase activity |
| **7** | Molecular function | GO:0019787 | small conjugating protein ligase activity |
| **7** | Molecular function | GO:0019829 | cation-transporting ATPase activity |
| **7** | Molecular function | GO:0019899 | enzyme binding |
| **7** | Molecular function | GO:0042624 | ATPase activity |
| **7** | Molecular function | GO:0046872 | metal ion binding |
| **7** | Molecular function | GO:0046982 | protein heterodimerization activity |
| **7** | Cellular Component | GO:0000786 | nucleosome |
| **7** | Cellular Component | GO:0005839 | proteasome core complex |
| **7** | Cellular Component | GO:0005856 | cytoskeleton |
| **7** | Cellular Component | GO:0005875 | microtubule associated complex |
| **7** | Cellular Component | GO:0016021 | integral to membrane |
