# Supplementary material for: The First Myriapod Genome Sequence Reveals Conservative Arthropod Gene Content and Genome Organisation in the Centipede Strigamia maritima
Source: PLoS Biol. 2014 Nov 25;12(11):e1002005. doi: 10.1371/journal.pbio.1002005 (PMC4244043; doi:10.1371/journal.pbio.1002005)
Supplement: Table S11 — Overview of S. maritima mitochondrial genome. (DOCX) [file pbio.1002005.s045.docx]

**Table S11. Overview of *Strigamia maritima* mitochondrial genome.**

| **Gene** | **Strand** | **Start position** | **End position** | **Length (bp)** | **Start Codon** | **Stop Codon** | **Intergenic nucleotides** |
| --- | --- | --- | --- | --- | --- | --- | --- |
| **cox1** | + | 1 | 1557 | 1557 | ATT | TAA |  |
| **cox2** | + | 1532 | 2215 | 684 | ATG | TAG | -25 |
| **cox3** | + | 2221 | 3063 | 843 | ATG | TAA | 6 |
| **nad6** | + | 3060 | 3524 | 465 | ATA | TAG | -3 |
| **nad2** | + | 3525 | 4487 | 963 | ATT | TAA | 3 |
| **trnF** | - | 4527 | 4606 | 79 |  |  | 40 |
| **nad5** | - | 4606 | 6306 | 1701 | ATG | TAG | 0 |
| **trnH** | - | 6287 | 6347 | 60 |  |  | -9 |
| **nad4** | - | 6348 | 7664 | 1317 | ATG | TAA | 1 |
| **nad4l** | - | 7658 | 7921 | 264 | ATT | TAA | -6 |
| **trnP** | - | 7909 | 7972 | 63 |  |  | -12 |
| **NC1** | + | 7923 | 8414 | 491 |  |  | 0 |
| **trnD** | + | 8415 | 8489 | 74 |  |  | 0 |
| **atp8** | + | 8461 | 8622 | 162 | ATA | TAA | -28 |
| **atp6** | + | 8616 | 9281 | 666 | ATG | TAA | -6 |
| **trnR** | + | 9331 | 9375 | 44 |  |  | 50 |
| **trnE** | + | 9409 | 9454 | 45 |  |  | 34 |
| **trnT** | + | 9455 | 9506 | 51 |  |  | 1 |
| **cob** | + | 9508 | 10641 | 1134 | ATC | TAG | 2 |
| **trnM** | + | 10652 | 10710 | 58 |  |  | 2 |
| **trnI** | + | 10706 | 10759 | 53 |  |  | 11 |
| **trnY** | - | 10765 | 10852 | 87 |  |  | 6 |
| **trnL1** | + | 10788 | 10848 | 60 |  |  | -64 |
| **trnV** | - | 10871 | 10939 | 68 |  |  | 23 |
| **NC2** | + | 10940 | 11331 | 391 |  |  | 0 |
| **trnS2** | + | 11332 | 11387 | 55 |  |  | 0 |
| **nad3** | + | 11382 | 11732 | 351 | ATT | TAA | -5 |
| **trnN** | + | 11752 | 11799 | 47 |  |  | 20 |
| **trnK** | - | 11852 | 11895 | 43 |  |  | 53 |
| **nad1** | - | 11942 | 12862 | 921 | ATT | TAG | 47 |
| **rrnL** | - | 12888 | 14258 | 1370 |  |  | 26 |
| **trnL2** | + | 14078 | 14148 | 70 |  |  | -180 |
| **rrnS** | - | 14111 | 14850 | 739 |  |  | -37 |
