# Supplementary material for: The First Myriapod Genome Sequence Reveals Conservative Arthropod Gene Content and Genome Organisation in the Centipede Strigamia maritima
Source: PLoS Biol. 2014 Nov 25;12(11):e1002005. doi: 10.1371/journal.pbio.1002005 (PMC4244043; doi:10.1371/journal.pbio.1002005)
Supplement: Table S12 — Species used in the synteny analyses and the sources of their sequence data. (DOCX) [file pbio.1002005.s046.docx]

| Species | Proteome build | File |
| --- | --- | --- |
| Arabidopsis thaliana | TAIR10.62 | ftp://ftp.ensemblgenomes.org/pub/plants/release-9/fasta/arabidopsis_thaliana/pep/Arabidopsis_thaliana.TAIR10.10.pep.all.fa.gz |
| Naegleria gruberi | Naegr1_best | ftp://ftp.jgi-psf.org/pub/JGI_data/Naegleria_gruberi/Naegr1_best_proteins.fasta.gz |
| Thalassiosira pseudonana | Thaps3_chromosomes_Filtered2 | ftp://ftp.jgi-psf.org/pub/JGI_data/Thalassiosira_pseudonana/v3.0/Thaps3_chromosomes_geneModels_FilteredModels2_aa.fasta.gz |
| Chlamydomonas reinhardtii | Chlre4_best | ftp://ftp.jgi-psf.org/pub/JGI_data/Chlamydomonas_reinhardtii/v4.0/annotation/Chlre4_best_proteins.fasta.gz |
| Phycomyces blakesleeanus | Phybl1_best | ftp://ftp.jgi-psf.org/pub/JGI_data/Phycomyces_blakesleeanus/annotation/v1.0/Phybl1_best_proteins.fasta.gz |
| Mucor circinelloides | Mucci1_best | ftp://ftp.jgi-psf.org/pub/JGI_data/Mucor_circinelloides/v1.0/annotation/Mucci1_best_proteins.fasta.gz |
| Schizosaccharomyces octosporus | Schizosaccharomyces_octosporus_protein | schizosaccharomyces_octosporus_6_proteins.fasta |
| Schizosaccharomyces cryophilus | Schizosaccharomyces_cryophilus_protein | schizosaccharomyces_cryophilus_4_proteins.fasta |
| Schizosaccharomyces pombe | Schizosaccharomyces_pombe_protein | schizosaccharomyces_pombe_972h-_2_proteins.fasta |
| Schizosaccharomyces japonicus | Schizosaccharomyces_japonicus_protein | schizosaccharomyces_japonicus_yfs275_5_proteins.fasta |
| Saccharomyces cerevisiae | EF2.62 | ftp://ftp.ensembl.org/pub/release-62/fasta/saccharomyces_cerevisiae/pep/Saccharomyces_cerevisiae.EF2.62.pep.all.fa.gz |
| Tremella mesenterica | Treme1_best | ftp://ftp.jgi-psf.org/pub/JGI_data/Tremella_mesenterica/v1.0/annotation/Treme1_best_proteins.fasta.gz |
| Batrachochytrium dendrobatidis | Batde5_best | ftp://ftp.jgi-psf.org/pub/JGI_data/Batrachochytrium_dendrobatidis/annotation/v1.0/Batde5_best_proteins.fasta.gz |
| Acropora digitifera | nomask_110621.TEremove | nomask_110621.prot.t1.TEremove.fa |
| Nematostella vectensis | Nemve1FilteredModels1 | ftp://ftp.jgi-psf.org/pub/JGI_data/Nematostella_vectensis/v1.0/annotation/proteins.Nemve1FilteredModels1.fasta.gz |
| Hydra magnipapillata | hydra_Hma2 | ftp://ftp.jgi-psf.org/pub/JGI_data/Hydra_magnipapillata/annotation/hydra_Hma2.pep.fa.gz |
| Capitella teleta | FilteredModelsv1.0 | ftp://ftp.jgi-psf.org/pub/JGI_data/Capitella/v1.0/FilteredModelsv1.0.aa.fasta.gz |
| Helobdella robusta | Helro1_FilteredModels3 | ftp://ftp.jgi-psf.org/pub/JGI_data/Helobdella_robusta/v1.0/proteins.Helro1_FilteredModels3.fasta.gz |
| Lottia gigantea | Lotgi1_GeneModels_FilteredModels1 | ftp://ftp.jgi-psf.org/pub/JGI_data/Lottia_gigantea/v1.0/Lotgi1_GeneModels_FilteredModels1_aa.fasta.gz |
| Schistosoma mansoni | sma_v3.1 | ftp://ftp.ensemblgenomes.org/pub/metazoa/release-9/fasta/schistosoma_mansoni/pep/Schistosoma_mansoni.sma_v3.1.1a.pep.all.fa.gz |
| Pristionchus pacificus | pp1.62 | ftp://ftp.ensemblgenomes.org/pub/metazoa/release-9/fasta/pristionchus_pacificus/pep/Pristionchus_pacificus.pp1.1a.pep.all.fa.gz |
| Caenorhabditis elegans | Caenorhabditis_elegans.WS220.220 | ftp://ftp.ensemblgenomes.org/pub/metazoa/release-9/fasta/caenorhabditis_elegans/pep/Caenorhabditis_elegans.WS220.220.pep.all.fa.gz |
| Drosophila grimshawi | dgri_r1.3_FB2008_07 | ftp://ftp.ensemblgenomes.org/pub/metazoa/release-11/fasta/drosophila_grimshawi/pep/Drosophila_grimshawi.dgri_r1.3_FB2008_07.pep.all.fa.gz |
| Drosophila melanogaster | BDGP5.25.62 | ftp://ftp.ensembl.org/pub/release-62/fasta/drosophila_melanogaster/pep/Drosophila_melanogaster.BDGP5.25.62.pep.all.fa.gz |
| Aedes aegypti | 2009-06-VectorBase | ftp://ftp.ensemblgenomes.org/pub/metazoa/release-11/fasta/aedes_aegypti/pep/Aedes_aegypti.AaegL1.pep.all.fa.gz |
| Culex quinquefasciatus | 2008-05-VectorBase | ftp://ftp.ensemblgenomes.org/pub/metazoa/release-11/fasta/culex_quinquefasciatus/pep/Culex_quinquefasciatus.CpipJ1.pep.all.fa.gz |
| Anopheles gambiae | AgamP3.6 | ftp://ftp.ensemblgenomes.org/pub/metazoa/release-11/fasta/anopheles_gambiae/pep/Anopheles_gambiae.AgamP3.pep.all.fa.gz |
| Bombyx mori | silkworm_glean_pep_v2.0 | silkpep.fa |
| Apis mellifera | 2005-BeeBase | ftp://ftp.ensemblgenomes.org/pub/metazoa/release-11/fasta/apis_mellifera/pep/Apis_mellifera.Amel_2.0.pep.all.fa.gz |
| Tribolium castaneum | 3.0_Tribolium_Official_Gene_sequences | ftp://bioinformatics.ksu.edu/pub/BeetleBase/3.0/Sequences/Tribolium_Official_Gene_Sequences/peptide.fa |
| Pediculus humanus | PhumU1.2 | ftp://ftp.ensemblgenomes.org/pub/metazoa/release-11/fasta/pediculus_humanus/pep/Pediculus_humanus.PhumU1.pep.all.fa.gz |
| Daphnia pulex | FilteredModelsv1.0 | ftp://ftp.jgi-psf.org/pub/JGI_data/Daphnia_pulex/v1.0/FilteredModelsv1.0.aa.fasta.gz |
| Strigamia maritima | Strigamia_6.1 | http://www.hgsc.bcm.tmc.edu/collaborations/insects/strigamia/Maker_results/centipede_maker_sept_2011/all_maker_protains.fa |
| Ixodes scapularis | IscaW1.62 | ftp://ftp.ensemblgenomes.org/pub/metazoa/release-9/fasta/ixodes_scapularis/pep/Ixodes_scapularis.IscaW1.1a.pep.all.fa.gz |
| Tetranychus urticae | PEP_20120618 | https://bioinformatics.psb.ugent.be/gdb/tetranychus/Tetur_PEP_20120618.tfa.gz |
| Strongylocentrotus purpuratus | SpBase_SPU | SpBase_SPU_peptide.fasta.gz |
| Saccoglossus kowalevskii | SkowalevskiiJGIv3.0 | ftp://ftp.jgi-psf.org/pub/JGI_data/Saccoglossus_kowalevskii/v3/annotation/SkowalevskiiJGIv3.0.longestTrs.pep.fa.gz |
| Gasterosteus aculeatus | Ensembl_64 | ftp://ftp.ensembl.org/pub/release-64/fasta/gasterosteus_aculeatus/pep/Gasterosteus_aculeatus.BROADS1.64.pep.all.fa.gz |
| Oryzias latipes | Ensembl_64 | ftp://ftp.ensembl.org/pub/release-64/fasta/oryzias_latipes/pep/Oryzias_latipes.MEDAKA1.64.pep.all.fa.gz |
| Danio rerio | Zv9.62 | ftp://ftp.ensembl.org/pub/release-62/fasta/danio_rerio/pep/Danio_rerio.Zv9.62.pep.all.fa.gz |
| Anolis carolinensis | Ensembl_64 | ftp://ftp.ensembl.org/pub/release-64/fasta/anolis_carolinensis/pep/Anolis_carolinensis.AnoCar2.0.64.pep.all.fa.gz |
| Gallus gallus | WASHUC2.62 | ftp://ftp.ensembl.org/pub/release-62/fasta/gallus_gallus/pep/Gallus_gallus.WASHUC2.62.pep.all.fa.gz |
| Homo sapiens | Homo_sapiens.GRCh37.61 | ftp://ftp.ensembl.org/pub/current/fasta/homo_sapiens/pep/Homo_sapiens.GRCh37.61.pep.all.fa.gz |
| Xenopus (Silurana) tropicalis | Ensembl_61 | ftp://ftp.ensembl.org/pub/current/fasta/xenopus_tropicalis/pep/Xenopus_tropicalis.JGI4.1.61.pep.all.fa.gz |
| Ciona intestinalis | Ciona_intestinalis.JGI2.52 | ftp://ftp.ensembl.org/pub/release-52/fasta/ciona_intestinalis/pep/Ciona_intestinalis.JGI2.52.pep.all.fa.gz |
| Branchiostoma floridae | Brafl1 | ftp://ftp.jgi-psf.org/pub/JGI_data/Branchiostoma_floridae/v1.0/proteins.Brafl1.fasta.gz |
| Trichoplax adhaerens | Triad1_best_proteins | ftp://ftp.jgi-psf.org/pub/JGI_data/Trichoplax_adhaerens_Grell-BS-1999/annotation/v1.0/Triad1_best_proteins.fasta.gz |
| Mnemiopsis leidyi | ML2.2 | ML2.2.aa |
| Amphimedon queenslandica | Aqu1 | ftp://ftp.jgi-psf.org/pub/JGI_data/Amphimedon_queenslandica/annotation/Aqu1.pep.fa.gz |
| Monosiga brevicollis | Monbr1_best_proteins | ftp://ftp.jgi-psf.org/pub/JGI_data/Monosiga_brevicollis/annotation/v1.0/Monbr1_best_proteins.fasta.gz |
| Capsaspora owczarzaki | capsaspora_owczarzaki_atcc_30864_2_proteins | capsaspora_owczarzaki_atcc_30864_2_proteins.fasta |
| Dictyostelium discoideum | created_03-15-2011 | dicty_primary_protein_20110315.gz |
