# Supplementary material for: The First Myriapod Genome Sequence Reveals Conservative Arthropod Gene Content and Genome Organisation in the Centipede Strigamia maritima
Source: PLoS Biol. 2014 Nov 25;12(11):e1002005. doi: 10.1371/journal.pbio.1002005 (PMC4244043; doi:10.1371/journal.pbio.1002005)
Supplement: Table S13 — Block-synteny summary statistics for pairs of species. Hs, Homo sapiens; Bf, B. floridae; Sm, S. maritima; Lg, Lottia gigantea; Ct, Capitella teleta; Nv, N. vectensis; Ta, Trichoplax adhaerens; Ag, Anopheles gambiae; Bm, B. mori. (DOCX) [file pbio.1002005.s047.docx]

| *P* | Hs | Bf | Sm | Lg | Ct | Nv | Ta | Aq | Bm |
| --- | --- | --- | --- | --- | --- | --- | --- | --- | --- |
| Hs |  | 59.9 | 58 | 54 | 51.4 | 50.8 | 52.1 | 42.6 | 39.9 |
| Bf |  |  | 64.8 | 60.5 | 54.9 | 57.3 | 57 | 48.6 | 42.9 |
| Sm |  |  |  | 59 | 60.1 | 54 | 56.6 | 47.2 | 47.4 |
| Lg |  |  |  |  | 52.4 | 46.2 | 57.7 | 45 | 40.7 |
| Ct |  |  |  |  |  | 49.9 | 50.3 | 41.1 | 38.2 |
| Nv |  |  |  |  |  |  | 56 | 47.1 | 35.2 |
| Ta |  |  |  |  |  |  |  | 48.3 | 36.3 |
| Aq |  |  |  |  |  |  |  |  | 34.1 |
| Bm |  |  |  |  |  |  |  |  |  |
