# Supplementary material for: The First Myriapod Genome Sequence Reveals Conservative Arthropod Gene Content and Genome Organisation in the Centipede Strigamia maritima
Source: PLoS Biol. 2014 Nov 25;12(11):e1002005. doi: 10.1371/journal.pbio.1002005 (PMC4244043; doi:10.1371/journal.pbio.1002005)
Supplement: Table S14 — Summary of numbers of homeobox genes per class of Strigamia , Branchiostoma , and Tribolium . (DOCX) [file pbio.1002005.s048.docx]

**Table S14. Summary of numbers of homeobox genes per class of *Strigamia*, *Branchiostoma* and *Tribolium*.**

| Homeobox class | *Strigamia maritima* | *Branchiostoma floridae* | *Tribolium castaneum* |
| --- | --- | --- | --- |
| ANTP | 54 | 60 | 45 |
| PRD | 26 | 28 | 25 |
| TALE | 8 | 9 | 8 |
| SINE | 3 | 3 | 3 |
| LIM | 6 | 7 | 7 |
| POU | 4 | 8 | 6 |
| HNF | 1 | 4 | 0 |
| CUT | 3 | 4 | 3 |
| PROS | 1 | 1 | 1 |
| ZF | 2 | 5 | 2 |
| CERS | 2 | 1 | 1 |
| others | 4 | 3 | 2 |
