# Supplementary material for: The First Myriapod Genome Sequence Reveals Conservative Arthropod Gene Content and Genome Organisation in the Centipede Strigamia maritima
Source: PLoS Biol. 2014 Nov 25;12(11):e1002005. doi: 10.1371/journal.pbio.1002005 (PMC4244043; doi:10.1371/journal.pbio.1002005)
Supplement: Table S16 — One-to-one S. maritima to human orthologues starting from genes on S. maritima scaffold 48457, which contains SmaHox3a . The third column is the chromosomal location of the human orthologue. Human Hox chromosomes are 2, 7, 12, and 17 and the ParaHox chromosomes are 4, 5, 13, and X. (DOCX) [file pbio.1002005.s050.docx]

**Table S16. One-to-one *S. maritima* to human orthologues starting from genes on *S. maritima* scaffold 48457, which contains *SmaHox3a*.**

| ***S. maritima* gene** | **Human gene** | **Human chromosome location** |
| --- | --- | --- |
| SMAR008046 | ENSP00000362704 | 1 |
| SMAR007985 | ENSP00000350967 | 18 |
| SMAR008065 | ENSP00000279206 | 11 |
| SMAR008014 | ENSP00000357973 | 6 |
| SMAR008000 | ENSP00000404030 | 2 |
| SMAR008072 | ENSP00000270517 | 19 |
| SMAR008026 | ENSP00000329137 | 1 |
| SMAR007995 | ENSP00000216862 | 20 |
| SMAR007986 | ENSP00000260983 | 2 |
| SMAR008066 | ENSP00000361236 | 6 |
| SMAR008018 | ENSP00000254190 | 15 |
| SMAR008023 | ENSP00000306340 | 4 |
| SMAR008004 | ENSP00000339918 | 11 |
| SMAR008073 | ENSP00000365014 | 9 |
| SMAR008048 | ENSP00000445955 | 12 |
| SMAR008058 | ENSP00000438978 | 22 |
| SMAR008029 | ENSP00000312397 | 5 |
| SMAR008013 | ENSP00000394071 | X |
| SMAR008051 | ENSP00000439188 | 7 |
| SMAR008009 | ENSP00000229270 | 12 |
| SMAR008017 | ENSP00000454828 | 15 |
| SMAR008024 | ENSP00000421488 | 4 |
| SMAR008019 | ENSP00000355877 | 1 |
| SMAR008008 | ENSP00000303525 | 4 |
