# Supplementary material for: The First Myriapod Genome Sequence Reveals Conservative Arthropod Gene Content and Genome Organisation in the Centipede Strigamia maritima
Source: PLoS Biol. 2014 Nov 25;12(11):e1002005. doi: 10.1371/journal.pbio.1002005 (PMC4244043; doi:10.1371/journal.pbio.1002005)
Supplement: Table S17 — Evolutionary conservation of RNA processing modes in the S. maritima and D. melanogaster Hox clusters. Type of RNA processing event concerning each one of the S. maritima (left) and D. melanogaster (right) Hox genes. We note that orthologous genes in both species undergo similar types of RNA processing: the three posterior-most Hox genes: Ubx, abd-a, and Abd-b display a specific type of APA (tandem APA) in both S. maritima and D. melanogaster (conserved patterns highlighted by red asterisks) providing an example of what might be a feature present in the ancestral Hox cluster to insects and myriapods. Nonetheless, for most other Hox genes, RNA processing patterns differ markedly between S. maritima and D. melanogaster, indicating that the conserved incidence of alternative RNA processing across arthropods can only be proposed for the posterior-most Hox genes. (PDF) [file pbio.1002005.s051.pdf]

Table S17. Evolutionary conservation of RNA processing modes in the *S. maritima* and *D. melanogaster* Hox clusters.

	Strigamia maritima			*Drosophila melanogaster*		
**Gene**	**Alternative Promoter Usage (APU)**	**Alternative Splicing (AS)**	**Alternative Polyadenylation (APA)**	**Alternative Promoter Usage (APU)**	**Alternative Splicing (AS)**	**Alternative Polyadenylation (APA)**
lab	No	No	Yes	Yes	No	No
pb	No	No	Yes	No	Yes	No
Dfd	Yes	Yes	Yes	No	No	No
Scr	No	No	No	Yes	No	No
ftz	Yes	Yes	No	No	No	No
Antp	No	No	No	Yes	No	Yes
Ubx	No	No	Yes*	No	Yes	Yes*
abd-A	No	No	Yes*	Yes	No	Yes*
Abd-B	No	No	Yes*	Yes	Yes	Yes*
