# Supplementary material for: The First Myriapod Genome Sequence Reveals Conservative Arthropod Gene Content and Genome Organisation in the Centipede Strigamia maritima
Source: PLoS Biol. 2014 Nov 25;12(11):e1002005. doi: 10.1371/journal.pbio.1002005 (PMC4244043; doi:10.1371/journal.pbio.1002005)
Supplement: Table S18 — Details of SmGr family genes and proteins. Columns are: Gene, the gene and protein name we are assigning (suffixes are PSE, pseudogene; FIX, assembly was repaired; JOI, gene model spans scaffolds); OGS, the official gene number in the 13,233 proteins (prefix is Smar_temp_); Scaffold, the genome assembly scaffold ID, prefix is scf718000 (amongst 14,739 scaffolds in assembly Smar05272011); Coordinates, the nucleotide range from the first position of the start codon to the last position of the stop codon in the scaffold; Strand – + is forward and − is reverse; introns, number of introns; ESTs, presence of an EST contig with appropriate splicing in one of the three transcriptome assemblies (F, female; M, male; E, eggs); AAs, number of encoded amino acids in the protein; comments, comments on the OGS gene model, repairs to the genome assembly, and pseudogene status (numbers in parentheses are the number of obvious pseudogenizing mutations). (DOC) [file pbio.1002005.s052.doc]

**Gene OGS Scaffold Coordinates Strand Introns ESTs AAs Comments**

Gr1PSE - 1246649 109524-110846 - 3 - 381 Pseudogene (1)

Gr2 - 1246649 107888-109256 - 3 - 393 New gene model

Gr3 - 1246649 106219-107595 - 3 - 391 New gene model

Gr4 - 1246649 104289-105628 - 3 - 388 New gene model

Gr5 - 1246649 100964-102309 - 3 - 387 New gene model

Gr6 - 1246649 95742-97115 - 3 - 395 New gene model

Gr7 - 1246649 94195-95522 - 3 - 386 New gene model

Gr8 - 1246649 90782-92159 - 3 - 401 New gene model

Gr9 - 1246649 89057-90447 - 3 - 394 New gene model

Gr10 002773 1246649 87150-88821 - 3 - 395 Multiple changes

Gr11 002772 1246649 79992-81394 - 3 E 397 Multiple changes

Gr12 - 1246649 78231-79594 - 3 - 397 New gene model

Gr13 002771 1246649 76640-78023 - 3 - 398 Multiple changes

Gr14 - 1248682 86030-87667 - 3 - 389 New gene model

Gr15PSE - 1248682 82213-83822 - 3 - 364 Pseudogene (2)

Gr16 - 1241819 24940-26301 - 3 - 394 New gene model

Gr17PSE - 1241819 37233-38572 + 3 - 374 Pseudogene (1)

Gr18 - 1241819 43368-46018 + 3 - 394 New gene model

Gr19 - 1241819 50175-51537 + 3 - 392 New gene model

Gr20 - 1247629 30015-31379 - 3 - 393 New gene model

Gr21 - 1246359 3677-4996 + 3 - 392 New gene model

Gr22 - 1248166 5063-6424 + 3 - 394 New gene model

Gr23PSE - 1239966 12314-13546 - 3 - 353 Pseudogene (2)

Gr24 - 1248616 10345-11719 - 3 - 393 New gene model

Gr25FIX - 1248616 5002-9784 - 3 - 391 Fix assembly

Gr26 - 1248423 32414-33896 + 3 - 426 New gene model

Gr27 - 1247860 21946-23334 - 3 - 396 New gene model

Gr28 011978 1248782 59128-60551 - 3 M 397 Fine as is

Gr29FIX - 1248782 50967-58406 - 3 - 407 Fix assembly

Gr30PSE - 1248782 54519-56100 - 3 - 400 Pseudogene (3)

Gr31 - 1248782 52606-54004 - 3 - 404 New gene model

Gr32 - 1248782 44091-45461 - 3 - 394 New gene model

Gr33 - 1248782 41349-42752 - 3 - 407 New gene model

Gr34PSE - 1248782 38810-39814 - 1 - 333 Pseudogene (4)

Gr35 - 1248782 32569-34016 - 3 - 417 New gene model

Gr36FIX - 1248782 30684-32234 - 3 - 397 Fix assembly

Gr37 - 1248782 28916-30338 - 3 - 410 New gene model

Gr38 - 1248782 26575-28044 - 3 - 417 New gene model

Gr39 - 1240936 229517-230924 - 3 - 405 New gene model

Gr40PSE - 1248616 103937-106051 + 2 - 360 Pseudogene (6)

Gr41 - 1248601 689597-691905 + 3 FE 407 New gene model

Gr42 - 1248773 524239-526716 - 3 - 395 New gene model

Gr43 011848 1248773 518823-520174 - 3 - 381 New gene model

Gr44 - 1248773 512799-515676 - 3 - 380 New gene model

Gr45PSE - 1248773 508527-510145 - 3 - 347 Pseudogene (5)

Gr46 - 1248773 503467-506688 - 3 - 380 New gene model

Gr47 - 1248773 495497-497856 - 3 - 382 New gene model

Gr48 - 1247874 5279-6584 - 3 - 378 New gene model

Gr49 - 1247874 2036-3366 - 3 - 383 New gene model

Gr50PSE - 1248428 777-4992 + 3 - 377 Pseudogene (5)

Gr51FIX - 1248428 6178-7567 + 3 - 379 Fix assembly

Gr52 - 1248428 8770-10048 - 2 - 382 New gene model

Gr53 - 1245773 11938-14084 + 3 - 390 New gene model

Gr54PSE - 1245773 14409-15468 + 3 - 268 Pseudogene (4)

Gr55 - 1245773 16996-19582 + 3 - 395 New gene model

Gr56 - 1245773 21470-22902 + 3 - 399 New gene model

Gr57 - 1245773 23623-25115 + 3 - 404 New gene model

Gr58 002542 1245773 26516-28547 + 3 - 402 Multiple changes

Gr59PSE - 1239276 376-1786 + 3 - 402 Pseudogene (1)

Gr60 - 1239276 5103-6451 + 3 - 387 New gene model

Gr61 - 1239276 7567-9030 + 3 - 395 New gene model

Gr62PSE - 1239276 11366-12623 + 2 - 370 Pseudogene (2)

Gr63FIX - 1237055 9157-10859 - 3 - 409 Fix assembly

Gr64 - 1247276 39454-41190 + 3 - 404 New gene model

Gr65 - 1247276 49963-51280 - 3 FME 373 New gene model

Gr66 003590 1247276 59991-61345 + 3 - 388 Multiple changes

Gr67FIX - 1247276 61991-63318 + 3 - 373 Fix assembly

Gr68JOI - 1247276 65190->66206 + 3 - 373 Join across scaffolds

1237410 1080->1209 -

Gr69PSE 011296 1248711 329-2202 + 3 M 373 Pseudogene (1)

Gr70 - 1247197 134081-135504 - 3 M 396 New gene model

Gr71 - 1247197 136787-138135 + 3 - 380 New gene model

Gr72 - 1247197 139864-141254 + 3 - 396 New gene model

Gr73 - 1236518 19788-21242 - 3 - 363 New gene model

Gr74 - 1248001 37879-39189 - 3 M 372 New gene model

Gr75 - 1239013 20148-21599 - 3 M 390 New gene model

Gr76FIX 009827 1248602 47739-50740 - 4 FME 421 Fix assembly
