# Supplementary material for: The First Myriapod Genome Sequence Reveals Conservative Arthropod Gene Content and Genome Organisation in the Centipede Strigamia maritima
Source: PLoS Biol. 2014 Nov 25;12(11):e1002005. doi: 10.1371/journal.pbio.1002005 (PMC4244043; doi:10.1371/journal.pbio.1002005)
Supplement: Table S19 — Total numbers of biogenic amine receptors in different species. (DOCX) [file pbio.1002005.s053.docx]

**Table S19. Numbers of biogenic amine GPCRs in a selection of different animals with a sequenced genome.**

| **Species** | **Number** |
| --- | --- |
| *Danio reiro* | 122^1^ |
| *Mus musculus* | 57^1^ |
| *Homo sapiens* | 44^1^ |
| *Branchiostoma floridae* | 44^1^ |
| *Takifugu rubripes* | 28^1^ |
| *Ciona intestinalis* | 23^1^ |
| ***Drosophila melanogaster*** | 22^1^ |
| *Caenorhabditis elegans* | 20^1^ |
| ***Tribolium castaneum*** | 20^1^ |
| ***Apis mellifera*** | 19^1^ |
| ***Anopheles gambiae*** | 18^1^ |
| ***Strigamia maritima*** | 19 |

^1^adapted from [92 and 93]. Arthropod species in bold.
