# Supplementary material for: The First Myriapod Genome Sequence Reveals Conservative Arthropod Gene Content and Genome Organisation in the Centipede Strigamia maritima
Source: PLoS Biol. 2014 Nov 25;12(11):e1002005. doi: 10.1371/journal.pbio.1002005 (PMC4244043; doi:10.1371/journal.pbio.1002005)
Supplement: Table S22 — Presence or absence of neuropeptide signaling systems in arthropods. The centipede S. maritima contains two CCHamide-1, two eclosion hormone and two FMRFamide genes (2 p). In some cases neuropeptide precursors could not be identified, but the corresponding receptor genes are present (R). We assume that this is due to sequencing gaps. For abbreviations see Table S21. (DOC) [file pbio.1002005.s056.doc]

**Table S22.** **Presence or absence of neuropeptide signaling systems in arthropods.** The centipede *S. maritima* contains two CCHamide-1, two eclosion hormone and two FMRFamide genes (2 p). In some cases, neuropeptide precursors could not be identified, but the corresponding receptor genes are present (R). We assume that this is due to sequencing gaps. Abbreviations: ACP, adipokinetic hormone/corazonin-related neuropeptide; AKH, adipokinetic hormone; ADF, antidiuretic factor; AST, allatostatin; CCAP, crustacean cardio-active peptides; DH (Calc.-like), calcitonin-like diuretic hormone; DH (CRF-like), corticotropin releasing factor-like diuretic hormone; EH, eclosion hormone; ETH, ecdysis triggering hormone; GPA2, glycoprotein hormone A2; GPB5, glycoprotein hormone B5; ILP, insulin-like peptide; ITP, ion transport peptide; NPF, neuropeptide F; NPLP, neuropeptide-like precursor; PDF, pigment dispersing factor; PTTH, prothoracicotropic hormone; sNPF, short neuropeptide F.

| **neuropeptide** | ***Drosophila***  ***melanogaster*** | ***Aedes***  ***aegypti*** | ***Bombyx***  ***mori*** | ***Tribolium***  ***castaneum*** | | ***Acro-***  ***myrmex***  ***echinatior*** | | ***Apis***  ***mellifera*** | ***Nasonia***  ***vitripennis*** | ***Acyrtho-siphon***  ***pisum*** | ***Daphnia***  ***pulex*** | ***Strigamia***  ***maritima*** | ***Ixodes***  ***scapularis*** |
| --- | --- | --- | --- | --- | --- | --- | --- | --- | --- | --- | --- | --- | --- |
|  |  |  |  |  |  | |  | |  |  |  |  |  |
| ACP | no | yes | yes | yes | no | | no | | yes | yes | no | yes | yes |
| Achatin | no | no | no | no | no | | no | | no | no | no | no | yes |
| AKH | yes | yes | yes (2 p) | yes (2 p) | yes | | yes | | yes | yes | yes | yes | no |
| ADF-b | no | no | no | yes | no | | no | | no | no | no | no | no |
| Allatotropin | no | yes | yes | yes | yes | | yes | | yes | yes | yes | yes | yes |
| AST-A | yes | yes | yes | no | yes | | yes | | yes | yes | yes | yes | yes |
| AST-B | yes | yes | yes | yes | no | | no | | no | yes | yes | yes | yes |
| AST-C | yes | yes | yes | yes | yes | | yes | | yes | yes | yes (2 p) | yes | yes |
| AST-CC | yes | yes | yes | yes | yes | | yes | | yes | yes | yes | yes | yes |
| Busricon-α | yes | yes | yes | yes | yes | | yes | | yes | yes | yes | yes | yes |
| Bursicon-β | yes | yes | yes | yes | yes | | yes | | yes | yes | yes | yes | yes |
| Capa | yes | yes | yes | yes | yes | | yes | | yes? (R) | yes | no | no | no |
| CCHamide-1 | yes | yes | yes | yes | yes | | yes | | yes | yes | yes | yes (2 p) | yes |
| CCHamide-2 | yes | yes | yes | yes | yes | | yes | | yes | yes | no | no | no |
| CCAP | yes | yes | yes | yes | yes | | yes | | yes | yes | yes | yes | yes |
| Corazonin | yes | yes | yes | no | yes | | yes | | yes | no | yes | yes | yes |
| DENamide | no | no | no | no | no | | no | | no | no | yes | no | no |
| DH (Calc.-like) | yes | yes | yes | yes | yes | | yes | | yes | yes | yes | yes | yes |
| DH (CRF-like) | yes | yes | yes | yes | yes | | yes | | yes | yes | yes | yes | yes |
| EFLamide | no | no | no | no | no | | no | | no | no | yes | yes | yes |
| Elevenin | no | no | no | no | yes | | yes | | no | no | yes | yes | no |
| EH | yes | yes (5 p) | yes | yes | yes | | yes | | yes | yes (3 p) | yes (2 p) | yes (2 p) | yes |
| **Table SN4** (continued) |  |  |  |  | |  | |  |  |  |  |  |  |
| **neuropeptide** | ***Drosophila***  ***melanogaster*** | ***Aedes***  ***aegypti*** | ***Bombyx***  ***mori*** | ***Tribolium***  ***castaneum*** | | ***Acro-***  ***myrmex***  ***echinatior*** | | ***Apis***  ***mellifera*** | ***Nasonia***  ***vitripennis*** | ***Acyrtho-siphon***  ***pisum*** | ***Daphnia***  ***pulex*** | ***Strigamia***  ***maritima*** | ***Ixodes***  ***scapularis*** |
|  |  |  |  |  |  | |  | |  |  |  |  |  |
| ETH | yes | yes | yes | yes | yes | | yes | | yes | yes | yes (2 p) | yes | no |
| FMRFa | yes | yes | yes | yes | no | | yes | | yes? (R) | yes | yes (2 p) | yes (2 p) | no |
| GPA2 | yes | yes | yes | yes | no | | no | | no | yes | yes | no | yes |
| GPB5 | yes | yes | yes | yes | no | | no | | no | yes | yes | no | yes |
| ILP-A | yes | yes | no | yes | no | | no | | no | no | no | no | no |
| ILP-B | yes (5 p) | yes (6 p) | yes (38 p) | yes (2 p) | yes | | yes | | yes | yes (7 p) | yes | yes | yes |
| ILP-C | yes | yes | no | yes | yes | | yes | | yes | yes | no | no | no |
| Inotocin | no | no | no | yes | yes | | no | | yes | no | yes | yes | yes |
| ITP | yes | yes | yes | yes | yes | | yes | | yes | yes | yes (2 p) | yes | yes |
| Kinin | yes | yes | yes | no | no | | yes | | no | yes | no | no | yes |
| Myosuppressin | yes | yes | yes | yes | yes | | yes | | yes | yes | yes | yes | no |
| Neuroparsin | no | yes | no | yes | yes | | yes | | yes | no | yes | no | no |
| NPF | yes | yes | yes | yes | yes | | yes | | yes | yes | yes | yes | no |
| NPLP-1 | yes | yes | yes | yes | yes | | yes | | no | yes | no | yes | no |
| Orcokinin | no | yes | yes | yes | yes | | yes | | yes | yes | yes (2 p) | no | yes |
| PDF | yes | yes | yes | yes? (R) | yes | | yes | | yes | no | yes | no | no |
| Proctolin | yes | no | no | yes | no | | no | | no | yes | yes (2 p) | yes | yes |
| PTTH | yes | yes | yes | yes | yes | | no | | yes | yes | no | no | no |
| Pyrokinin | yes | yes | yes | yes | yes | | yes | | yes | yes | no | yes | yes |
| RYamide | yes | yes | yes | yes | no | | yes | | yes | yes | yes | yes | yes |
| Sex peptide | yes | no | no | no | no | | no | | no | no | no | no | no |
| SIFamide | yes | yes | yes (2 p) | yes | yes | | yes | | yes | yes | yes | yes | yes |
| sNPF | yes | yes | yes | yes | yes | | yes | | yes | yes | yes | yes | yes |
| Sulfakinin | yes | yes | yes | yes | yes | | yes | | no | no | yes | yes | yes |
| Tachykinin | yes | yes | yes | yes | yes | | yes | | yes | yes | yes | yes | yes |
| Trissin | yes | yes | yes | yes | yes | | no | | no | no | no | no | yes |
