# Supplementary material for: The First Myriapod Genome Sequence Reveals Conservative Arthropod Gene Content and Genome Organisation in the Centipede Strigamia maritima
Source: PLoS Biol. 2014 Nov 25;12(11):e1002005. doi: 10.1371/journal.pbio.1002005 (PMC4244043; doi:10.1371/journal.pbio.1002005)
Supplement: Table S23 — Genes commonly implicated in arthropod juvenoids biosynthesis (green) and degradation (blue), and their potential regulators (purple) [98]–[101]. Common abbreviations, and presence in the centipede S. maritima. (DOCX) [file pbio.1002005.s057.docx]

| **Gene name** | **Abbreviation** | ***Strigamia*** |
| --- | --- | --- |
| Juvenile hormone acid methyltransferase | **JHAMT** | 3 copies |
| Cytosolic juvenile hormone binding protein | **JHBP** | 1 copy |
| Juvenile hormone esterase | **JHE** | 2 copies |
| Juvenile hormone esterase binding protein | **JHEBP** | 1 copy |
| Juvenile hormone epoxide hydrolase | **JHEH** | 3 copies |
| Allatostatin-A, -B, -C | **Ast-A, Ast-B, Ast-C** | 1 copy each |
| Allatostatin-A, -B, -C receptor | **Ast-A-R, Ast-B-R, Ast-C-R** | 1 copy each |
| Methoprene-tolerant | **Met** | 1 copy |
| Retinoid X receptor (ultraspiracle) | **Usp** | 1 copy, 1 pseudogene |

Biosynthetic and degradation pathway components Canonical regulatory components as shown in other arthropods
