# Supplementary material for: The First Myriapod Genome Sequence Reveals Conservative Arthropod Gene Content and Genome Organisation in the Centipede Strigamia maritima
Source: PLoS Biol. 2014 Nov 25;12(11):e1002005. doi: 10.1371/journal.pbio.1002005 (PMC4244043; doi:10.1371/journal.pbio.1002005)
Supplement: Table S24 — List of genes commonly implicated as potential regulators of arthropod juvenoids biosynthesis (purple) [98]–[101]. Common abbreviations, and presence in the centipede S. maritima. (DOCX) [file pbio.1002005.s058.docx]

| **Gene name** | **Abbreviation** | ***Strigamia*** | **Role** |
| --- | --- | --- | --- |
| Broad | **Br** | Not in assembly | Target of Met^98^ |
| Chd64 | **Chd64** | 2 copies | JH Response Element Binding Protein^99^ |
| FKBP39 | **FKBP39** | 1 copy | JH Response Element Binding Protein^99^ |
| Hexamarin | **Hex** | Not in assembly | Regulator of JH^100^ |
| Crustacean hyperglycemic hormone/  Ion transport peptide | **ITP** | 1 copy | Potential Regulator of MF^101^ |

Other potential regulatory components not mentioned in the text
