# Supplementary material for: The First Myriapod Genome Sequence Reveals Conservative Arthropod Gene Content and Genome Organisation in the Centipede Strigamia maritima
Source: PLoS Biol. 2014 Nov 25;12(11):e1002005. doi: 10.1371/journal.pbio.1002005 (PMC4244043; doi:10.1371/journal.pbio.1002005)
Supplement: Table S25 — Wnt genes in the genome of S. maritima . SMAR, the gene identification number, and scaffold, the scaffold identification number. Wnt 1, 6, and 10 are clustered together on the same scaffold (yellow highlighting), which is likely a remnant of the ancestral wnt gene cluster (see text for details). (PDF) [file pbio.1002005.s059.pdf]

Wnt	SMAR	Scaffold	Orientation	exact position
1	SMAR002920	JH431262	-	JH431262:1251-10636
2	SMAR004376	JH431477	+	JH431477:348546-351888
3	absent			
4	SMAR013747	JH431581		JH431581:51660-57306
5	SMAR008487	JH431845		JH431845:98065-103552
6	SMAR002921	JH431262	+	JH431262:10405-17870
7a<br><br>7b (partial)	SMAR012534<br><br>SMAR004621	JH432147<br><br>JH431512		JH432147:616902-620452<br><br>JH431512:2960-3358
8	absent			
9	SMAR014558	JH431549		JH431549:26-4967
10	SMAR002922	JH431262	+	JH431262:18145-23485
11<br><br>11	SMAR013488<br><br>SMAR015633	JH432107<br><br>JH432107		JH432107:26645-29318<br><br>JH432107:28062-29318
16<br><br>16-like1<br><br>16-like2 (partial)	SMAR001019<br><br>SMAR004315<br><br>SMAR001022	 <br>JH431466 		AFFK01014592:25825-27850<br><br>JH431466:16092-19535<br><br>AFFK01014592:59416-61453
A	SMAR015773	JH431250		JH431250:56204-61501
