# Supplementary material for: The First Myriapod Genome Sequence Reveals Conservative Arthropod Gene Content and Genome Organisation in the Centipede Strigamia maritima
Source: PLoS Biol. 2014 Nov 25;12(11):e1002005. doi: 10.1371/journal.pbio.1002005 (PMC4244043; doi:10.1371/journal.pbio.1002005)
Supplement: Table S26 — Selenoproteins in the S. maritima genome. (DOCX) [file pbio.1002005.s060.docx]

| **Symbol** | **Gene Name** |
| --- | --- |
| SPS2 | Selenophosphate Synthetase 2 |
| GPx1 | Glutathione Peroxidase 1 |
| GPx3 | Plasma Glutathione Peroxidase 3 |
| GPx4 | Phospholipid Glutathione Peroxidase 4 |
| TrxR1 | Thioredoxin Reductase 1 |
| TrxR2 | Thioredoxin Reductase 2 |
| MsrA | Methionine-S-Sulfoxide Reductase A |
| Sel15 | Selenoprotein 15 |
| SelM | Selenoprotein M |
| SelR | Selenoprotein R – Methionine-R-Sulfoxide Reductase B |
| SelT | Selenoprotein T |
| SelT2 | Selenoprotein T2 |
| SelU | Selenoprotein U |
| SelW2A | Selenoprotein W2-A |
| SelW2B | Selenoprotein W2-B |
| SelP | Selenoprotein P |
| SelK | Selenoprotein K |
| SelS | Selenoprotein S |
| SelO1 | Selenoprotein O-1 |
| SelO2 | Selenoprotein O-2 |

**Table S26. Selenoproteins in the *S. maritima* genome.**
