# Supplementary material for: The First Myriapod Genome Sequence Reveals Conservative Arthropod Gene Content and Genome Organisation in the Centipede Strigamia maritima
Source: PLoS Biol. 2014 Nov 25;12(11):e1002005. doi: 10.1371/journal.pbio.1002005 (PMC4244043; doi:10.1371/journal.pbio.1002005)
Supplement: Table S27 — Histone encoding loci of S. maritima . (DOCX) [file pbio.1002005.s061.docx]

| ***Gene ID*** | ***Symbol*** | ***Scaffold (Smar1)*** |
| --- | --- | --- |
| ***Histone H1 class*** |  |  |
| Smar000113 | H1.1 | JH429682 |
| Smar000668 | H1.2 | AFFK01014262 |
| Smar004517 | H1.3 | JH431494 |
|  |  |  |
| ***Histone H2A class*** |  |  |
| Smar004837 | H2A.1 | JH431541 |
| Smar000115 | H2A.2 | JH429682 |
| Smar009589 | H2A.3 | JH431924 |
| Smar000381 | H2A.4 | JH429986 |
| Smar000329 | H2A.5 | JH429928 |
| Smar000009 | H2A.6 | JH429551 |
| Smar001636 | H2A.X | JH430694 |
|  |  |  |
| ***Histone H2B class*** |  |  |
| Smar000117 | H2B.1 | JH429682 |
| Smar008027 | H2B.2 | JH431820 |
| Smar000438 | H2B.3 | JH430028 |
| Smar009006 | H2B.4 | JH431868 |
| Smar010158 | H2B.5 | JH431968 |
| Smar000445 | H2B.6 | JH430028 |
| Smar002154 | H2B.7 | JH431045 |
| Smar008069 | H2B.8 | JH431820 |
| Smar000443 | H2B.9 | JH430028 |
| Smar008469 | H2B.10 | JH431844 |
| Smar008470 | H2B.11 | JH431844 |
| Smar005958 | H2B.12 | AFFK01020108 |
| Smar005959 | H2B.13 | AFFK01020108 |
| Smar012570 | H2B.14 | JH432159 |
| Smar015655 | H2B.15 | JH429682 |
|  |  |  |
| ***Histone H3 class*** |  |  |
| Smar000116 | H3.1 | JH429682 |
| Smar006372 | H3.2 | JH431694 |
| Smar009941 | H3.3 | JH431952 |
| Smar006373 | H3.4 | JH431694 |
|  |  |  |
| ***Histone H4 class*** |  |  |
| Smar010742 | H4.1 | JH431998 |
| Smar000114 | H4.2 | JH429682 |
| Smar008052 | H4.3 | JH431820 |
| Smar008039 | H4.4 | JH431820 |

**Table S27. Histone encoding loci of *S. maritima*.**
