# Supplementary material for: The First Myriapod Genome Sequence Reveals Conservative Arthropod Gene Content and Genome Organisation in the Centipede Strigamia maritima
Source: PLoS Biol. 2014 Nov 25;12(11):e1002005. doi: 10.1371/journal.pbio.1002005 (PMC4244043; doi:10.1371/journal.pbio.1002005)
Supplement: Table S28 — Number of loci within the genomes of arthropod species encoding the five classes of histones. Orthologues for A. aegypti, D. pulex, T. urticae, and I. scapularis were obtained by BLAST analysis. Orthologues for A. mellifera and A. pisum were obtained from published literature [108],[109]. (DOCX) [file pbio.1002005.s062.docx]

|  | *Aedes aegypti* | *Apis mellifera* | *Acyrthosiphon pisum* | *Daphnia pulex* | *Tetranychus urticae* | *Ixodes scapularis* | ***Strigamia maritima*** |
| --- | --- | --- | --- | --- | --- | --- | --- |
| H1 | 6 | 2 | 6 | 5 | 1 | 4 | **3** |
| H2A | 19 | 6 | 5 | 10 | 4 | 6 | **7** |
| H2B | 11 | 5 | 5 | 12 | 7 | 4 | **15** |
| H3 | 18 | 6 | 7 | 10 | 6 | 4 | **4** |
| H4 | 15 | 4 | 5 | 6 | 3 | 1 | **4** |

**Table S28. Number of loci within the genomes of arthropod species encoding the five classes of histones.**
