# Supplementary material for: The First Myriapod Genome Sequence Reveals Conservative Arthropod Gene Content and Genome Organisation in the Centipede Strigamia maritima
Source: PLoS Biol. 2014 Nov 25;12(11):e1002005. doi: 10.1371/journal.pbio.1002005 (PMC4244043; doi:10.1371/journal.pbio.1002005)
Supplement: Table S29 — Germ line and RNAi genes annotated in the S. maritima genome. The name of the Drosophila orthologue is shown unless indicated with “(Mo),” for mouse. (DOCX) [file pbio.1002005.s063.docx]

| **Gene name** | **Number of putative *S. maritima* orthologues** | **ENSEMBL SMAR ID#** |
| --- | --- | --- |
| *Argonaute 1* | 1 | SMAR007228 |
| *Argonaute 2* | 2 | SMAR010593 &  Scaffold JH431691: 41,476-47,800 |
| *Argonaute 3* | 1 | SMAR005944 |
| *aubergine* | 0 | no orthologue |
| *barentz* | 1 | SMAR013358 |
| *Blimp-1 (Mo)* | 1 | SMAR011793 |
| *c-Myc* | 0 | no orthologue |
| *Capsuleen (PRMT5)* | 1 | SMAR013508 |
| *Dicer-1* | 1 | SMAR007746 |
| *Dicer-2* | 1 | SMAR009083 |
| *fear of intimacy* | 0 | no orthologue |
| *germ cell less* | 1 | SMAR006000 |
| *HMG Coenzyme A reductase* | 1 | SMAR006801 |
| *Lasp* | 1 | SMAR012805 |
| *lin-28* | 1 | SMAR011791 |
| *loquacious* | 1 | SMAR009997 |
| *maelstrom* | 1 | SMAR005907 |
| *mago nashi* | 1 | SMAR008803 |
| *mus209 (PCNA)* | 1 | SMAR013089 |
| *nanos* | 2 | SMAR012913  SMAR000830 |
| *oskar* | 0 | no orthologue |
| *piwi* | 3 | SMAR003821  SMAR003822  SMAR015088 |
| *PL10/belle* | 1 | SMAR005518 |
| *pumilio* | 1 | SMAR007422 |
| *spalt major* | 1 | SMAR008725 |
| *staufen* | 1 | SMAR004496 |
| *Trapped in endoderm 1* | 1 | SMAR012626 |
| *Tropomyosin* | 1 | SMAR012691 |
| *tudor* | 1 | SMAR006920 |
| *valois* | 0 | no orthologue |
| *vasa* | 1 | SMAR015390 |
| *Stella (Mo)* | 0 | no orthologue |

**Table S29. Germ line and RNAi genes annotated in the *S. maritima* genome.**
