# Supplementary material for: The First Myriapod Genome Sequence Reveals Conservative Arthropod Gene Content and Genome Organisation in the Centipede Strigamia maritima
Source: PLoS Biol. 2014 Nov 25;12(11):e1002005. doi: 10.1371/journal.pbio.1002005 (PMC4244043; doi:10.1371/journal.pbio.1002005)
Supplement: Text S1 — Supporting Methods Text. (DOCX) [file pbio.1002005.s072.docx]

**Supporting Information index**

**1. Supplemental Material and Methods**

**2. Repetitive element isolation and classification.**

**3. Phylome analysis and phylogenomics.**

**4. Synteny methodology.**

**5. Homeobox genes.**

**6. Non-homeobox gene clusters: innexin, Runt, E(spl)-C.**

**7. Chemosensation: Gustatory receptors (GRs).**

**8. Developmental signalling systems.**

**9. Histones and Histone modifying enzymes in *S. maritima.***

**10. Germ line genes.**

**11. Meiosis genes.**

**12. CpG methylation.**

**13. Non-protein-coding RNA genes in the *S. maritima* genome.**

**14**. **mRNA purification and sequencing library construction.**

**15. References.**

**16. Supporting Information Figure Legends.**

**17. Supporting Information Table Legends.**

**18. Supporting Information data files.**

**Supporting Information**

## 1. Supplemental Material and Methods

*Genome sequencing and annotation*

*S. maritima* raw sequence, and assembled genome sequence data is available at the NCBI under bioproject [PRJNA20501](http://www.ncbi.nlm.nih.gov/bioproject/PRJNA20501), Assembly ID GCA_000239455.1. The genome was sequenced using 454 sequencing technology. Three whole genome shotgun libraries were used to produce the data, a 454 Titanium fragment library (made from DNA isolated from a single male) and 454 paired end libraries with targets of 3kb and 8 kb mate pair insert sizes (made from DNA from ~20 pooled individuals of both sexes). About 22.2 million reads were assembled, representing about 8,190 Mb of sequence and about 45.5x coverage of the *S. maritima* genome.

The genome was assembled using the CABOG Celera assembler (Celera 6.1) to yield a total of 173.6 MB of assembled sequence, to yield genome release Smar 1.0. Illumina data was used to correct homo-polymer errors originating from the 454 data. This assembly comprises 24,087 contiguous sequence fragments (contigs), with an N50 size of 24.7kb, linked by the paired end reads into 14,745 scaffolds with an N50 size of 139.4 kb. (The N50 size is the length such that 50% of the assembled genome lies in blocks of the N50 size or longer.) When the estimated gaps between contigs in scaffolds are included, the total span of the assembly is 176.2 Mb. This assembled sequence omits many repeat sequences, which probably account for the difference between the assembly length and the prior genome size estimate based on feulgen image analysis densitometry, of 290Mb [1]. In fact approximately 42% of the input reads remained un-assembled, which on a raw data basis would predict a total of 58% of the genome or ~168Mb would be accounted for in the assembly, close to our actual assembly size of 176.2Mb.

*Population sequencing*

We sequenced at 25X coverage four individuals (females A, B and D – SRA accessions SRX326837, SRX326839 and SRX326840 respectively – and male J, accession SRX326841) collected in Brora, northern Scotland June 2009, and subsequently starved for 30 days. DNA was extracted from whole individuals using Qiagen Genomic DNA extraction kit. Sequence was generated on the Illumina GAII and HiSeq platforms. Paired 95bp reads were aligned to Smar1.0 using BWA, indels were locally realigned using GATK. SNPS and indels were called using the GATK unified genotyper with standard parameters. SNP density was calculated using VCF tools using the –SNPdensity option.

*RNA sequencing*

Total RNA was isolated from adult males and females collected and treated as in the previous paragraph, and liquid nitrogen frozen mixed eggs (107) from 7 clutches, collected during the 2006, 2007 and 2009 collections, using Qiagen mini RNA extraction kit. mRNA was purified from total RNA using Dynabeads® mRNA Purification Kit, first strand cDNA was reverse transcribed from poly-A mRNA using random hexamer and SuperScript® First-Strand Synthesis kit. The second strand cDNA was synthesized using DNA polymerase I and purified with 1.8X Agencourt AMPure XR beads. Double stranded cDNA was constructed into Illumina paired-end libraries, and assembled using Bowtie/Tophat.

*Gene Annotation*

A first pass automated annotation was generated via 3 iterations of a modified version of the Maker2 annotation pipeline [2], using ab initio gene prediction, protein homology (using as evidence the entire UniProt Metazoa database) and mapped EST evidence from the assembled transcriptomes with ab initio re-training between each iteration. This yielded 13,233 putative gene models. 1,377 of these automated gene models were manually checked and annotated. This identified very few assembly errors, but a small number of sequence errors (largely in homopolymer runs). However, in a significant number of cases, the automated annotation had fused adjacent genes, largely on the basis of confounding RNASeq evidence.

To avoid such gene model merging, greedy extension was used to cluster the BLAST alignments to the entire UniProt Metazoa protein database into discrete genomic loci. Putatively merged RNASeq-derived transcripts were then identified as those that spanned multiple protein clusters. For these protein-cluster-spanning transcripts, we examined the original Bowtie/Tophat derived splice-junction mappings for the presence of poorly supported splice-junctions (using read-coverage as a measure). Where identified, such transcripts were split, in some cases losing 5’ and 3’ UTR information. The resulting transcripts were used to re-predict the gene set using the same modified Maker2 pipeline as described above, yielding 14,911 putative gene models. Manual annotation, quality control and tracking was performed by uploading new submissions to a centralised instance of the VectorBase Community Annotation Pipeline (CAP) system [3]. These were subsequently integrated into the new annotation set using the VectorBase Patch-build system [3] to yield a final gene set of 14,992 gene models of which 1,095 had been subjected to manual reappraisal. To allow for gene identifier consistency with the original gene set release the Ensembl stable identifier pipeline was used to allocate identifiers for the final gene set.

A notable contaminant identified in the original assembly was ribosomal RNA sequence closely similar to that of nematodes in the genus *Pristionchus*, which are known arthropod parasites. No single-copy genes from this nematode were identified, suggesting the abundance of the contamination is low, and only the multi-copy rRNA genes had enough sequence to assemble. Scaffolds containing identified contaminants were removed from the annotated assembly, but it remains possible that some sequences of nematode origin remain.

The final gene-set contains 14,992 coding genes, 1,202 non-coding genes, 16,215 transcripts, and is available from the Ensembl Metazoa website: <http://metazoa.ensembl.org/Strigamia_maritima/Info/Index> . To assess the completeness of gene recovery we looked for “core” genes identified by [4]. Comparing these models to our gene models we identify 95.1% of the CEGMA core genes, and at the bp level we have a median and mean sensitivity of 0.99 and 0.94 respectively (coding bases of CEGMA core genes overlapping Smar gene models) and median and mean specificity of 0.99 and 0.87 respectively. Additionally, 14,090 gene models (89.9%) have 10 or more overlapping RNAseq reads from the three tissues (adult male and female and mixed sex embryos) we used to support the annotation (the percentage increases to 93.4% if you require only 1 or more RNAseq reads). This annotated genome was then used to deduce the *S. maritima* phylome as well as phylogenomic analyses as described below.

**2. Repetitive element isolation and classification.**

*Methodology*

We aimed to detect and annotate repetitive elements in the assembled portion of the genome, and have left the likely repetitive heterochromatic 40% of the genome that could not be assembled for future work. Thus the analysis and results described here are for the assembled genome only. Repetitive elements were detected and annotated with the REPET software package ([5], version 2.0) consisting of two pipelines integrating a set of bioinformatics programs. First, repeated sequences were detected by similarity (all-by-all BLAST using BLASTER) and LTR retrotransposons were detected by structural search (LTRharvest). The similarity matches were clustered with GROUPER, RECON and PILER, and the structural matches with single-linkage NCBI Blastclust. From each cluster a consensus sequence is generated by multiple alignment with Map. The consensus sequences were analyzed for terminal repeats (TRsearch), tandem repeats (TRF), open reading frames (dbORF.py, REPET) and poly-A tails (polyAtail, REPET). In addition, the consensuses were screened for matches to nucleotide and amino acid sequences from known transposable elements (RepBase 17.01, [6]) using BLASTER (tblastx, blastx) as well as searched for HMM profiles (Pfam database 26.0, [7]) using hmmer3. Based on the detected structural features and homologies, the consensuses are classified by PASTEC according to [8]. Redundancies are removed (BLASTER, MATCHER) as well as elements classified as SSRs (>0.75 SSR coverage) or unclassified elements built from less than 10 fragments.

This set of *de novo* detected repetitive elements was used to mine the genome in the second pipeline with BLASTER (NCBI BLAST, sensitivity 4, followed by MATCHER), RepeatMasker (NCBI BLAST/ CrossMatch, sensitivity q, cutoff at 200) and CENSOR (NCBI BLAST). False positive matches were removed by an empirical statistical filter. Satellites were detected with TRF, MREPS and RepeatMasker and were then merged. In addition, the genomic sequences were screened for matching nucleotide and amino acid sequences from known transposable elements (RepBase 17.01, [6]) via BLASTER (tblastx, blastx) followed by MATCHER. Finally a removal of redundant TEs, removal of SSR annotations included within TE annotations and "long join procedure" to connect distant fragments was performed. Sequences from the de novo repetitive element library which were found to have at least one perfect match in the genome were then used to rerun the whole analysis.

To ensure compatibility and to avoid introducing a bias, we refrained from a manual curation or clustering of the *denovo* detected elements before mining the genome. However, post hoc we manually analyzed all elements which were previously classified into class I retrotransposon or class II DNA transposon elements or unclassified elements with detected coding element features (similarity to known transposable elements) due to potential chimeric insertion. We excluded at this stage derivative elements (LARD, TRIM, MITE) from detailed further inspection unless carrying such a feature. Elements classified as “*potential Hostgene*” or unclassified elements (*noCat*) were also excluded at this stage. Manual inspection was done with ORF Finder (NCBI, http://www.ncbi.nlm.nih.gov/gorf/gorf.html), CDD search (NCBI, [9]), with a search in the most up-to-date online RepBase database (accessed December 2012-February 2013) via CENSOR ([10]) and phylogenetic analysis for LINE RT domains with RTclass1 ([11]) in order to achieve a detailed classification for each element, determine its potential relation to a family of known elements, to evaluate the completeness and to detect potential active elements. We defined an element to be complete if it possessed the relevant coding parts with the element-typical domains and the structural features (LTR, TIR). The potential activity was defined according to the region an intact ORF, if present, covered. If an intact ORF seemed to cover a complete region, including the typical domains (e.g. GAG as well as POL, Tase), then the element is considered to be potentially active. If a Tase domain is covered by a truncated ORF or the Tase itself appears to be truncated but is covered by an intact ORF, or if the RT domain is covered by an active ORF but not the remaining element-typical domains, then the element is considered to be maybe potentially active. During the manual classification to at least superfamily level, novel transposable element types not covered by the system of [8] were also considered: *Kolobok*, *Sola*, *Chapaev*, *Ginger*, *Academ*, *Novosib* and *ISL2EU* class II DNA transposons ([12], [13]).

Simple sequence repeats and other low complexity regions were extracted from the REPET pipeline database and processed with a custom Perl script to calculate the total coverage of these types of repetitive DNA by omitting overlaps with transposable element or other repetitive element annotations.

*Results*

Processing the centipede *S. maritima* assembly with the REPET pipeline yielded 7463 *de novo* predicted repetitive elements, of which 3715 were validated by annotation of at least one complete copy. In total 48.82% (86.03 Mb) of the genome assembly appears to be repetitive. Non-interspersed repeats (SSR, low complexity) accounted for 6.38% (11.24 Mb), whereas interspersed repeats represented 42.44% (74.79 Mb) of the centipede assembly.

All orders and most of the superfamilies of retro-transposable elements were detected in the genome of *S. maritima*. In comparison to other animals (e.g. human, insects, nematodes), LTR retrotransposons are very abundant: they account for 22.06 % of the assembly (38.86 Mb). By far the most frequent are elements from the *Copia* and *Gypsy* superfamilies, whereas *BelPao* elements are rare. Also elements from the orders DIRS, PLE, LINE and SINE were rare (each below 1% of the assembly). The small amount of LINEs is different to other organisms in which elements of this type are typically much more frequent. TRIMs and LARDs are derivatives of retro-elements and were detected in larger numbers, occupying 23.83 Mb (13.52%) of the assembly (Table S1).

Class II DNA transposons were less frequent and account for 2.3% of the assembly (4.06 Mb). The majority of these elements were TIR Transposons, especially of the *Mariner* and *Mutator* superfamilies. Interestingly, no fragments or elements of the *PiggyBac* superfamily could be found. Elements of this type are common is some insect genomes. Other types of DNA transposons, *Maverick (Polinton)* and *Helitron* could be found in small numbers only. The DNA transposon derivatives (MITEs) that were detected account for less than 0.74 % (1.3 Mb) (Table S1).

Besides the well-classified sequences, numerous elements could not be assigned to a superfamily or even class. The latter contains a larger number of elements (2.39%, 4.2 Mb), which could represent novel types but need further investigation. ‘Not categorized’ elements or detected elements which contained no typical transposable element feature, but had profiles from protein coding genes, were separately annotated and accounted. Both together comprise 13.04 % (23 Mb) of the assembly (Table S1).

Most of the elements appear to be fragmented and incomplete. Although some still contain sequences of typical transposable element protein domains, they seem to be inactive due to stop codons and frameshift mutations. However, we detected more than 700 retro- and 18 DNA transposons with RT or *Tase* domains, respectively. In particular, a high number of elements of the *Copia* and *Gypsy* superfamily appeared to be complete (n=75) and/or possess active ORFs containing at least the RT domain (n=189). Such elements were also found among the LINEs (complete n=7 / potentially active n=15; especially from the *RTE* superfamily), and were found from the PLE order (n=2 / n=2), as well as from TIR DNA Transposons (n=7 / n=8, especially *Mariner*, *hAT* and *Mutator*). These elements also appear to have higher abundance and a higher number of chimeric inserts (cf. e.g. Copia, Gypsy, Table S1), which would be consistent with recent activity.

If compared to other organisms, the genomic coverage of transposable elements is rather high, and is most striking for the retro-transposons. Other animal species have lower contents of such elements. Especially if compared to insects, the centipede shows a high amount of transposable elements in the genome (48 vs. 2-37%: [14], [15], [16], [17], [18], [19], [20], [21], [22], [23], [24], [25]). However, the genome of the mosquito *Aedes aegypti* appears to contain amounts similar to the centipede (50%, [26]). It is noteworthy, however, that the *S. maritima* assembly does not contain much of the repeat-rich heterochromatin, introducing a degree of ambiguity into simple comparisons of repeat density between such draft genomes.

Some superfamilies of DNA transposons could not be found or only in small quantities. For example *PiggyBac*, *hAT* and *P* elements are frequent in genomes of *Bombus impatiens* and *Drosophila*, the pea aphid, a lizard or *Atta cephalotes* ([27], [21], [28], [14], [29]), but were barely detected here.

We did not perform a particular scan for known Viruses, but while inspecting the transposable element sequences, some conserved protein domains or sequences similar to Baculoviridae were found.

*Abbreviations*

ORF open reading frame

LTR long terminal repeat

TIR terminal inverted repeat

SSR simple sequence repeat

RT reverse transcriptase

TASE Transposase

GAG GAG-Protein of retrotransposons

POL POL-polyprotein of retrotransposons

LARD large retrotransposon derivative

TRIM terminal repeat retrotransposon in miniature

MITE miniatiure inverted-repeat transposable element

SINE short interspersed element

LINE long interspersed element

DIRS Dictyostelium intermediate repeat sequence

PLE Penelope

**3. Phylome analysis and phylogenomics.**

*Phylome reconstruction.*

Proteins encoded in 18 fully-sequenced genomes, including the *S. maritima* genome, were downloaded from various sources (Table S2). The final database used for the phylome reconstruction contained 14,959 unique protein sequences for the centipede *S. maritima*. The resulting phylome comprises 11,112 single trees, which represents 74.28% of the used proteins.

To perform the phylome reconstruction, a Smith-Waterman [30] search was used to retrieve homologous sequences using an e-value cut-off of 1e-5, and considering only sequences that aligned with a continuous region representing at least 50% of the query sequence. Then, selected homologous sequences were aligned using three different programs: MUSCLE v3.8 [31], MAFFT v6.712b [32], and KAlign v2.08 [33]. Alignments were performed in forward and reverse direction (i.e. using the Head or Tail approach [34]), and the six resulting alignments were combined using M-Coffee [35]. The resulting combined alignment was subsequently trimmed with trimAl v1.4 [36], using a consistency score cut-off of 0.1667 and a gap score cut-off of 0.1, to remove poorly aligned regions.

Phylogenetic trees based on the Maximum Likelihood (ML) approach were inferred from these alignments. ML trees were reconstructed using the best-fitting evolutionary model. The selection of the evolutionary model best fitting each protein family was performed as follows: A phylogenetic tree was reconstructed using a Neighbour Joining (NJ) approach as implemented in BioNJ [37]; The likelihood of this topology was computed, allowing branch-length optimization, using nine different models (JTT, WAG, MtREV, VT, LG, Blosum62, DCMut, MtArt and Dayhoff), as implemented in PhyML v3 [38]; The two evolutionary models best fitting the data were determined by comparing the likelihood of the used models according to the AIC criterion [39]. Then, ML trees were derived using the two best-fitting models with the default tree topology search method NNI (Nearest Neighbor Interchange), and the one with best likelihood was used for further analyses. A similar approach based on NJ topologies to select the best-fitting model for a subsequent ML analysis has been shown previously to be highly accurate [40]. Branch support was computed using an aLRT (approximate likelihood ratio test) parametric test based on a chi-square distribution, as implemented in PhyML. In all cases, a discrete gamma-distribution with four rate categories plus invariant positions was used, estimating the gamma parameter and the fraction of invariant positions from the data.

*Orthology/paralogy predictions.*

Orthology and paralogy relationships among *S. maritima* genes and those encoded by the other considered genomes were inferred using a phylogenetic approach [41] (summarized in Tables S3 and S4). In brief, a species-overlap algorithm, as implemented in ETE v2 [42], was used to label each node in the phylogenetic tree as duplication or speciation depending on whether the descendant partitions have, at least one, common species or not (i.e. using a Species Overlap Score of 0). The resulting orthology and paralogy predictions can be accessed through phylomeDB.org [43]. These predictions have been used in subsequent analyses such as orthology-based functional annotation, identification of gene expansions, or duplication dating.

*Phylogenomics.*

We took the opportunity provided by the first complete myriapod genome to investigate arthropod relationships from a genome-wide perspective. A possible advantage of using complete genomes to reconstruct evolutionary relationships among arthropods is that large data sets minimise stochastic or sampling error. A multi-gene phylogeny for the species included in the phylome was inferred using 1,491 gene families with a clear, phylogeny-based, one-to-one orthology present in at least 15 out of the 18 species included in the analyses (Figure S2). Protein sequence alignments were performed as described above and then concatenated into a single alignment of 842,150 columns. Species relationships were inferred from this alignment using a Maximum Likelihood (ML) approach as implemented in PhyML [39], using LG as the evolutionary model, since in 1,330 out of 1,491 gene families this model was the best-fitting, with the tree topology search method set to SPR (Subtree Pruning and Regrafting). Branch supports were computed using an aLRT (approximate likelihood ratio test) parametric test based on a chi-square distribution.

*Increasing taxon sampling for phylogenetic inference.*

In order to increase the taxon sampling for the Chelicerata, 5 additional species were used to infer a species phylogeny. Depending on the current status of each genome, two different strategies were used to identify the original 1,491 sets of widespread single-copy proteins in these newly considered species. If only the assembly was available then an exonerate [44] *protein2genome* search was executed using all sequences from each dataset as queries. Only 5 best-hits were retrieved and predictions were filtered out to keep only those with a single copy on the target genome with introns with sizes smaller than 10,000 bp. If a complete proteome was available, then a Bi-directional Best Hit (BBH) search using BLAST [30] with similar parameters to the ones used during phylome reconstruction was performed. Table S5 shows the newly added species as well as how many protein-coding genes were identified using the two strategies.

*Investigating* S. maritima *phylogenetic position in the context of Arthropoda evolution.*

The link between myriapods and chelicerates (Myriochelata) suggested by some molecular studies is in conflict with morphological characters linking the Myriapoda with the Pancrustacea. As mentioned in the main text, current consensus suggests that myriapods, insects and crustaceans form a monophyletic group, the Mandibulata. Support for Myriochelata is widely held to stem from difficulty in resolving the short Mandibulata node coupled with subtle effects of systematic biases in the data. As the difference between the two hypotheses hinges on the placement of the outgroup taxa, the use of a closely related outgroup that does not exhibit obvious systematic bias is desirable.

To further investigate the phylogenetic position of *S. maritima* in the context of Arthropoda evolution, a new species phylogeny was reconstructed using 5 additional Chelicerata species and removing 6 distant and fast-evolving species from the initial set (Figure S3). Alignments were reconstructed for this new set of species and best-fitting evolutionary models determined as described above. Then, a multi-gene phylogeny was reconstructed based on the concatenation of the 1,491 sets of widespread single-copy protein-coding genes. A maximum-likelihood tree was derived from the concatenated alignment of 829,729 columns by using PhyML [39] with LG as the evolutionary model, since in 1,229 out of 1,491 gene families this model was the best-fitting, with the tree topology search method set to SPR (Subtree Pruning and Regrafting). Branch supports were computed using an aLRT (approximate likelihood ratio test) parametric test based on a chi-square distribution.

To investigate the statistical support for the current placement of *S. maritima* regarding the different groups of arthropod species in the two reconstructed species phylogenies, different topologies were evaluated (see Figure S4). Using ETE v2 [42] three different topologies were generated with all possible placements of *S. maritima* relative to the two arthropod groups considered in this analysis: Chelicerata and Pancrustacea. In order to avoid any potential bias on the likelihood values introduced by a specific organization within each group, only specific nodes were constrained and, therefore, the groups’ internal organization was inferred in a later step.

Maximum likelihood trees were reconstructed with PhyML v3.0 [39] using as input the alignment corresponding to the 1,491 marker genes and the three different alternative topologies evaluated. LG was used as the evolutionary model since it best fits most of the individual marker genes in both cases, and the SPR (Subtree Pruning and Regrafting) algorithm was used as the tree topology search method. Branch support was computed using an aLRT (approximate likelihood ratio test) parametric test based on a chi-square distribution. In all cases, a discrete gamma-distribution with four rate categories plus invariant positions was used, estimating the gamma parameter and the fraction of invariant positions from the data. PhyML was set to follow constraints on the input topologies while the internal organization of the different collapsed groups was optimized. Likelihood values for each alternative topology were used to evaluate the statistical support of alternative positions of *S. maritima* with CONSEL [45]. CONSEL evaluates, using 8 statistical tests, the likelihood values for each of the input topologies and decides whether the observed differences, in terms of likelihood, are significant or not and, therefore, if alternative topologies to the most supported one should be considered. Tables S6 and S7 shows the results after applying CONSEL to the likelihood values generated for the initial set of species (Table S6) and for the new set of species after including 5 additional Chelicerata species and removing 6 distant and fast-evolving out-group species (Table S7).

The most recent analysis, combining many genes and a denser taxon sampling than we can achieve using whole genomes, recovers the Mandibulata with significant support. It is in the context of this phylogeny that we interpret our phylogenomics data as inconclusive. While our large number of genes is likely to have removed stochastic error, systematic error may remain. Additional genomic data from slowly evolving ecdysozoan outgroups, such as priapulids, and from additional myriapods would likely help in resolving this issue.

*Orthology-based functional annotation.*

To complement genome functional annotation, we searched for centipede proteins that had one-to-one orthology relationships with 9 arthropod species: *A. pisum, A. gambiae, B. mori, D. pulex, D. melanogaster, I. scapularis, N. vitripennis, P. humanus* and *T. castaneum*, for which GO terms are available. Of the 5,984 one-to-one orthologues (~40% of centipede genome), 4,930 of them mapped to at least one arthropod gene with some GO annotation. Annotated GO terms using this strategy are provided in File S1.

*Lineage specific expansions in* S. maritima*.*

We focused on lineage-specific expansions in the centipede genome for which 4,796 protein-coding genes (~32%) were mapped to such events. Since many protein-coding genes were detected as part of expansions across several single-gene trees, a clustering step was performed in order to group such genes into unique events. Genes were assigned to the same cluster if the overlap among expansions, in terms of shared genes, was at least of 50%. Using this cut-off 76.5% of the genes mapped to lineage-specific expansions were assigned to a unique cluster. Figure S5 shows the frequency of number of protein-coding genes per cluster in those cases with 5 or more members.

*Functional categorization of the largest lineage specific expansions.*

Clusters of duplicated centipede protein-coding genes specifically expanded in this lineage were analysed, looking for any statistically significant functional enrichment. Functional enrichment is provided for the 10 biggest clusters with statistically significant enriched terms. Enrichment analyses of over-represented GO terms for these expanded families compared with the annotated *S. maritima* genes were performed by using FatiGo as implemented in Babelomics webserver [46] using the Fisher exact test for genome comparison and e-value cut-off of 0.001. GO terms redundancy was reduced using REViGO webserver [47] with default parameters. Table S8 shows these enriched functional terms for the 10 largest clusters.

*Dating of duplications.*

We scanned the phylome to detect and date duplication events, using a previously described algorithm [48]. We focused on events assigned to seven different relative evolutionary periods: Age (01) *S. maritima* specific, Age (02) Arthropoda I which groups *S. maritima* and *I.* s*capularis* according to the most likely species tree, Age (03) Arthropoda II which groups *S. maritima*, *I. scapularis* with all Pancrustacea species included in the study, Age (04) duplications at Ecdysozoa level, Age (05) duplications dated at Protostomia level, Age (06) duplications mapped to the Bilateria group of species, and Age (07) which includes duplications dated at the base of all species used in this study, equating to the base of Eumetazoa. Individual trees were scanned and all duplication events that involved the seed protein and others centipede proteins were dated. Summary about such analysis can be found in Table S9.

*Functional enrichment for dated duplicates.*

*S. maritima* proteins duplicated at different relative ages were analyzed looking for any functional enrichment. Enrichment analyses for over-represented GO terms for the dated duplicated protein-coding genes compared to the whole set of annotated centipede proteins were performed using FatiGO as implemented in Babelomics webserver [46]. A Fisher exact test looking for overrepresented terms in specific sets of proteins against the whole annotated genome was used with an e-value cut-off of 0.001. GO terms redundancy was reduced using the webserver REViGO [47] with default parameters. Table S10 shows over-represented terms grouped by age and ontology. Notably, given the few duplications detected at age 2: Arthropod I, there are no enriched functional terms for this category.

**4. Synteny methodology.**

Synteny analysis tested for linkage of orthologous genes on the same chromosomes (or scaffolds in the case of incomplete assembly) in pairs of species. This is sometimes called *macro-synteny* to distinguish it from analysis of more localized *micro-synteny.* A separate gene orthology analysis was performed than that in section 2 of this supporting information, as described in [50] and its supplemental data, except with a larger species tree (see list of species used here in Table S12). The clustering method performs two merging steps at each node of the species tree, working from leaves to root. In the first step (omitted at the leaves), two gene clusters from different sides of a branch are merged based on mutual best BLASTP hits with each other’s members (without considering outgroups). In the second step, clusters within the current node’s subtree are merged if they have mutual BLASTP hits not blocked by better hits to genes in the outgroup.

To test for significant conservation of macro-synteny we made comparisons to a null model of the number of orthologous genes that two regions in different genomes would share by chance. If the effects of gene duplication and loss are ignored, the number of shared orthologue groups would follow a hypergeometric distribution (applying Fisher’s exact test). Differences in gene family size resulting from gene duplication and loss make this distribution only approximate, and we limit the effect of such changes by excluding orthologue groups with more than ten members from the analysis.

We determine the block-synteny summary statistic, *P,* as follows. Given the computed clusters of orthologous genes, within each genome we pre-grouped the scaffolds (or chromosome segments, in the of cases of *Homo sapiens* and *Trichoplax adhaerens*) into Putative Ancestral Linkage groups (PALs) as described in [50]. *P* then represents the percentage of genes in the two species having cross-species orthologues and having them in the PAL homologous to their own PALs.

To assign PAL homology relationships in pairwise genome comparisons, we used the log-likelihood score, log(mp), to measure the orthologue concentration for each pair of PALs, where m is the number of pairwise PAL comparisons between each pair of genomes (*i.e.* a multiple test correction) and p is the probability of the observed number of shared orthologues relative to the null model that the two PALs draw their genes independently from the their common ancestor. Each PAL is considered homologous to the PAL with which it has its lowest log-likelihood score in the other genome.

Comparing the block-synteny summary statistic, *P*, *S. maritima* shows the greatest synteny of any non-chordate (Table S13). In particular, it shows upwards of 60 percent synteny with chordates such as humans and *B. floridae*, while the moth *Bombyx mori* shows only slightly more than 40 percent synteny with the analysed species other than *S. maritima*.

**5. Homeobox genes**

*Homeobox gene inventory and retention of Dmbx, Vax and Hmbox*

We used the complete homeobox catalogues of an insect and chordate (*Tribolium castaneum* and *Branchiostoma floridae* respectively) as queries for a saturated search of the whole genome assembly as well as the unassembled reads of the *S. maritima* genome sequencing project. We found 113 homeobox-containing genes. This compares to 133 homeobox genes in the chordate amphioxus and 104, 103, and 93 in insects such as *Drosophila melanogaster, Tribolium castaneum* and *Apis mellifera*. Of these 113 *S. maritima* homeobox genes, seven are very divergent and it is difficult to determine their orthology precisely. However, with a combination of molecular phylogenetics with Neighbour-Joining, Maximum-likelihood and Bayesian approaches, and using additional information from domains or sequence conservation outside of the homeodomain, we can include three of the seven genes in the ANTP class (two) and PRD class (one). Apart from the remaining four unclassified sequences, we find 54 ANTP-class genes, 26 PRD-class genes and 29 distributed amongst the nine remaining classes that are usually recognized. We found two genes with more than one homeobox, one in the Zinc Finger (ZF) class (containing four homeoboxes) and one in the Cut class (containing two homeoboxes) (Figure S7, Figure S8, Figure S9; Table S14, Table S15).

The number of *S. maritima* homeobox genes is slightly larger than the numbers found in most other arthropods analysed so far. This, at least in part, may be due to several instances of lineage-specific duplications alongside a distinct lack of homeobox gene loss in *S. maritima*. We find multiple copies (usually two to three) of Eve, Not, Vnd, BarH, Btn, Cad, Ind, Unc4, Otd and Irq. There is also a duplication of a potential Hox3 gene, discussed below. A further distinctive feature of the *S. maritima* homeobox complement is the presence of Vax and Dmbx, which have not previously been found in an arthropod genome. These genes can no longer be thought of as representing losses from the Arthropoda as a whole. Also, we find a *S. maritima* Hmbox gene, which is a member of the HNF-class. This is interesting on two counts. Firstly, the HNF class as a whole is missing from other arthropod genomes like those of the insects, and so this represents the first example of an arthropod HNF class gene described to date. Secondly, Hmbox genes have previously been proposed as chordate-specific, in contrast to more ancient members of the HNF class like HNF1/Tcf (a gene present in diploblasts as well as several bilaterians) [51]. Thus, this *S. maritima* Hmbox gene (which possesses a POU-like domain, the typical insertion for HNF-class genes of 15-20 amino acids between the second and third helix in the homeodomain, and bootstrap support of 92.6% for a grouping with chordate Hmbox genes in a HNF-class tree Figure S10) implies that Hmbox genes are not chordate-specific but have been widely lost in multiple lineages of the animal kingdom. Also, the ancient HNF1/Tcf family has instead been lost from *S. maritima*.

*The first ecdysozoan Xlox ParaHox gene?*

The clustering and linkage of homeobox genes is often of functional significance (e.g. the Hox genes) or provides an important insight into the origins of this gene family as well as a useful proxy for the degree of genome rearrangement relative to other species. In contrast to the intact Hox cluster, its evolutionary sister the ParaHox gene cluster is not intact, which reflects the situation found in other ecdysozoans as well. In addition to the break-up of the ParaHox cluster, the ParaHox genes of *S. maritima* have undergone duplications, producing two copies of Ind (and a third Ind-like gene) and three of Cad, which is likely to have implications for their roles in early development of the ectoderm, nervous system and gut. No ecdysozoan Xlox, which is the third ParaHox gene, has been described to date. The counterpart to the Xlox ParaHox gene from the Hox cluster (following the ProtoHox to Hox/ParaHox model of [52]) is Hox3. In *S. maritima* Hox3 is absent from the Hox cluster, but elsewhere within the genome there are two genes with sequence affinities to Hox3/Xlox. It is thus interesting to try to determine whether these two *S. maritima* Hox3/Xlox genes are either Hox genes that have somehow translocated out of the Hox cluster (and Xlox is absent from *S. maritima* as with other ecdysozoans), or instead these genes are the first examples of ecdysozoan Xlox genes (and Hox3 has been deleted from the *S. maritima* Hox cluster and genome). The further possibility that one of these genes is a Hox3 orthologue and the other is *S. maritima* Xlox is unlikely, due to the highly supported grouping of both genes together in phylogenetic trees, which implies that they arose from a duplication specific to the *S. maritima* lineage. A Neighbour-Joining phylogenetic tree of the entire coding sequences of these *S. maritima* Hox3/Xlox genes along with a selection of Hox1, Hox2, Hox3, Hox4 and Xlox genes reveals some affinity of the *S. maritima* genes with the Xlox genes of amphioxus, *Lottia* and *Capitella*. However, it is noteworthy that the bootstrap support value for this association is very low (only 33%) and so the grouping of the *S. maritima* genes with Xlox genes of other species cannot be considered as significant (Figure S11). Further phylogenetic analysis, focusing on the most similar regions of the Xlox and Hox sequences, including the hexapeptide and homeodomain regions (Figure S12) and rooting the trees with some members of the PRD class, now reveals a possible affinity with Hox3 genes rather than Xlox (Figure S13). But again there are no significant support values for this Hox3 grouping (the 42.9% support value is not shown in the tree as the threshold is 50%).

An alternative approach to phylogenetic trees that can sometimes help with resolving gene orthology is comparison of synteny [53]. One of the *S. maritima* Hox3/Xlox genes (*Hox3b_Sma*) is on a small scaffold with no gene neighbours and so comparative synteny cannot be analysed, but the second gene (*Hox3a_Sma*) is on a scaffold with 94 other genes (scaffold JH431820). We find that by reciprocal best BLAST searches against the human genome (v68 from ENSEMBL) we retrieve 24 one-to-one *S. maritima* to human orthologues (Table S16). Examining the locations in the human genome of these 24 genes reveals that five genes are located within chromosomes bearing human Hox clusters, five within chromosomes bearing human ParaHox loci and 14 in chromosomes with neither a Hox or ParaHox association (non-Hox/ParaHox chromosomes). Using Fisher’s Exact Tests we find no significant associations with Hox, ParaHox or non-Hox/ParaHox chromosomes (Figure S14) (all the tests p >=0.6). As with the phylogenetic analyses, the synteny analyses also unfortunately do not resolve whether these *S. maritima* genes are orthologues of Hox3 or Xlox. Further sampling of other ecdysozoan lineages is thus required in order to determine whether Xlox really has been lost from all lineages of this super-phylum.

*Homeobox gene clusters: NK, Irx, Otp-Rx-Hbn*

In addition to the clustering of Hox genes, some arthropods also contain an NK gene cluster, which is involved in mesoderm development and provides an additional example of gene clustering that is likely due to the regulatory mechanisms operating on the genes (which as yet are poorly characterized) [54, 55]. *S. maritima* does not possess an intact NK cluster, but does have some gene pairs that are remains from the ancestral cluster, potentially reflecting the retention of some shared regulatory mechanism(s). These pairs are tinman and bagpipe (often known as NK4 and NK3 in chordates), and slouch (NK1) and Drop (Msx) (Figure 4). In addition, the NK cluster remnant of *bagpipe* (*bap*) and *tinman* (*tin*) is linked with Vax (Figure 4), this linkage being relatively tight as there are only seven intervening genes. This linkage is also conserved in the mollusc *L. gigantea*, however, the number of intervening genes is larger as is the distance between bap and Vax (which is 747 Kb). Thus, the linkage of Vax with the NK cluster is likely an ancient aspect of the organisation of these genes, dating to at least the divergence of the Ecdysozoa and Lophotrochozoa. Vax can thus be included as a new member of the ancestral ANTP-class Mega-homeobox cluster that arose deep in animal ancestry [56, 57] (see below).

There is also a cluster of three Irx/Iroquois homeobox genes in *S. maritima* (Figure 4). The three-gene Irx/Iroquois clusters of insects and chordates are independently derived [51, 58, 59]. The three-gene cluster of *D. melanogaster* arose from an ancestral state (still present in most other insects) of two genes, one being orthologous to *mirror* and the second being pro-orthologous to *araucan* and *caupolican*. Two of the *S. maritima* *Irx* genes have affinity with the insect *mirror* gene in phylogenetic trees (Figure S15). This may indicate that the three-gene cluster of this myriapod arose by duplication of the *mirror* gene rather than the *araucan/caupolican* gene, in contrast to the route to the three-gene cluster of *Drosophila*. The *S. maritima* Irx/Iroquois cluster thus represents a further example of the repeated independent expansion of this gene cluster in multiple lineages of the animal kingdom [51, 58, 59].

An additional example of an ancient homeobox gene cluster is the PRD class cluster involving *Orthopedia* (*Otp*), *Rax* (*Rx*) and *Homeobrain* (*Hbn*). This cluster, which is present in *S. maritima* (Figure 4), is also found in cnidarians, insects and molluscs [60].

*Homeobox gene clusters: SuperHox and Mega-homeobox*

The ANTP-class of genes (including the Hox, ParaHox and NK genes) evolved very early in animal evolution, probably via states in which many of the genes were clustered into a Mega-homeobox cluster before the origin of the bilaterians and a SuperHox cluster in the Urbilaterian [56, 57, 61]. We have found some remains of this SuperHox cluster in *S. maritima* (Figure 4), represented by the linkage of Exex (Mnx)-Nedx-BtnA (Mox) in scaffold JH431734 and the linkage of BtnB (Mox) with En in scaffold JH431870. The Hmbox gene is linked to the Exex-Nedx-BtnA SuperHox remnant in *S. maritima* (Figure 4). It remains to be seen, following further phylogenetically widespread genome sequencing, whether such a linkage represents a remnant of an ancestral state, and hence a new member of the SuperHox cluster.

The tight linkage of *Ems* with the *IndB* ParaHox gene is potentially revealing with regards to the evolution of the Mega-homeobox cluster. *Ems/Emx* is a member of the ancestral NK linkage group [57, 62], whilst *IndB* is a ParaHox gene. This tight linkage of these two genes in *S. maritima* may thus be a remnant of their existence in the Mega-cluster from early in animal evolution, with *S. maritima* thus providing new evidence in support of the Mega-homeobox cluster hypothesis. We note, however, that NK and ParaHox genes have become secondarily linked again in vertebrates (having been on distinct chromosomes in the chordate and lophotrochozoan ancestors) [62]. Whilst this tight *Ems – IndB* linkage is intriguing, further, phylogenetically widespread examination of ANTP-class homeobox linkage patterns is certainly required to establish the veracity (or otherwise) of the Mega-cluster hypothesis. Similarly, the linkage of the ParaHox-like gene, *Ind-like*, with the NK gene *scro* may also be indicative of an ancestral linkage in the Mega cluster. However, this *Ind-like – scro* linkage in *S. maritima* is looser than the linkage of *Ems – IndB* (273kb versus 10kb (Figure 4)) and so a secondary association cannot presently be excluded.

Finally for the homeobox super-class, the linkage of the SINE class gene, *sine oculis* (*so*), with *Ems* is not unique to *S. maritima*. Humans have two semi-orthologues of *so*, namely *SIX1* and *SIX2*, and two semi-orthologues of *Ems*, namely *EMX1* and *EMX2*. *SIX2* is linked with *EMX1* on human chromosome 2, a linkage that is also echoed on zebrafish linkage group 13. A linkage of these SINE and ANTP-class genes at least as old as the bilaterian ancestor thus seems likely.

The assumptions underpinning the deductions about the Mega-homeobox and SuperHox clusters are that these homeobox genes are most likely to have arisen via tandem duplications and that close linkage of these genes in multiple lineages is most likely indicative of an ancestral condition, rather than reflecting a secondary ‘coming together’ of the genes. The various alternative hypotheses are discussed in Hui et al (2012) [62], which concludes that more needs to be known about the evolutionary dynamics of genome organisation in order to more reliably assess the true likelihood of the Mega-homeobox and SuperHox cluster hypotheses. For the present time, however, the Mega-homeobox and SuperHox hypotheses remain the most parsimonious frameworks for understanding the evolution of homeobox gene linkage patterns.

*Hox mRNA processing in* S. maritima

The generation of alternative RNA isoforms through RNA processing mechanisms such as alternative splicing (AS), alternative polyadenylation (APA) and alternative promoter usage (APU) is a prominent feature of the *Drosophila* Hox genes [63 – 75]. Several studies have looked at the functional implications of Hox RNA processing in fruit flies and concluded that AS (leading to the generation of distinct protein isoforms) as well as APA (which produces mRNA transcripts bearing different 3’ un-translated regions, 3’UTRs) can influence gene expression and function during fruit fly development [75 – 80]. Although recent work has identified alternative RNA isoforms for some of the Hox genes in other insects (Bomtorim et al., *pers. comm.*) and even mammals (Patraquim et al., *pers. comm.*) the evolutionary origin and developmental roles of Hox RNA processing within the arthropods remain very poorly understood. The availability of genomic and RNA sequencing data from *S. maritima* offers an unusual opportunity to explore these questions.

Based on the information currently available from the *S. maritima* genome and transcriptome project we note the existence of at least nine Hox genes in this organism (see main text Figure4A). Of these, RNA sequencing data indicate that at least six *S. maritima (Sm)* Hox genes (i.e. *Antp, Ubx, abd-A, lab, Dfd, pb*) produce more than one mRNA isoform (Figure S16, Figure S17). In all these six cases APA generates mRNAs bearing distinct 3’UTR sequences which might interact differentially with RNA regulators such as RNA binding proteins (RBPs) and microRNAs (miRNAs). Differential splicing with concomitant APU events concern two *S. maritima* Hox genes *Dfd* and *ftz* (Figure S16, Figure S17).

All in all, more than three quarters of the *S. maritima* Hox genes undergo RNA processing of one type or other (Figure S17, Panel A). Similarly, seven out of the eight *D. melanogaster* Hox genes produce different mRNA isoforms (Figure S17, A) (FlyBase, http://flybase.org/). Three *D. melanogaster* Hox genes undergo AS and five produce different transcripts via APA (Figure S17, Panel B) (FlyBase http://flybase.org/). In addition 5 fruit fly Hox genes form different RNA species by APU (Figure S17, Panel B). From this comparison we conclude that the patterns of AS and APA affecting the centipede and *Drosophila* Hox genes are relatively similar to one another; in contrast, APU seems more prevalent in the *Drosophila* (5 out if 8 genes) than in the centipede (2 out of 9 genes) Hox genes.

Regarding the developmental progression of Hox RNA processing patterns in *S. maritima* we note that some genes such as *Ubx* display high heterogeneity in 3’UTR sequences within the embryonic transcriptome (“eggs” data) suggesting the possibility that *S. maritima Ubx* APA might be developmentally controlled and/or display a tissue-specific pattern (Figure S16). However this is not a general case as the data available for most other *S. maritima* Hox genes do not support developmentally variable APA patterns. In contrast, during *D. melanogaster* embryogenesis several Hox genes undergo APA (Thomsen et al. 2010).

We also see that the *S. maritima* transcriptome data supports a previously described bicistronic *Hox* mRNA unit bearing the coding sequences for *Ubx* and *Antp* [81]; interestingly, the transcriptome data would also be consistent with a similar bicistronic structure concerning *ftz* and *Scr,* however this signal could also be explained as the product of antisense transcription over these genes.

A possible reason underlying the similarities between *S. maritima* and *D. melanogaster* Hox AS and APA patterns might be that these RNA processing patterns represent an ancestral feature of the arthropod Hox clusters retained in both organisms. Alternatively, both organisms might have developed similar molecular strategies concerning the RNA regulation of their Hox genes as a result of convergent evolutionary processes. To discriminate among these alternative scenarios a possibility is to look in higher detail at particular RNA processes affecting specific genes, scanning for molecular signatures that could imply common ancestry (or highly improbable convergent processes). We see such a signature in the three posterior-most *Hox* genes: *Ubx, abd-a, Abd-b* which undergo a specific type of APA (tandem APA) in both *S. maritima* and *D. melanogaster,* providing an example of what might be a feature present in the ancestral Hox cluster to insects and myriapods (Table S17). Nonetheless for most other Hox genes RNA processing patterns differ markedly between *S. maritima* and *D. melanogaster.*

Emerging genomic and transcriptomic information from spiders, crustaceans and non-Drosophilid insects should provide important elements to deduce the most likely evolutionary sequences concerning the molecular control of Hox gene expression by RNA processing.

**6. Non-homeobox gene clusters: innexin, Runt, E(spl)-C**

Clustering is not confined to homeobox genes. Innexins are a family of gap junction proteins, related to the vertebrate Pannexins [82]. We identified thirteen *innexin* genes in *S. maritima*. Five of them are located in a cluster composed of an innexin2, two innexin7s, an innexin1 and an innexin8 orthologue. This cluster is also present in *N. vitripennis* and *T. castaneum*, but is broken up in *D. melanogaster.*

In insect genomes, besides the clusters described above, Runt and Enhancer of Split (E(spl)-C) complexes exist. In contrast to the widespread occurrence of the various homeobox gene clusters, the Runt and E(spl)-C complexes appear to be arthropod specific. In most insects, the Runt complex comprises four Runt domain transcription factors [83]. In *Daphnia pulex*, an orthologue of one of these genes is present, clustered with two out of three other *D. pulex* Runt domain genes that are difficult to classify by phylogenetics. The chelicerate *I. scapularis* has two Runt domain genes, neither clear orthologues of the genes in the insect cluster. In contrast to these species, the *S. maritima* genome has only a single Runt domain transcription factor, providing evidence that the *Drosophila* Runt complex was an insect innovation not found in other arthropods.

E(spl)-C is a conserved Notch responsive element comprising four genes of both basic helix-loop-helix (bHLH) and bearded class genes [84]. The complex is greatly expanded in *D. melanogaster*, present in *D. pulex,* but absent from *I. scapularis*. *S. maritima* possesses 12 bHLH genes, most not found in complexes. Two complexes of these genes do exist, one made up of hairy and deadpan-like genes, the other comprising two E(spl)-like genes, but with no clear orthology relationship to E(spl)-C genes, which have characteristic bHLH-orange domains, and no bearded class genes. These data imply that E(spl)-C is a crustacean/insect complex with no orthologous complex in other arthropods.

**7. Chemosensation: Gustatory receptors (GRs).**

The gustatory receptor (GR) family of seven-transmembrane proteins mediates most of insect gustation (e.g. [85, 86]), as well as some aspects of olfaction, for example, the carbon dioxide receptors in flies (e.g. [87]). It ranges in size from 10-200 genes, but most insects examined so far have 50-100 genes. The GR family is more ancient than the OR family, which was clearly derived from within it, and is found in the crustacean *Daphnia* *pulex* [88], the tick *Ixodes scapularis* (HM Robertson, unpublished), and many other animals (HM Robertson, unpublished).

The GR family was manual annotated using methods employed for insect, *Daphnia*, and tick genomes. Briefly, TBLASTN searches were performed using major lineages of insect, Daphnia, and tick GRs as queries, and gene models were manually assembled in TextWrangler. Iterative searches were conducted with each new centipede protein as query until no new genes were identified in each major subfamily or lineage. When available, contigs of ESTs from RNA-seq experiments on whole animals of each sex and eggs were employed to confirm or refine gene models (Table S18). Two checks for possible divergent genes/proteins were performed. The first was a PSI-BLASTP search of the automated annotations with two iterations, and the second was TBLASTN searches of the three transcriptome assemblies with all of the existing GRs. Neither revealed additional GR lineages, although the presence of only a few of the identified GRs in the automated gene models and in the EST contigs means these checks are not conclusive. All of the SmGr genes and encoded proteins are detailed in Table S18. All SmGr proteins are provided in FASTA format (SI_file3).

Several difficulties with the genome assembly were encountered in this gene family. These were primarily length differences in homopolymer regions that in the assembly appeared to cause frameshifts within exons, but on examination of the raw reads these could be corrected. These presumably result from the known homopolymer length difficulties encountered with 454 pyro-sequencing. Seven gene models were corrected (suffix FIX in the figure, table, and FASTA). One gene model (Gr68) was designed that spans scaffolds, with no support other than the agreement of the available exons on both scaffolds, and their appropriate relatedness to similar genes in the tandem array in scaffold scf7180001247276. These problems are noted in Table S18.

Pseudogenes were translated as best possible to provide an encoded protein that could be aligned with the intact proteins for phylogenetic analysis, and attention was paid to the number of pseudogenizing mutations in each pseudogene. A 200 amino acid minimum was enforced for including pseudogenes in the analysis (roughly half the length of a typical GR), and there are several shorter fragments of genes that were not included in Table S18 or the analysis. All *Daphnia* and *Ixodes* GRs, and representative carbon dioxide and sugar receptors from insects (the most highly conserved GR lineages in insects), were aligned in CLUSTALX v2.0 [89] using default settings. Problematic gene models and pseudogenes were refined in light of these alignments.

For phylogenetic analysis, the poorly aligned and variable length N-terminal and C-terminal regions were excluded (specifically 15 amino acids before a conserved G residue in the N-terminus and immediately after the conserved TYhhhhhQF motif in the C-terminal TM7 domain, which somewhat unusually in these SmGRs has S or T instead of the final F, and this is often the final or penultimate amino acid), as was a major internal region of length differences, specifically a long length difference region in the internal loop 2. Other regions of potentially uncertain alignment between these highly divergent proteins were retained, because while potentially misleading for relationships of major subfamilies (which are poorly supported anyway), they provide important information for relationships within subfamilies.

Phylogenetic analysis of this set of 202 proteins was carried out in the same fashion as for previous GR analyses (e.g. [90, 91], involving a combination of model-based correction of distances between each pair of proteins, and distance-based phylogenetic tree building. Pairwise distances were corrected for multiple changes in the past using the BLOSUM62 amino acid exchange matrix in the maximum likelihood phylogenetic program TREEPUZZLE v5.2 [92]. These corrected distances were fed into PAUP*v4.0b10 [93] where a full heuristic distance search was conducted with tree-bisection-and-reconnection branch swapping to search for the shortest tree. The resultant tree is shown in Figure S18 and Figure S19. Bootstrap analysis with 10,000 replications of neighbour-joining using uncorrected distances was performed to assess the confidence of major branches in the tree, and is shown above major branches in the tree. The tree was manually coloured and labels attached to lineages and subfamilies in Adobe Illustrator. The circular tree in Figure 5A has the same structure, but less detail.

The SmGr gene set consists of 76 models, comparable to that for *Daphnia* and *Ixodes*, and many insects such as *Drosophila* flies. Thirteen (17%) of these are apparent pseudogenes, seven gene models required repair of the assembly, and one was joined across scaffolds. The result is 62 apparently intact GR proteins. Less obvious pseudogenes (for example with small in-frame deletions or insertions, crucial amino acid changes, or promoter defects) would not be recognized, so this total might be high. Approximately eight gene fragments remain so short and incomplete they were not included, but some might represent intact genes.

The automated gene modeling had access to all available arthropod GRs in GenBank, for comparative information, but succeeded in building gene models for just nine of these 76 genes, only one of which was precisely correct. All others required at least one change, while 49 new gene models were generated (not including pseudogenes or those requiring repair of the assembly) (Table S18). Unfortunately, because these genes are typically expressed at low levels in only a few cells, only nine genes are represented by appropriately spliced EST contigs in the three transcriptomes (Table S18), nevertheless these manually built gene models are highly confident, because there are representative EST contigs for most subfamilies, and the basic gene structure for the entire SmGr set is a long first exon, followed by three short C-terminal exons separated by three phase 0 introns. The locations of these introns and their phases are the same as predicted by [94] to be ancestral to the entire insect chemoreceptor superfamily, and are also shared with Gr genes in other animals (HM Robertson unpublished). There were only two exceptions: Gr52 lost the first intron, and Gr76 gained a N-terminal phase 1 intron.

None of the major gene subfamilies known in the insects GRs are present in this centipede GR family, consistent with the large phylogenetic distance of centipedes from insects. Thus there are no obvious members of the sugar receptor subfamily (e.g. [90]), the fructose receptor (e.g. [95]), or relatives of the otherwise highly conserved carbon dioxide receptor subfamily (e.g. [91]). Instead, the centipede, *Daphnia*, and tick GRs form exclusive lineages in the tree (Figure S18 and Figure S19). The centipede GRs form six recently amplified subfamilies, with only a few older divergent proteins (GRs 12/13, 41/42, 52, and 75/76). This pattern of multiple recent gene subfamily expansions suggests that this centipede lineage has recently adapted to new chemical ecologies that have led to the retention and differentiation of new genes in multiple subfamilies. This pattern is reinforced by the presence of multiple pseudogenes within most of these subfamilies, presumably as some genes became redundant for the changing chemical ecology. Furthermore, most of these subfamily expansions involve tandem duplications, which is presumably how these new genes arose through unequal crossing over. The largest of these is Gr1-13 (Table S18), although the phylogenetic relationships of the genes in this expansion are complicated (Figure S18 and Figure S19), suggesting that this tandem array predates the divergence of subfamilies A and B. Rather strangely, this array was apparently duplicated at some point and separately expanded as Grs 14-27 in the same two subfamilies, but most of these genes are instead now singletons spread around the genome. Similarly, most of subfamily C (Gr28-38) is in a single tandem array. Unfortunately, given the extreme divergence of these centipede GRs from all insect GRs with known ligands or functions, no inferences of function can be ascribed to them, indeed it is possible that some are expressed in the antennae and involved in olfaction, as adaptation to terrestriality occurred independently in myriapods and insects.

**8. Developmental signalling systems**

*Phylogenetic analysis of the Transforming Growth Factorβ (TGFβ) ligands in Arthropods.*

For Figure S23, Maximum likelihood analysis using the WAG+i+g amino acid substitution model was carried out as described in [102]. Bootstrap values (1000 replicates) are indicated in percentages. The TGFβ protein family is divided in a Bone Morphogenetic Protein (BMP) and an Activin subfamily. In our analysis, the Mavericks belong to the Activins. Concerning the BMP subfamily, the *S. maritima* genome contains two closely related *decapentaplegic* (*dpp*) duplicates. *S. maritima* reveals that arthropods ancestrally possess a BMP10 orthologue, a protein that has been lost in *Drosophila*, and an ADMP (anti-dorsalizing morphogenetic protein) orthologue, a protein that was lost in an ancestor of the beetles and flies [102]. Interestingly, we found an orthologue of the inhibitory BMP3 ligand that was suggested only to be present in deuterostomes [103]. We propose that this gene was lost in the holometabolous insects. *S. maritima* possesses one clear Glass bottom boat (Gbb) orthologue. As previously shown in detail [104, 105], *Drosophila* and *Megaselia* *scw* are diverged duplicates of *gbb*. Two *S. maritima* BMPs (SMAR009587 and SMAR007428) do not group with significant support to any particular TGFβ family in our phylogeny and were not given a name. SMAR009587 clusters with the Gbbs, albeit with very low bootstrap values. SMAR007428 sometimes even clusters with the Activins. Phylogenetic analyses did not detect any close relation to vertebrate Nodal or Lefty of these BMPs; future analyses should reveal more about their evolutionary origin and function. Concerning the Activin subfamily, *S. maritima* possesses a clear Activinβ orthologue, but no orthologue of the Activin-like protein (Alp) Dawdle. A clear Myostatin and a clear Maverick orthologue were identified in the *S. maritima* genome. The branching order of the Myostatins is reversed and of the Mavericks slightly disturbed, possibly because of incomplete *Acyrthosiphon* sequences. Addition of the molluscan *Lottia gigantea* Myostatin sequence did not alter the directionality. The alignment is available upon request.

Abbreviations: Is=*Ixodes scapularis*; Dp=*Daphnia pulex*; Ap=*Acyrthosiphon pisum*; Ph=*Pediculus humanus*; Nv=*Nasonia vitripennis*; Am=*Apis mellifera*; Tc=*Tribolium castaneum*; Ag=*Anopheles gambiae*; Dm=*Drosophila melanogaster*; Ca=*Clogmia albipunctata*; Ma=*Megaselia abdita*; Lg=*Lottia gigantea*.

Genbank accession numbers for non-*Strigamia* genes used in the analyses: IsDpp=ISCW023553; DpDpp=EFX89580 (DAPPUDRAFT_347232); ApDpp1=XP_001945626; ApDpp2=XP_001946010; ApDpp3=XP_001944147; ApDpp4= XP_003245371; PhDpp=PHUM346320; NvDpp= XM_001607627; AmDpp=XP_001122815; TcDpp= EFA02913; AgDpp= AGAP007987; DmDpp= NP_477311; DpBMP10=EFX72705(DAPPUDRAFT_346932); TcBMP10=XP_973577; AmBMP10=XP_001120039; IsADMP=ISCW019844; DpADMP=EFX77345 (DAPPUDRAFT_225730); AmADMP=XP_392320; NvADMP=XM_001604700; IsBMP3=ISCW021631; ApBMP3=XP_001944767; DpBMP3=EFX74191 (DAPPUDRAFT_346933); IsGbb=ISCW019587; DpGbb=EFX74626 (DAPPUDRAFT_347233); ApGbb=XP_001947957; PhGbb=PHUM150910; AmGbb=XP_394252; NvGbb=XP_001603876; TcGbb1=EFA04645; TcGbb2=EFA04646; AgGbb1= XM_316789; AgGbb2= XM_320599; CaGbb, MaGbb and MaScw were obtained from http://diptex.crg.es [105]; DmGbb= NP_477340; DmScw= NP_524863; DpAlp=EFX87955 (DAPPUDRAFT_305466); IsAlp=ISCW010227; PhAlp=PHUM033950; AmAlp=XP_001122210; NvAlp=XP_003425497; TcAlp=XM_965262; DmAlp=NP_523461; IsAct=ISCW016200; ApAct=XM_003246878; PhAct=PHUM193490; NvAct=XM_001602234; AmAct=XP_001123044; TcAct=EFA05602; DmAct=NP_651942; AgAct=AGAP000342; LgMyo=ESO82089; IsMyo=ISCW005998; DpMyo=EFX67990 (DAPPUDRAFT_130202); PhMyo=PHUM135650; ApMyo1=ACYPI20476; ApMyo2=ACYPI49127; ApMyo3=ACYPI38027; TcMyo=XP_966819; NvMyo=XM_001602205; AgMyo=AGAP005289; DmMyo=NP_726604; DpMav=EFX89436 (DAPPUDRAFT_17212); AmMav=XM_001122118; NvMav was predicted from the genome sequence by extending XM_001606098; AgMav was predicted from the genomic sequence using XM_001656165 and AGAP012076. These two predictions are available upon request. ApMav=XM_003240719; AmMav=NP_524626; TcMav=XM_001811382.

**9. Histones and Histone modifying enzymes in *S. maritima***

The core unit of chromatin is the nucleosome, a highly conserved repeating unit composed of two copies of each of the four ‘core’ histone proteins (H2A, H2B, H3, H4) assembled into an octamer and wound around 146-147 bp of DNA. The linker histone H1 binds the nucleosome and locks the DNA into place by binding the entry and exit sites of the DNA.

Modification of the histone proteins by methylation, acetylation and phosphorylation dynamically influences the structure of the chromatin. Chromatin structure regulates gene expression by influencing the recruitment of transcription factors, the recruitment of RNA polymerase, and also additional histone modifying enzymes.

## *Histone genes in S. maritima.*

The core of the nucleosome is made up of the histone proteins H2A, H2B, H3 and H4.

The ‘core’ histones are highly conserved and orthologues can be reliably identified by BLAST analysis (Table S27). The histone H1 family are more divergent at the sequence level but we have identified three orthologues in *S.* *maritima.*

In general, *S. maritima* has fewer histone encoding loci than dipterans such as *Aedes aegypti* and *D. melanogaster* [106, 107] but number of loci encoding each class of histone are consistent with other arthropods (Table S28, [108, 109]). There are, however, more genes encoding the H2B core histone than observed in non-dipteran arthropods (Table S28).

In *Drosophila* the histone genes are present in the genome in large numbers of quintet clusters, each cluster having one gene from each of the five classes of histones. This arrangement of genes is observed in other insects such as the pea aphid [109], and we see one quintet cluster of histone genes in *S. maritima* (Figure S31, panel A). The remainder of the histone genes are only present as single copies on a scaffold, are interrupted by non-histone encoding genes (Figure S31, panel B) or are the result of recent gene duplications (Figure S31, panels C, D).

Two loci encoding the variant histones H2A.X and H3.3 were identified. H2A.X/H2A.Z (His2AV in *Drosophila*) is found throughout eukaryotes and is associated with heterochromatin and collapsed replication forks. Phosphorylation of this histone variant is associated with double stranded breaks in the DNA [110]. The H3.3 variant histone is also evolutionarily conserved and is associated with diverse regions of the genome in eukaryontes, including pericentromeric and telomeric regions. H3.3 is also enriched in actively transcribed genes where it is thought to replace the canonical histone H3 proteins during gene transcription [110].

We could not identify an orthologue of the male specific gene *mst77F,* which encodes a sperm specific linker histone in *Drosophila* [111].

## *Histone modifying genes in S. maritima.*

Histone acetyltransferases (HATs)

These enzymes catalyse the addition of acetyl groups to lysine residues on core histones. This favours a chromatin conformation that is accessible to transcriptional machinery and thus tends to favour active gene expression.

HATs are divided into three classes

1. MYST-type acetyltransferases: S. maritima has orthologues of all four D. melanogaster MYST acetyltransferase enzymes (Males absent on the first, Tip60, Enoki mushroom and Chameau).
2. GNAT (GCN5-type N-acetyltransferase)-type HATs: Orthologues of four D. melanogaster GNAT enzymes were found in S. maritima (CG2051, ATAC complex component 2, elongator complex protein 3 and Pcaf/GCN5)
3. p300/CBP(CREB binding protein) HATs: S. maritima has two orthologues of CREB binding protein, one (Smar_008296) has very high sequence similarity to D. melanogaster CBP (Neijre), the second (Smar_011410) is more diverged, but is most similar to D. melanogaster CBP.

HAT activity has also been ascribed to TBP-associated factor 1 [112] which has RNA polymerase II transcription factor activity and involved in pre-initiation complex assembly. S. maritima has two orthologues of Taf1 (Smar_007466 and Smar_005203).

The HAT gene complement of S. maritima is similar to that of other arthropods [109].

Histone deacetylases (HDACs)

HDAC enzymes remove the acetyl groups added to lysine residues on histones by the HAT enzymes. There are two classes of HDAC enzymes in animals; RPD3-type and Sir2 type (silent information regulator 2 or sirtuin 2).

The S. maritima genome encodes seven Rpd-type HDACs and four Sir2-type HDACs. Phylogenetic analysis indicates that the Rpd-type HDAC family of S. maritima includes two orthologues of Rpd3 (HDAC1), one orthologue each of HDAC3 and 4, two orthologues of HDAC8, and one orthologue of the class IV HDAC, HDACX.

Notably S. maritima does not have an orthologue of the HDAC6 gene. HDAC6 is an unusual histone deacetylase as it is located in the cytoplasm. HDAC6 binds to ubiquitin and deacetylates tubulin, and is functionally distinct from other HDACs. HDAC6 appears to function as a sensor of stressful environmental stimuli and an effector, which mediates and coordinates appropriate cell responses [113]. HDAC6 is highly conserved and is present in the genomes of *T. urticae*, *D. melanogaster* and *A. pisum*.

The sirtuin genes are NAD^+^ dependent deacetylase enzymes that have been hypothesised to be potentially responsive to environmental perturbation (including diet [114]). S. maritima has two orthologues of Sirt2, one of Sir2 and one of Sirt6. Sirt6 is an unusual HDAC as it is present in the cytoplasm.

**10. Germ line genes**

A small number of genes play conserved roles in germ line specification and development in all metazoans. As germ line specification relies heavily on post-transcriptional regulation, many of these genes encode RNA binding proteins, piRNA interacting proteins and translational regulators. In basally branching metazoans, multiple copies of genes such as *vasa, piwi* and *nanos* are present, whereas most of these genes are present only in a single copy in bilaterian genomes, barring genome-wide duplications. The evolution of such gene families is unclear for the arthropods, as genome data are available principally for insects.

We searched the *S. maritima* genome for the presence of 32 genes with known germ line function in at least *D. melanogaster* or mouse (see Table S29). For six of these genes (*c-Myc, fear of intimacy, aubergine, valois, Stella* and *oskar*) we failed to find any likely orthologues. However, we have found at least one putative *S. maritima* orthologue for each of the remaining 26 genes, most of which are found in a single copy. The *vasa* family of DEAD-box helicases comprises one *vasa-like* gene, one *PL10/belle* orthologue, and eight additional DEAD-box-containing genes, designated *Smar DEAD Box 1* through to *Smar DEAD Box 8*, which potentially represent a *S. maritima* specific expansion (Figure S32). The *piwi/Argonaute* family, which plays conserved roles in metazoan piRNA and stem cell regulation, is discussed in the section on Immunity and RNAi. In contrast to most other arthropod genomes examined to date, we found two *nanos* paralogues, both of which contain the conserved CCHC Zn-finger domains characteristic of *nanos* genes, and one of which is significantly shorter than most metazoan *nanos* orthologues. Duplication of the arthropod *nanos* has previously only been documented in the pea aphid *A. pisum*. The functional significance of these lineage specific duplications of *nanos* remains to be tested.

**11. Meiosis genes**

Among arthropod lineages, the diversity of reproductive modes often requires modifications to the key processes of meiosis. For example, parthenogenesis requires meiotic innovations to produce diploid eggs from asexual females (*i.e.* cyclical parthenogenesis) or sperm from haploid males (*i.e.* arrhenotokous parthenogenesis). In addition, *Drosophila* males undergo achiasmate meiosis (*i.e.* the absence of chiasmata between homologue pairs), which is reflected by the absence of meiotic recombination. *S. maritima* is an obligate sexual species, but little is known about meiosis within the Myriapoda. We have surveyed the genome of *S. maritima* for the presence of >50 meiosis-related genes. These genes are involved in many processes of meiosis, including cell-cycle regulation, homologue pairing, meiotic recombination and DNA repair. We also searched for these genes in the genomes of 23 additional arthropods, including members of the Hexapoda, Crustacea and Chelicerata (Figure S33). We performed phylogenetic analyses to confirm the identity of orthologues and to distinguish paralogues. For *S. maritima,* the majority of meiosis-related genes (including several meiosis specific genes) were identified. Genes absent in *S. maritima* have also been sporadically lost in other arthropods, suggesting that certain genes are dispensable for meiosis. Gene duplications found in *S. maritima* were not unique to that lineage, as paralogues were also identified in other arthropod genomes.

**12. CpG methylation.**

Gene body sequences were extracted from the predicted *S. maritima* gene set using CLC genomics workbench (version 5). For analysis of whole genome CpG[o/e] the genome sequence was split into 1000 nt non-overlapping fragments using a custom perl script. Nucleotide and dinucleotide content of gene body sequences and whole genome sequences were calculated using a custom perl script. The number of components in these distributions was estimated in R (www.r-project.org) using mclust [116] model-based clustering. The best fitting model was identified among several non-nested models using Bayesian information criteria (BIC). See text for further details and Figure S28, Figure S29 and Figure S30.

**13. Non-protein-coding RNA genes in the *S. maritima* genome**

Centipede microRNAs were computationally identified by two independent approaches, which produced >90% overlapping results. First, we retrieved precursor sequences of microRNA families conserved in all bilaterian animals, in invertebrates, in arthropods or in insects from miRBase (v 18; [117]). For each family, we searched for homologous sequences of its members in the *S. maritima* genome using BLASTN with the following parameters: -word_size=4 -reward=5 -penalty=-4 -gapopen=8 -gapextend=6. We then used INFERNAL 1.0.2 [118] to build covariance models based on the multiple sequence alignments of each microRNA family, and searched for similar profiles in the regions of *S,maritima* genome determined by BLASTN. Significant hits were added to the existing alignments, and results were manually inspected. In addition, we used MapMi [119] to map all known animal mature microRNAs to the *S.maritima* genome allowing three mismatches. Results scoring 35 or above were aligned and inspected manually for good sequence conservation and folding into microRNA-like hairpin using RALEE [120]. tRNA genes were predicted using tRNAscan-SE 1.23 with default parameters [121], and other non non-coding RNAs with the Rfam annotation pipeline (version 10; [122]) using INFERNAL 1.0.2 [118] and BLAST [123].

**14**. **mRNA purification and sequencing library construction.**

mRNA is purified from total RNA using Dynabeads® mRNA Purification Kit (Life tech, catalog number: 610-06). Briefly, 2.5 ug total RNA in 50ul DEPC-treated H_2_O was denatured at 65^o^C for 5 minutes to disrupt the secondary structure, and immediately cooled on ice for 1 minute. 100 ul of oligo (dT)_25_ Dynabeads was washed and resuspended in 50 ul of binding buffer before use. The denatured total RNA was added into prewashed Dynabeads and incubated at room temperature for 5 minutes on a rotary shaker. mRNA-Bead complex was captured on magnet rack and washed twice with 200 ul washing buffer. The mRNA was eluted by adding 11 ul of H_2_O to mRNA-Bead complex followed by heated to 75^o^C for 2 minutes. Tube was placed on magnet rack immediately for 30 seconds. The supernatant containing the purified mRNA was transfer to a fresh RNAse-free PCR tube while the tube was on magnet rack.

The first strand cDNA was reverse transcribed from poly-A mRNA using random hexamer and SuperScript® First-Strand Synthesis kit (Life Tech, catalogue number: 11904-018). Random hexamer was annealed to mRNA by heating mRNA/random hexamer mixture to at 65^o^C for 5 minutes then cooled on ice. 8.5 ul of The first strand synthesis reaction mix containing 500 uM dNTP, 20 units RNaseOut, 10 mM DTT, 200 unit Superscript II as added to mRNA/random hexamer. The first strand cDNA was synthesized by incubation reaction at 25^o^C 10 minutes, 42^o^C 60 minutes, 70^o^C 15 minutes and hold at 4^o^C. The second strand cDNA was synthesized using DNA polymerase I (life Tech, catalog number: 18010-025). The second strand cDNA synthesis was incubated at 16^o^C for 2 hrs in a thermocycler. The double strand cDNA was purified with 1.8X Agencourt AMPure XR beads (Beckman coulter, catalogue number: A63882 ).

Double stranded cDNA was constructed into Illumina paired-end libraries according to the manufacturer’s protocol (Illumina Inc.). Double strand cDNA was sheared to fragments of approximately 400 bp with the Covaris S2 or E210 system (Covaris, Inc. Woburn, MA). The setting was 10% Duty cycle, Intensity of 4,200 Cycles per Burst, for 55 seconds. Fragments were processed through DNA End-Repair in 100ul containing sheared DNA, 10ul 10X buffer, 5ul End­Repair Enzyme Mix and H2O (NEBNext End-Repair Module; Cat. No. E6050L) at 20°C for 30 minutes; A-tailing was performed in 50ul containing End-Repaired DNA, 5ul 10X buffer, 3ul Klenow Fragment (NEBNext dA-Tailing Module; Cat. No. E6053L) at 37°C for 30 minutes, each step followed by purification using a QIAquick PCR purification kit (Cat. No. 28106). Resulting fragments were ligated with Illumina PE adapters and the NEBNext Quick Ligation Module (Cat. No. E6056L). After ligation, size selection was carried out by using 2% low-melt agarose gel running in 1X TBE. Gel slices were excised from 290bp to 340bp and the size-selected DNA was purified using a Qiagen MinElute gel extraction kit and eluted in 30ul EB buffer. PCR with Illumina PE 1.0 and 2.0 primers was performed in 25-μl reactions containing 12.5 ul of 2x Phusion High-Fidelity PCR master mix, 2.5ul size-selected fragment DNA, 0.3ul each primer and H2O. The standard thermocycling for PCR was 30 s at 98°C for the initial denaturation followed by 12 cycles of 10 s at 98°C, 30 s at 65°C and 30 s at 72°C and a final extension of 5 min. at 72°C. 1.8X Agencourt® XP® Beads (Beckman Coulter Genomics, Inc.; Cat. No. A63882) were used to purify the PCR products. After Bead purification, PCR products were quantified using PicoGreen (Cat. No. P7589) and their size distribution analyzed using the Agilent Bioanalyzer 2100 DNA Chip 7500 (Cat. No. 5067-1506). Then, 15ul of 10nM final library was sequenced on Illumina’s Genome Analyzer IIx system according to the manufacturer’s specifications. Briefly, cluster generations were performed on an Illumina cluster station. 36-76 cycles of sequencing were carried out with each library in a separate, single flow cell lane on the Illumina GA II. Sequencing analysis was first done with Illumina analysis pipeline. Sequencing image files were processed to generate base calls and phred-like base quality scores and to remove low-quality reads.

**15. References.**

1. Gregory TR (2014) Animal Genome Size Database. <http://www.genomesize.com>.

2. Cantarel BL, Korf I, Robb SMC, Parra G, Ross E, et al. (2008) MAKER: An easy-to-use annotation pipeline designed for emerging model organism genomes. Genome Res 18: 188-196.

3. Megy K, Emrich SJ, Lawson D, Campbell D, Dialynas E, et al. (2012) VectorBase: improvements to a bioinformatics resource for invertebrate vector genomics. Nuc Acid Res 40: D729-D734.

4. Parra G, Bradnam K, Korf I (2007) CEGMA: a pipeline to accurately annotate core genes in eukaryotic genornes. Bioinformatics 23: 1061-1067.

5. Flutre T, Duprat E, Feuillet C, Quesneville H (2011) Considering transposable element diversification in de novo annotation approaches. PLoS ONE, 6:e16526.

6. Jurka J, Kapitonov VV, Pavlicek A, Klonowski P, Kohany O, et al. (2005) Repbase Update, a database of eukaryotic repetitive elements. Cytogenetic and Genome Research 110: 462–467.

7. Punta M, Coggill PC, Eberhardt RY, Mistry J, Tate J, et al. (2012) The Pfam protein families database. Nucleic Acids Res 40: D290–301.

8. Wicker T, Sabot F, Hua-Van A, Bennetzen JL, Capy P, et al. (2007) A unified classification system for eukaryotic transposable elements. Nature Reviews Genetics 8: 973–982.

9. Marchler-Bauer A, Anderson JB, Derbyshire MK, DeWeese-Scott C, Gonzales NR, et al. (2007) CDD: a conserved domain database for interactive domain family analysis. Nucleic Acids Res 35: D237–240.

10. Kohany O, Gentles AJ, Hankus L, Jurka J (2006) Annotation, submission and screening of repetitive elements in Repbase: RepbaseSubmitter and Censor. BMC Bioinformatics 7:474.

11. Kapitonov V, Tempel S, Jurka J (2009) Simple and fast classification of non-LTR retrotransposons based on phylogeny of their RT domain protein sequences. Gene 448: 207–213.

12. Yuan Y-W, Wessler SR (2011) The catalytic domain of all eukaryotic cut-and-paste transposase superfamilies. Proc Natl Acad Sci USA 108: 7884–7889.

13. Kapitonov VV, Jurka J (2008) A universal classification of eukaryotic transposable elements implemented in Repbase. Nature Reviews Genetics 9: 411–412; author reply 414.

14. Suen G, Teiling C, Li L, Holt C, Abouheif E, et al.(2011) The genome sequence of the leaf-cutter ant *Atta cephalotes* reveals insights into its obligate symbiotic lifestyle. PLoS Genetics 7:e1002007.

15. Werren JH, Richards S, Desjardins C, Niehuis O, Gadau J, et al. (2010) Functional and evolutionary insights from the genomes of three parasitoid *Nasonia* species. Science 327: 343–348.

16. Smith CR, Smith CD, Robertson HM, Helmkampf M, Zimin A, et al. (2011) Draft genome of the red harvester ant *Pogonomyrmex barbatus*. Proc Natl Acad Sci USA 108: 5667–5672.

17. Nygaard S, Zhang G, Schiøtt M, Li C, Wurm Y, et al. (2011) The genome of the leaf-cutting ant *Acromyrmex echinatior* suggests key adaptations to advanced social life and fungus farming. Genome Res 21: 1339–1348.

18. Bonasio R, Zhang G, Ye C, Mutti NS, Fang X, et al. (2010) Genomic comparison of the ants *Camponotus floridanus* and *Harpegnathos saltator*. Science 329: 1068–1071.

19. Smith CD, Zimin A, Holt C, Abouheif E, Benton R, et al. (2011) Draft genome of the globally widespread and invasive Argentine ant (*Linepithema humile*). Proc Natl Acad Sci USA 108: 5673–5678.

20. Wurm Y, Wang J, Riba-Grognuz O, Corona M, Nygaard S, et al. (2011) The genome of the fire ant *Solenopsis invicta*. Proc Natl Acad Sci USA 108: 5679–5684.

21. The International Aphid Genomics Consortium (2010) Genome sequence of the pea aphid *Acyrthosiphon pisum*. PLoS Biology 8:e1000313.

22. Holt RA, Subramanian GM, Halpern A, Sutton GG, Charlab R, et al. (2002) The genome sequence of the malaria mosquito *Anopheles gambiae*. Science 298: 129-149.

23. Arensburger P, Megy K, Waterhouse RM, Abrudan J, Amedeo P, et al. (2010) Sequencing of *Culex quinquefasciatus* establishes a platform for mosquito comparative genomics. Science 330: 86-88.

24. Xia Q, Zhou Z, Lu C, Cheng D, Dai F, et al. (2004) A draft sequence for the genome of the domesticated silkworm (*Bombyx mori*). Science 306: 1937-1940.

25. Marinotti O, Cerqueira GC, de Almeida LG, Ferro MI, Loreto EL, et al. (2013) The genome of *Anopheles darlingi*, the main neotropical malaria vector. Nucleic Acids Res 41(15): 7387–7400.

26. Nene V, Wortman JR, Lawson D, Haas B, Kodira C, et al. (2007) Genome sequence of *Aedes aegypti*, a major arbovirus vector. Science 316: 1718-1723.

27. Kaminker JS, Bergman CM, Kronmiller B, Carlson J, Svirskas R, et al. (2002) The transposable elements of the *Drosophila melanogaster* euchromatin: a genomics perspective. Genome Biology 3:RESEARCH0084.

28. Novick PA, Smith JD, Floumanhaft M, Ray DA, Boissinot S (2011) The evolution and diversity of DNA transposons in the genome of the Lizard *Anolis carolinensis*. Genome Biol Evol 3: 1–14.

29. Pace JK, Feschotte C (2007) The evolutionary history of human DNA transposons: evidence for intense activity in the primate lineage. Genome Res 17: 422–432.

30. Smith TF, Waterman MS. (1981) Identification of common molecular subsequences. J Mol Biol 147: 195–197.

31. Edgar RC (2004) MUSCLE: a multiple sequence alignment method with reduced time and space complexity. BMC Bioinformatics 5:113.

32. Katoh K, Toh H (2008) Recent developments in the MAFFT multiple sequence alignment program. Briefings in bioinformatics 9: 286–298.

33. Lassmann T, Frings O, Sonnhammer ELL (2009) Kalign2: high-performance multiple alignment of protein and nucleotide sequences allowing external features. Nucleic Acids Res 37: 858–865.

34. Landan G, Graur D (2007) Heads or tails: a simple reliability check for multiple sequence alignments. Mol Biol Evol 24: 1380–1383.

35. Wallace IM, O’Sullivan O, Higgins DG, Notredame C (2006) M-Coffee: combining multiple sequence alignment methods with T-Coffee. Nucl Acid Res 34: 1692–1699.

36. Capella-Gutiérrez S, Silla-Martínez JM, Gabaldón T (2009) TrimAl: a tool for automated alignment trimming in large-scale phylogenetic analyses. Bioinformatics 25: 1972–1973.

37. Gascuel O (1997) BIONJ: an improved version of the NJ algorithm based on a simple model of sequence data. Mol Biol Evol 14: 685–695.

38. Guindon S, Dufayard JF, Lefort V, Anisimova M, Hordijk W, et al. (2010) New algorithms and methods to estimate maximum-likelihood phylogenies: assessing the performance of PhyML 3.0. Syst Biol 59: 307–321.

39. Akaike H (1974) A new look at the Statistical Model Identification. IEEE Transactions on Automatic Control 19: 716–723.

40. Huerta-Cepas J, Capella-Gutierrez S, Pryszcz LP, Denisov I, Kormes D, et al. (2011) PhylomeDB v3.0: an expanding repository of genome-wide collections of trees, alignments and phylogeny-based orthology and paralogy predictions. Nucleic Acids Res 39: D556–560.

41. Gabaldón T (2008) Large-scale assignment of orthology: back to phylogenetics? Genome Biology 9:235.

42. Huerta-Cepas J, Dopazo J, Gabaldón T (2010) ETE: a python Environment for Tree Exploration. BMC Bioinformatics 11:24.

43. Huerta-Cepas J, Capella-Gutierrez S, Pryszcz LP, Marcet-Houben M, Gabaldón T, et al. (2014) PhylomeDB v4: zooming into the plurality of evolutionary histories of a genome. Nucleic Acids Res. 42: D897-902.

44. Slater GSC, Birney E (2005) Automated generation of heuristics for biological sequence comparison. BMC Bioinformatics 6(1):31.

45. Shimodaira H, Hasegawa M (2001) CONSEL: for assessing the confidence of phylogenetic tree selection. Bioinformatics 17(12): 1246.

46. Medina I, Carbonell J, Pulido L, Madeira SC, Goetz S, et al. (2010) Babelomics: an integrative platform for the analysis of transcriptomics, proteomics and genomic data with advanced functional profiling. Nucleic Acids Res 38: W210–213.

47. Supek F, Bošnjak M, Škunca N, Šmuc T (2011) REVIGO summarizes and visualizes long lists of Gene Ontology terms. PLoS ONE 6:e21800.

48. Huerta-Cepas J, Gabaldón T (2011) Assigning duplication events to relative temporal scales in genome-wide studies. Bioinformatics 27(1): 38-45.

49. Podsiadlowski L, Kohlhagen H, Koch M (2007) The complete mitochondrial genome of *Scutigerella causeyae* (Myriapoda: Symphyla) and the phylogenetic position of Symphyla. Mol Phylogenet Evol 45(1): 251-260.

50. Simakov O, Marletaz F, Cho SJ, Edsinger-Gonzales E, Havlak P, et al. (2013) Insights into bilaterian evolution from three spiralian genomes. Nature 493: 526-531.

51. Takatori N, Butts T, Candiani S, Pestarino M, Ferrier DEK, et al. (2008) Comprehensive survey and classification of homeobox genes in the genome of amphioxus, *Branchiostoma floridae*. Dev Genes Evol 218: 579-590.

52. Brooke NM, Garcia-Fernàndez J, Holland PWH (1998) The ParaHox gene cluster is an evolutionary sister of the Hox gene cluster. Nature 392: 920-922.

53. Hui JHL, Holland PWH, Ferrier DEK (2008) Do cnidarians have a ParaHox cluster? Analysis of synteny around a *Nematostella* homeobox gene cluster. Evol Dev 10: 725-730.

54. Jagla K, Bellard M, Frasch M (2001) A cluster of *Drosophila* homeobox genes involved in mesoderm differentiation programs. Bioessays 23(2): 125-133.

55. Doran Cande J, Chopra VS, Levine M (2009) Evolving enhancer-promoter interactions within the tinman complex of the flour beetle, *Tribolium castaneum.* Development 136: 3153-3160.

56. Pollard SL, Holland PWH (2000) Evidence for 14 homeobox gene clusters in human genome ancestry. Curr Biol 10(17): 1059-1062.

57. Garcia-Fernàndez J (2005) The genesis and evolution of homeobox gene clusters. Nature Reviews Genetics 6: 881-892.

58. Irimia M, Maeso I, Garcia-Fernàndez J (2008) Convergent evolution of clustering of Iroquois homeobox genes across metazoans. Mol Biol Evol 8: 1521-1525.

59. Kerner P, Ikmi A, Coen D, Vervoort M (2009) Evolutionary history of the Iroquois/Irx genes in metazoans. BMC Evol Biol 9:74.

60. Mazza ME, Pang K, Reitzel AM, Martindale MQ, Finnerty JR (2010) A conserved cluster of three PRD-class homeobox genes (*homeobrain, rx* and *orthopedia*) in the Cnidaria and Protostomia. EvoDevo 1: 3.

61. Butts T, Holland PWH, Ferrier DEK (2008) The Urbilaterian Super-Hox cluster. Trends in Genetics 24: 259-262.

62. Hui JHL, McDougall C, Monteiro AS, Holland PWH, Arendt D, et al. (2012) Extensive chordate and annelid macrosynteny reveals ancestral homeobox gene organization. Mol Biol Evol 29: 157-165.

63. Akam ME, Martinez-Arias A (1985) The distribution of Ultrabithorax transcripts in *Drosophila* embryos. EMBO J 4: 1689-1700.

64. Celniker SE, Keelan DJ, Lewis EB (1989) The molecular genetics of the bithorax complex of *Drosophila*: characterization of the products of the Abdominal-B domain. Genes Dev 3: 1424-1436.

65. Celniker SE, Sharma S, Keelan DJ, Lewis EB (1990) The molecular genetics of the bithorax complex of *Drosophila*: cis-regulation in the Abdominal-B domain. EMBO J 9: 4277-4286.

66. Kornfeld K, Saint RB, Beachy PA, Harte PJ, Peattie DA et al. (1989) Structure and expression of a family of Ultrabithorax mRNAs generated by alternative splicing and polyadenylation in *Drosophila*. Genes Dev 3: 243-258.

67. Kuziora MA, McGinnis W (1988) Different transcripts of the *Drosophila Abd-B* gene correlate with distinct genetic sub-functions. EMBO J 7: 3233- 3244.

68. Laughon A, Boulet AM, Bermingham JR, Laymon RA, Scott MP (1986) Structure of transcripts from the homeotic *Antennapedia* gene of *Drosophila melanogaster*: two promoters control the major protein-coding region. Mol Cell Biol 6: 4676-4689.

69. O’Connor MB, Binari R, Perkins LA, Bender W (1988) Alternative RNA products from the Ultrabithorax domain of the bithorax complex. EMBO J 7: 435-445.

70. Rowe A, Akam ME (1988) The structure and expression of a hybrid homeotic gene. EMBO J 7: 1107-1114.

71. Sanchez-Herrero E, Crosby MA (1988) The *Abdominal-B* gene of *Drosophila melanogaster*: overlapping transcripts exhibit two different spatial distributions. EMBO J 7: 2163-2173.

72. Schneuwly S, Kuroiwa A, Baumgartner P, Gehring WJ (1986) Structural organization and sequence of the homeotic gene *Antennapedia* of *Drosophila melanogaster*. EMBO *.* 5: 733-739.

73. Scott MP, Weiner AJ, Hazelrigg TI, Polisky BA, Pirrotta V, et al. (1983) The molecular organization of the Antennapedia locus of *Drosophila*. Cell 35: 763-776.

74. Stroeher VL, Jorgensen EM, Garber RL (1986) Multiple transcripts from the *Antennapedia* gene of *Drosophila melanogaster*. Mol Cell Biol 6: 4667-4675.

75. Tyler DM, Okamura K, Chung WJ, Hagen JW, Berezikov E, et al. (2008) Functionally distinct regulatory RNAs generated by bidirectional transcription and processing of microRNA loci. Genes Dev 22: 26-36.

76. Subramaniam V, Bomze HM, López AJ. (1994) [Functional differences between Ultrabithorax protein isoforms in Drosophila melanogaster: evidence from elimination, substitution and ectopic expression of specific isoforms.](http://www.ncbi.nlm.nih.gov/pubmed/7911774) Genetics 136: 979-991.

77. Reed HC, Hoare T, Thomsen S, Weaver TA, White RAH, et al. (2010) Alternative splicing modulates Ubx protein function in *Drosophila melanogaster****.*** Genetics 184: 745-758.

78. Thomsen S, Azzam G, Kaschula R, Williams LS, Alonso CR (2010) Developmental RNA processing of 3'UTRs in Hox mRNAs as a context-dependent mechanism modulating visibility to microRNAs. Development 137: 2951-2960.

79. De Navas LF, Reed HC, Akam ME, Barrio R, Alonso CR, et al. (2011) Integration of RNA processing and expression level control modulates the functions of the *Drosophila* Hox gene *Ultrabithorax* during adult development. Development 138: 107-116.

80. Venables JP, Tazi J, Juge F (2012) [Regulated functional alternative splicing in *Drosophila*](http://www.ncbi.nlm.nih.gov/pmc/articles/PMC3245913/). Nucleic Acids Res. 40(1): 1–10.

81. Brena C, Chipman AD, Minelli A, Akam ME (2006) Expression of trunk Hox genes in the centipede *Strigamia maritima*: sense and anti-sense transcripts. Evol Dev 8: 252–265.

82. Abascal F, Zardoya R (2013) Evolutionary analyses of gap junction protein families. Biochim Biophys Acta 1828: 4-14.

83. Duncan EJ, Wilson MJ, Smith JM, Dearden PK (2008) Evolutionary origin and genomic organisation of runt-domain containing genes in arthropods. BMC Genomics 9: 558.

84. Duncan EJ, Dearden PK (2010) Evolution of a genomic regulatory domain: The role of gene co-option and gene duplication in the Enhancer of Split Complex. Genome Res 20: 917-928.

85. Su CY, Menuz K, Carlson JR (2009) Olfactory perception: receptors, cells, and circuits. Cell 139: 45-59.

86. Touhara K, Vosshall LB (2009) Sensing odorants and pheromones with chemosensory receptors. Annu Rev Physiol 71: 307-332.

87. Jones WD, Cayirlioglu P, Kadow IG, Vosshall LB (2007) Two chemosensory receptors together mediate carbon dioxide detection in *Drosophila*. Nature 445: 86-90.

88. Penalva-Arana DC, Lynch M, Robertson HM (2009) The chemoreceptor genes of the water flea *Daphnia* *pulex*: many GRs but no ORs. BMC Evol Biol 9: e79.

89. Larkin MA, Blackshields G, Brown NP, Chenna R, McGettigan PA, et al. (2007) Clustal W and Clustal X version 2.0. Bioinformatics 23: 2947-2948.

90. Kent LB, Robertson HM (2009) Evolution of the sugar receptors in insects. BMC Evol Biol 9: e41.

91. Robertson HM, Kent LB (2009) Evolution of the gene lineage encoding the carbon dioxide heterodimeric receptor in insects. J Insect Sci 9: e19.

92. Schmidt HA, Strimmer K, Vingron M, von Haeseler A (2002) TREE-PUZZLE: maximum likelihood phylogenetic analysis using quartets and parallel computing. Bioinformatics. 18: 502-504.

93. Swofford DL (2003) PAUP*. Phylogenetic Analysis Using Parsimony (*and Other Methods). Version 4. Sinauer Associates, Sunderland, Massachusetts.

94. Robertson HM, Warr CG, Carlson JR (2003) Molecular evolution of the insect chemoreceptor gene superfamily in *Drosophila melanogaster.* Proc Natl Acad Sci U S A 100 Suppl 2: 14537-14542.

95. Miyamoto T, Slone J, Song X, Amrein H (2012) A fructose receptor functions as a nutrient sensor in the *Drosophila* brain. Cell 151: 1113-1125.

96. Fredriksson R, Schiöth HB (2005) The repertoire of G-protein-coupled receptors in fully sequenced genomes. Mol Pharmacol 67: 1414-1425.

97. Hauser F, Cazzamali G, Williamson M, Park Y, Li B, et al. (2008) A genome-wide inventory of neurohormone GPCRs in the red flour beetle *Tribolium castaneum*. Front Neuroendocrin 29: 142-165.

98. Konopova and Jindra (2008) Broad-Complex acts downstream of Met in juvenile hormone signalling to coordinate primitive holometabolan metamorphosis. Development 135: 559-568.

99. Li Y, Zhang Z, Robinson GE, Palli SR (2007) Identification and characterization of a Juvenile Hormone response element and its binding proteins. J Biol Chem 282: 37605-37617.

100. Zhou X, Tarver MR, Scharf ME (2007) Hexamerin-based regulation of juvenile hormone-dependent gene expression underlies phenotypic plasticity in a social insect. Development 134: 601-610.

101. Wainwright G, Webster SG, Wilkinson MC, Chung JS, Rees HH (1996) Structure and significance of mandibular organ-inhibiting hormone in the crab, *Cancer pagurus*. Involvement in multihormonal regulation of growth and reproduction. J Biol Chem 271: 12749-12754.

102. Van der Zee M, da Fonseca RN, Roth S (2008) TGFbeta signaling in *Tribolium*: vertebrate-like components in a beetle. Dev Genes Evol 218: 203-213.

103. Lowery JW, Lavigne AW, Kokabu S, Rosen V (2013) Comparative genomics identifies the mouse Bmp3 promoter and an upstream evolutionary conserved region (ECR) in mammals. PLoS ONE 8: e57840.

104. Fritsch C, Lanfear R, Ray RP (2010) Rapid evolution of a novel signalling mechanism by concerted duplication and divergence of a BMP ligand and its extracellular modulators. Dev Genes Evol 220: 235-250.

105. Wotton KR, Alcaine Colet A, Jaeger J, Jimenez-Guri E (2013) Evolution and expression of BMP genes in flies. Dev Genes Evol 223: 335-340.

106. Lifton RP, Goldberg ML, Karp RW, Hogness DS (1977) Organization of histone genes in *Drosophila melanogaster* - functional and evolutionary implications. Cold Spring Harb Sym 42: 1047-1051.

107. Matsuo Y, Yamazaki T (1989) Transfer-RNA derived insertion element in histone gene repeating unit of *Drosophila melanogaster*. Nucleic Acids Res 17: 225-238.

108. Lyko F, Foret S, Kucharski R, Wolf S, Falckenhayn C, et al. (2010) The honey bee epigenomes: differential methylation of brain DNA in queens and workers. PLoS Biology 8: e1000506.

109. Rider SD, Srinivasan DG, Hilgarth RS (2010) Chromatin-remodelling proteins of the pea aphid, *Acyrthosiphon pisum* (Harris). Insect Mol Biol 19: 201-214.

110. Millar CB (2013) Organizing the genome with H2A histone variants. The Biochemical Journal 449: 567-579.

111. Jayaramaiah Raja S, Renkawitz-Pohl R (2005) Replacement by *Drosophila melanogaster* protamines and Mst77F of histones during chromatin condensation in late spermatids and role of sesame in the removal of these proteins from the male pronucleus. Mol Cell Biol 25: 6165-6177.

112. Mizzen CA, Yang XJ, Kokubo T, Brownell JE, Bannister AJ, et al. (1996) The TAF(II)250 subunit of TFIID has histone acetyltransferase activity. Cell 87: 1261-1270.

113. Matthias P, Yoshida M, Khochbin S (2008) HDAC6 a new cellular stress surveillance factor. Cell Cycle 7: 7-10.

114. Feil R, Fraga MF (2011) Epigenetics and the environment: emerging patterns and implications. Nature Reviews Genetics 13: 97-109.

115. Nagel S, Grossbach U (2000) Histone H1 genes and histone gene clusters in the genus *Drosophila*. J Mol Evol 51: 286-298.

116. Fraley C, Raftery AE (2003) Enhanced model-based clustering, density estimation, and discriminant analysis software: MCLUST. J Classif 20(2):263-286.

117. Kozomara A, Griffiths-Jones S (2011) miRBase: integrating microRNA annotation and deep-sequencing data. Nucleic Acids Res. 39: D152-D157.

118. Nawrocki EP, Kolbe DL, Eddy SR (2009) Infernal 1.0: inference of RNA alignments. Bioinformatics 25: 1335-1337.

119. Guerra-Assunção JA, Enright AJ (2010) MapMi: automated mapping of microRNA loci. BMC bioinformatics 11: 133.

120. Griffiths-Jones S (2005) RALEE--RNA ALignment editor in Emacs. Bioinformatics 21: 257-259.

121. Lowe TM, Eddy SR (1997) tRNAscan-SE: a program for improved detection of transfer RNA genes in genomic sequence. Nucleic Acids Res 25: 955-964.

122. Gardner PP (2009) The use of covariance models to annotate RNAs in whole genomes. Briefings in functional genomics & proteomics 8: 444-450.

123. Altschul SF, Gish W, Miller W, Myers EW, Lipman DJ (1990) Basic local alignment search tool. J Mol Biol 215: 403-410.

124. Rota-Stabelli O, Campbell L, Brinkmann H, Edgecombe GD, Longhorn SJ, et al. (2011) A congruent solution to arthropod phylogeny: phylogenomics, microRNAs and morphology support monophyletic Mandibulata. Proc Roy Soc Biol Sci 278: 298-306.

**16. Supporting Information Figure Legends**

**Figure S1. Frequency histogram showing the distribution of gene lengths in the *S. maritima* genome.**

Gene length data used in this plot are available in File S4.

**Figure S2. Multi-gene phylogeny for the 18 species included in the phylogenomics analysis.**

1,491 widespread single-copy sets of orthologue sequences in at least 15 out of the 18 species were concatenated into a single alignment of 842,150 columns. Then, a maximum-likelihood tree was inferred using LG as evolutionary model by using PhyML.

**Figure S3. Multi-gene phylogeny for 12 species included in the phylogenomics analysis plus 5 additional Chelicerata species.**

1,491 widespread single-copy sets of orthologue sequences were concatenated into a single alignment of 829,729 positions. Then, a maximum-likelihood tree was inferred using LG as the evolutionary model by using PhyML.

**Figure S4. Alternative topological placements of *S. maritima* relative to the main arthropod groups considered in the study: Chelicerata and Pancrustacea.**

Internal organization of each group was initially collapsed and, therefore, optimized during Maximum-Likelihood reconstruction.

**Figure S5**. **Clusters of genes specifically expanded in the centipede lineage.**

On the plot, only clusters grouping 5 or more protein-coding genes were considered. The data underlying this plot is available in File S4.

**Figure S6. Mitochondrial gene organisation.**

Shaded regions represent differences from the ground pattern. Gene translocations in Myriapoda have been noted in *Scutigerella causeyae* (Myriapoda: Symphyla) [49]. The previous example of the small conserved region trnaF-nad5-H-nad4-nad4L on the minus strand between *Limulus, Lithobius and Strigamia* is not a conserved feature in all Chilopoda, because *Scutigera colepotrata* have an interruption between nad5 and H-nad4 with elements on the minus and plus strands accompanied by a translocation of nad4L to a position immediately preceding nad5.

**Figure S7. Classification of all *S. maritima* (Sma) homeodomains (excluding Pax2/5/8/sv) via phylogenetic analysis using *T. castaneum* (Tca) and *B. floridae* (Bfl) homeodomains.**

This phylogenetic analysis was constructed using Neighbour-Joining with a JTT distance matrix and 1000 bootstrap replicates. Gene classes are indicated by colours. The genes coloured in grey are those genes that cannot be assigned to known classes. Further classification was performed using additional domains outside the homeodomain and by performing additional phylogenetic analysis for particular gene classes using maximum-likelihood and bayesian approaches. Pax2/5/8/sv is excluded due to the gene possessing only a partial homeobox.

**Figure S8. Phylogenetic analysis of ANTP class homeodomains of *S. maritima* (Sma) using *T. castaneum* (Tca) and *B. floridae* (Bfl) for comparison.**

These phylogenetic analyses were constructed using Neighbour-Joining with a JTT distance matrix, 1000 bootstrap replicates (support values in black). Nodes with support equal to or above 500 in the maximum-likelihood (LG+G) analysis are in blue and nodes with posterior probabilities equal to or above 0.5 (LG+G) in the Bayesian analysis are in red.

**Figure S9. Phylogenetic analysis of PRD class homeodomains of *S. maritima* (Sma) using *T. castaneum* (Tca) and *B. floridae* (Bfl) for comparison.**

These phylogenetic analyses were constructed using Neighbour-Joining with a JTT distance matrix, 1000 bootstrap replicates (support values in black). Nodes with support equal to or above 500 in the maximum-likelihood (LG+G) analysis are in blue and nodes with posterior probabilities equal to or above 0.5 (LG+G) in the Bayesian analysis are in red.

**Figure S10. Phylogenetic analysis of HNF class homeodomains of *S. maritima* (Sma) using *B. floridae* (Bfl)*,* human (*Homo sapiens*, Hsa) and sea anemone (*Nematostella vectensis*, Nve) for comparison.**

These phylogenetic analyses were constructed using Neighbour-Joining with a JTT distance matrix, 1000 bootstrap replicates (support values in black). Nodes with support equal to or above 500 in the maximum-likelihood (LG+G) analysis are in blue and nodes with posterior probabilities equal to or above 0.5 (LG+G) in the Bayesian analysis are in red.

**Figure S11. Phylogenetic analysis of Xlox/Hox3 genes of *S. maritima* (Sma) using a selection of Hox1, Hox2, Hox3, Hox4 and Xlox sequences.**

This analysis was based upon the whole coding sequence of the genes, and was constructed using Neighbour-Joining with a JTT distance matrix and 1000 bootstrap replicates . The blue support value (of 333) is the node that reveals the affinity between the Xlox/Hox3 genes of *S. maritima* and Xlox sequences. Ame = *Apis mellifera*, Bfl = *Branchiostoma floridae*, Cte = *Capitella teleta*, Dme = *Drosophila melanogaster*, Lgi = *Lottia gigantea* and Tca = *Tribolium castaneum*.

**Figure S12. Multiple alignment of relevant residues of the Hox1, Hox2, Hox3, Hox4 and Xlox sequences of different lineages compared to *S. maritima* Hox3a and Hox3b sequences.**

Three Paired class genes are included as an outgroup. The grading of purple colouring of the amino acids shows the identity level of these sequences. The red rectangles in the multiple alignment delimit the core of the hexapeptide motif and the homeodomain. This is the alignment used to construct the phylogenetic tree in Figure S13. Ame = *Apis mellifera*, Bfl = *Branchiostoma floridae*, Cte = *Capitella teleta*, Dme = *Drosophila melanogaster*, Lgi = *Lottia gigantea* and Tca = *Tribolium castaneum*.

**Figure S13. Phylogenetic analysis of *S. maritima* Xlox/Hox3 homeodomain and hexapeptide motifs using a selection of Hox1, Hox2, Hox3, Hox4 and Xlox sequences.**

This analysis used a section of the coding sequence including the hexapeptide and some flanking residues plus the homeodomain (alignment in Figure S12). Three Paired class genes are included as an outgroup. This phylogeny was constructed using Neighbor-Joining with the JTT distance matrix and 1000 bootstrap replicates. Maximum Likelihood support values are shown in blue and Bayesian posterior probabilities in red. Ame = *Apis mellifera*, Bfl = *Branchiostoma floridae*, Cte = *Capitella teleta*, Dme = *Drosophila melanogaster*, Lgi = *Lottia gigantea* and Tca = *Tribolium castaneum*.

**Figure S14. Fisher’s Exact Test to distinguish whether *S. maritima* scaffold 48457 has significant synteny conservation with ParaHox or Hox chromosomes of humans.**

No significant Hox or ParaHox association is found.

**Figure S15. Phylogenetic analysis of TALE class homeodomains of *S. maritima* (Sma) using *T. castaneum* (Tca) and *B. floridae* (Bfl)*,* including the Iroquois/Irx genes.**

These phylogenetic analyses were constructed using Neighbour-Joining with a JTT distance matrix, 1000 bootstrap replicates (support values in black). Nodes with support equal to or above 500 in the maximum-likelihood (LG+G) analysis are in blue and nodes with posterior probabilities equal to or above 0.5 (LG+G) in the Bayesian analysis are in red.

**Figure S16.** **RNA processing in the Hox cluster of *S. maritima.***

The transcriptome of *S. maritima (Sm)* eggs (blue), females (green) and males (red) was mapped to the Hox gene cluster (top panel – see Figure 4 in the main text) and transcript models were inferred for each gene within the cluster (shaded area) taking into account the presence of open-reading frames (ORF) and polyadenylation signals (PAS) to support the existence of RNA processing events. We note the occurrence of more than one mRNA isoform of six *S. maritima* Hox genes (i.e. *Antp, Ubx, abd-A, lab, Dfd, pb*). In all these six cases alternative polyadenylation (APA) generates mRNAs bearing distinct 3’ untranslated regions (UTR; alternative UTR sizes at the bottom). Alternative splicing (AS) with concomitant alternative promoter use (APU) events concern two *S. maritima* Hox genes *Dfd* and *ftz* (see alternative ORF sizes at the bottom). We also see that some genes such as *S. maritima Ubx* display high heterogeneity in 3’UTR sequences within the embryonic transcriptome (“eggs” data) suggesting the possibility that *S. maritima Ubx* APA might be developmentally controlled and/or display a tissue-specific pattern (see inset for further details on symbols).

**Figure S17. RNA processing in the *S. maritima* and *D. melanogaster* Hox clusters.**

A) The incidence of alternatively processed mRNAs is comparable between *S. maritima* and *D. melanogaster*, in that over 75% of the *S. maritima* Hox genes undergo RNA processing of one type or another. Similarly, seven out of the eight *Drosophila* Hox genes produce different mRNA isoforms (FlyBase, <http://flybase.org/>). B) Three *D. melanogaster* Hox genes undergo AS (blue) and five produce different transcripts via APA (red, FlyBase <http://flybase.org/>). In addition 5 fruit fly Hox genes form different RNA species by APU (green). C) Classification of all alternatively processed mRNA events in the *S. maritima* Hox cluster based on the same categorisation as in B). Note that patterns of AS and APA affecting *S. maritima* and *D. melanogaster* Hox genes are relatively comparable; in contrast, APU seems more prevalent in the *Drosophila* (5 out if 8 genes) than in the centipede (2 out of 9 genes) Hox genes.

**Figure S18. Phylogenetic tree of the *S. maritima, D. pulex, I. scapularis*, and representative insect GRs, part one.**

This is a corrected distance tree and was rooted at the midpoint in the absence of a clear outgroup, an approach that clearly indicates the distinctiveness of the centipede GRs. It is a more detailed version of Figure 5A. The *S. maritima, D. pulex, I. scapularis*, and representative insect gene/protein names are highlighted in red, blue, brown, and green, respectively, as are the branches leading to them to emphasize gene lineages. Bootstrap support levels in percentage of 10,000 replications of neighbour-joining with uncorrected distance is shown above major branches. Comments on major gene lineages are on the right. Suffixes after the gene/protein names are: PSE – pseudogene; FIX – sequence fixed with raw reads; JOI – gene model joined across scaffolds. Note than in Figure 5A for space reasons the IsGr47 and 59 proteins are included in the carbon dioxide and sugar receptor groupings, respectively, however there is no bootstrap support for these branches, and no such functional assignment is claimed. Similarly, it is unlikely that the DpGr57/58 proteins are fructose receptors.

**Figure S19. Phylogenetic tree of the *S. maritima, D. pulex, I. scapularis*, and representative insect GRs, part two.**

This is a corrected distance tree and was rooted at the midpoint in the absence of a clear outgroup, an approach that clearly indicates the distinctiveness of the centipede GRs. It is a more detailed version of Figure 5A. The *S. maritima, D. pulex, I. scapularis*, and representative insect gene/protein names are highlighted in red, blue, brown, and green, respectively, as are the branches leading to them to emphasize gene lineages. Bootstrap support levels in percentage of 10,000 replications of neighbour-joining with uncorrected distance is shown above major branches. Comments on major gene lineages are on the right. Suffixes after the gene/protein names are: PSE – pseudogene; FIX – sequence fixed with raw reads; JOI – gene model joined across scaffolds. Note than in Figure 5A for space reasons the IsGr47 and 59 proteins are included in the carbon dioxide and sugar receptor groupings, respectively, however there is no bootstrap support for these branches, and no such functional assignment is claimed. Similarly, it is unlikely that the DpGr57/58 proteins are fructose receptors.

**Figure S20. Neuropeptide precursor sequences identified in the *S. maritima* genome.**

The putative signal peptides (predicted by SignalP) are underlined, the putative active neuropeptides or protein hormones (based on similarity to neuropeptides or protein hormones identified in other invertebrates) are marked in yellow. Green indicates putative basic cleavage sites flanking the putative neuropeptides. Glycines used for amidation are shown in blue, cysteines proposed to form cysteine bridges are shown in red. Dots indicate missing N- or C-termini.

**Figure S21. Examples of tandem duplications of neuropeptide receptor genes.**

Structure of the two inotocin receptor genes found head-to-head on opposite strands of scaffold JH431865 (A). Structure of the two SIFamide receptor genes found tail-to-head on the same strand of scaffold JH432116 (B).

**Figure S22.** **Schematic diagram showing sesquiterpenoids/juvenoids synthesis (upper) and degradation (lower) pathways in arthropods.**

Molecules/hormones in synthesis are shown in **bold**, enzymes are shown in *italics,* and species/clades are shown in ***bold italics.***

**Figure S23. Phylogenetic analysis of the Transforming Growth Factorβ (TGFβ) ligands in Arthropods.**

See Text S1 for details. Abbreviations: Is=*Ixodes scapularis*; Dp=*Daphnia pulex*; Ap=*Acyrthosiphon pisum*; Ph=*Pediculus humanus*; Nv=*Nasonia vitripennis*; Am=*Apis mellifera*; Tc=*Tribolium castaneum*; Ag=*Anopheles gambiae*; Dm=*Drosophila melanogaster*; Ca=*Clogmia albipunctata*; Ma=*Megaselia abdita*; Lg=*Lottia gigantea*.

**Figure S24. Range of Wnt genes present in *S. maritima*.**

Wnt genes present and number of *Wnt* subfamilies absent in *S. maritima* in comparison with other arthropods and three non-arthropod outgroups.

**Figure S25. Phylogeny of FGF receptor (FGFR) genes indicating that FGFR genes duplicated independently in *S. maritima* and *D. melanogaster*.**

See text for details. Alignment was performed using Clustal-Omega ([http://www.ebi.ac.uk/Tools/services/web](https://unimail.st-andrews.ac.uk/owa/redir.aspx?C=abRjuJIguU6-17tyTEJRcKRD7s684dAI8YKmOd9sBcwlYmeeAhdMwbnlYmCdqRISbRh3pcsGoaY.&URL=http%3a%2f%2fwww.ebi.ac.uk%2fTools%2fservices%2fweb)). The evolutionary history was inferred using the Neighbor-Joining method with bootstrapping to determine node support values (10000 replicates). The evolutionary distances were computed using the Poisson correction method. Evolutionary analyses were conducted in MEGA5.

**Figure S26. Phylogeny including the three FGF genes of *S. maritima*.**

See text for details. Alignment was performed using Clustal-Omega ([http://www.ebi.ac.uk/Tools/services/web](https://unimail.st-andrews.ac.uk/owa/redir.aspx?C=abRjuJIguU6-17tyTEJRcKRD7s684dAI8YKmOd9sBcwlYmeeAhdMwbnlYmCdqRISbRh3pcsGoaY.&URL=http%3a%2f%2fwww.ebi.ac.uk%2fTools%2fservices%2fweb)). The evolutionary history was inferred using the Neighbor-Joining method with bootstrapping to determine node support values (10000 replicates). The evolutionary distances were computed using the Poisson correction method. Evolutionary analyses were conducted in MEGA5.

**Figure S27. *Cap ‘n’ collar (cnc)* genes.**

A) The two genes are located on different scaffolds. *Cnc1* is a long transcript consisting of 11 exons. *Cnc2* is shorter (eight exons), the three exons at the 3’ end of the gene that encode the C-terminal region of the protein including the conserved domain (B) show a similar structure. (B) *S. maritima* Cnc protein structure. Both proteins contain the bZip domain in a similar position at the C-terminus. *Cnc1* encodes a long protein (925 amino acids). Bits of the N-terminal region (blue lines) align with *D. melanogaster* Cnc isoform C and *T. castaneum* Cnc variant A. (C) Cnc protein sequence alignment, only showing the aligning bits in the N-terminal region. Blue lines show short stretches of sequence that form a consensus motif. These motifs are not present in the proteins encoded by *Sm-cnc2, Dm-cnc* isoforms A and B, and *T. castaneum cnc* variant B.

**Figure S28. Frequency histograms of observed versus expected dinucleotide content in *S. maratima* gene bodies***.*

(A – P) The y-axis depicts the number of genes with the specific dinucleotide_[o/e]_ values given on the *x*-axis. The distribution of all dinucleotide pairs, with the exception of CpG, is best described as a unimodal distribution. The distribution of CpG dinucleotides is best described as a trimodal distribution, with ‘high’ and ‘low’ CpG_[o/e]_ classes. The data underlying this figure is available in File S5.

**Figure S29. Frequency histogram of CpG_[o/e]_ observed in 1000 bp windows of the *S. maritima* genome*.***

The y-axis depicts the number of genes with the specific CpG_[o/e]_ values given on the *x*-axis. The distribution of CpG_[o/e]_ in *S. maritima* genome is a bimodal distribution, with a high CpG_[o/e]_ peak observed similar to that observed in the gene bodies (Figure 9). The data underlying this figure is available in File S6.

**Figure S30. Contrasting patterns of DNA methylation, as measured by over- and under-representation of CpG dinucleotides in coding regions (CpG_(o/e)_), within arthropod species.**

In all graphs the y-axis depicts the number of genes with the specific CpG_(o/e)_ values given on the x-axis. A) *D. melanogaster* coding regions show a unimodal peak reflective of the lack of DNA methylation in this species. B) *Apis mellifera* shows a bimodal peak consisting of genes with a lower than expected CpG_(o/e)_ (green distribution) and a higher than expected CpG_(o/e)_ (blue distribution). The presence of a bimodal distribution in this species is consistent with depletion of CpG dinculeotides in the coding regions of genes over evolutionary time as a result of DNA methylation. C) A single unimodal peak is also observed for *Tetranychus urticae,* a species that has very low levels of DNA methylation. D) The *S. maritima* distribution is best explained as a mixture of three distinct distributions that we have deemed ‘low’ (green distribution), ‘medium’ (blue distribution) and ‘high’ (grey distribution). The genes within the low distribution likely contain genes that are historically methylated, whilst the ‘high’ distribution can be explained by regions of the genome that are comparatively CpG-rich (as determined by the analysis of the *S. maritima* genome, Figure S29). The data underlying this figure is available in File S7.

**Figure S31. Chromosomal organisation of histone gene clusters in *S. maritima*.**

In insects such as *Drosophila* [115] and the pea aphid [109] histone encoding genes are present in quintet clusters, each cluster containing one gene from each of the five classes of histone. Only one such cluster could be identified in *S. maritima* (A). The other four clusters identified in the *S. maritima* genome (B-D) all consist of a 2 – 3 copies of a histone encoding gene of a single class. It is possible that these have arisen as a result of recent gene duplication.

**Figure S32. *S. maritima* *vasa* DEAD-box helicase germline gene phylogeny.**

Maximum likelihood tree of *vasa/PL10* family genes. One gene is a likely *vasa* orthologue (SMAR015390), one groups with the *PL10* family (SMAR005518), and the majority group in an apparently distinct DEAD-box-containing clade. Bootstrap values for 2000 replicates are shown at each node. Accession numbers for protein sequences are as follows: *Apis* Belle (XP_391829.3), *Apis* Vasa (NP_001035345.1), *Danio* PL10 (NP_571016.2), *Danio* Vasa (AAI29276.1), *Drosophila* Belle (NP_536783.1), *Drosophila* Vasa (NP_723899.1), *Gryllus* Vasa (BAG65665.1), *Mus* Mvh (NP_001139357.1), *Mus* PL10 (NP_149068.1), *Nasonia* Belle (XP_001605842.1), *Nasonia* Vasa (XP_001603956.2), *Nematostella* PL10 (XP_001627306.1), *Nematostella* Vasa 1 (XP_001628238.1), *Nematostella* Vasa 2 (XP_001639051.1), *Oncopeltus* Vasa (AGJ83330.1), *Parhyale* Vasa (ABX76969.1), *Tribolium* Belle (NP_001153721.1), *Tribolium* Vasa (NP_001034520.2), *Xenopus* PL10 (NP_001080283.1), *Xenopus* VLG1 (NP_001081728.1).

**Figure S33. Phylogenomic inventory of meiotic genes in arthropods.**

Red genes are specific to meiosis in model species in which functional data is available. “+” and “-“ indicate the presence and absence of orthologues respectively. Numbers indicate copy number of duplicated genes.

**Figure S34. Patterns of microRNA gain and loss across the animal kingdom with the inclusion of *S. maritima*.**

The number of microRNAs that were gained or lost at each node are shown in green and red, respectively, and names are listed below each taxon. MicroRNAs that are found in the *S.maritima* genome are in bold, and families for which more than one homologue is found are marked with an asterisk. The tree depicts the Mandibulata hypothesis rather than the Myriochelata, as in [124].

**17. Supporting Information Table Legends.**

**Table S1. Detailed overview for the repetitive elements in *S. maritima*.**

For each group the number of elements (putative families), the number of their fragments or copies in the genome, the cumulative length, the proportion of the assembly and some features are shown. This includes elements containing nested inserts of other elements (n), elements which appear to be complete (i.e. all typical structural and coding parts present, even if containing stop codons or frameshifts), elements with a RT or *Tase* domain detected (n), elements which potentially could be active as they contain an intact ORF with all the typical domains even though they could lack other structural features like terminal repeats, and elements which contain an intact ORF for the RT domain or parts of the *Tase* domain and could thus be partly active. The elements which could not be categorized or contained features of protein coding regions are shown at the bottom, whereby they probably do not belong to the transposable elements.

**Table S2**. **Set of species used in the comparative genomics analyses related to the *S. maritima* genome.**

Columns include, in this order, scientific names, the species code according to UNIPROT, the number of the longest unique transcript used in the analyses, the data source and the date in which data was retrieved.

**Table S3**. **Orthologues detected between a given species and *S. maritima*.**

First column indicates how many trees have been used to detect such orthologues. Columns “uniq” refers to the number of orthologues detected for each pair of species after removing redundancy. In one-to-many and many-to-many orthology relationships it is possible to count a given protein more than once. Regarding the ratios values, “all” column refers to the orthology ratio computed using all orthologue pairs meanwhile “uniq” refers to the ratio computed using “uniq” columns.

**Table S4**. **Orthology ratios for a given species related to *S. maritima.***

This table is similar to Table S3, but in this case orthology relationships with 10 or more proteins for any of the species are discarded in order to avoid biases introduced by species-specific gene family expansions.

**Table S5. Newly added Chelicerata species used to increase the taxon sampling for the species phylogeny.**

First column indicates the scientific species name, the second one indicates which strategy has been used to identify single copy protein-coding genes. Third column shows how many single-copy genes have been identified in each species from the initial set of 1,491 used to reconstruct the species phylogeny. Last two columns show the data source and the date on which data was retrieved.

**Table S6.** **Results after applying the different statistical tests implemented in CONSEL for the alternative placement of *S. maritima* relative to Pancrustacea and Chelicerata groups of species (as shown in Figure S4) in the context of the 18 species used for the phylogenomics analyses.** The ‘item’ column relates to Figure S4 as follows, (1) topology arrangement corresponding to Figure S4 left-hand panel, in which *S. maritima* was grouped with Chelicerata species. (2) Topology arrangement corresponding to Figure S4 central panel, in which *S. maritima* branches off before the split of Pancrustacea and Chelicerata. (3) Topology arrangement corresponding to Figure S4 right-hand panel, in which *S. maritima* was grouped with Pancrustacea species.

**Table S7.** **Results after applying the different statistical tests implemented in CONSEL for the alternative placement of *S. maritima* relative to the two arthropod groups, Pancrustacea and Chelicerata (as shown in Figure S4), with the inclusion of extra chelicerates.** Taxon sampling for the Chelicerata was increased after including sequences from 5 additional species. In order to reduce any potential bias introduced by distant and/or fast-evolving out-groups, 6 out-group species from the initial set were removed. The ‘item’ column relates to Figure S4 as follows, (1) topology arrangement corresponding to Figure S4 left-hand panel, in which *S. maritima* was grouped with Chelicerata species. (2) Topology arrangement corresponding to Figure S4 central panel, in which *S. maritima* branches off before the split of Pancrustacea and Chelicerata. (3) Topology arrangement corresponding to Figure S4 right-hand panel, in which *S. maritima* was grouped with Pancrustacea species.

**Table S8**. **Enriched functional GO Terms for the 10 largest clusters of duplicated *S. maritima* protein-coding genes specifically expanded in the centipede lineage, as compared with the whole genome.**

**Table S9**. **Statistics regarding the duplications of centipede genes relative to seven specific ages detected using all available trees on the phylome.**

**Table S10**. **Enriched functional GO terms for proteins duplicated at the different relative ages shown in Table S9.**

Columns show relative age, gene ontology namespace, the GO term id and its name, respectively.

**Table S11. Overview of *Strigamia maritima* mitochondrial genome.**

**Table S12. Species used in the synteny analyses and the sources of their sequence data.**

**Table S13. Block-synteny summary statistics for pairs of species.**

Hs = *Homo sapiens*, Bf = *Branchiostoma floridae*, Sm = *Strigamia maritima*, Lg = *Lottia gigantea*, Ct = *Capitella teleta*, Nv = *Nematostella vectensis*, Ta = *Trichoplax adhaerens*, Ag = *Anopheles gambiae*, Bm = *Bombyx mori*.

**Table S14. Summary of numbers of homeobox genes per class of *Strigamia*, *Branchiostoma* and *Tribolium*.**

**Table S15. Names and identification numbers of all *S. maritima* homeobox genes along with their orthologues from the beetle, *T. castaneum*, and amphioxus, *B. floridae*.**

**Table S16. One-to-one *S. maritima* to human orthologues starting from genes on *S. maritima* scaffold 48457, which contains *SmaHox3a*.**

The third column is the chromosomal location of the human orthologue. Human Hox chromosomes are 2, 7, 12 and 17 and the ParaHox chromosomes are 4, 5, 13 and X.

**Table S17. Evolutionary conservation of RNA processing modes in the *S. maritima* and *D. melanogaster* Hox clusters.**

Type of RNA processing event concerning each one of the *S. maritima* (left) and *D. melanogaster* (right) Hox genes. We note that orthologous genes in both species undergo similar types of RNA processing: the three posterior-most Hox genes: *Ubx*, *abd-a* and *Abd-b* display a specific type of APA (tandem APA) in both *S. maritima* and *D. melanogaster* (conserved patterns highlighted by red asterisks) providing an example of what might be a feature present in the ancestral Hox cluster to insects and myriapods. Nonetheless, for most other Hox genes, RNA processing patterns differ markedly between *S. maritima* and *D. melanogaster,* indicating that the conserved incidence of alternative RNA processing across arthropods can only be proposed for the posterior-most Hox genes.

**Table S18. Details of SmGr family genes and proteins.**

Columns are: Gene – the gene and protein name we are assigning (suffixes are PSE – pseudogene, FIX – assembly was repaired; JOI – gene model spans scaffolds); OGS – the official gene number in the 13,233 proteins (prefix is Smar_temp_); Scaffold – the genome assembly scaffold ID, prefix is scf718000 (amongst 14,739 scaffolds in assembly Smar05272011); Coordinates – the nucleotide range from the first position of the start codon to the last position of the stop codon in the scaffold; Strand – + is forward and - is reverse; Introns – number of introns; ESTs – presence of an EST contig with appropriate splicing in one of the three transcriptome assemblies (F - female, M - male, E - eggs); AAs – number of encoded amino acids in the protein; Comments – comments on the OGS gene model, repairs to the genome assembly, and pseudogene status (numbers in parentheses are the number of obvious pseudogenizing mutations).

**Table S19. Total numbers of biogenic amine receptors in different species.**

**Table S20. A comparison between the *D. melanogaster* and *S. maritima* biogenic amine receptors.**

The orthologues are given next to each other. When there is no orthologue, a dash (–) is written instead.

**Table S21. Genes encoding neuropeptide precursors and neuropeptide receptors annotated in *S. maritima*.**

Abbreviations: ACP, adipokinetic hormone/corazonin-related neuropeptide; AKH, adipokinetic hormone; ADF, antidiuretic factor; AST, allatostatin; CCAP, crustacean cardio-active peptides; DH (Calc.-like), calcitonin-like diuretic hormone; DH (CRF-like), corticotropin releasing factor-like diuretic hormone; EH, eclosion hormone; ETH, ecdysis triggering hormone; GPA2, glycoprotein hormone A2; GPB5, glycoprotein hormone B5; ILP, insulin-like peptide; ITP, ion transport peptide; NPF, neuropeptide F; NPLP, neuropeptide-like precursor; PDF, pigment dispersing factor; PTTH, prothoracicotropic hormone; sNPF, short neuropeptide F.

**Table S22. Presence or absence of neuropeptide signaling systems in arthropods.**

The centipede *S. maritima* contains two CCHamide-1, two eclosion hormone and two FMRFamide genes (2 p). In some cases neuropeptide precursors could not be identified, but the corresponding receptor genes are present (R). We assume that this is due to sequencing gaps. For abbreviations see Table S21.

**Table S23. Genes commonly implicated in arthropod juvenoids biosynthesis (green) and degradation (blue), and their potential regulators (purple)**[98 - 101].

Common abbreviations, and presence in the centipede *S. maritima*.

**Table S24. List of genes commonly implicated as potential regulators of arthropod juvenoids biosynthesis (purple)**[98 - 101].

Common abbreviations, and presence in the centipede *S. maritima.*

**Table S25. Wnt genes in the genome of *S. maritima*.**

SMAR = the gene identification number, and scaffold = the scaffold identification number. Wnt 1, 6 and 10 are clustered together on the same scaffold (yellow highlighting), which is likely a remnant of the ancestral wnt gene cluster (see text for details).

**Table S26. Selenoproteins in the *S. maritima* genome.**

**Table S27. Histone encoding loci of *S. maritima*.**

**Table S28. Number of loci within the genomes of arthropod species encoding the five classes of histones.**

Orthologues for *A. aegypti, D. pulex, T. urticae and I. scapularis* were obtained by BLAST analysis. Orthologues for *A. mellifera* and A*. pisum* were obtained from published literature [108, 109].

**Table S29. Germ line and RNAi genes annotated in the *S. maritima* genome.**

The name of the *Drosophila* orthologue is shown unless indicated with “(Mo)”, for mouse.

**Table S30. Details of the manually annotated genes of *S. maritima*.**

**18. Supporting Information data files.**

**File S1.** One2One_GOTerms_GenomeIDs for Orthology-based functional annotation.

**File S2.** Strigamia_pals for Figure 3.

**File S3.** Gustatory receptor sequences.

**File S4.** Raw data for Figure 2, Figure 9, Figure S1 and Figure S5.

**File S5.** Raw data for Figure S28.

**File S6.** Raw data for Figure S29.

**File S7.** Raw data for Figure S30.
