# Supplementary material for: The First Myriapod Genome Sequence Reveals Conservative Arthropod Gene Content and Genome Organisation in the Centipede Strigamia maritima
Source: PLoS Biol. 2014 Nov 25;12(11):e1002005. doi: 10.1371/journal.pbio.1002005 (PMC4244043; doi:10.1371/journal.pbio.1002005)
Supplement: Figure S8 — Phylogenetic analysis of ANTP class homeodomains of S. maritima (Sma) using T. castaneum (Tca) and B. floridae (Bfl) for comparison. These phylogenetic analyses were constructed using neighbour-joining with a JTT distance matrix, 1,000 bootstrap replicates (support values in black). Nodes with support equal to or above 500 in the maximum-likelihood (LG+G) analysis are in blue and nodes with posterior probabilities equal to or above 0.5 (LG+G) in the Bayesian analysis are in red. (PDF) [file pbio.1002005.s008.pdf]

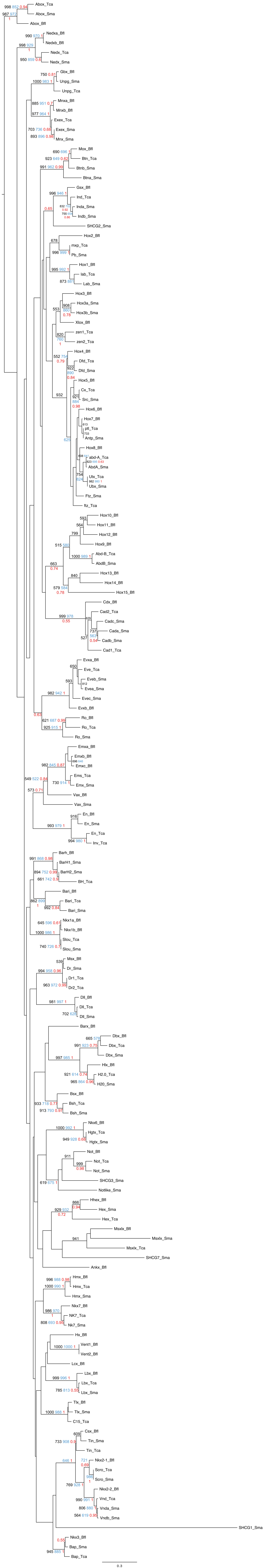
Abox_Tca
Abox_Sma
Abox_Bfl
Nedxa_Bfl
Nedxb_Bfl
Nedx_Tca
Nedx_Sma
Gbx_Bfl
Unpg_Sma
Unpg_Tca
Mnxa_Bfl
Mnxb_Bfl
Exex_Tca
Exex_Sma
Mnx_Sma
Mox_Bfl
Btn_Tca
Btnb_Sma
Btna_Sma
Gsx_Bfl
Ind_Tca
Inda_Sma
Indb_Sma
SHCG2_Sma
Hox2_Bfl
mxp_Tca
Pb_Sma
Hox1_Bfl
lab_Tca
Lab_Sma
Hox3_Bfl
Hox3a_Sma
Hox3b_Sma
Xlox_Bfl
zen1_Tca
zen2_Tca
Hox4_Bfl
Dfd_Tca
Dfd_Sma
Hox5_Bfl
Cx_Tca
Src_Sma
Hox6_Bfl
Hox7_Bfl
ptl_Tca
Antp_Sma
Hox8_Bfl
abd-A_Tca
AbdA_Sma
Utx_Tca
Ubx_Sma
Ftz_Sma
ftz_Tca
Hox10_Bfl
Hox11_Bfl
Hox12_Bfl
Hox9_Bfl
Abd-B_Tca
AbdB_Sma
Hox13_Bfl
Hox14_Bfl
Hox15_Bfl
Cdx_Bfl
Cad2_Tca
Cadc_Sma
Cada_Sma
Cadb_Sma
Cad1_Tca
Evxa_Bfl
Eve_Tca
Eveb_Sma
Evea_Sma
Evec_Sma
Evxb_Bfl
Ro_Bfl
Ro_Tca
Ro_Sma
Emxa_Bfl
Emxb_Bfl
Emxc_Bfl
Ems_Tca
Emx_Sma
Vax_Bfl
Vax_Sma
En_Bfl
En_Sma
En_Tca
Inv_Tca
Barh_Bfl
BarH1_Sma
BarH2_Sma
BH_Tca
Bari_Bfl
Bari_Tca
Bari_Sma
Nkx1a_Bfl
Nkx1b_Bfl
Slou_Tca
Slou_Sma
Msx_Bfl
Dr_Sma
Dr1_Tca
Dr2_Tca
Dll_Bfl
Dll_Tca
Dll_Sma
Barx_Bfl
Dbx_Bfl
Dbx_Tca
Dbx_Sma
Hlx_Bfl
H2.0_Tca
H20_Sma
Bsx_Bfl
Bsh_Tca
Bsh_Sma
Nkx6_Bfl
Hgtx_Tca
Hgtx_Sma
Not_Bfl
Not_Tca
Not_Sma
SHCG3_Sma
Notlike_Sma
Hhex_Bfl
Hex_Sma
Hex_Tca
Msxlx_Bfl
Msxlx_Sma
Msxlx_Tca
SHCG7_Sma
Ankx_Bfl
Hmx_Bfl
Hmx_Tca
Hmx_Sma
Nkx7_Bfl
NK7_Tca
Nk7_Sma
Hx_Bfl
Vent1_Bfl
Vent2_Bfl
Lcx_Bfl
Lbx_Bfl
Lbx_Tca
Lbx_Sma
Tlx_Bfl
Tlx_Sma
C15_Tca
Csx_Bfl
Tin_Sma
Tin_Tca
Nkx2-1_Bfl
Scro_Tca
Scro_Sma
Nkx2-2_Bfl
Vnd_Tca
Vnda_Sma
Vndb_Sma
SHCG1_Sma
Nkx3_Bfl
Bap_Sma
Bap_Tca
0.3
